# Enantioselective Copper‐Catalyzed Borylative Cyclization for the Synthesis of Quinazolinones

**DOI:** 10.1002/anie.202103259

**Published:** 2021-05-19

**Authors:** Quentin Dherbassy, Srimanta Manna, Chunling Shi, Watcharapon Prasitwatcharakorn, Giacomo E. M. Crisenza, Gregory J. P. Perry, David J. Procter

**Affiliations:** ^1^ Department of Chemistry University of Manchester Oxford Road Manchester M13 9PL UK; ^2^ School of Material and Chemical Engineering Xuzhou University of Technology Xuzhou 221018 P.R. China

**Keywords:** asymmetric, boron, copper, cyclization, nitrogen-containing heterocycles

## Abstract

Quinazolinones are common substructures in molecules of medicinal importance. We report an enantioselective copper‐catalyzed borylative cyclization for the assembly of privileged pyrroloquinazolinone motifs. The reaction proceeds with high enantio‐ and diastereocontrol, and can deliver products containing quaternary stereocenters. The utility of the products is demonstrated through further manipulations.

## Graphical abstract

The quinazolinone motif is an important feature in natural products and drug candidates although enantioselective approaches to such scaffolds remain rare. An enantioselective Cu‐catalyzed borylative cyclization delivers pyrroloquinazolines, featuring quaternary stereocenters and synthetic handles for further derivatization, with high enantio‐ and diastereocontrol.

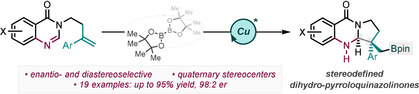

Since the seminal reports of Hosomi^[1]^ and Miyaura,^[2]^ the copper‐catalyzed borylative functionalization of olefins has emerged as a powerful method for stereocontrolled, complex molecule construction.^[3]^ Subsequent studies by Ito and Sawamura,^[4]^ and others,^[5]^ have shown the utility of this process in cyclization reactions. In particular, several groups have used this strategy to construct valuable nitrogen‐containing heterocycles, such as indolines^[6]^ and tetrahydroquinolines^[7]^ (Scheme 1 A). In particular, Lautens has recently described a copper‐catalyzed stereoselective synthesis of tetrahydroquinolines through a conjugate borylation/Mannich cyclization cascade.^[7a]^ This process illustrates the potential of copper‐catalyzed borylative cyclizations by: 1) forming several stereocentres with high control; 2) incorporating a boron group that can undergo further derivatization; 3) preparing an important class of nitrogen‐containing heterocycle, in this case tetrahydroquinolines

**Scheme 1:**
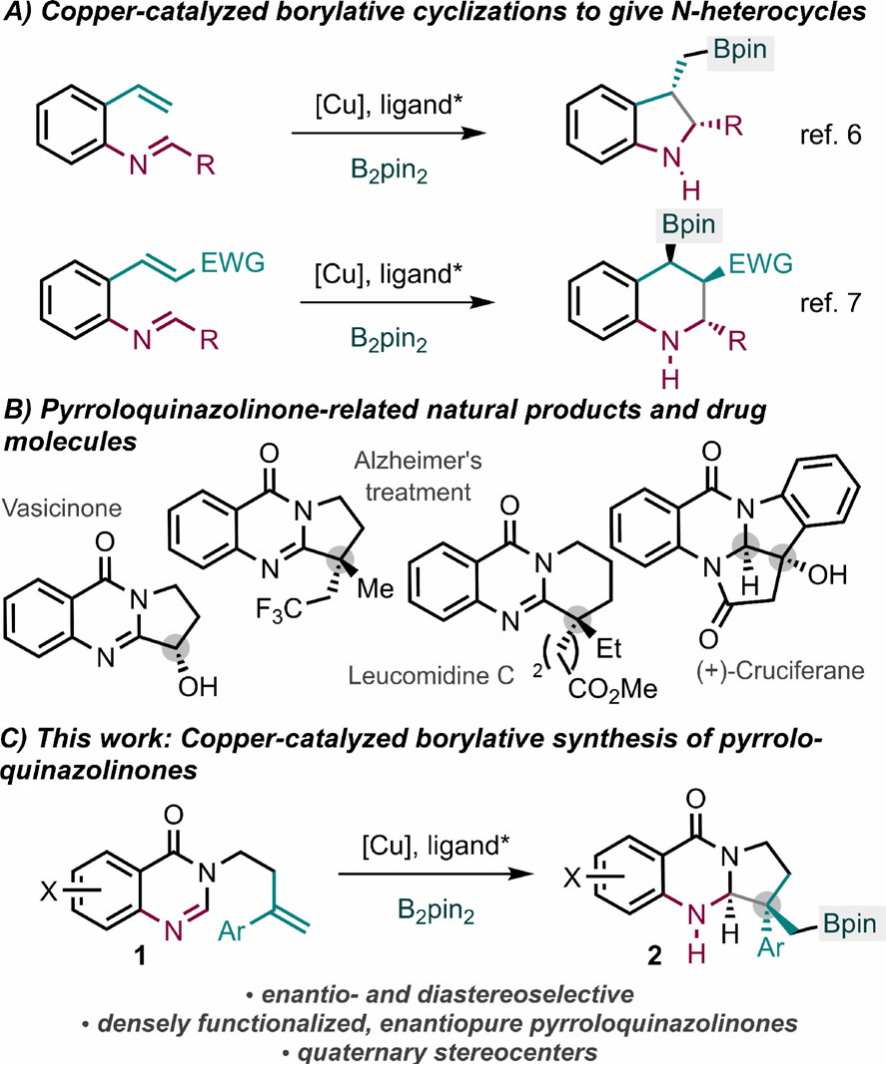
Copper‐catalyzed borylative cyclizations for the enantioselective synthesis of N‐heterocycles. A) Enantioselective approaches to indolines and tetrahydroquinolines. B) Biologically active pyrroloquinazolinones. C) Enantioselective, copper‐catalyzed borylative synthesis of pyrroloquinazolinones.

Quinazolinones display important bioactivity.^[8]^ In particular, pyrroloquinazolinones are common tricyclic motifs found in drug molecules and natural products (Scheme 1 B). It is important to prepare these compounds enantioselectively as quinazolinone enantiomers can display different bioactivities.^[9]^ Few current methods for the construction of quinazolinones are enantioselective,[^8^, ^10^] and classical chiral resolution and chiral pool synthesis are typically used, for example, to access the enantiopure quinazolinones shown in Scheme 1 B.^[11]^ More recently, dihydroquinazolinones have been prepared enantioselectively, typically from 2‐aminobenzamide and aldehydes,^[12]^ however, few enantioselective methods extend to the delivery of important pyrroloquinazolinone scaffolds.^[12h]^ Thus, new enantioselective approaches to pyrroloquinazolinone building blocks are needed for the synthesis of known and as yet unknown bioactive targets.

We recognized that the enantioselective, copper‐catalyzed borylative cyclizations of substrates **1**, involving intramolecular addition of an organocopper intermediate to a C=N electrophile,^[3a]^ would constitute a valuable route to important enantiomerically enriched pyrroloquinazolinone derivatives **2** (Scheme 1 C). The resulting new process is highly enantio‐ and diastereoselective, uses an inexpensive and non‐toxic catalyst, and exploits commercially available chiral ligands. Furthermore, through subsequent derivatization, a variety of potentially bioactive quinazolinones can be accessed.

We initially examined the borylative cyclization of substrate **1 a** using CuCl and ligand Ph‐BPE (**L1**, Table 1). Although this ligand is commonly used in related copper‐catalyzed functionalizations, its use here proved ineffective (Table 1, entry 1).^[13]^ Fortunately, screening of other phosphine ligands (Table 1, entries 1–3) revealed both ligands **L2** ((*S*,*S*)‐BDPP)[^5f^, ^5g^, ^5i^] and **L3** ((*R*)‐QuinoxP^®^)[^4a^, ^4b^, ^5j^] gave the product **2 a** with encouraging enantiocontrol, albeit in moderate yield. Additional phosphine and NHC ligands that have been used in previous borylative functionalizations were unsuccessful here (See Supporting Information).^[14]^ We then tested copper sources, bases and solvents (Table 1, entries 4–6) and found Cu(MeCN)_4_PF_6_ with KO*t*Bu in THF to be optimal (Table 1, entry 6).

**Table 1:** Reaction optimization.^[a]^ 
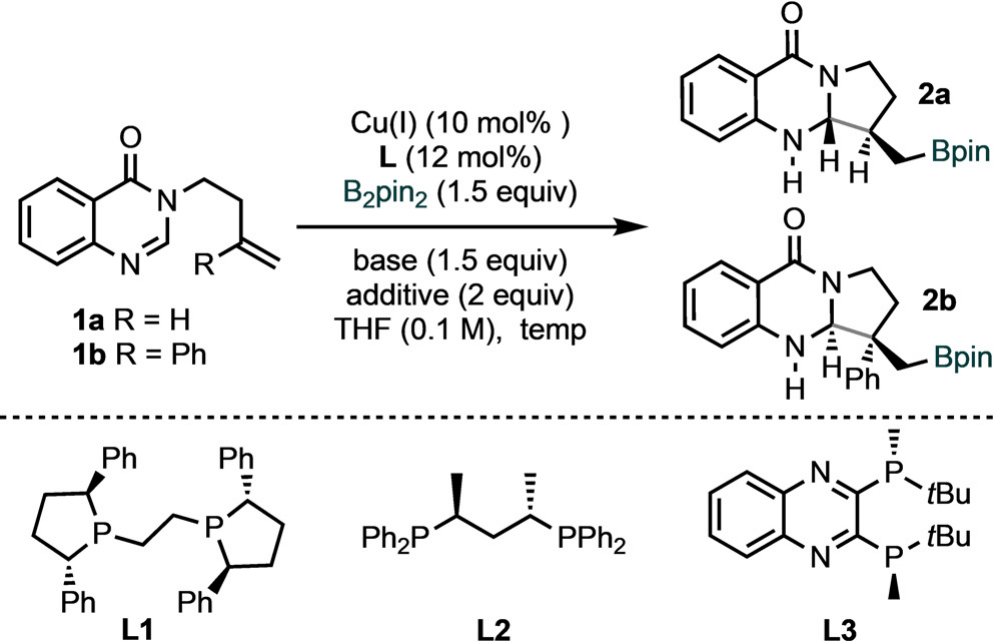

Entry	Cu^I^	Additive	Ligand	Yield	dr	er
1^[b,c]^	CuCl	–	**L1**	10	83:17	64:26
2^[b,c]^	CuCl	–	**L2**	51	>95:5	83:17
3^[b,c]^	CuCl	–	**L3**	15	>95:5	79:21
4^[b,d]^	CuCl	–	**L3**	10	>95:5	98:2
5^[b,d,e]^	CuCl	–	**L3**	11	>95:5	95:5
6^[b,d]^	Cu(MeCN)_4_PF_6_	–	**L3**	35	>95:5	94:6
7^[b,d]^	Cu(MeCN)_4_PF_6_	*t*BuOH	**L3**	85	>95:5	84:16
8^[b,d]^	Cu(MeCN)_4_PF_6_	*i*PrOH	**L3**	82	>95:5	93:7
9^[d,f]^	Cu(MeCN)_4_PF_6_	*i*PrOH	**L3**	87	80:20	58:42
10^[d,f]^	Cu(MeCN)_4_PF_6_	*i*PrOH	**L2**	75	91:9	95:5
11^[b,d]^	Cu(MeCN)_4_PF_6_	*i*PrOH	**L2**	10	87:13	95:5

[a] For further details of the reaction optimization, see the Supporting Information. Reaction conditions: **1** (0.2 mmol), B_2_pin_2_ (0.3 mmol), Cu^I^ (10 mol %), ligand (12 mol %) in THF (2.0 mL) at 25 °C or 35 °C for 2–6 h under nitrogen. The diastereoselectivity was determined by ^1^H NMR analysis of the crude product mixtures. NMR yields are given. [b] With **1 a** to give **2 a**. [c] Using NaO*t*Bu (1.5 equiv). [d] Using KO*t*Bu (1.5 equiv). [e] Using dioxane (0.2 m). [f] With **1 b** to give **2 b**.

Interestingly, the addition of alcohols greatly influenced the yield of the process (Table 1, entries 7 and 8), and **2 a** was isolated in high yield, with excellent diastereo‐ and enantiocontrol (Table 1, entry 8). The exact role of the alcohol in this process remains unclear although it may facilitate catalyst turnover by protonation of a copper–amide intermediate to deliver product and regenerate a copper alkoxide.^[15]^ Finally, we tested the phenyl‐substituted substrate **1 b** under our optimized conditions (Table 1, entries 9 and 10). Although ligand **L3** was unsuccessful (Table 1, entry 9), the product **2 b** was isolated in excellent yield and with very high diastereo‐ and enantiocontrol when using ligand **L2** (Table 1, entry 10). Exposing substrate **1 a** to the latter conditions gave **2 a** in substantially reduced yield (Table 1, entry 11).

We next explored the performance of various aryl‐substituted alkenes **1 b**–**1 k** in the process (Scheme 2). In almost all cases, borylative cyclization and construction of two adjacent stereocentres—including a quaternary stereocentre—proceeded efficiently to deliver pyrroloquinazolinones **2 b**–**k** with very good to excellent enantio‐ and diastereocontrol. For example, aryl groups bearing electron‐rich substituents at both *meta*‐ and *para*‐positions gave products with very high enantiocontrol (**2 c**–**2 f**). *Ortho*‐, *meta*‐ and *para*‐halogenated aryl groups were also well tolerated (**2 g**–**2 i**). Finally, substrates bearing 2‐napthyl and 2‐thienyl groups gave the desired products in high yield and with excellent enantiocontrol (**2 j**, **2 k**). Additional substrates bearing heteroaryl groups gave rise to unstable products (see Supporting Information). The relative and absolute stereochemistry of the products was determined by X‐ray crystallographic analysis of a derivative of **2 e** and **2 f**.^[16]^

**Scheme 2:**
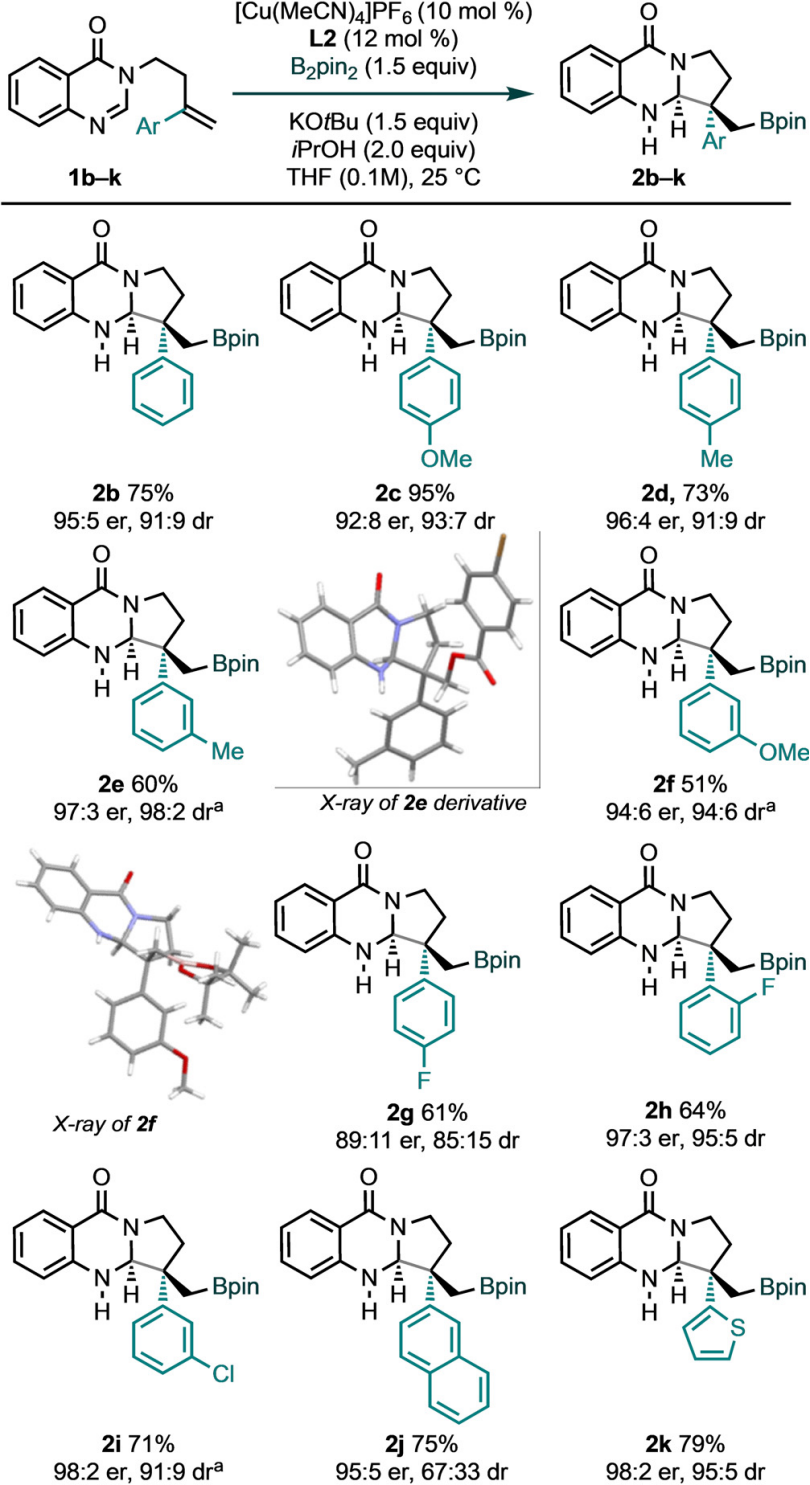
Scope with respect to the alkene. Reaction conditions: **1** (0.2 mmol), B_2_pin_2_ (0.3 mmol), [Cu(MeCN)_4_]PF_6_ (0.02 mmol), **L2** (0.024 mmol), KO*t*Bu (0.3 mmol in 1 m sol. THF), *i*PrOH (0.4 mmol) in THF (1.7 mL) at 25 °C for 2–4 h under nitrogen. Yields of isolated product are given. The diastereoselectivity was determined by ^1^H NMR analysis of the crude products and er values were measured by HPLC on chiral stationary phase. [a] Reaction run at 0 °C.

Various substitution on the aryl ring of the amidine component of **1** was also tolerated (Scheme 3). For example, the methyl‐ and fluorine‐containing products **2 l** and **2 m** were obtained in high yield and with good to excellent enantiocontrol. A thiophene‐fused substrate was also compatible with our standard conditions to give **2 n** with moderate enantiocontrol. Building on our initial optimization (Table 1, entry 8), we investigated the scope of the process with additional monosubstituted alkene substrates **1 o**–**r**. The product **2 o** was obtained in high yield and with excellent diastereo‐ and enantiocontrol, thus suggesting that electron‐rich substrates are particularly well‐suited to the process. Halogenated substrates were also tested (**2 p**–**2 r**); borylative cyclization proceeded well, albeit with lower enantiocontrol for substrates **1 q** and **1 r**. The relative and absolute stereochemistry of the products **2 a**, **2 o**–**2 r** was assigned after X‐ray crystallographic analysis of a derivative of **2 a**.^[16]^ Substrates bearing substitution at the terminus of the alkene proved unreactive (see Supporting Information).

**Scheme 3:**
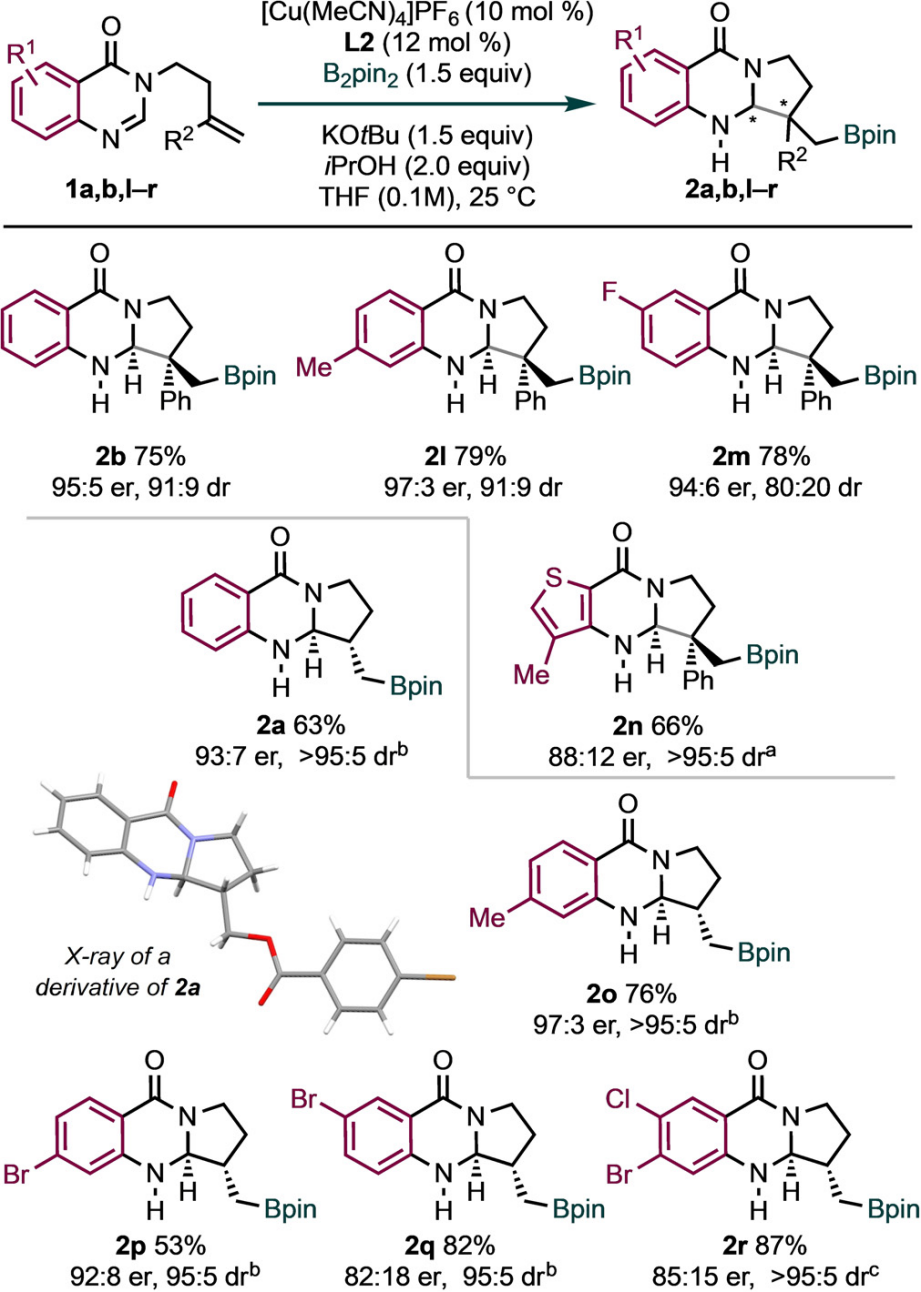
Scope with respect to the amidine. Reaction conditions: **1** (0.2 mmol), B_2_pin_2_ (0.3 mmol), [Cu(MeCN)_4_]PF_6_ (0.02 mmol), **L2** (0.024 mmol), KO*t*Bu (0.3 mmol in 1 m sol. THF), *i*PrOH (0.4 mmol) in THF (1.7 mL) at 25 °C or 30 °C for 2–4 h under nitrogen. Yields of isolated product are given. The diastereoselectivity was determined by ^1^H NMR analysis of the crude products. *ee* values were measured by HPLC on chiral stationary phase. [a] Reaction was run without *i*PrOH. [b] B_2_pin_2_ (0.4 mmol), KO*t*Bu (0.4 mmol in 1 m sol. THF) and (*R*)‐QuinoxP^®^ **L3** (0.024 mmol) were used. [c] (*R*,*R*)‐(−)‐2,3‐bis(*tert*‐Butylmethylphosphino)benzene (BenzP*; 0.024 mmol) was used as a ligand.

The functionality in the dihydroquinalozinone products **2** presents opportunities for further transformations (Scheme 4). The material (**2 b**) for these transformations was obtained by performing the enantioselective, borylative cyclization on a gram‐scale; essentially identical yield, enantio‐ and diastereocontrol were observed (c.f. Table 1, entry 10). We first converted product **2 b** into the trifluoroborate salt **3**,^[17]^ and the alcohol **4**; the latter by oxidation with H_2_O_2_. Methylation of the free amine group was also carried out to give product **5**. Finally, oxidation with DDQ provided pyrroloquinazolinone product **6**. It is noteworthy that judicious choice of oxidant (H_2_O_2_ or DDQ) leads selectively to either product **4** or **6**. Products related to **6** are common in medicine (Scheme 1 B)^[8]^ and our preparation of **6** represents a rare example of an enantioselective approach to this class of compound.

**Scheme 4:**
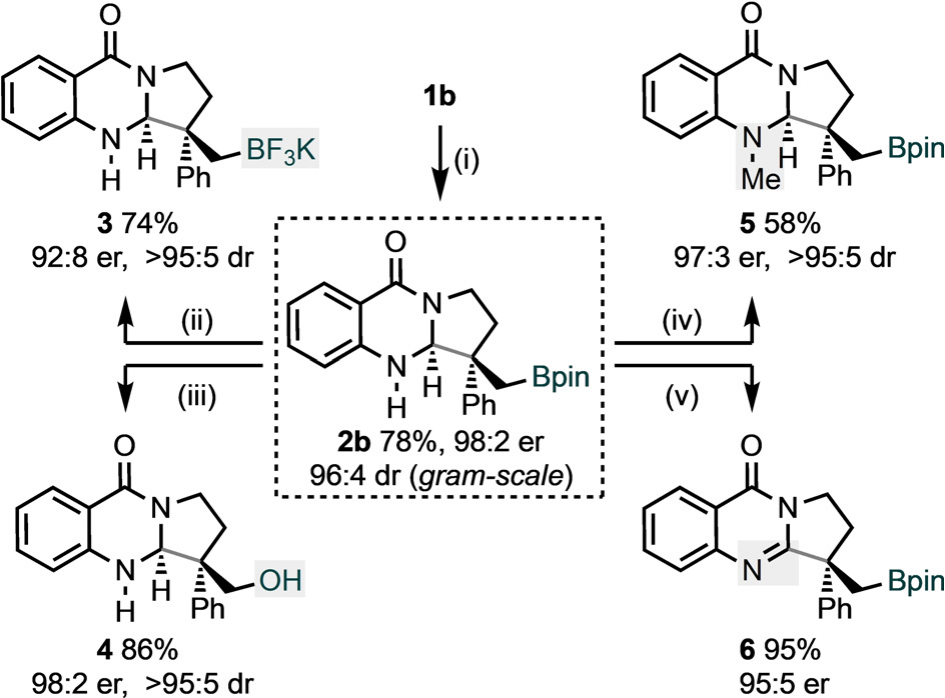
Gram‐scale reaction and derivatizations of **2 b**. Conditions: (i) see Scheme 2; (ii) KHF_2_ 4 equiv, MeOH/H_2_O, 0 °C to RT; (iii) H_2_O_2_ 2 equiv, K_2_CO_3_ 2 equiv, THF, −20 °C; (iv) NaH 1.5 equiv, MeI 1.5 equiv, THF, 0 °C to RT; (v) DDQ 1.5 equiv, CH_2_Cl_2_, 0 °C to RT.

We propose a tentative mechanism and stereochemical model to rationalize the observed outcome of the cyclization of aryl‐substituted alkene substrates **1 b** (Scheme 5). Upon formation of copper–boryl species **II**, enantioselective borocupration occurs across the double bond of the alkene to give **III**. Our stereochemical model (Scheme 5 B) suggests this addition occurs with the smaller methylene group (R) oriented towards the ligand P‐aryl ring, rather than the larger phenyl group on the substrate (**TS‐1 a** vs. **TS‐1 b**). Based on previous reports, a favourable face‐to‐face interaction between the phenyl group on the alkene of the substrate and the P‐aryl ring might further stabilize **TS‐1 a**, whereas unfavourable edge‐to‐face interactions might be present in **TS‐1 b**.^[18]^ The diastereoselective, C−C bond‐forming cyclization of **III** can then proceed via **TS‐2** to give the intermediate **IV**. We suggest that copper coordinates to the nitrogen atom during this step, in agreement with previous reports.^[19]^ Finally, in line with the positive influence of alcohols on reactivity, we suggest that R′OH (R′=*i*Pr, *t*Bu) protonates intermediate **IV** to give the desired product **2 b** and regenerate the active copper alkoxide catalyst **I**.

**Scheme 5:**
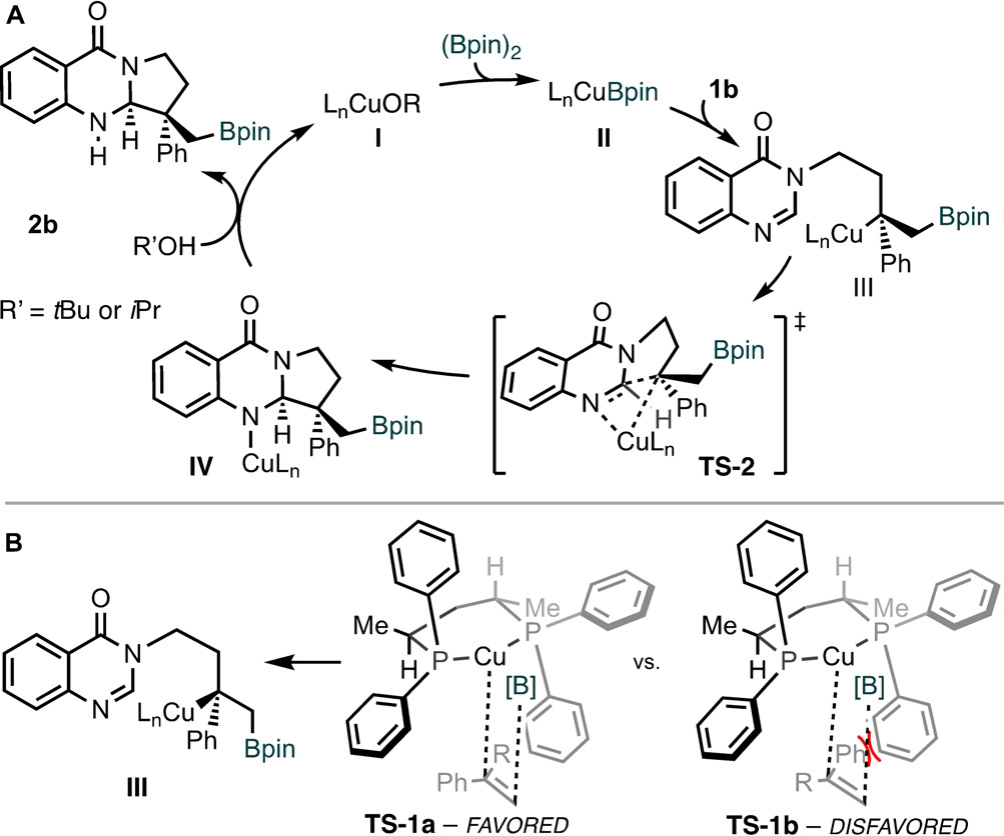
Proposed catalytic cycle and model for the origin of stereocontrol.

A highly enantio‐ and diastereoselective copper‐catalyzed borylative cyclization constructs two adjacent stereocentres—including a quaternary stereocentre—and delivers a range of pyrroloquinazolinone derivatives that are currently difficult to access. The new process exploits an inexpensive and non‐toxic copper catalyst and commercially available chiral phosphine ligands. Selective manipulation of the products allows access to enantiomerically enriched quinazolinones of medicinal relevance.

## Conflict of interest

The authors declare no conflict of interest.

## Supporting information

As a service to our authors and readers, this journal provides supporting information supplied by the authors. Such materials are peer reviewed and may be re‐organized for online delivery, but are not copy‐edited or typeset. Technical support issues arising from supporting information (other than missing files) should be addressed to the authors.

Supplementary
