# Supplementary material for: Enantioselective Copper‐Catalyzed Borylative Cyclization for the Synthesis of Quinazolinones
Source: Angew Chem Int Ed Engl. 2021 May 19;60(26):14355–9. doi: 10.1002/anie.202103259 (PMC8252434; doi:10.1002/anie.202103259)
Supplement: Supplementary file 1 — Supplementary [file ANIE-60-14355-s001.pdf]

Angewandte Chemie
Eine Zeitschrift der Gesellschaft Deutscher Chemiker

Supporting Information

Enantioselective Copper-Catalyzed Borylative Cyclization for the Synthesis of Quinazolinones

*Quentin Dherbassy+, Srimanta Manna+, Chunling Shi, Watcharapon Prasitwatcharakorn, Giacomo E. M. Crisenza, Gregory J. P. Perry, and David J. Procter**

anie_202103259_sm_miscellaneous_information.pdf

Table of Contents

**General Information**	**S3**
**General procedures**	**S3**
**Optimization studies**	**S6**
**Unsuccessful substrates**	**S8**
**Characterization and NMR data for substrates 1**	**S9**
**Characterization and NMR data for products 2**	**S35**
**Data for gram-scale reaction and product manipulation (compounds 3-6)**	**S84**
**X-ray crystallographic data**	**S97**
**References**	**S101**

General Information

All experiments were performed under an atmosphere of nitrogen, using anhydrous solvents, unless stated otherwise. THF was distilled from sodium / benzophenone. ^{1}H, ^{13}C, ^{19}F and ^{11}B NMR spectra were recorded using 400 and 500 MHz spectrometers, with chemical shift values being reported in ppm relative to residual chloroform (δ_H = 7.26 or δ_C = 77.0) as internal standards. All coupling constants (*J*) are reported in Hertz (Hz). Mass spectra were obtained using positive and negative electrospray ($ES^{\pm}$), atmospheric-pressure chemical ionization (APCI) or gas chromatography (GC) methodology. Infra-red spectra were recorded as evaporated films or neat using a FT/IR spectrometer. Column chromatography was carried out using 40 – 63 µm, 60 Å silica gel. Routine TLC analysis was carried out on aluminium sheets coated with silica gel 60 F254, 0.2 mm thickness and plates were viewed using a 254 nm ultraviolet lamp and dipped in aqueous potassium permanganate or *p*-anisaldehyde. Melting points were measured on a melting point apparatus and are uncorrected.

General Procedures

General procedure A *for the synthesis N-(alkenyl)quinazolinones under Mitsunobu conditions.*

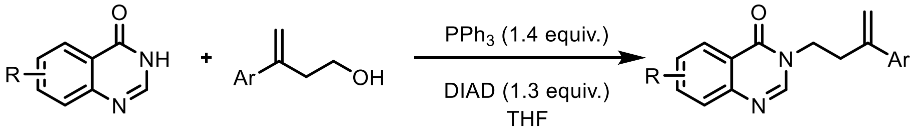

These compounds were synthesised according to a reported literature procedure.[1] To a 12 mL reaction vial was added 4-hydroxyquinazoline (1 mmol), 3-aryl-3-buten-1-ol derivatives (1.1 mmol), triphenylphosphine (1.4 mmol), diisopropyl azodicarboxylate (1.3 mmol) and THF (4 mL). The mixture was stirred at room temperature for 16-24 h and, then, treated with a solution of KOH (3 mmol) in water (3 mL) and methanol (3 mL). The resulting mixture was stirred at 70 °C for 6 h. After completion, the reaction was cooled to room temperature and the mixture was concentrated in vacuo. The reaction mixture was extracted with EtOAc, and the combined organic layer was washed with water and brine. The organic layer was dried over anhydrous magnesium sulfate, filtered and evaporated to dryness under vacuum. Purification of the crude material by column chromatography (EtOAc/hexanes) gave the desired product.

General procedure B *for the synthesis N-(alkenyl)quinazolinones using bromoalkene derivatives.*

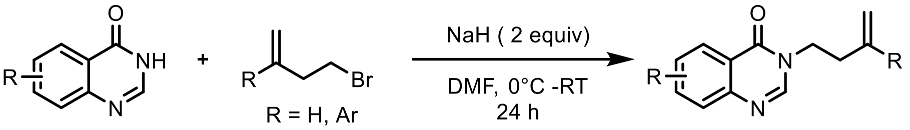

To a solution of quinazolinone (1-5 mmol) in DMF (0.25 M), at 0°C and under nitrogen atmosphere, was added NaH (*60% dispersion in mineral oil*, 2 equiv.), portion-wise. The mixture was stirred at 0°C for 15-20 min and, then, the corresponding alkenyl bromide (1.3 equiv.) was added dropwise. The mixture was warmed to room temperature and stirred for 12-24 h. The reaction was quenched with H_2O and the crude mixture extracted with EtOAc. The combined organic layer was washed with water and brine. The organic layer was dried over anhydrous magnesium sulfate, filtered and concentrated *in vacuo* to give the crude material. Purification of the latter by column chromatography on silica gel (EtOAc/hexanes) gave the desired product.

General procedure C *for the enantioselective Cu-catalyzed borylative cyclization of N-(3-aryl-but-3-en-1-yl)quinazolinones.*

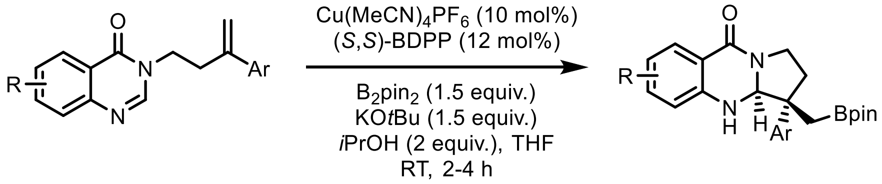

To a 10 mL reaction vial was added $Cu(MeCN)_4PF_6$ (0.02 mmol, 7.4 mg, 10 mol %), (2S,4S)-2,4-bis(diphenylphosphino)pentane (0.024 mmol, 10.6 mg), KO*t*Bu (0.3 mmol, 0.3 mL in 1 M THF solution) and THF (1.0 mL). The mixture was stirred at room temperature for 10-15 min before the addition of B_2pin_2 (0.3 mmol, 78.0 mg). After brief stirring (10 min), a solution of amidine (0.2 mmol) in anhydrous THF (0.7 mL) was then added at room temperature under nitrogen atmosphere. Degassed and dry isopropanol (0.4 mmol, 30 µL) was added under nitrogen atmosphere and the reaction mixture was then stirred at the temperature indicated for each entry for 2-4 h. After reaction completion, the resulting solution was filtered through a silica plug, and the crude material was concentrated in vacuo. ^1H NMR analyses were used to determine both crude NMR yield and diastereoselectivity using 1,3,5-trimethoxybenzene (0.2 mmol, 33.6 mg) as the internal standard. After NMR analysis, the combined crude material

was concentrated under vacuum. Column chromatography of the crude material, under the conditions specified for each case, afforded the target compounds.

General procedure D *for the enantioselective Cu-catalyzed borylative cyclization of N-(but-3-en-1-yl)quinazolinones.*

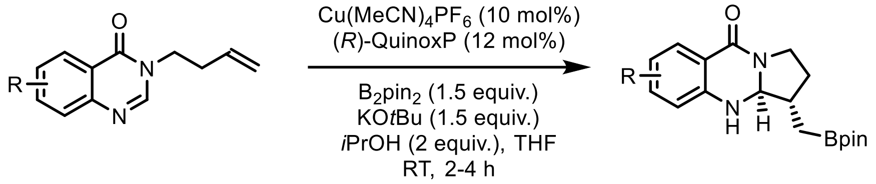

To a 10 mL reaction vial was added $Cu(MeCN)_4PF_6$ (0.02 mmol, 7.4 mg, 10 mol %), (*R*,*R*)-(–)-2,3-bis(*tert*-butylmethylphosphino)quinoxaline (0.024 mmol, 8.0 mg), KO*t*Bu (0.3 mmol, 0.3 mL in 1 M THF solution) and THF (1.0 mL). The mixture was stirred at room temperature for 10-15 min before the addition of B_2pin_2 (0.3 mmol, 78.0 mg). After brief stirring (10 min), solution of amidine (0.2 mmol) in anhydrous THF (0.7 mL) was then added at room temperature under nitrogen atmosphere. Degassed and dry isopropanol (0.4 mmol, 30 µL) was added under nitrogen atmosphere and the reaction mixture was then stirred at the temperature indicated for each entry for 2-4 h. After reaction completion, the resulting solution was filtered through a silica plug, and the crude material was concentrated in vacuo. ^{1}H NMR analyses were used to determine both crude NMR yield and diastereoselectivity using 1,3,5-trimethoxybenzene (0.2 mmol, 33.6 mg) as the internal standard. After NMR analysis, the combined crude material was concentrated under vacuum. Column chromatography of the crude material, under the conditions specified for each case, afforded the target compounds.

*The racemic mixtures for products **2** were obtained performing the corresponding Cu-catalyzed borylative cyclisation reaction, according to the abovementioned procedures, but using* Xantphos (0.024 mmol, 13.9 mg)*, as the ligand.*

Optimization studies

Table 1. Optimization studies with ligands

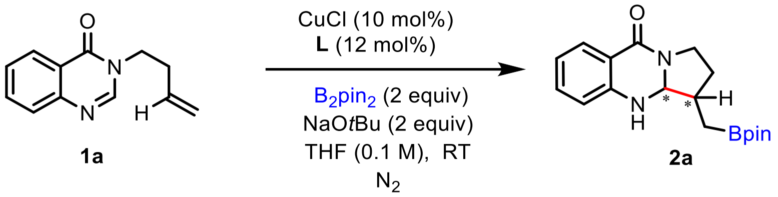

Entry	Ligand	NMR yield (%)	d.r.	er
1	**L1**	10	83:17	69:31
2	**L2**	51	>95:5	83:17
3	**L3**	15	>95:5	79:21
4	**L4**	0	-	-
5	**L5**	0	-	-
6	**L6**	20	>95:5	88:22
7	**L7**	24	83:17	63:37
8	**L8**	0	-	-
9	**L9**	0	-	-
10	**L10**	0	-	-
11	**L11**	0	-	-
12	**L12**	0	-	-

Reaction conditions: **1a** (0.2 mmol), B_2pin_2 (0.4 mmol), CuCl (10 mol%), bisphosphine ligand (12 mol%) and NaO*t*Bu (0.4 mmol in 2 M THF solution) in THF (0.1 M) at room temperature for 16 h under nitrogen. The diastereoselectivity and yield were determined by ^{1}H NMR analysis of the crude products. The er values were measured by chiral HPLC.

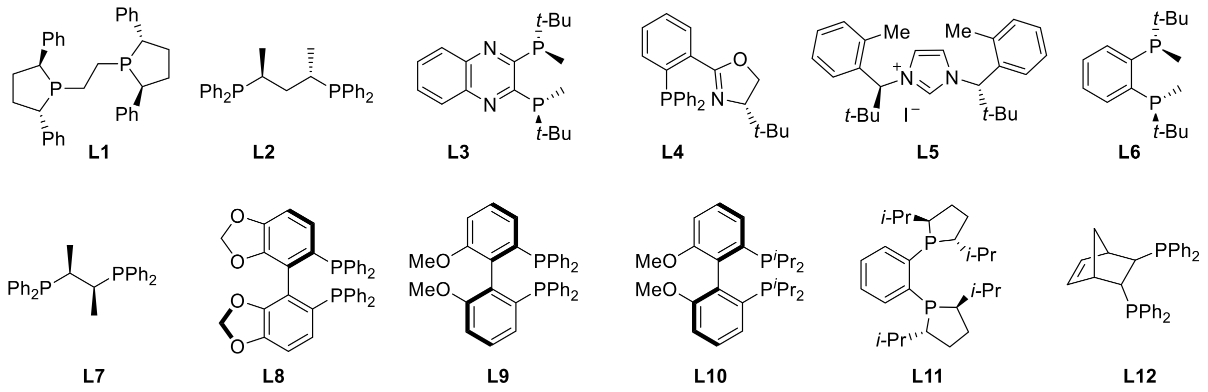

Table 2. Optimization studies with solvents and bases

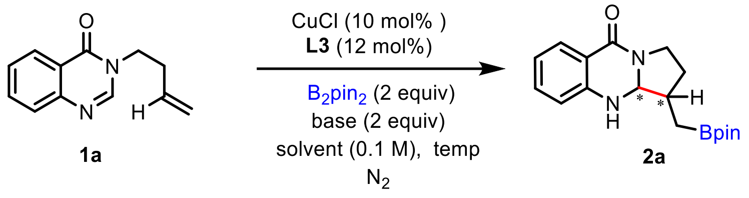

Entry	Solvent	Base (equiv)	Temp (˚C)	NMR Yield (%)	d.r.	er
1	THF	NaO*t*Bu (2)	RT	15	>95:5	79:21
2	Toluene	NaO*t*Bu (2)	35˚C	-	>95:5	-
3	THF:MTBE (1:1)	NaO*t*Bu (2)	35˚C	10	>95:5	82:18
4	THF	NaO*t*Bu (2)	35˚C	65	>95:5	87:13
5[a]	THF	LiO*t*Bu (3)	RT	85	>95:5	84:16
**6**	**THF**	**KO*t*Bu (2)**	**RT**	**11 (10)[b]**	**>95:5**	**98:2**
7	Dioxane	KO*t*Bu (2)	RT	11	>95:5	95:5
8	Dioxane	KO*t*Bu (2)	35˚C	46[c]	>95:5	80:20
9	THF	KO*t*Bu (2)	35˚C	51	>95:5	88:12
10	DMA	KO*t*Bu (2)	35˚C	trace	-	-

Reaction conditions: **1a** (0.2 mmol), B_2pin_2 (0.4 mmol), CuCl (10 mol%), bisphosphine ligand (12 mol%) and NaO*t*Bu (0.4 mmol in 2 M THF solution) in THF (0.1 M) at room temperature for 16 h under nitrogen atmosphere. The diastereoselectivity and yield were determined by ^{1}H NMR analysis of the crude products. The er values were measured by chiral HPLC. [a] 2 equiv. *t*BuOH was used; [b] Repeated reaction; [c] isolated yield reported.

Table 3. Optimization studies with Cu-salts

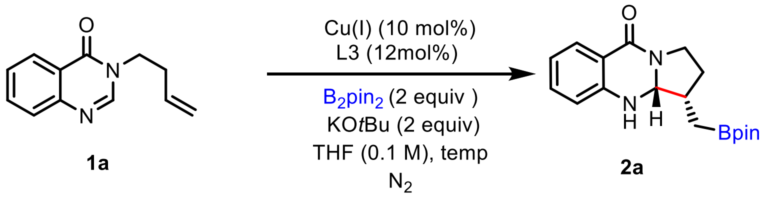

Entry	Cu(I)-Salt	Temp (˚C)	NMR Yield (%)	d.r.	er
1	CuCl	RT	15	>95:5	79:21
2	CuCl	35 ˚C	63	>95:5	89:11
3	CuTC	35 ˚C	33	>95:5	89:11
4	$Cu(MeCN)_4OTf$	35 ˚C	43	>95:5	88:12
5	$Cu(MeCN)_4PF_6$	35 ˚C	63	>95:5	88:12
6	$Cu(MeCN)_4PF_6$	45 ˚C	58	>95:5	87:13
**7**	**$Cu(MeCN)_4PF_6$**	**25 °C**	**35**	**>95:5**	**94:6**
8	$CuBr•SMe_2$	35 ˚C	trace	-	-

Reaction conditions: **1a** (0.2 mmol), B_2pin_2 (0.4 mmol), CuX (10 mol%), bisphosphine ligand (12 mol%) and KO*t*Bu (0.4 mmol in 1 M THF soln) in THF (0.1 M) at the stated temperature for 16 h under nitrogen atmosphere. The diastereoselectivity and yield were determined by ^{1}H NMR analysis of the crude products. The er values were measured by chiral HPLC.

Table 4. Optimization studies with additive

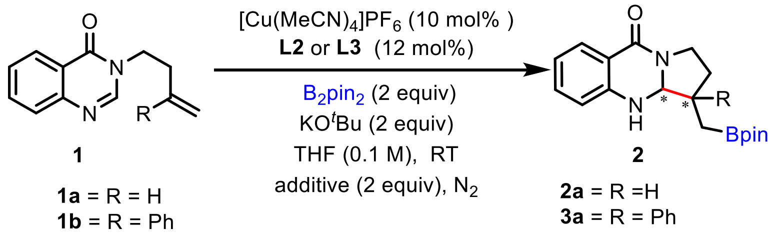

Entry	Substrate	Ligand	Additive	NMR Yield (%)	d.r	er
1	**1a**	**L3**	-	35	>95:5	94:6
2	**1a**	**L3**	*t*BuOH	85	>95:5	84:16
**3**	**1a**	**L3**	***i*PrOH**	**82**	**>95:5**	**93:7**
4	**1a**	**L3**	18-Crown-6	50	>95:5	87:13
**5[a]**	**1b**	**L2**	***i*PrOH**	**75[b]**	**91:9**	**95:5**

Reaction conditions: **1** (0.2 mmol), B_2pin_2 (0.4 mmol), $[Cu(MeCN)_4]PF_6$ (10 mol%), bisphosphine ligand (12 mol%) and KO*t*Bu (0.4 mmol in 1 M THF solution) in THF (0.1 M) at room temperature for 2-16 h under nitrogen atmosphere. The diastereoselectivity and yield were determined by ^{1}H NMR analysis of the crude products. The er values were measured by chiral HPLC. [a] KO*t*Bu (1.5 equiv) and B_2pin_2 (1.5 equiv) were used; [b] isolated yield reported.

Unsuccessful substrates

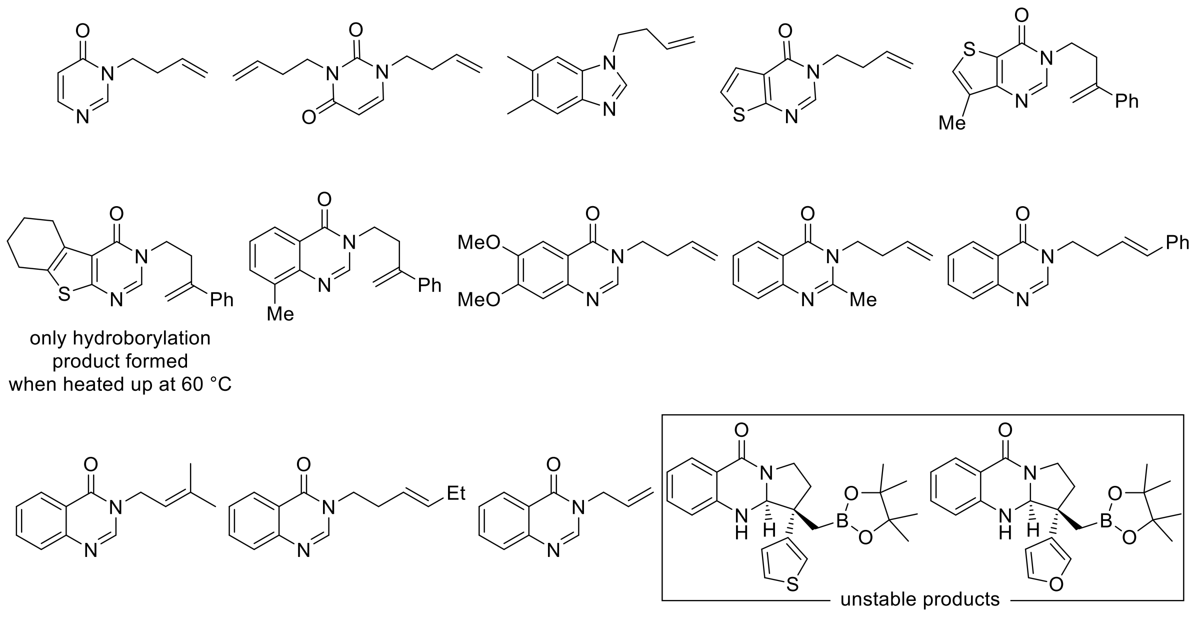

Characterization and NMR data for substrates 1

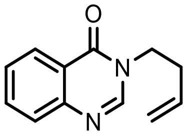

3-(But-3-en-1-yl)quinazolin-4(3*H*)-one (1a)

The title compound was synthesised following General Procedure B. **1a** was obtained in 90% yield (5 mmol scale, 4.5 mmol, 901 mg, 90%).

^{1}H NMR (400 MHz, $CDCl_3$) δ ppm 8.37 – 8.28 (m, 1H, ArC*H*), 8.17 – 8.00 (s, 1H, ArC*H*), 7.83 – 7.69 (m, 2H, ArC*H* + NC*H*), 7.57 – 7.46 (m, 1H, ArC*H*), 5.81 (ddt, *J* = 17.3, 10.4, 7.0 Hz, 1H, CH_2=C*H*), 5.14 – 5.04 (m, 2H, CH=CH_2), 4.09 (td, *J* = 6.9, 1.9 Hz, 2H, NCH_2), 2.57 (d, *J* = 6.9 Hz, 2H, NCH_2CH_2).

^{13}C NMR (101 MHz, $CDCl_3$) δ ppm 161.2 (CON), 148.3 (ArC), 146.7 (N=CH), 134.3 (ArCH), 133.6 (ArCH), 127.6 (CH_2=*CH*), 127.4 (ArCH), 126.8 (ArCH), 122.3 (ArC), 118.8 (CH=CH_2), 46.6 (NCH_2), 33.5 (NCH_2CH_2).

The spectroscopic properties of this compound were consistent with the data available in the literature.[1]

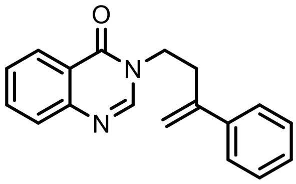

3-(3-Phenylbut-3-en-1-yl)quinazolin-4(3*H*)-one (1b)

The title compound was synthesised following General Procedure B. **1b** was obtained in 78% yield (5 mmol scale, 3.9 mmol, 1.08 g, 78%).

^{1}H NMR (500 MHz, $CDCl_3$) δ ppm 8.21 (ddd, *J* = 8.1, 1.5, 0.6 Hz, 1H, ArCH), 7.65 (ddd, *J* = 8.4, 6.9, 1.5 Hz, 1H, ArCH), 7.58 (s, 1H, NCH), 7.56 (dd, *J* = 6.9, 1.5 Hz, 1H, ArCH), 7.45 – 7.32 (m, 3H, ArCH), 7.31 – 7.15 (m, 3H, ArCH), 5.25 (d, *J* = 1.2 Hz, 1H, C=CH_2), 4.94 (d, *J* = 1.2 Hz, 1H, C=CH_2), 3.96 (t, *J* = 6.7 Hz, 2H, NCH_2), 2.95 (td, *J* = 6.7, 1.1 Hz, 2H, NCH_2CH_2).

^{13}C NMR (101 MHz, $CDCl_3$) δ ppm 161.1 (CON), 148.3 (ArC), 146.9 (N=CH), 144.1 (ArC), 139.5, (ArC) 134.3 (ArCH), 128.9 (ArCH x 2), 128.2 (ArCH), 127.5 (ArCH), 127.3 (ArCH), 126.7 (ArCH), 126.3 (ArCH x 2), 122.2 (*C*=CH_2), 116.2 (C=CH_2), 46.3 (NCH_2), 34.6 (NCH_2CH_2).

^{1}H NMR of 1b (500 MHz, $CDCl_3$)

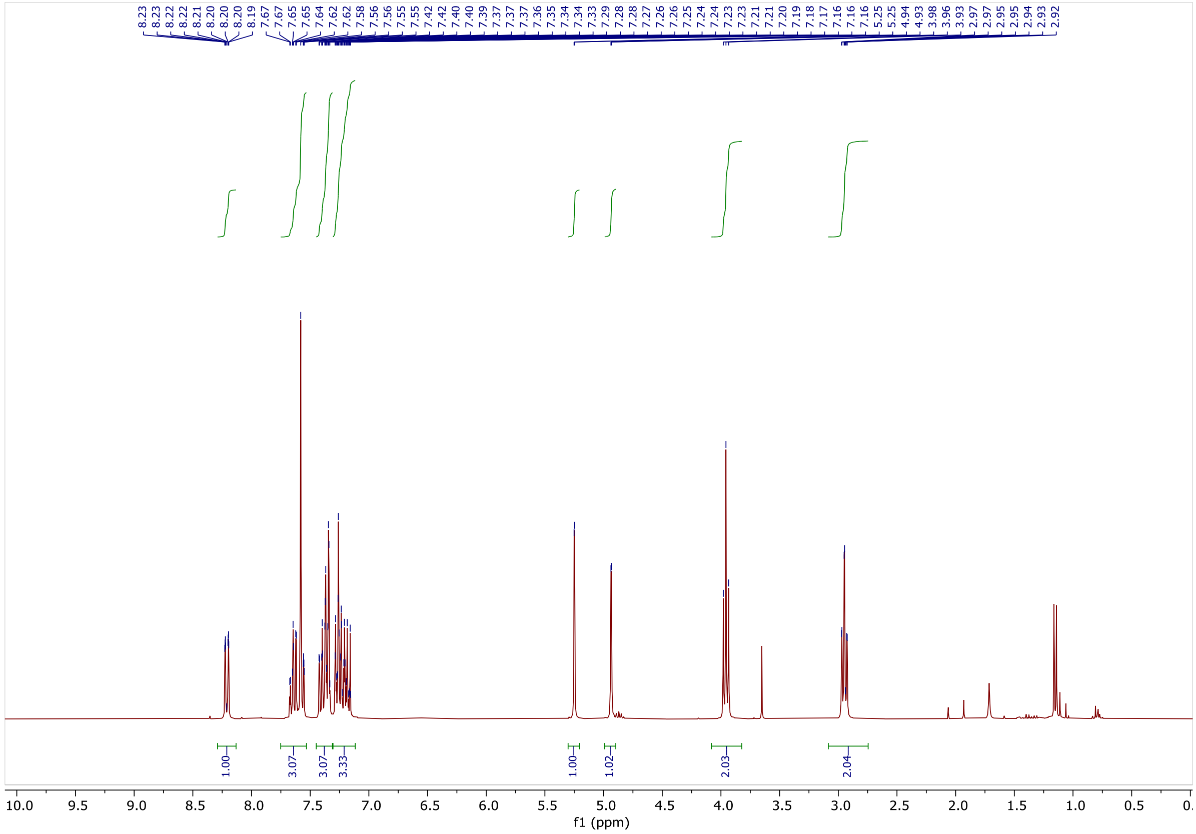

^{13}C NMR of 1b (101 MHz, $CDCl_3$)

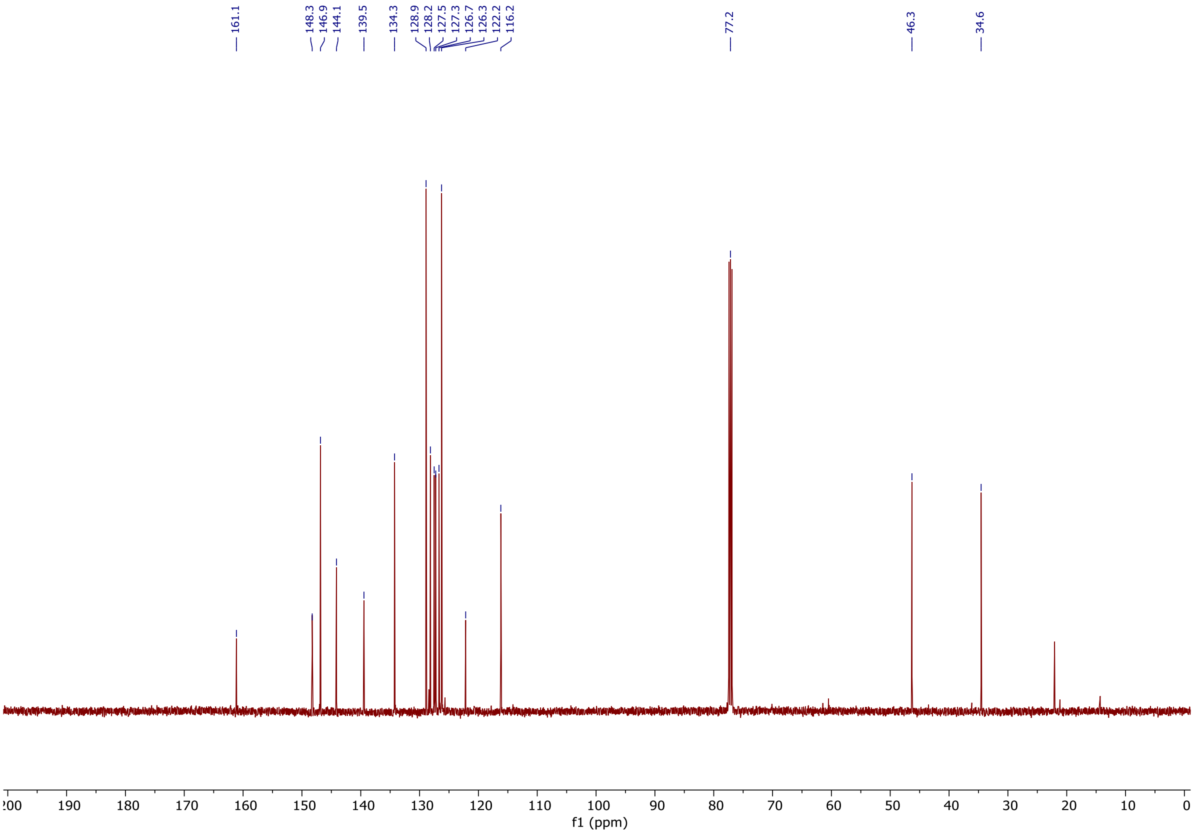

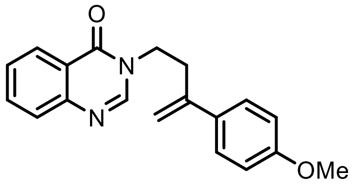

3-(3-(4-Methoxyphenyl)but-3-en-1-yl)quinazolin-4(3*H*)-one (1c)

The title compound was synthesised following General Procedure A. **1c** was obtained in 40% yield (2.16 mmol scale, 0.864 mmol, 265 mg, 40%).

^{1}H NMR (400 MHz, $CDCl_3$) δ ppm 8.34 (dd, *J* = 8.0, 1.5 Hz, 1H, ArCH), 7.77 (ddd, *J* = 8.4, 7.0, 1.5 Hz, 1H, ArCH), 7.71 (s, 1H, N=CH), 7.70 (dd, *J* = 7.7, 1.2 Hz, 1H, ArCH), 7.53 (ddd, *J* = 8.1, 7.0, 1.3 Hz, 1H, ArCH), 7.45 – 7.40 (m, 2H, *AA'*BB' system), 6.94 – 6.89 (m, 2H, AA'*BB'* system), 5.30 (d, *J* = 1.2 Hz, 1H, C=CH_2), 4.97 (d, *J* = 1.2 Hz, 1H, C=CH_2), 4.09 (t, *J* = 6.7 Hz, 2H, NCH_2), 3.83 (s, 3H, OCH_3), 3.04 (td, *J* = 6.7, 1.0 Hz, 2H, NCH_2CH_2).

^{13}C NMR (101 MHz, $CDCl_3$) δ ppm 161.1 (CON), 160.0 (ArCOMe), 148.2 (*C*=CH_2), 146.8 (N=CH), 143.3 (ArC), 134.2 (ArCH), 131.7 (ArC), 127.4 (ArCH), 127.3 (ArCH x 2), 127.2 (ArCH), 126.6 (ArCH), 122.1 (ArC), 114.5 (C=CH_2), 114.1 (ArCH x 2), 55.3 (OCH_3), 46.3 (NCH_2), 34.5 (NCH_2CH_2).

^{1}H NMR of 1c (400 MHz, $CDCl_3$)

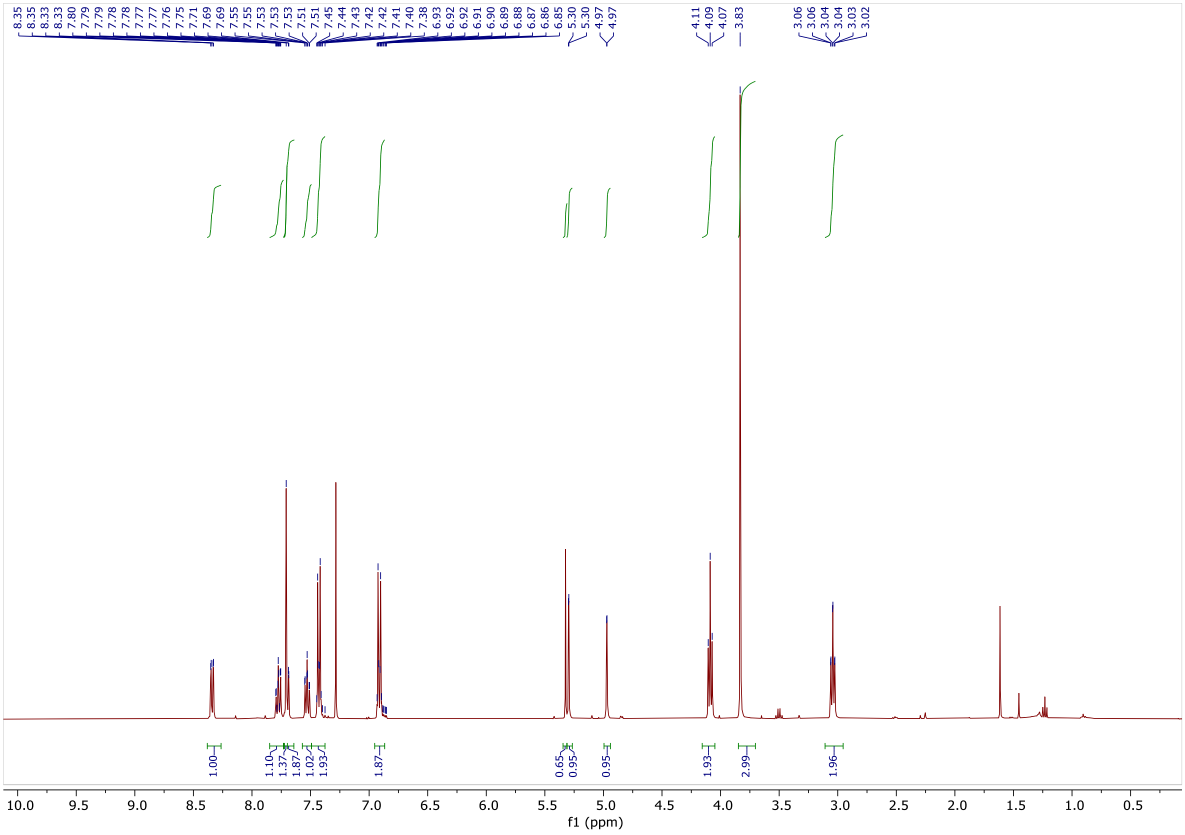

^{13}C NMR of 1c (101 MHz, CDCl$_3$)

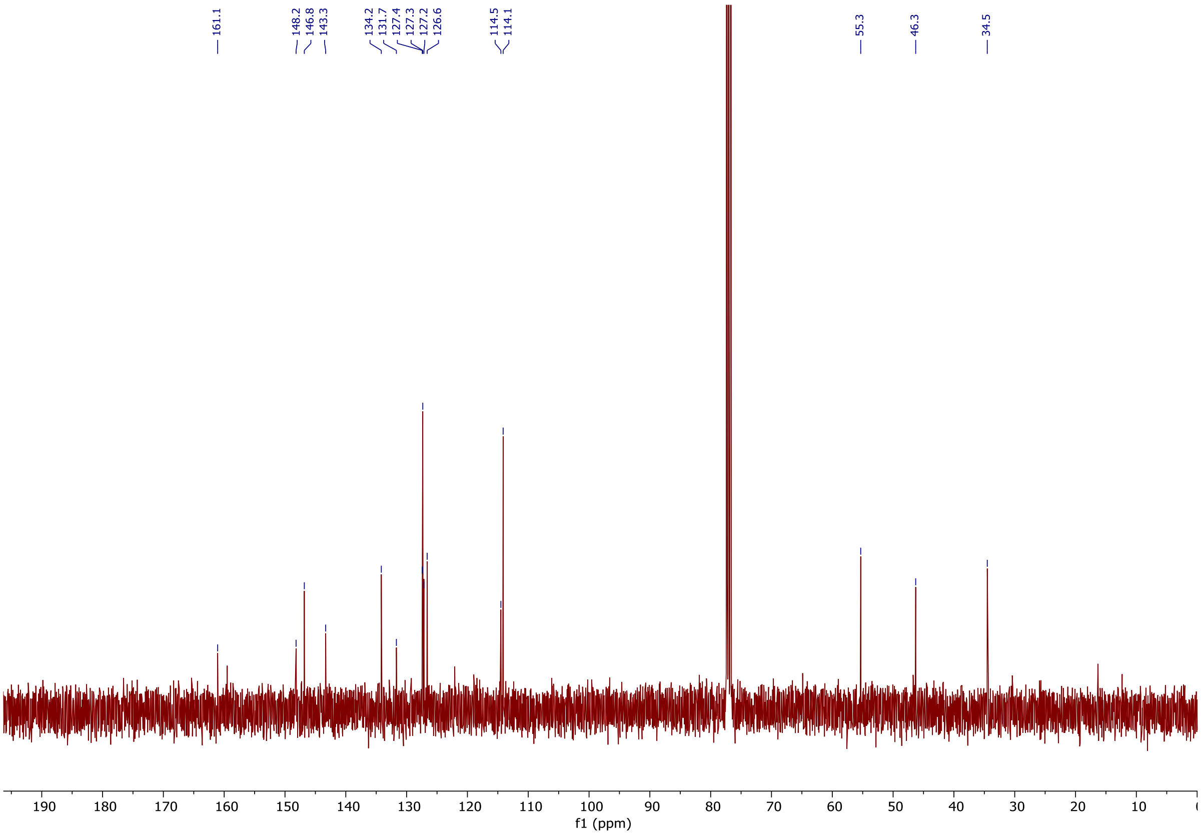

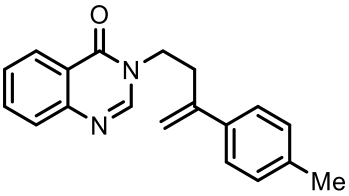

3-(3-(*p*-Tolyl)but-3-en-1-yl)quinazolin-4(3*H*)-one (1d)

The title compound was synthesised following General Procedure A. **1d** was obtained in 54% yield (2 mmol scale, 1.73 mmol, 311 mg, 54%).

^{1}H NMR (500 MHz, CDCl$_3$) δ ppm 8.31 (dd, *J* = 7.9, 1.6 Hz, 1H, ArCH), 7.74 (tt, *J* = 7.2, 1.5 Hz, 1H, ArCH), 7.69 – 7.65 (m, 2H, ArCH + N=CH), 7.50 (t, *J* = 7.5 Hz, 1H, ArCH), 7.35 (d, *J* = 7.8 Hz, 2H, ArCH x 2), 7.16 (d, *J* = 7.8 Hz, 2H, ArCH x 2), 5.31 (s, 1H, C=CH$_2$), 4.98 (s, 1H, C=CH$_2$), 4.05 (t, *J* = 6.7 Hz, 2H, NCH$_2$), 3.03 (t, *J* = 6.7 Hz, 2H, NCH$_2$*CH$_2$*), 2.33 (s, 3H, CH$_3$).

^{13}C NMR (126 MHz, CDCl$_3$) δ ppm 161.2 (CON), 148.3 (*C*=CH$_2$), 146.9 (N=CH), 143.9 (ArC), 138.0 (ArC), 136.5 (ArC), 134.2 (ArCH), 129.6 (ArCH x 2), 127.5 (ArCH), 127.2 (ArCH), 126.7 (ArCH), 126.2 (ArCH x 2), 122.2 (ArC), 115.4 (C=*C*H$_2$), 46.3 (NCH$_2$), 34.6 (NCH$_2$*C*H$_2$), 21.2 (CH$_3$).

^{1}H NMR of 1d (500 MHz, $CDCl_3$)

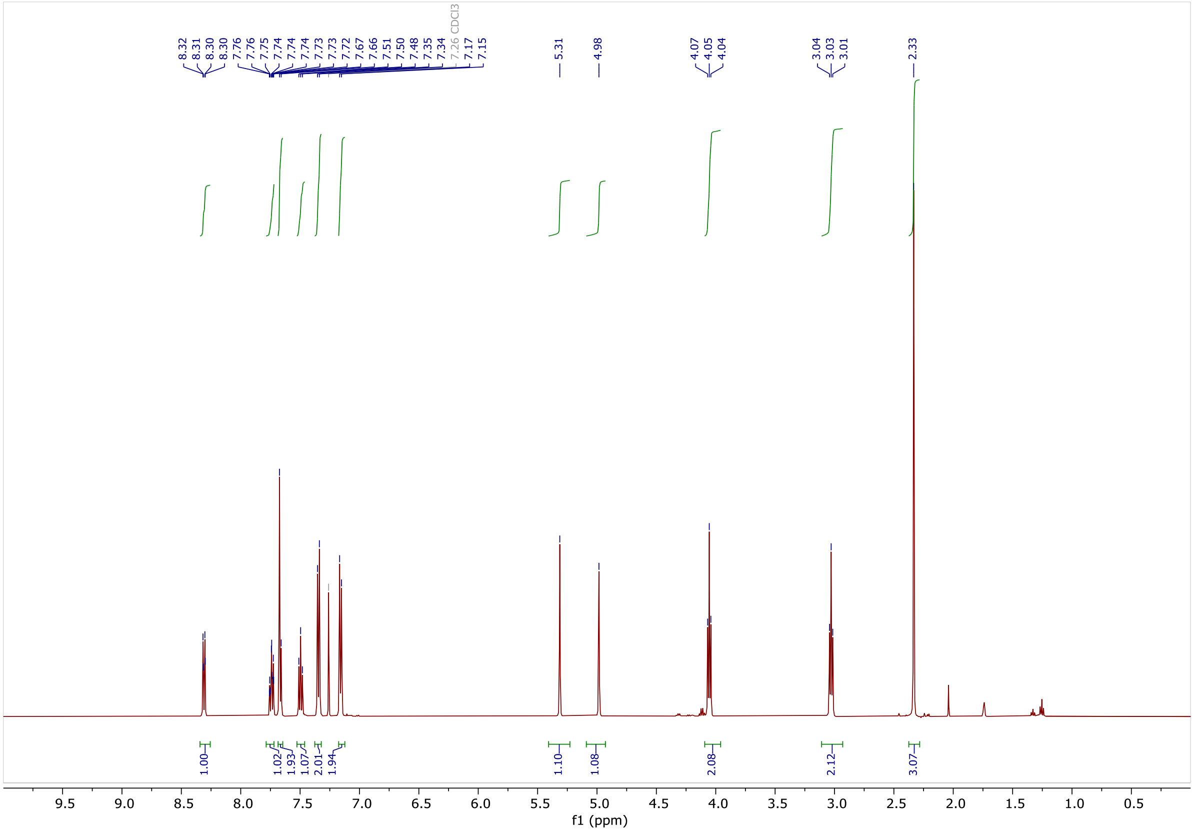

^{13}C NMR of 1d (126 MHz, $CDCl_3$)

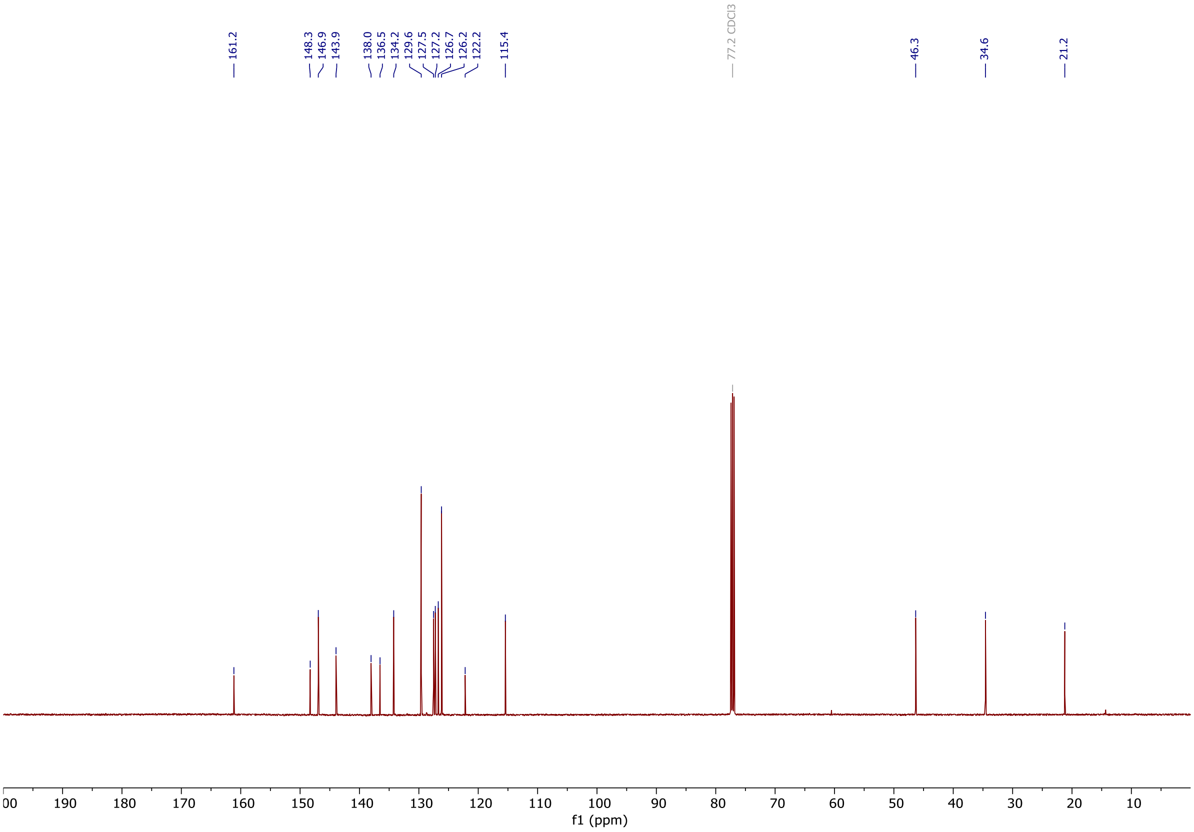

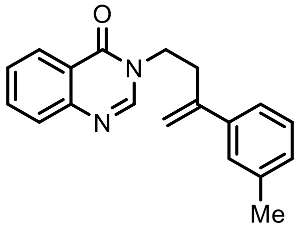

3-(3-(m-Tolyl)but-3-en-1-yl)quinazolin-4(3*H*)-one (1e)

The title compound was synthesised following General Procedure A. **1e** was obtained in 36% yield (2.85 mmol scale, 1.03 mmol, 299 mg, 36%).

^{1}H NMR (400 MHz, $CDCl_3$) 8.32 (dd, *J* = 8.0, 1.5 Hz, 1H, ArCH), 7.75 (td, *J* = 7.0, 1.5 Hz, 1H, ArCH), 7.69 (s, 1H, N=CH), 7.67 (dd, *J* = 8.2, 1.2 Hz, 1H, ArCH), 7.50 (dt, *J* = 7.0, 1.2 Hz, 1H, ArCH), 7.26 – 7.23 (m, 3H, ArCH), 7.12 – 7.06 (m, 1H, ArCH), 5.34 (d, *J* = 1.2 Hz, 1H, $C=CH_2$), 5.02 (d, *J* = 1.1 Hz, 1H, $C=CH_2$), 4.07 (t, *J* = 6.7 Hz, 2H, NCH_2), 3.04 (td, *J* = 6.7, 1.0 Hz, 2H, $NCH_2\mathit{CH_2}$), 2.34 (s, 3H, CH_3).

^{13}C NMR (101 MHz, $CDCl_3$) 161.2 (CON), 148.3 (*C*=CH_2), 146.9 (N=CH), 144.3 (ArC), 139.5 (ArC), 138.5 (ArC), 134.3 (ArCH), 128.9 (ArCH), 128.8 (ArCH), 127.5 (ArCH), 127.2 (ArCH), 127.0 (ArCH), 126.8 (ArCH), 123.4 (ArCH), 122.2 (ArC), 116.0 (C=$\mathit{CH_2}$), 46.4 (NCH_2), 34.6 ($NCH_2\mathit{CH_2}$), 21.6 (CH_3).

^{1}H NMR of 1e (400 MHz, $CDCl_3$)

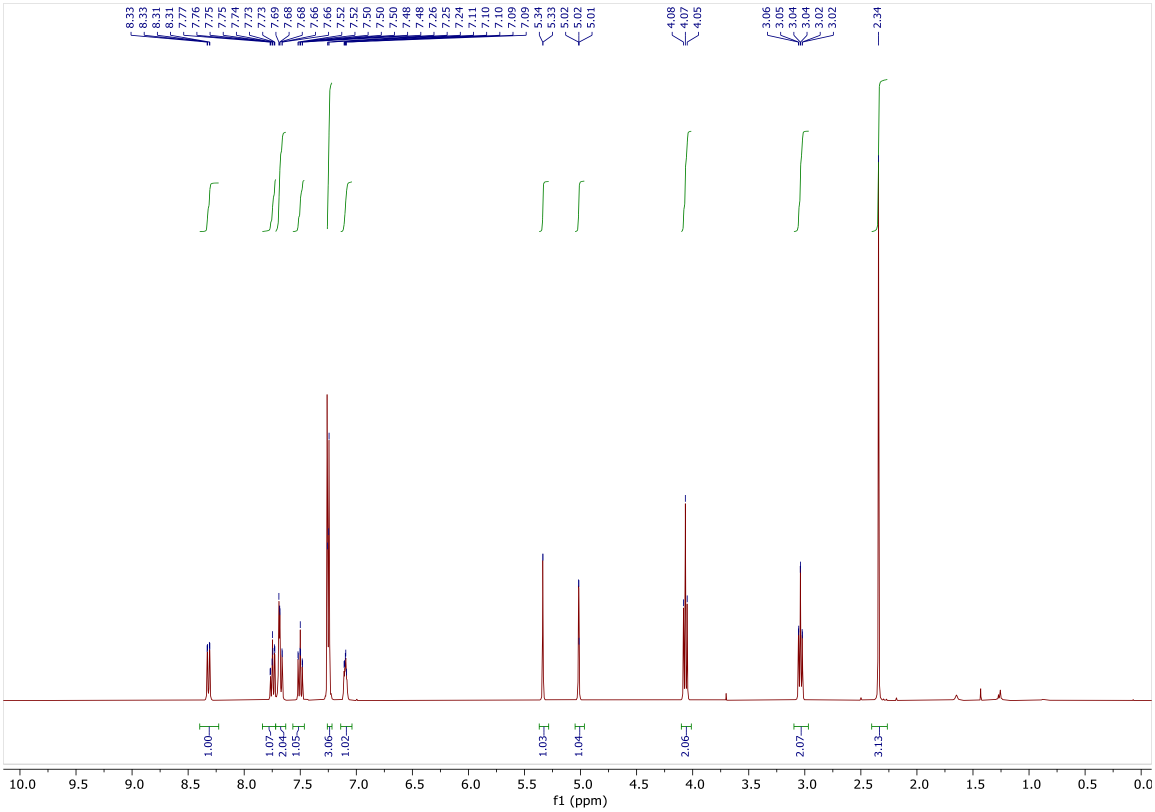

^{13}C NMR of 1e (101 MHz, CDCl$_3$)

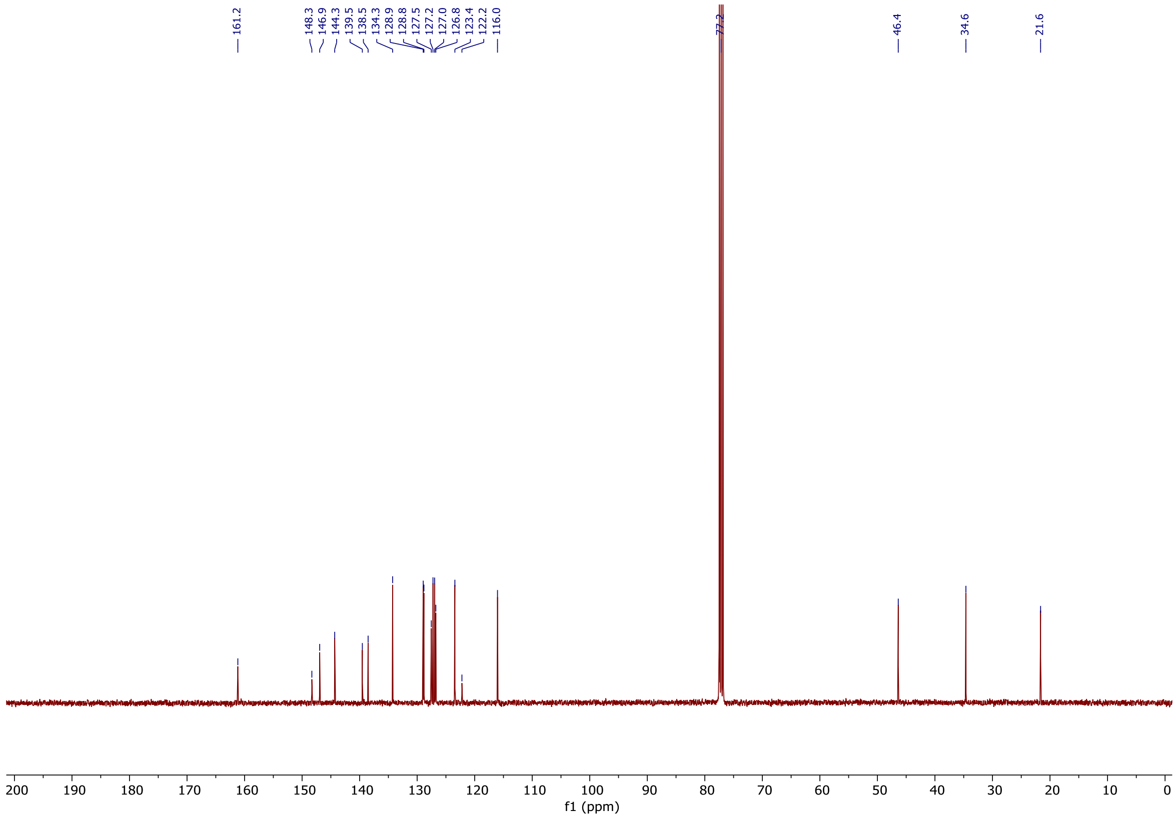

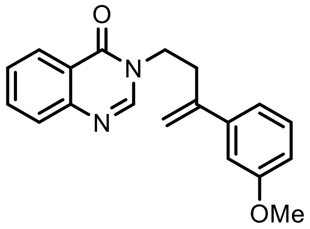

3-(3-(3-Methoxyphenyl)but-3-en-1-yl)quinazolin-4(3*H*)-one (1f)

The title compound was synthesised following General Procedure A. **1f** was obtained in 42% yield (1.70 mmol scale, 0.714 mmol, 219 mg, 42%).

^{1}H NMR (400 MHz, CDCl$_3$) δ ppm 8.31 (dd, *J* = 8.0, 1.5 Hz, 1H, ArCH), 7.75 (ddd, *J* = 8.4, 7.0, 1.5 Hz, 1H, ArCH), 7.71 (s, 1H, N=CH), 7.67 (td, *J* = 7.6, 1.0 Hz, 1H, ArCH), 7.50 (ddd, *J* = 8.2, 7.1, 1.3 Hz, 1H, ArCH), 7.29 (t, *J* = 7.9 Hz, 1H, ArCH), 7.05 (ddd, *J* = 7.7, 1.7, 1.0 Hz, 1H, ArCH), 6.98 (t, *J* = 2.1 Hz, 1H, ArCH), 6.83 (ddd, *J* = 8.2, 2.6, 0.9 Hz, 1H, ArCH), 5.36 (d, *J* = 1.2 Hz, 1H, C=CH$_2$), 5.05 (d, *J* = 1.1 Hz, 1H, C=CH$_2$), 4.07 (t, *J* = 6.7 Hz, 2H, NCH$_2$), 3.82 (s, 3H), 3.03 (t, *J* = 6.8 Hz, 2H, NCH$_2$*CH$_2$*).

^{13}C NMR (126 MHz, CDCl$_3$) δ ppm 160.9 (CON), 159.8 (ArCOMe), 148.1 (*C*=CH$_2$), 146.8 (N=CH), 143.9 (ArC), 140.8 (ArC), 134.1 (ArCH), 129.8 (ArCH), 127.4 (ArCH), 127.1 (ArCH), 126.5 (ArCH),

122.0 (ArC), 118.6 (ArCH), 116.1 (C=CH_2), 113.2 (ArCH), 112.0 (ArCH), 55.2 (NCH_2), 46.2 (NCH_2CH_2), 34.5 (OCH_3).

^{1}H NMR of 1f (400 MHz, $CDCl_3$)

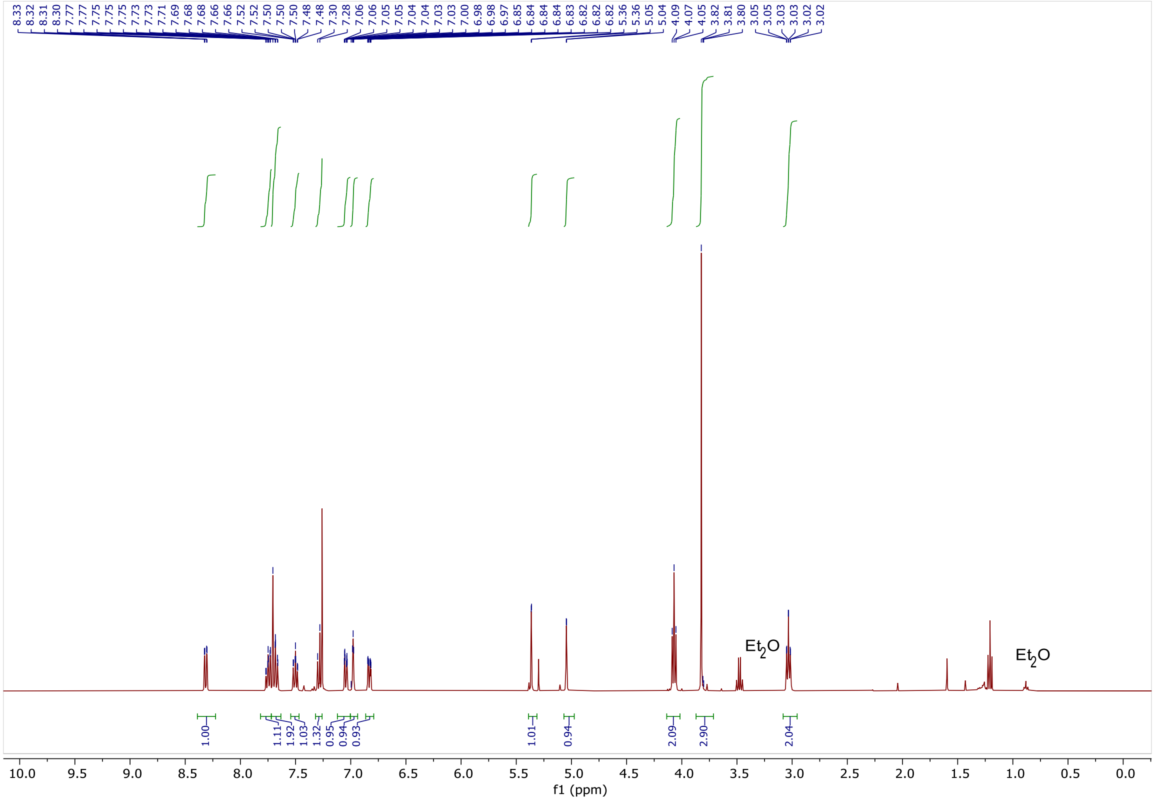

^{13}C NMR of 1f (126 MHz, $CDCl_3$)

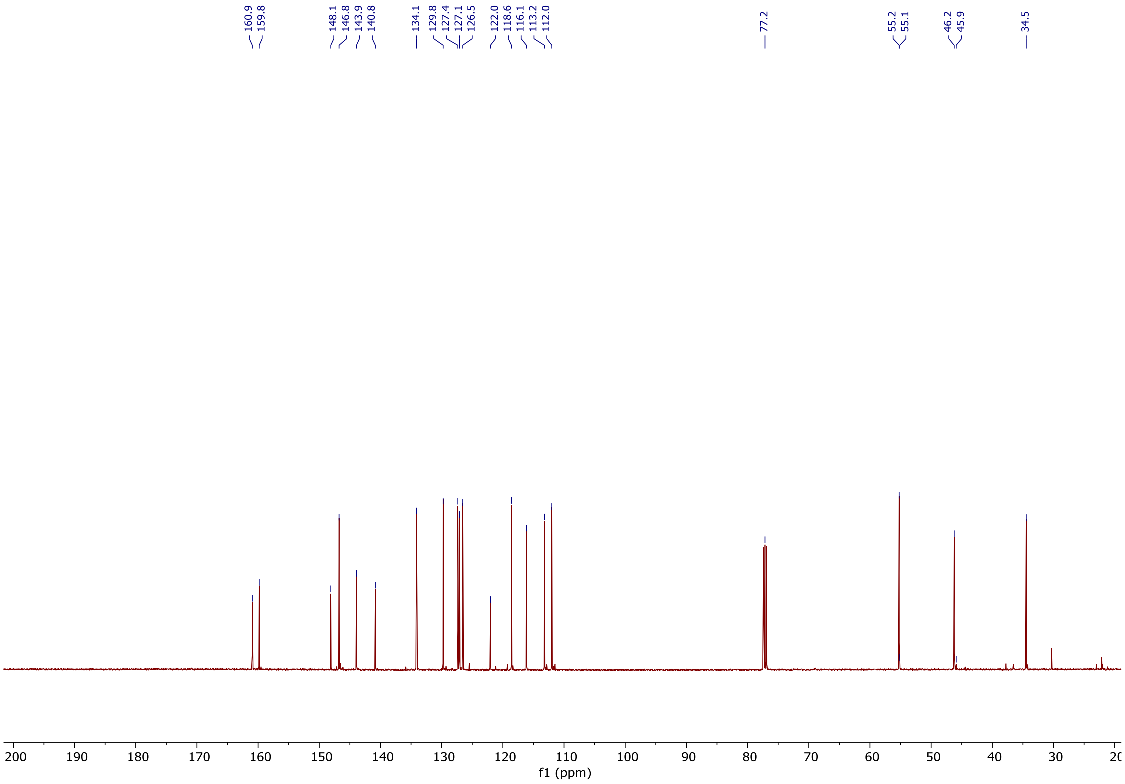

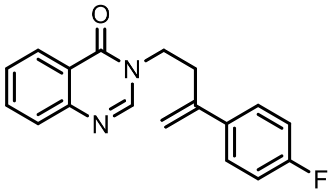

3-(3-(4-Fluorophenyl)but-3-en-1-yl)quinazolin-4(3*H*)-one (1g)

The title compound was synthesised following General Procedure A. **1g** was obtained in 37% yield (1.70 mmol scale, 0.629 mmol, 184 mg, 37%).

^{1}H NMR (400 MHz, $CDCl_3$) δ ppm 8.31 (dd, J = 8.0, 0.9 Hz, 1H, ArCH), 7.75 (ddd, J = 8.4, 7.0, 1.6 Hz, 1H, ArCH), 7.71 (s, 1H, N=CH), 7.68 (d, J = 7.7 Hz, 1H, ArCH), 7.51 (ddd, J = 8.2, 7.0, 1.2 Hz, 1H. ArCH), 7.46 – 7.40 (m, 2H, *AA'*BB' system), 7.08 – 6.99 (m, 2H, AA'*BB'* system), 5.31 (d, J = 0.9 Hz, 1H, C=CH_2), 5.05 (d, J = 1.1 Hz, 1H, C=CH_2), 4.06 (t, J = 6.9 Hz, 2H, NCH_2), 3.02 (td, J = 6.9, 1.0 Hz, 2H, NCH_2*CH_2*).

^{13}C NMR (101 MHz, $CDCl_3$) δ ppm 162.7 (d, $^1J_{C\text{-}F}$ = 247.5 Hz, ArC), 161.2 (CON), 148.3 (*C*=CH_2), 146.7 (N=CH), 143.2 (ArC), 135.6 (d, $^4J_{C\text{-}F}$ = 3.3 Hz, ArC), 134.4 (ArCH), 127.9 (d, $^3J_{C\text{-}F}$ = 7.9 Hz, ArCH x 2), 127.6 (ArCH), 127.4 (ArCH), 126.7 (ArCH), 122.2 (ArC), 116.0 (C=*CH_2*), 115.8 (d, $^2J_{C\text{-}F}$ = 21.5 Hz, ArCH x 2), 46.4 (NCH_2), 34.7 (NCH_2*C*H_2).

^{19}F NMR (376 MHz, $CDCl_3$) δ ppm -114.1 (tt, J = 8.5, 5.4 Hz, 1F).

^{1}H NMR of 1g (400 MHz, $CDCl_3$)

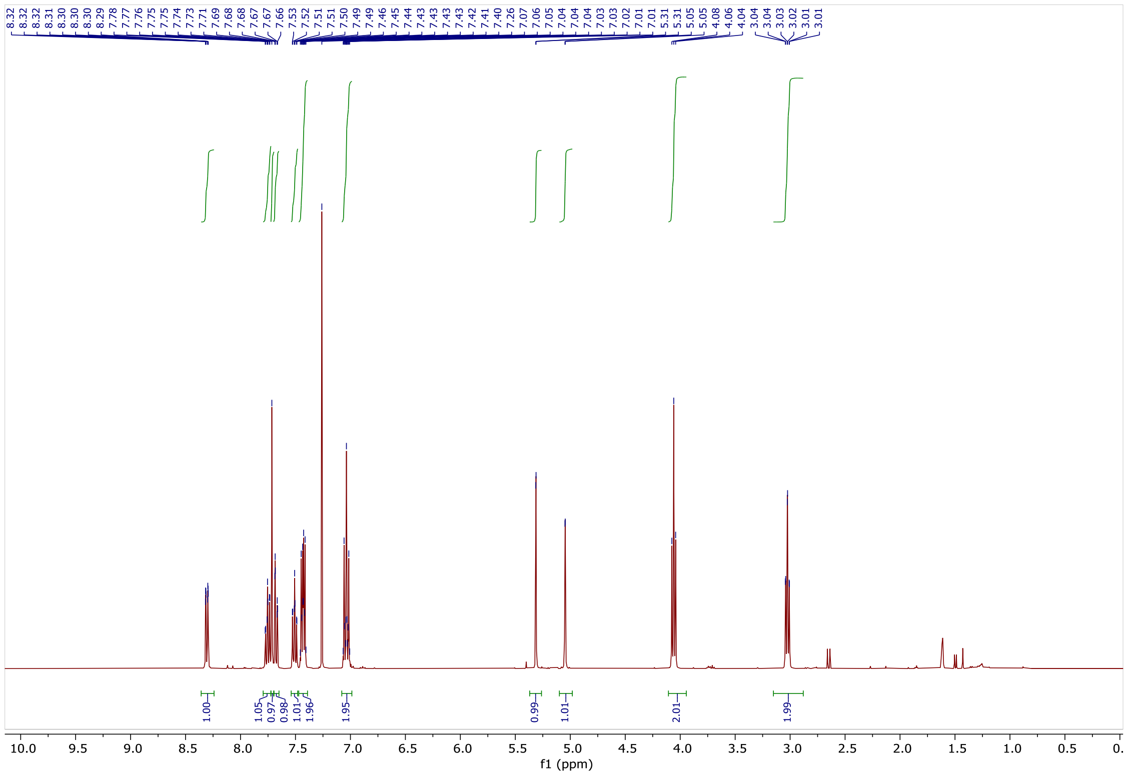

^{13}C NMR of 1g (101 MHz, $CDCl_3$)

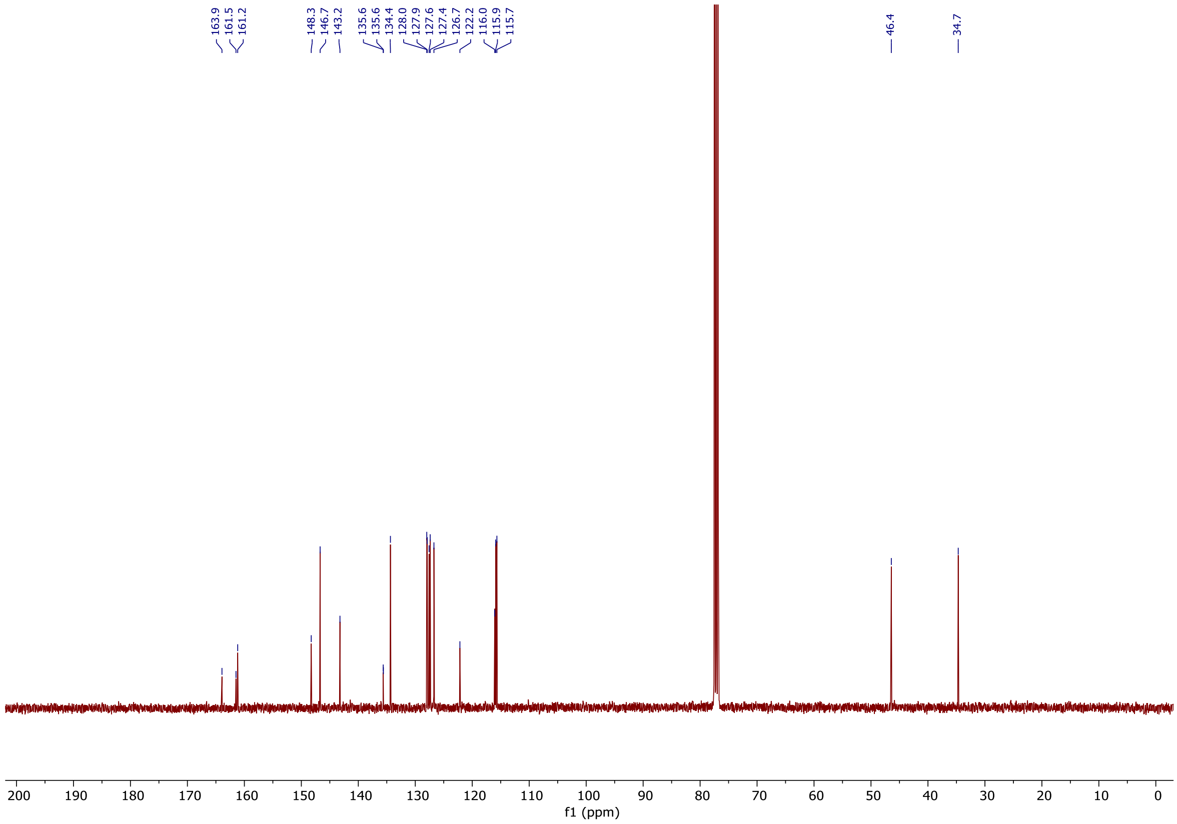

^{19}F NMR of 1g (376 MHz, $CDCl_3$)

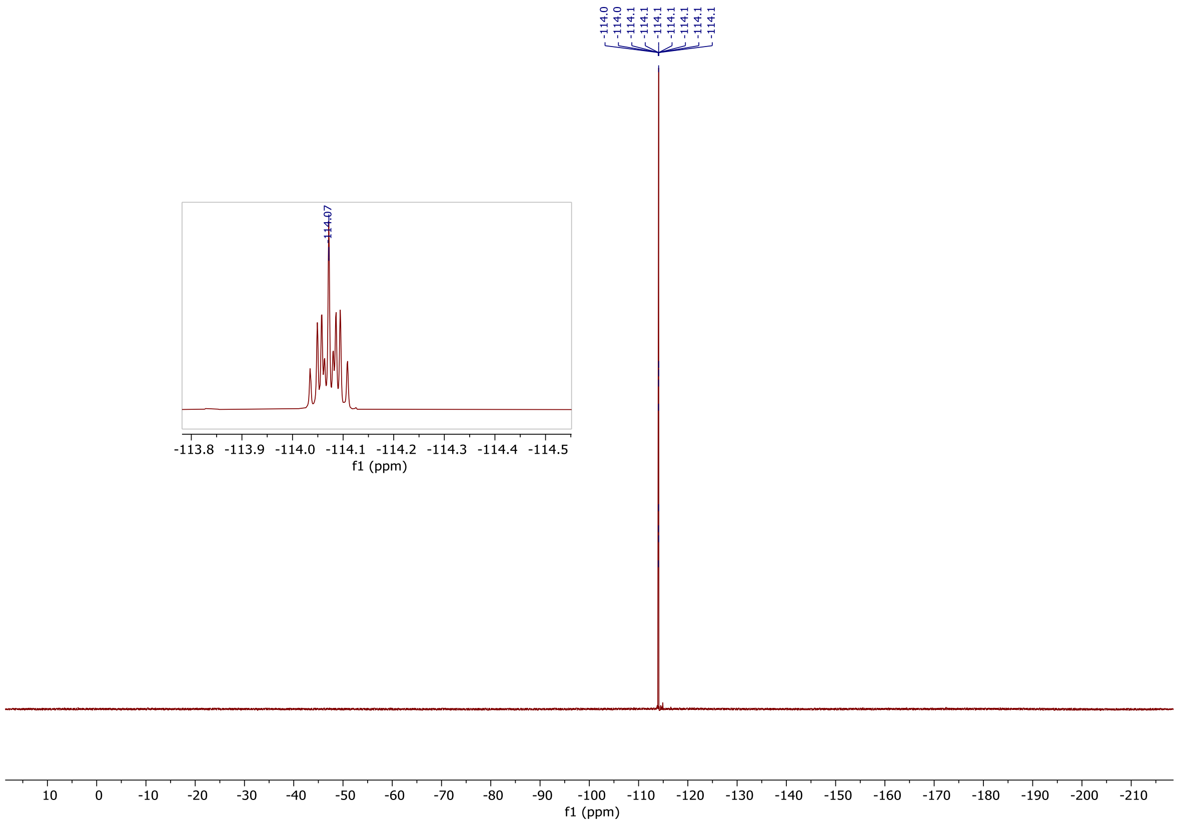

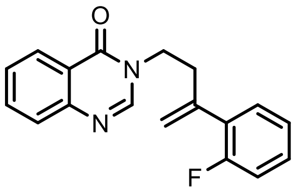

3-(3-(2-Fluorophenyl)but-3-en-1-yl)quinazolin-4(3*H*)-one (1h)

The title compound was synthesised following General Procedure A. **1h** was obtained in 56% yield (1.98 mmol scale, 327 mg, 1.11 mmol, 56%).

^{1}H NMR (400 MHz, $CDCl_3$) δ ppm 8.30 (dd, *J* = 8.0, 1.5 Hz, 1H, ArCH), 7.79 – 7.72 (m, 2H, N=CH + ArC*H*), 7.68 (dd, *J* = 8.2, 1.2 Hz, 1H, ArC*H*), 7.54 – 7.45 (m, 1H, ArC*H*), 7.35 – 7.22 (m, 2H, ArC*H*), 7.17 – 7.02 (m, 2H, ArC*H*), 5.29 (s, 1H, C=CH_2), 5.22 (d, *J* = 1.4 Hz, 1H, C=CH_2), 4.04 (t, *J* = 6.9 Hz, 2H, NCH_2), 3.05 (t, 6.9 Hz, 2H, NCH_2CH_2).

^{13}C NMR (101 MHz, $CDCl_3$) δ ppm 161.2 (CON), 160.0 (d, $^1J_{C\text{-}F}$ = 247.9 Hz, ArC), 148.3 (*C*=CH_2), 146.8 (N=CH), 140.6 (ArC), 134.3 (ArCH), 130.2 (d, $^3J_{C\text{-}F}$ = 4.0 Hz, ArCH), 129.7 (d, $^3J_{C\text{-}F}$ = 8.4 Hz, ArCH), 128.2 (d, $^2J_{C\text{-}F}$ = 13.6 Hz, ArC), 127.6 (ArCH), 127.3 (ArCH), 126.8 (ArCH), 124.6 (d, $^4J_{C\text{-}F}$ = 3.6 Hz, ArCH), 122.2 (ArC), 119.8 (d, $^4J_{C\text{-}F}$ = 2.5 Hz, C=CH_2), 116.3 (d, $^2J_{C\text{-}F}$ = 22.9 Hz, ArCH), 46.3 (NCH_2), 35.5 (d, ^4J = 3.9 Hz, NCH_2CH_2).

^{19}F NMR (376 MHz, $CDCl_3$) δ ppm -114.3 (dt, *J* = 12.3, 6.6 Hz).

^{1}H NMR of 1h (400 MHz, $CDCl_3$)

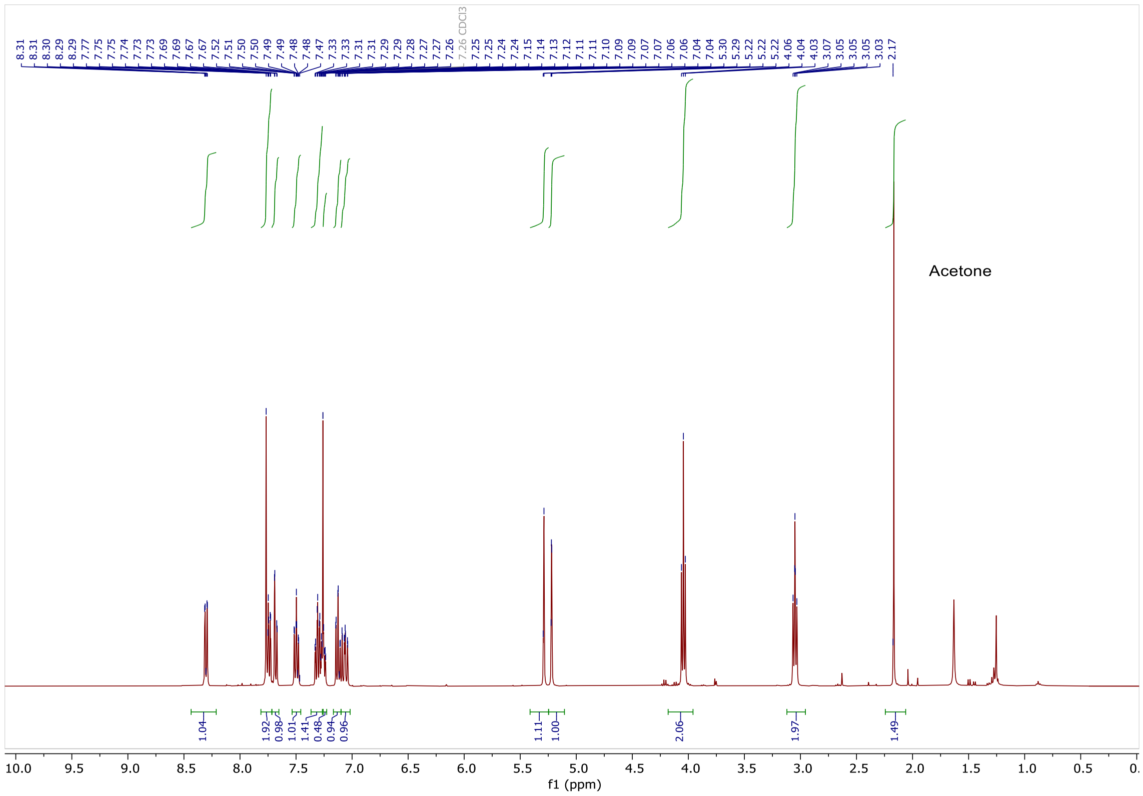

^{13}C NMR of 1h (101 MHz, $CDCl_3$)

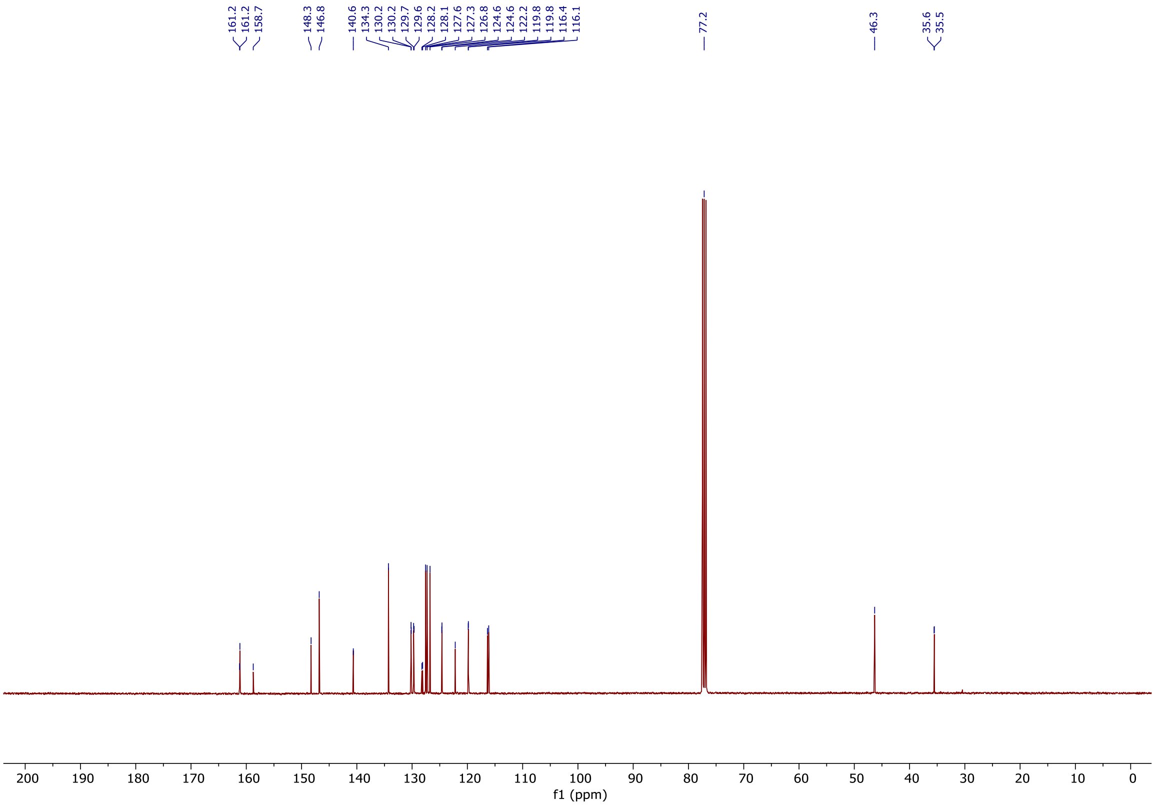

^{19}F NMR of 1h (376 MHz, $CDCl_3$)

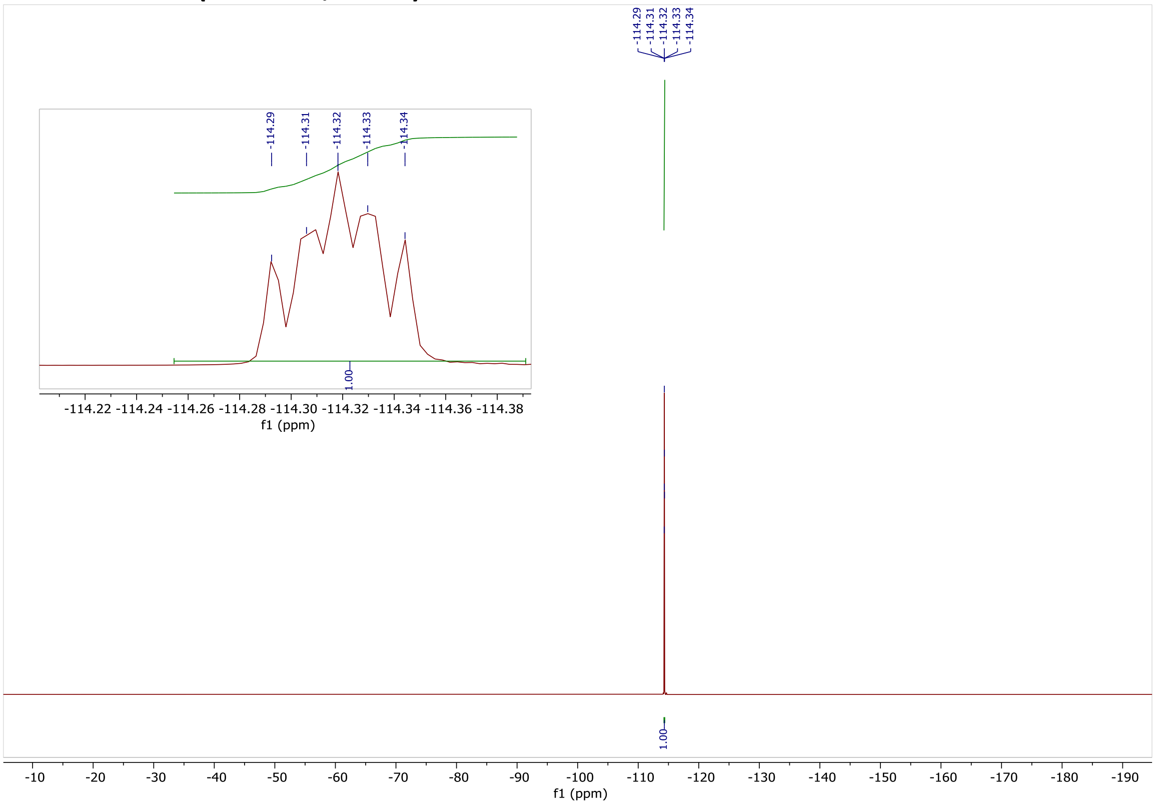

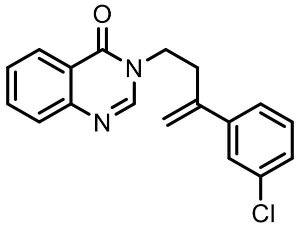

3-(3-(3-Chlorophenyl)but-3-en-1-yl)quinazolin-4(3*H*)-one (1i)

The title compound was synthesised following General Procedure A. **1i** was obtained in 53% yield (2.10 mmol scale, 346 mg, 1.11 mmol, 53%).

^{1}H NMR (400 MHz, $CDCl_3$) δ ppm 8.44 (d, *J* = 2.3 Hz, 1H, ArCH), 7.82 (dd, *J* = 8.7, 2.3 Hz, 1H, ArCH), 7.66 (s, 1H, N=CH), 7.54 (d, *J* = 8.6 Hz, 1H, ArCH), 7.47 – 7.40 (m, 2H, ArCH), 7.41 – 7.33 (m, 2H, ArCH), 7.32 – 7.28 (m, 1H, ArCH), 5.35 (d, *J* = 1.2 Hz, 1H, C=CH_2), 5.03 (d, *J* = 1.2 Hz, 1H, C=CH_2), 4.06 (t, *J* = 6.7 Hz, 2H, NCH_2), 3.05 (td, *J* = 6.7, 1.1 Hz, 2H, NCH_2CH_2).

^{13}C NMR (126 MHz, $CDCl_3$) δ ppm 160.9 (CON), 148.0 (*C*=CH_2), 146.5 (N=CH), 142.9 (ArC), 141.3 (ArC), 134.6 (ArC), 134.1 (ArCH), 130.0 (ArCH), 128.0 (ArCH), 127.4 (ArCH), 127.2 (ArCH), 126.5 (ArCH), 126.2 (ArCH), 124.3 (ArCH), 121.9 (ArC), 117.0 (C=*CH_2*), 46.1 (NCH_2), 34.2 (NCH_2CH_2).

^{1}H NMR of 1i (400 MHz, $CDCl_3$

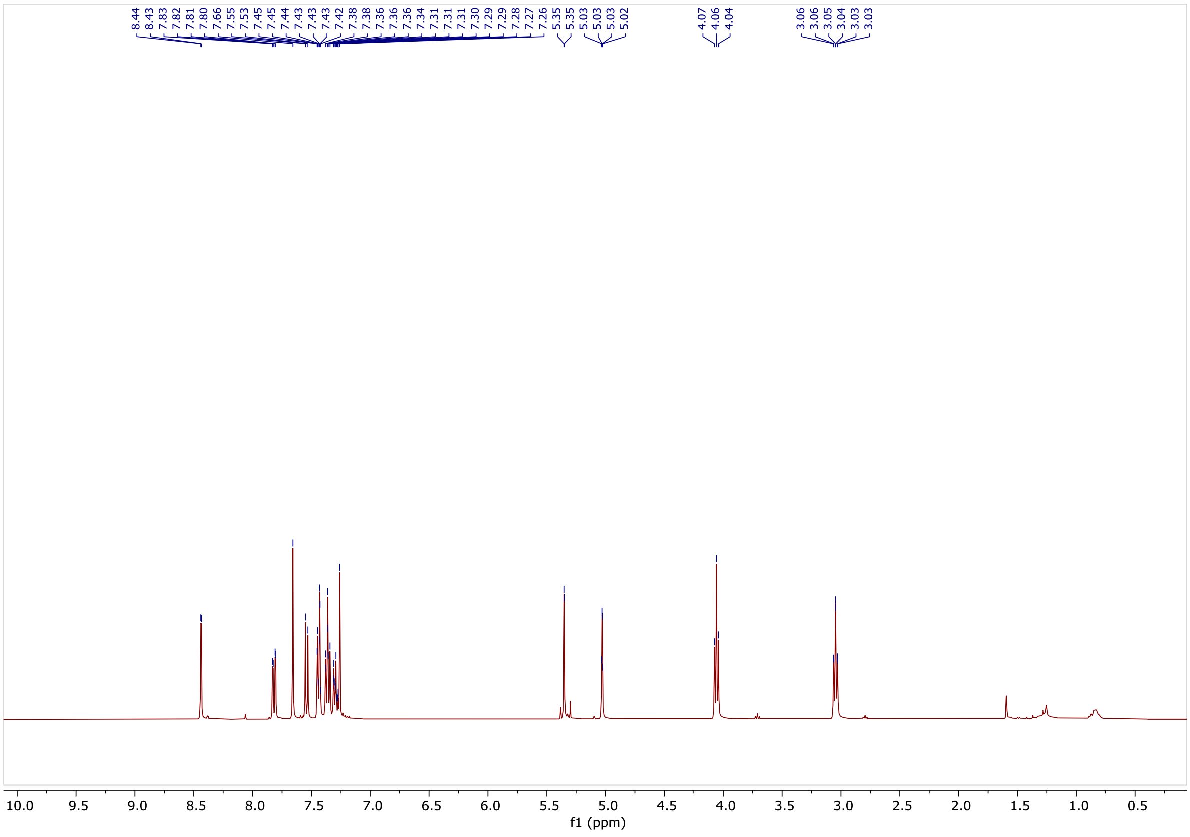

^{13}C NMR of 1i (126 MHz, CDCl$_3$)

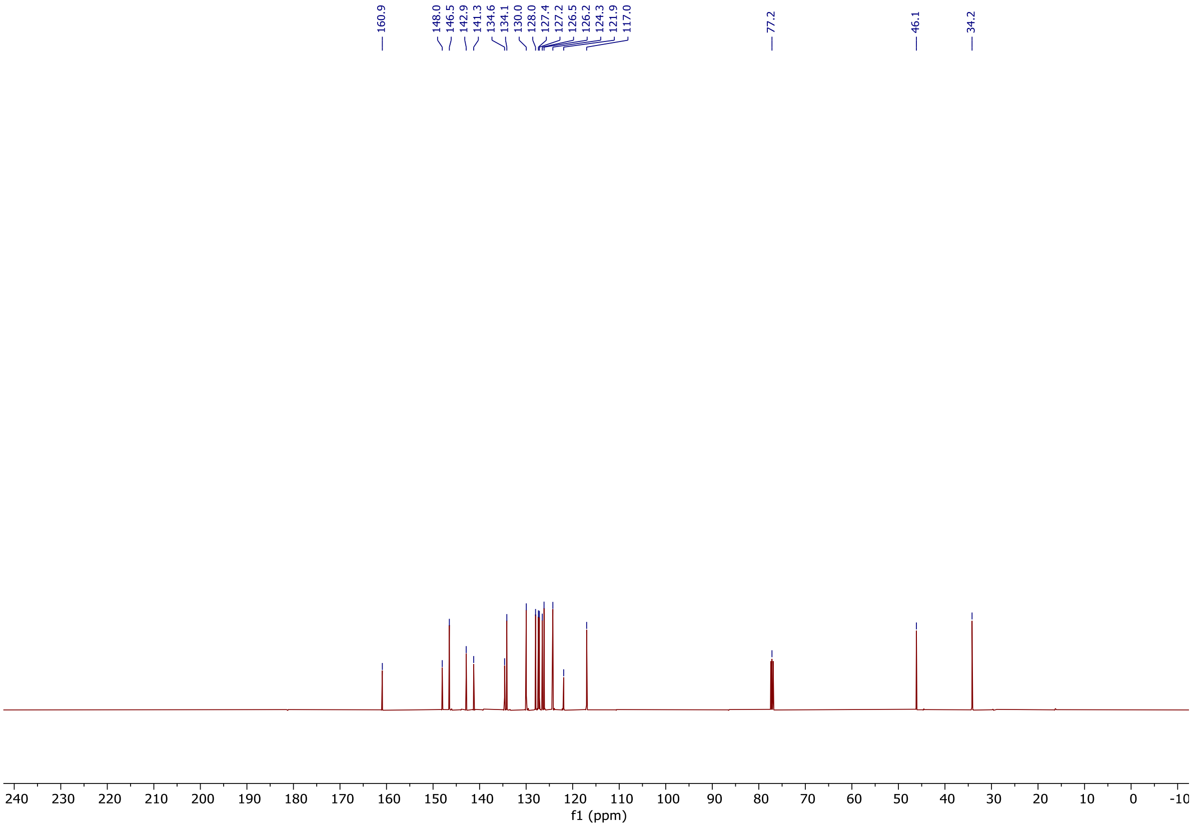

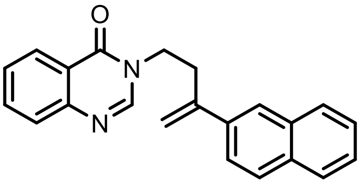

3-(3-(Naphthalen-2-yl)but-3-en-1-yl)quinazolin-4(3*H*)-one (1j)

The title compound was synthesised following General Procedure A. **1j** was obtained in 33% yield after recrystallization (1.25 mmol scale, 135 mg, 0.41 mmol, 33%).

^{1}H NMR (400 MHz, CDCl$_3$) δ ppm 8.32 (dd, *J* = 8.0, 1.5 Hz, 1H, ArCH), 7.90 (d, *J* = 1.8 Hz, 1H, ArCH), 7.87 – 7.79 (m, 3H, ArCH), 7.73 (dt, *J* = 7.2, 1.6 Hz, 1H, ArCH), 7.70 (s, 1H, N=CH), 7.65 (d, *J* = 7.6 Hz, 1H, ArCH), 7.60 (dd, *J* = 8.6, 1.8 Hz, 1H, ArCH), 7.52 – 7.44 (m, 3H, ArCH), 5.51 (s, 1H, C=CH$_2$), 5.14 (s, 1H, C=CH$_2$), 4.12 (t, *J* = 6.7 Hz, 2H, NCH$_2$), 3.17 (t, *J* = 6.7 Hz, 2H, NCH$_2$*CH$_2$*).

^{13}C NMR (101 MHz, CDCl$_3$) δ 161.2 (CON), 148.3 (*C*=CH$_2$), 146.9 (N=CH), 144.0 (ArC), 136.7 (ArC), 134.3 (ArCH), 133.5 (ArC), 133.1 (ArC), 128.7 (ArCH), 128.4 (ArCH), 127.7 (ArCH), 127.5 (ArCH), 127.3 (ArCH), 126.7 (ArCH), 126.6 (ArCH), 126.4 (ArCH), 125.1 (ArCH), 124.4 (ArCH), 122.2 (ArC), 116.7 (C=*CH$_2$*), 46.4 (NCH$_2$), 34.6 (NCH$_2$*CH$_2$*).

^{1}H NMR of 1j (400 MHz, $CDCl_3$)

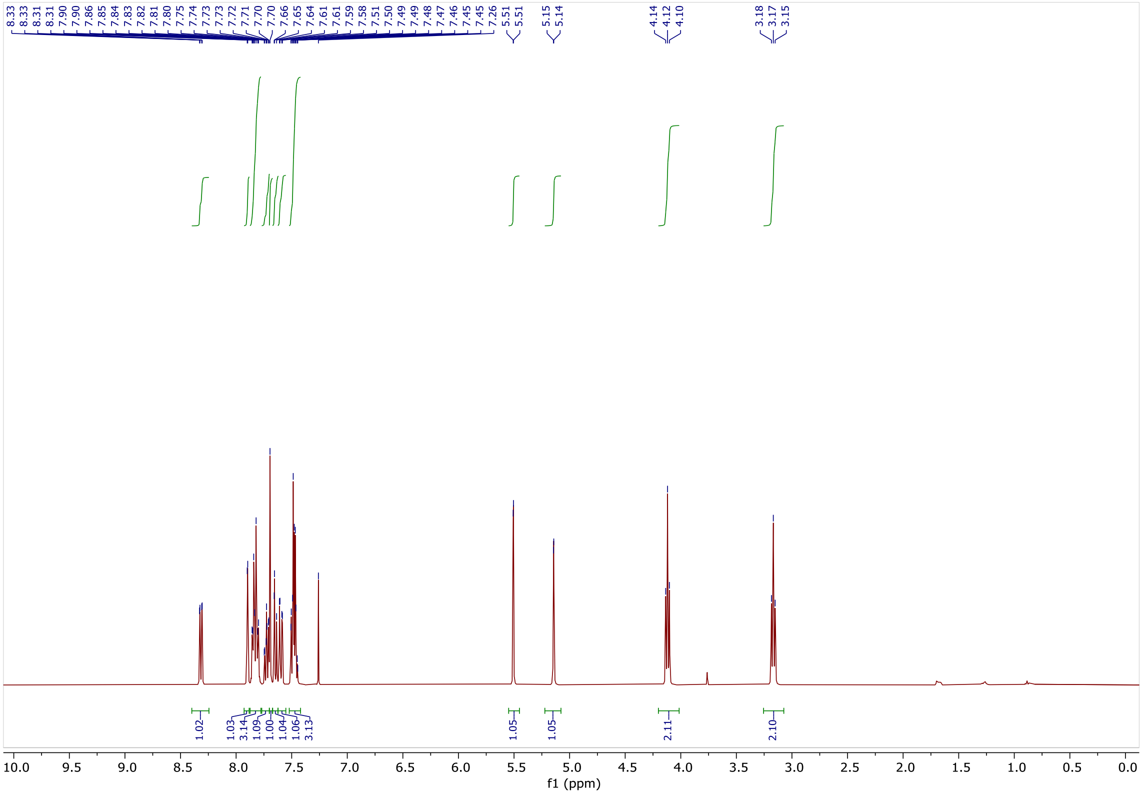

^{13}C NMR of 1j (101 MHz, $CDCl_3$)

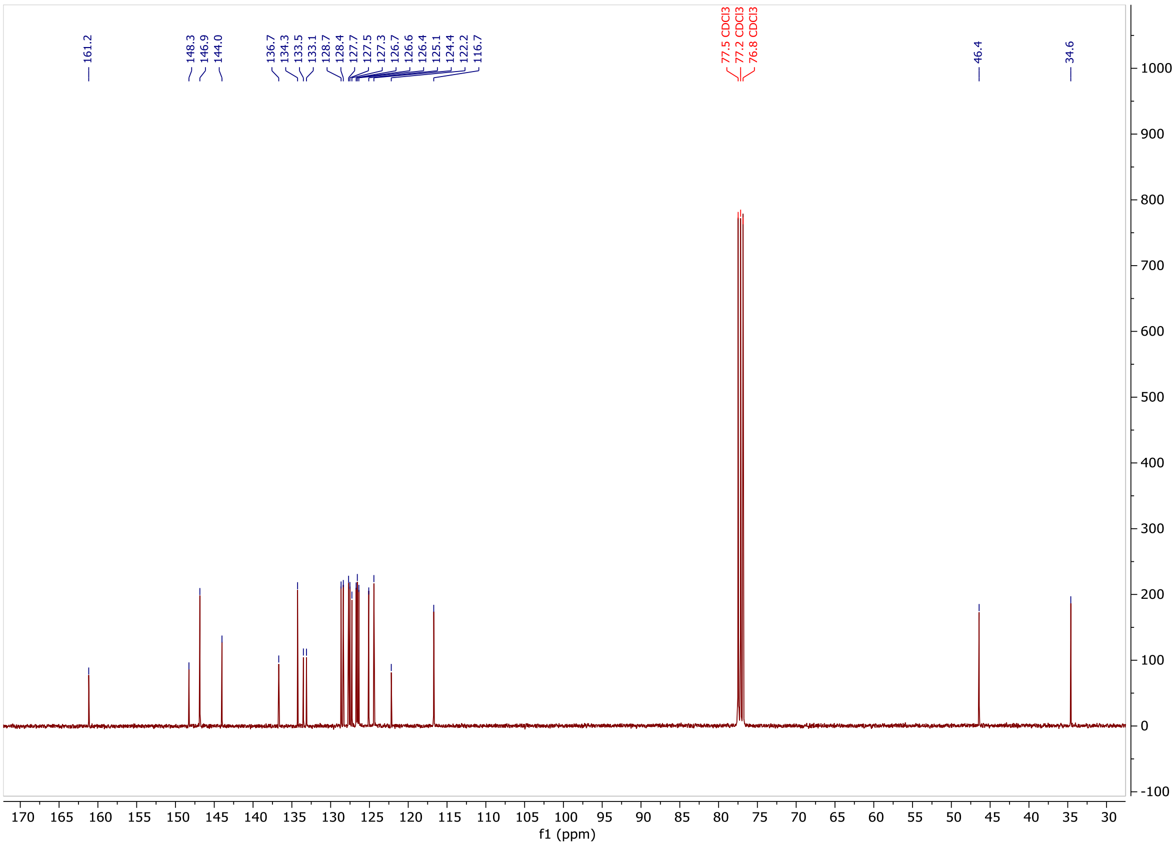

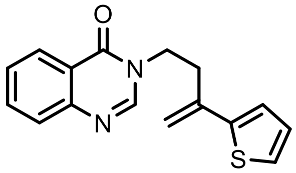

3-(3-(Thiophen-2-yl)but-3-en-1-yl)quinazolin-4()-one (1k)

The title compound was synthesised following General Procedure A. **1k** was obtained in 48% yield (1 mmol scale, 135 mg, 0.478 mmol, 48%).

^{1}H NMR (400 MHz, $CDCl_3$) δ ppm 8.32 (dd, *J* = 8.0, 1.6 Hz, 1H, ArCH), 7.81 (s, 1H, N=CH), 7.75 (ddd, *J* = 8.4, 7.0, 1.6 Hz, 1H, ArCH), 7.68 (dd, *J* = 8.3, 1.2 Hz, 1H), 7.52 (t, *J* = 7.1 Hz, 1H, ArCH), 7.23 – 7.14 (m, 2H, ArCH), 7.00 (dd, *J* = 5.1, 3.6 Hz, 1H, ArCH), 5.43 (s, 1H, C=C*H2*), 4.91 (s, 1H, C=C*H2*), 4.18 (t, *J* = 6.9 Hz, 2H, NCH_2), 2.98 (t, *J* = 6.9 Hz, 2H, , NCH_2CH_2).

^{13}C NMR (101 MHz, $CDCl_3$) δ 161.2 (CON), 148.3 (*C*=CH_2), 146.9 (N=CH), 143.6 (ArCH), 137.6 (ArC), 134.3 (ArCH), 127.9 (ArCH), 127.6 (ArCH), 127.3 (ArCH), 126.7 (ArCH), 125.2 (ArCH), 124.3 (ArC), 122.2 (ArC), 114.5 (C=*CH2*), 46.6 (NCH_2), 35.2 (NCH_2CH_2).

^{1}H NMR of 1k (400 MHz, $CDCl_3$)

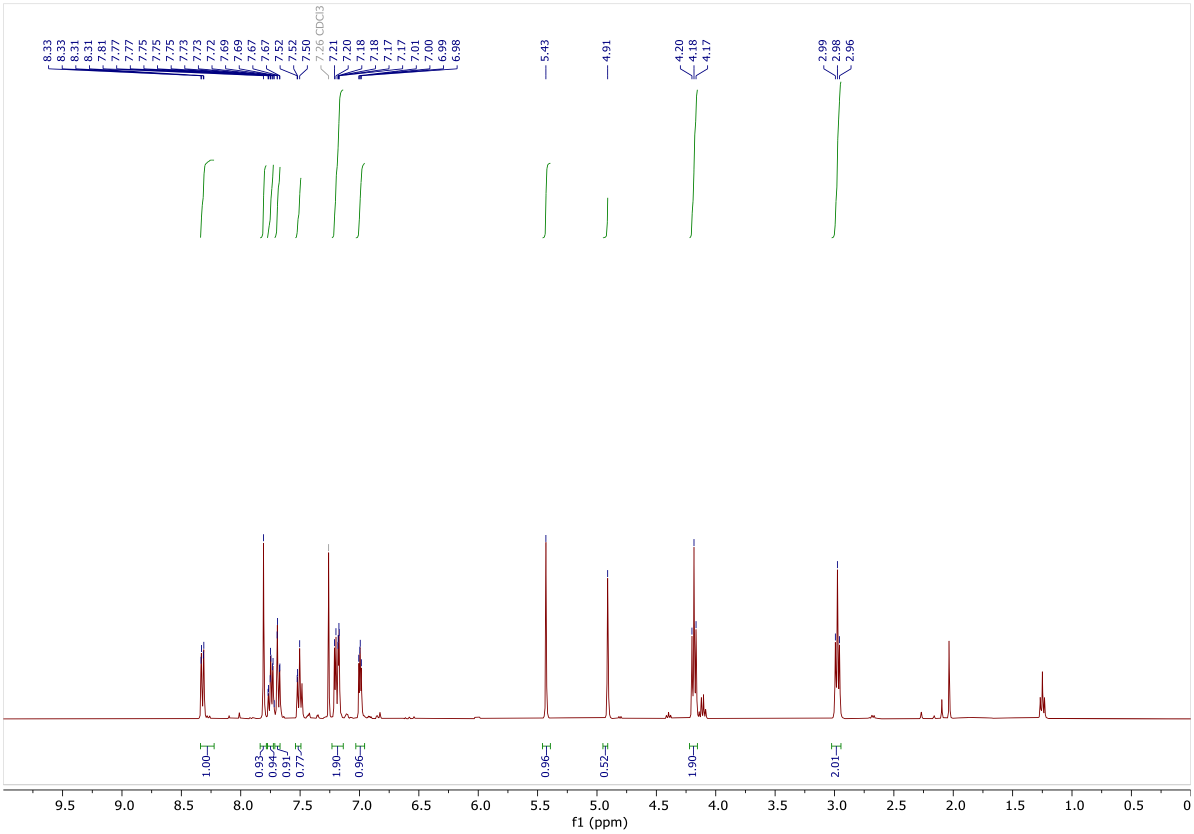

^{13}C NMR of 1k (101 MHz, CDCl$_3$)

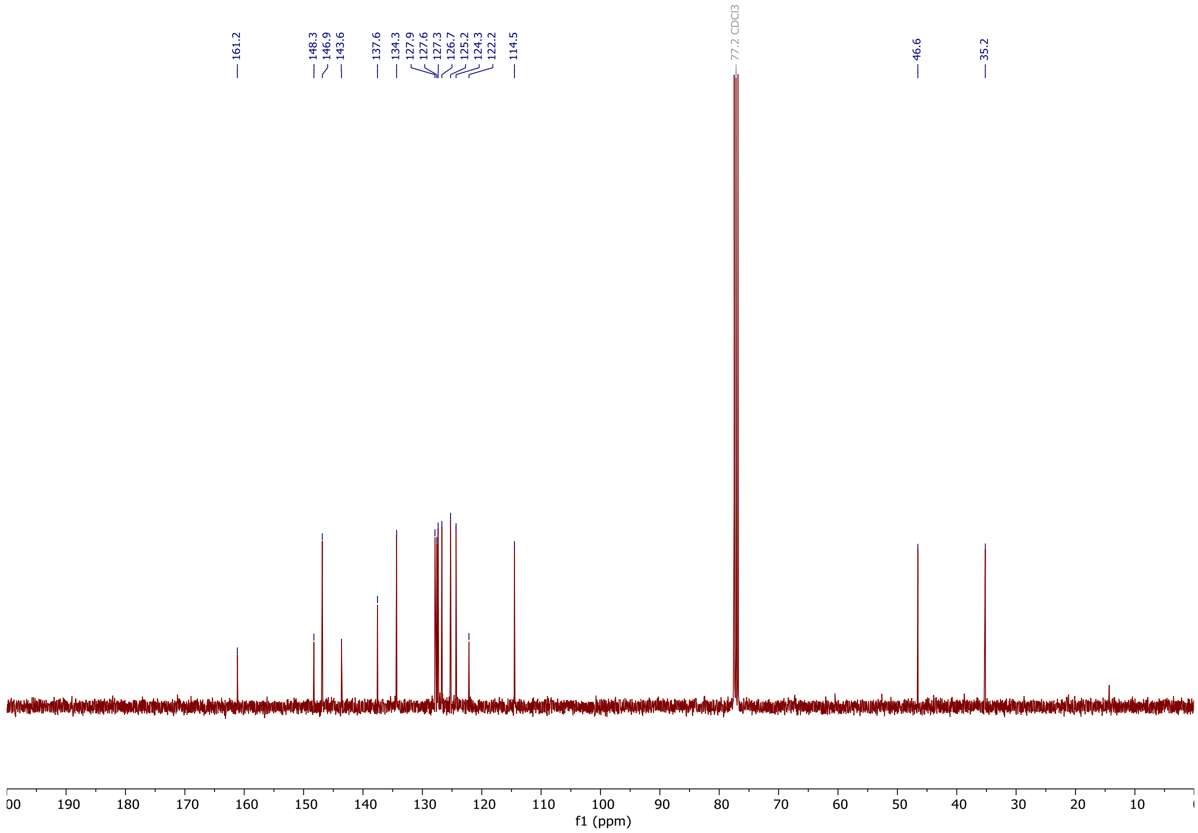

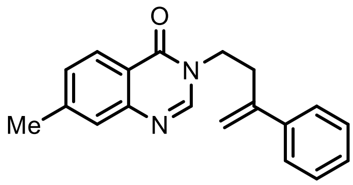

7-Methyl-3-(3-phenylbut-3-en-1-yl)quinazolin-4(3*H*)-one (1l)

The title compound was synthesised following General Procedure B. **1l** was obtained in 73% yield (1.63 mmol scale, 1.19 mmol, 345 mg, 73%).

^{1}H NMR (400 MHz, CDCl$_3$) δ ppm 8.19 (d, *J* = 8.1 Hz, 1H, ArCH), 7.66 (s, 1H, N=CH), 7.49 – 7.42 (m, 3H, ArCH), 7.40 – 7.27 (m, 4H, ArCH), 5.35 (d, *J* = 1.2 Hz, 1H, C=CH$_2$), 5.03 (d, *J* = 1.2 Hz, 1H, C=CH$_2$), 4.04 (t, *J* = 6.8 Hz, 2H, NCH$_2$), 3.04 (td, *J* = 6.8, 1.1 Hz, 2H, NCH$_2$*CH$_2$*), 2.50 (s, 3H, CH$_3$).

^{13}C NMR (101 MHz, CDCl$_3$) δ ppm 161.1 (CON), 148.4 (*C*=CH$_2$), 147.0 (N=CH), 145.3 (ArC), 144.2 (ArC), 139.5 (ArC), 128.9 (ArCH x 2), 128.9 (ArCH), 128.2 (ArCH), 127.3 (ArCH), 126.5 (ArCH), 126.3 (ArCH x 2), 119.8 (ArC), 116.2 (C=*CH$_2$*), 46.2 (NCH$_2$), 34.6 (NCH$_2$*CH$_2$*), 22.0 (CH$_3$).

^{1}H NMR of 1l (400 MHz, CDCl$_3$)

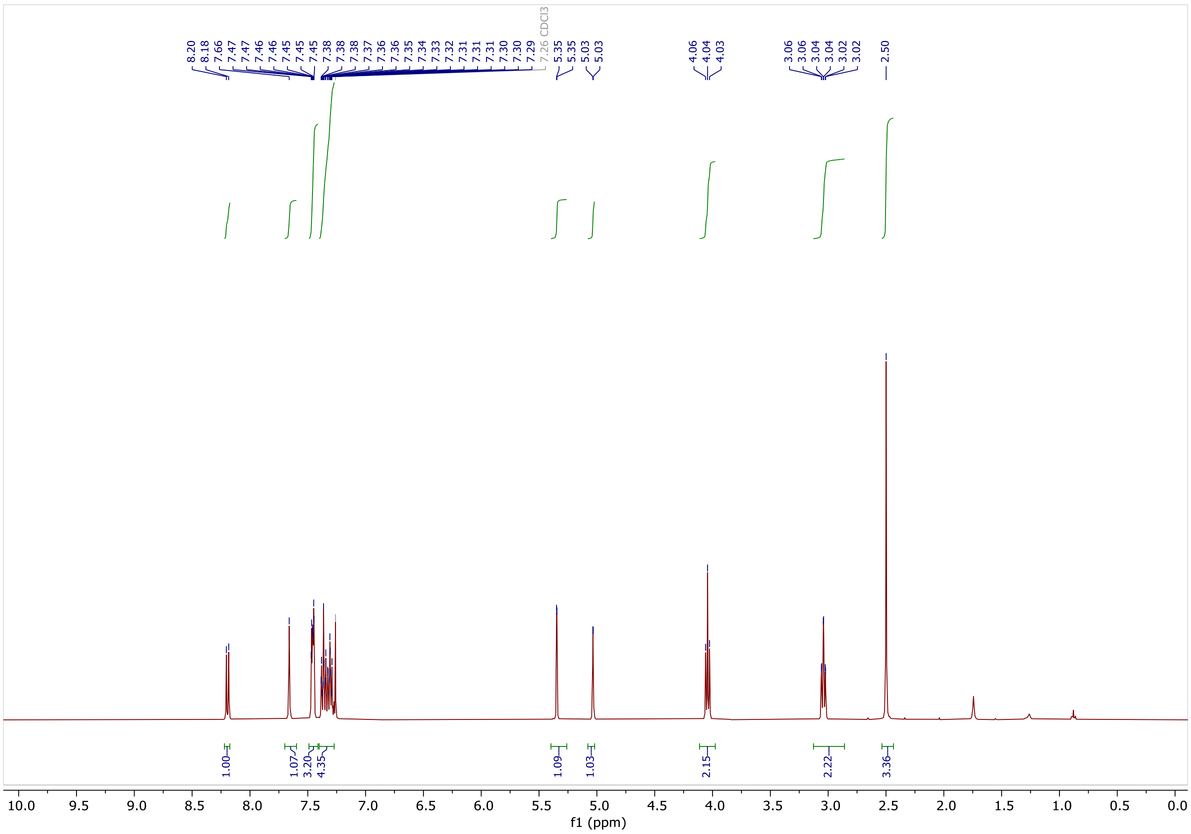

^{13}C NMR of 1l (101 MHz, CDCl$_3$)

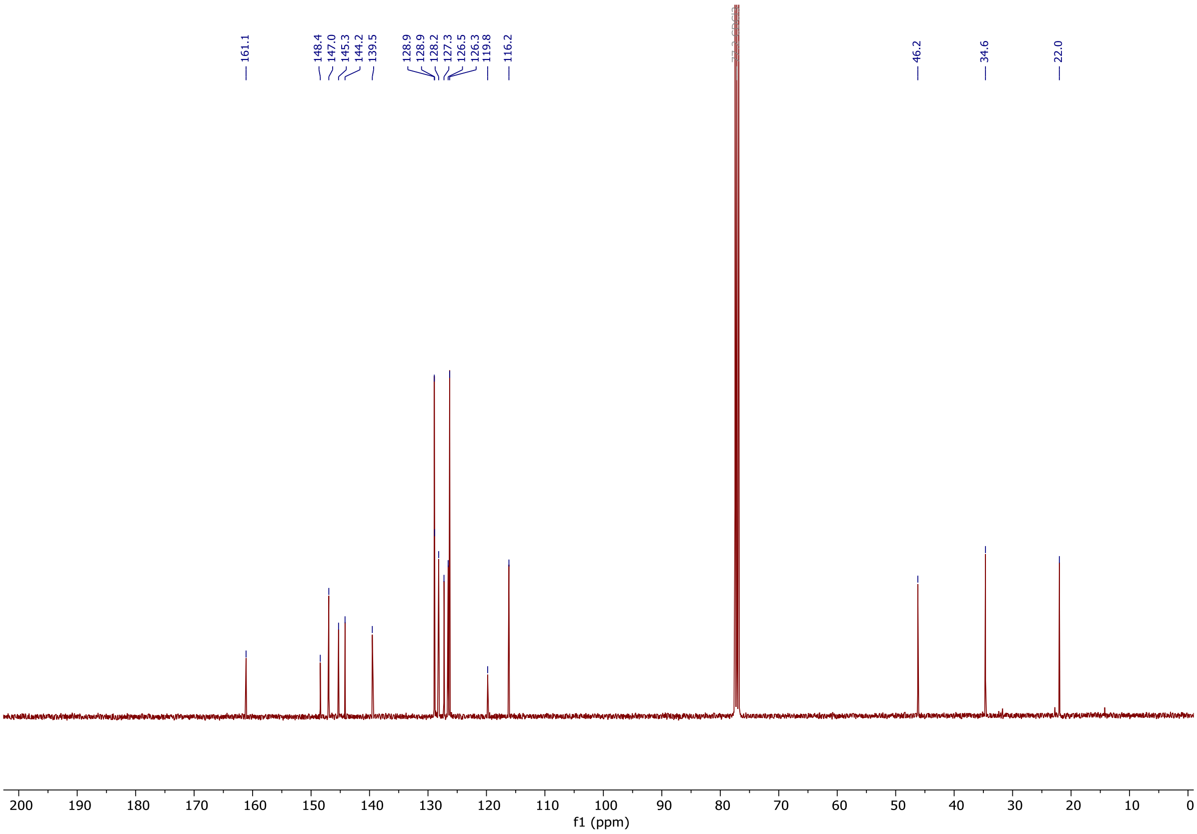

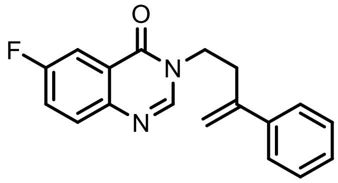

6-Fluoro-3-(3-phenylbut-3-en-1-yl)quinazolin-4(3*H*)-one (1m)

The title compound was synthesised following General Procedure B. **1m** was obtained in 36% yield (1 mmol scale, 0.357 mmol, 105 mg, 36%).

^{1}H NMR (500 MHz, CDCl$_3$) δ ppm 7.93 (dd, *J* = 8.7, 2.9 Hz, 1H, ArCH), 7.69 – 7.65 (m, 1H, ArCH), 7.64 (s, 1H, N=CH), 7.48 – 7.41 (m, 3H, ArCH), 7.38 – 7.32 (m, 2H, ArCH), 7.31 – 7.25 (m, 1H, ArCH), 5.35 (s, 1H, C=CH$_2$), 5.04 (s, 1H, C=CH$_2$), 4.06 (t, *J* = 6.7 Hz, 2H, NCH$_2$), 3.04 (t, *J* = 6.8 Hz, 2H, NCH$_2$C*H$_2$*).

^{13}C NMR (126 MHz, CDCl$_3$) δ ppm 161.2 (d, $^1J_{C\text{-}F}$ = 248.8 Hz, ArC), 160.4 (d, $^4J_{C\text{-}F}$ = 3.4 Hz, CON), 146.2 (d, *J* = 2.4 Hz, N=CH), 144.9 (d, $^4J_{C\text{-}F}$ = 2.1 Hz, ArC), 144.1 (*C*=CH$_2$), 139.4 (ArC), 130.0 (d, $^3J_{C\text{-}F}$ = 8.3 Hz, ArCH), 128.9 (ArCH x 2), 128.2 (ArCH), 126.3 (ArCH x 2), 123.5 (d, $^3J_{C\text{-}F}$ = 8.8 Hz, ArC), 122.9 (d, $^2J_{C\text{-}F}$ = 24.3 Hz, ArCH), 116.3 (C=*CH$_2$*), 111.6 (d, $^2J_{C\text{-}F}$ = 23.5 Hz, ArCH), 46.4 (NCH$_2$), 34.4 (NCH$_2$*CH$_2$*).

^{1}H NMR of 1m (500 MHz, CDCl$_3$)

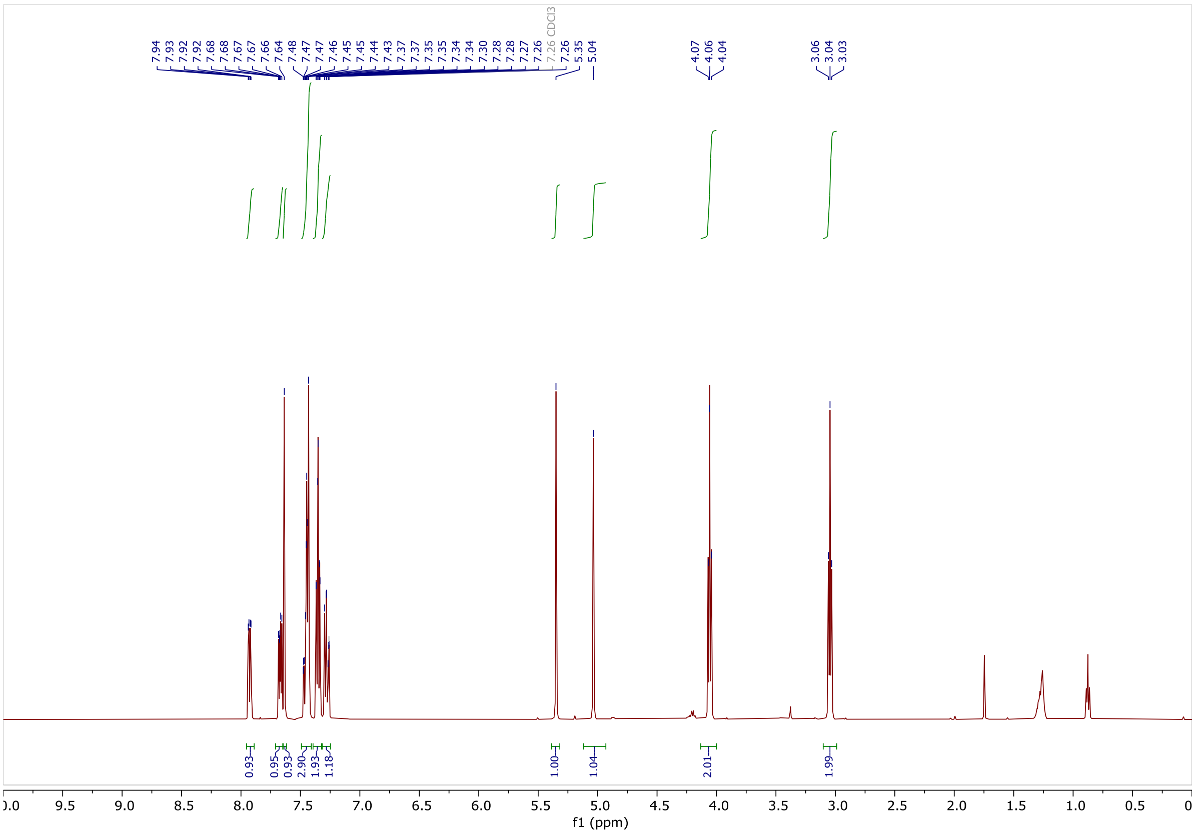

^{13}C NMR of 1m (126 MHz, $CDCl_3$)

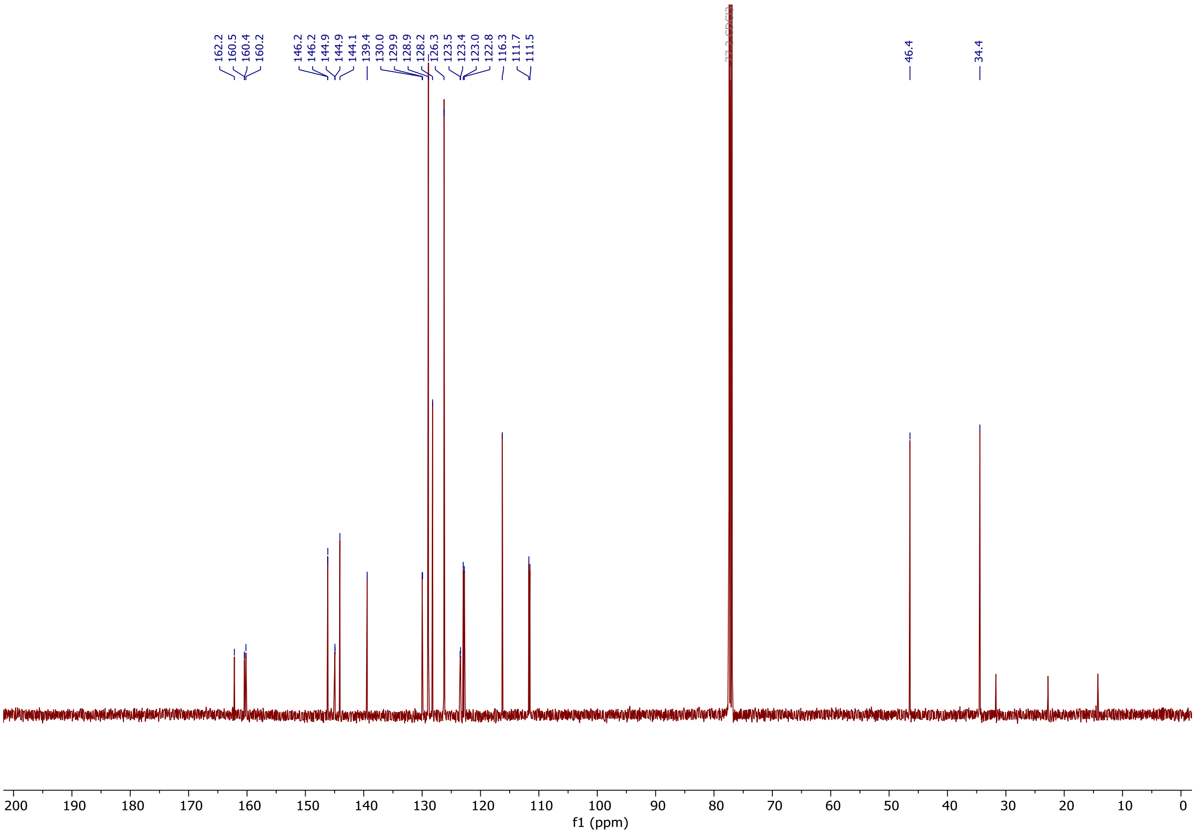

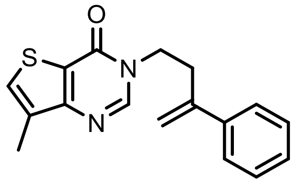

7-Methyl-3-(3-phenylbut-3-en-1-yl)thieno[3,2-d]pyrimidin-4(3*H*)-one (1n)

The title compound was synthesised following General Procedure A. **1n** was obtained in 37% yield (2 mmol scale, 0.738 mmol, 219 mg, 37%).

^{1}H NMR (400 MHz, $CDCl_3$) δ ppm 7.57 (s, 1H, N=CH), 7.48 – 7.43 (m, 2H, ArCH), 7.40 – 7.34 (m, 2H, ArCH), 7.34 – 7.27 (m, 1H, ArCH), 7.13 (d, *J* = 1.3 Hz, 1H, ArCH), 5.36 (d, *J* = 1.2 Hz, 1H, C=C*H₂*), 5.03 (d, *J* = 1.3 Hz, 1H, C=C*H₂*), 4.05 (t, *J* = 6.8 Hz, 2H, NCH_2), 3.03 (t, *J* = 6.7 Hz, 2H, NCH_2CH_2), 2.54 (d, *J* = 1.2 Hz, 3H, CH_3).

^{1}H NMR of 1n (400 MHz, $CDCl_3$)

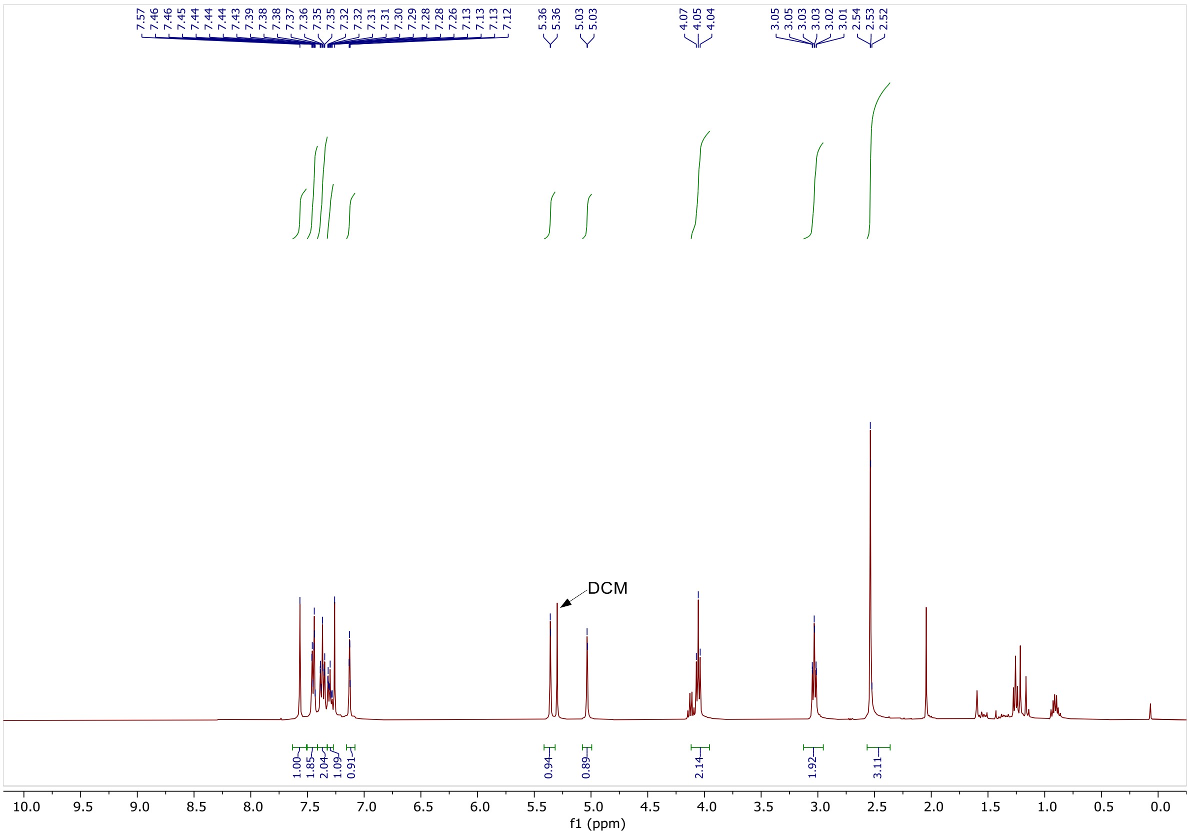

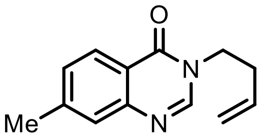

3-(But-3-en-1-yl)-7-methylquinazolin-4(3H)-one (1o)

The title compound was synthesised following General Procedure B. **1o** was obtained in 73% yield (1.63 mmol scale, 1.19 mmol, 255 mg, 73%).

^{1}H NMR (400 MHz, $CDCl_3$) δ ppm 8.19 (d, *J* = 8.1 Hz, 1H, ArCH), 7.96 (s, 1H, N=CH), 7.48 (d, *J* = 1.6 Hz, 1H, ArCH), 7.32 (dd, *J* = 8.2, 1.6 Hz, 1H, ArCH), 5.80 (ddt, *J* = 17.2, 10.4, 7.0 Hz, 1H, CH_2=CH), 5.12 – 5.02 (m, 2H, CH=CH_2), 4.05 (t, *J* = 7.0 Hz, 2H, NCH_2), 2.55 (qt, *J* = 7.1, 1.3 Hz, 2H, NCH_2CH_2), 2.50 (s, 3H, CH_3).

^{13}C NMR (101 MHz, $CDCl_3$) δ ppm 161.1 (CON), 148.4 (ArC), 146.8 (N=CH), 145.3 (ArC), 133.7 (CH_2=*CH*), 128.9 (ArCH), 127.3 (ArCH), 126.6 (ArCH), 119.8 (ArC), 118.8 (C=CH_2), 46.5 (NCH_2), 33.5 (NCH_2CH_2), 22.0 (CH_3).

^{1}H NMR of 1o (400 MHz, $CDCl_3$)

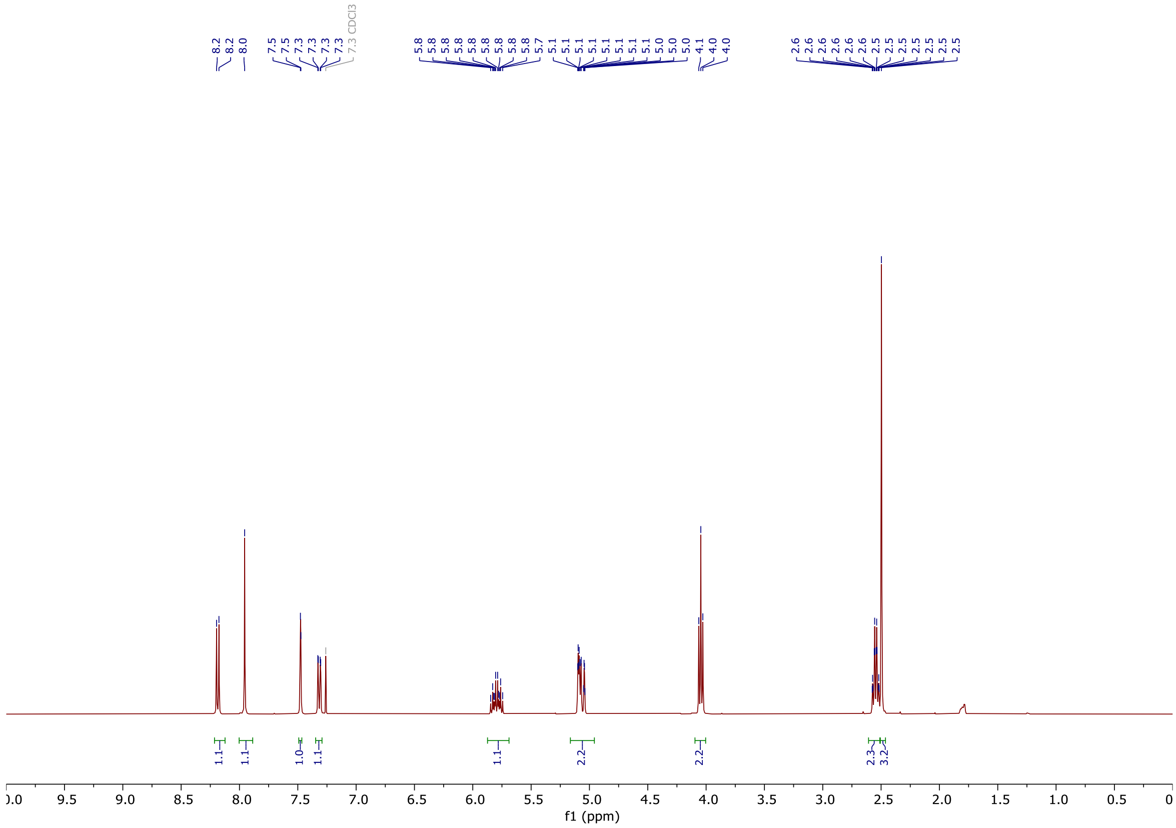

^{13}C NMR of 1o (101 MHz, $CDCl_3$)

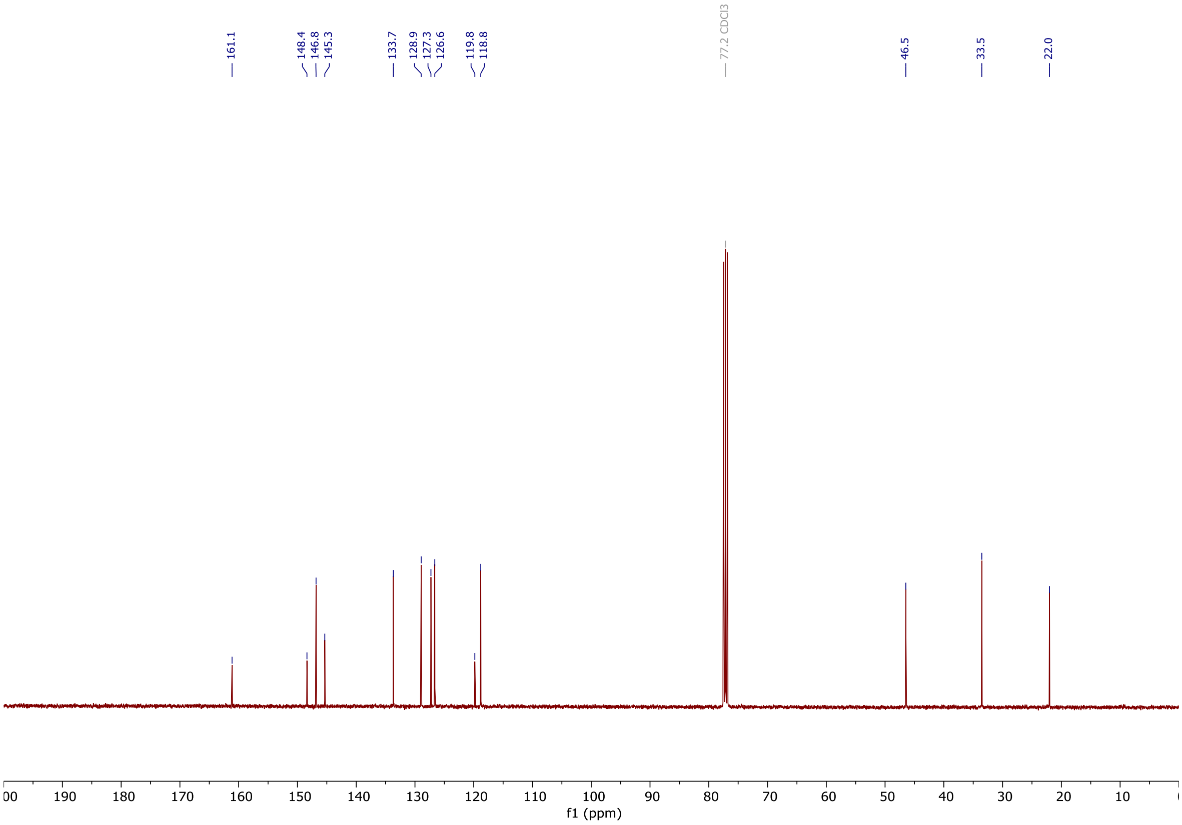

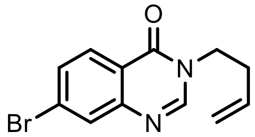

7-Bromo-3-(but-3-en-1-yl)quinazolin-4(3*H*)-one (1p)

The title compound was synthesised following General Procedure B. **1p** was obtained in 72% yield (2 mmol scale, 1.44 mmol, 401 mg, 72%).

^{1}H NMR (400 MHz, $CDCl_3$) δ ppm 8.15 (d, *J* = 8.5 Hz, 1H, ArCH), 7.97 (s, 1H, N=CH), 7.87 (d, *J* = 1.9 Hz, 1H, ArCH), 7.59 (dd, *J* = 8.5, 1.9 Hz, 1H, ArCH), 5.79 (ddt, *J* = 17.2, 10.3, 7.0 Hz, 1H, CH_2=*CH*), 5.13 – 5.03 (m, 2H, CH=*CH_2*), 4.05 (t, *J* = 6.9 Hz, 2H, NCH_2), 2.55 (qt, *J* = 6.9, 1.3 Hz, 2H, NCH_2*CH_2*).

^{13}C NMR (101 MHz, $CDCl_3$) δ ppm 160.7 (CON), 149.3 (ArC), 147.8 (N=CH), 133.4 (CH_2=*CH*), 130.7 (ArCH), 130.3 (ArCH), 129.0 (ArC), 128.3 (ArCH), 121.1 (ArC), 119.0 (CH=*CH_2*), 46.6 (NCH_2), 33.4 (NCH_2*CH_2*).

^{1}H NMR of 1p (400 MHz, $CDCl_3$)

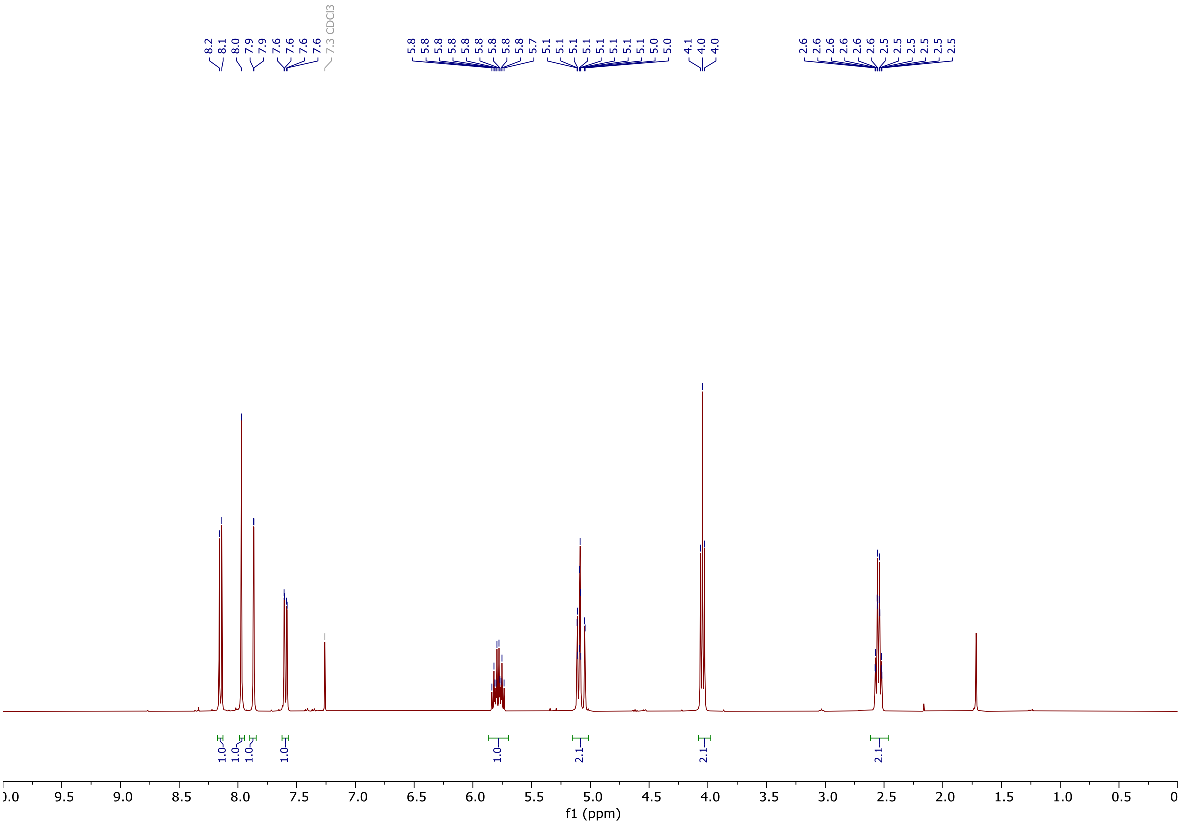

^{13}C NMR of 1p (101 MHz, CDCl$_3$)

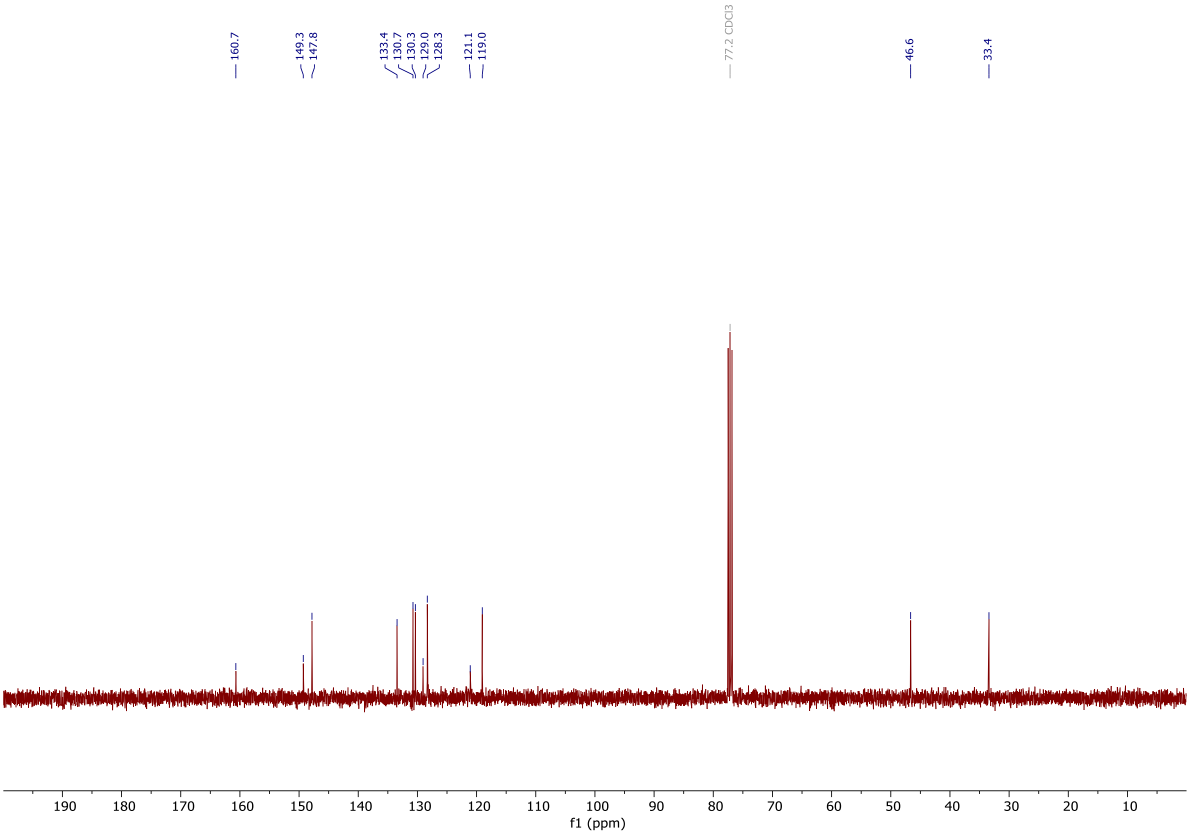

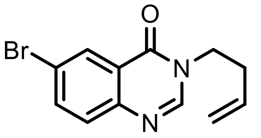

6-Bromo-3-(but-3-en-1-yl)quinazolin-4(3H)-one (1q)

The title compound was synthesised following General Procedure B. **1q** was obtained in 73% yield (0.71 mmol scale, 0.52 mmol, 145 mg, 73%).

^{1}H NMR (400 MHz, CDCl$_3$) δ ppm 8.43 (dd, J = 2.3, 0.9 Hz, 1H, ArCH), 7.98 (s, 1H, N=CH), 7.82 (ddd, J = 8.6, 2.4, 0.8 Hz, 1H, ArCH), 7.57 (d, J = 8.7 Hz, 1H, ArCH), 5.79 (ddt, J = 17.2, 10.3, 7.0 Hz, 1H, *CH*=CH$_2$), 5.15 – 5.02 (m, 2H, CH=CH_2), 4.06 (t, J = 6.9 Hz, 2H, NCH$_2$), 2.55 (qt, J = 7.0, 1.3 Hz, 2H, NCH$_2$$CH_2$).

^{13}C NMR (101 MHz, CDCl$_3$) δ ppm 160.7 (CON), 149.3 (ArC), 147.8 (N=CH), 133.4 (CH$_2$=*CH*), 130.7 (ArCH), 130.3 (ArCH), 129.0 (ArC), 128.3 (ArCH), 121.1 (ArC), 119.0 (CH=CH_2), 46.6 (NCH$_2$), 33.4 (NCH$_2$$CH_2$).

^{1}H NMR of 1q (400 MHz, $CDCl_3$)

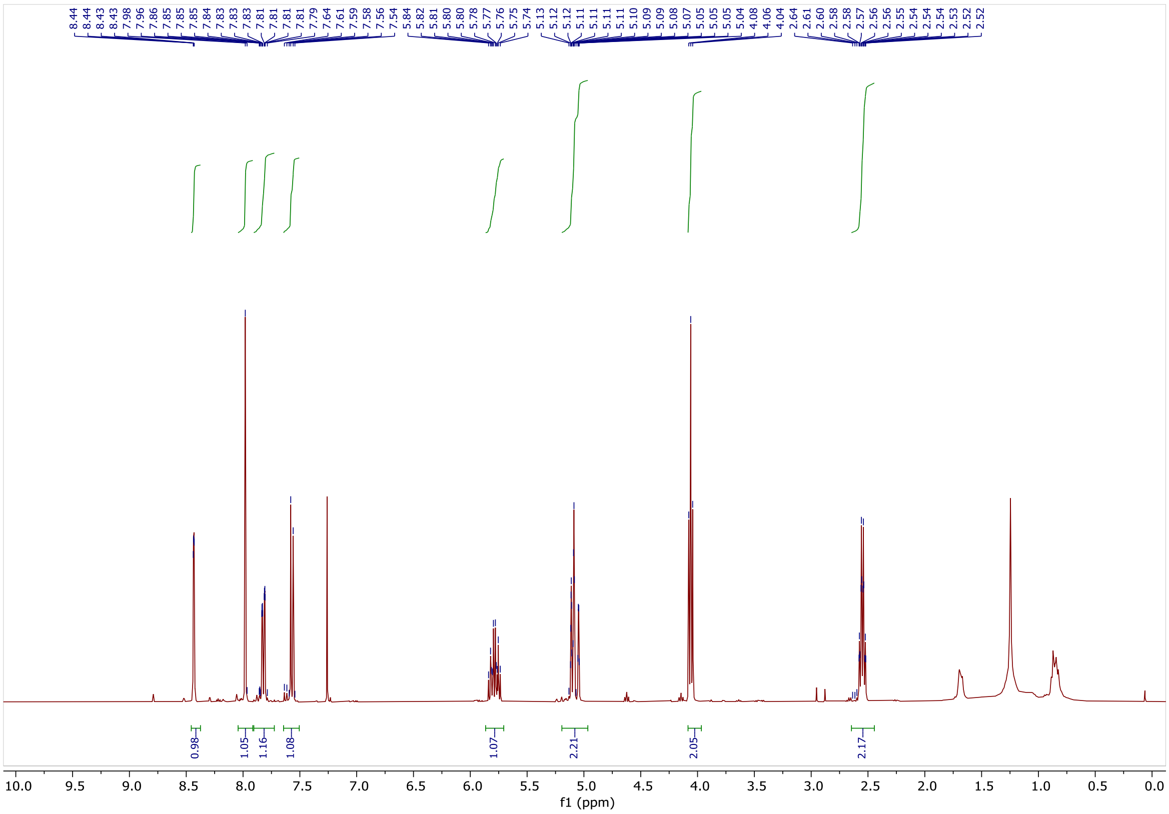

^{13}C NMR of 1q (101 MHz, $CDCl_3$)

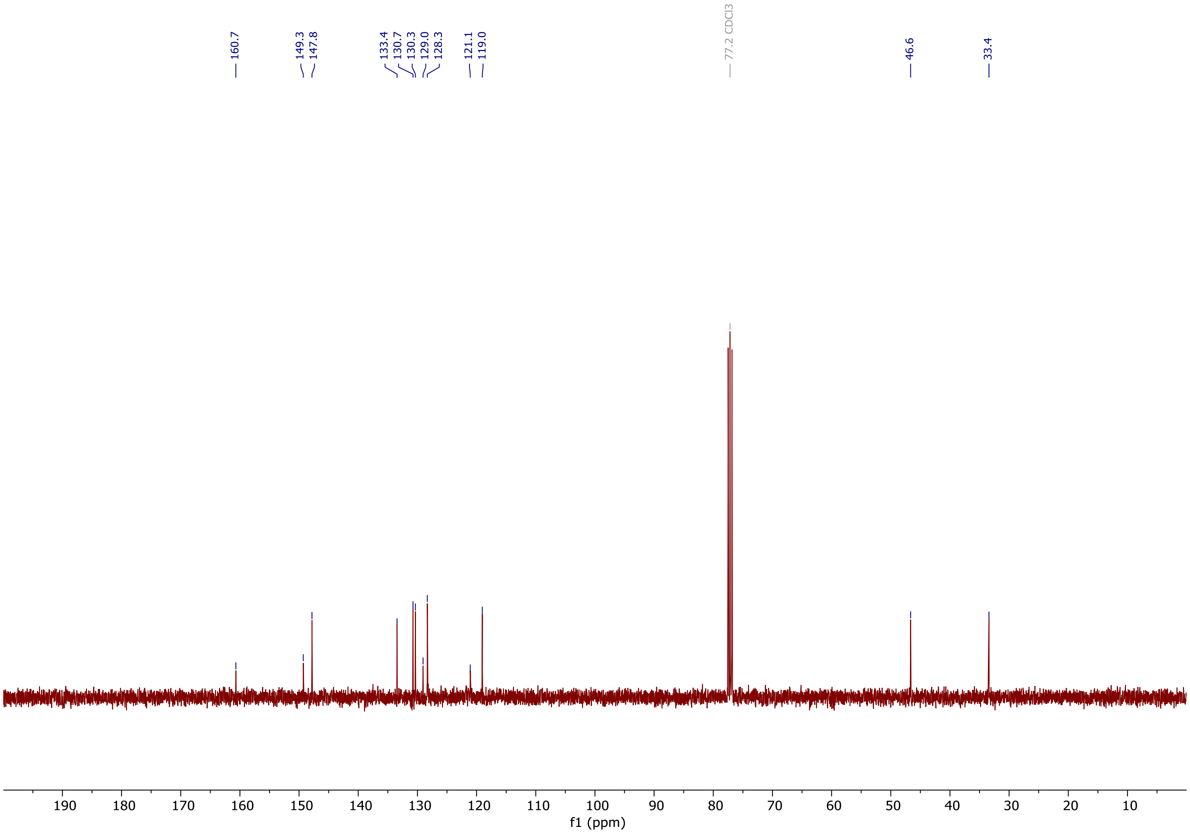

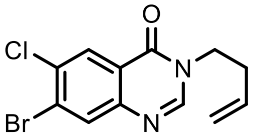

7-Bromo-3-(but-3-en-1-yl)-6-chloroquinazolin-4(3H)-one (1r)

The title compound was synthesised following General Procedure B. **1r** was obtained in 95% yield (1 mmol scale, 0.95 mmol, 298 mg, 95%).

^{1}H NMR (400 MHz, $CDCl_3$) δ ppm 8.35 (d, *J* = 1.1 Hz, 1H, ArCH), 8.00 (d, *J* = 0.9 Hz, 1H, ArCH), 7.95 (s, 1H, N=CH), 5.78 (ddt, J = 17.2, 10.3, 7.0 Hz, 1H, CH_2=*CH*), 5.13 – 5.03 (m, 2H, CH=*CH_2*), 4.05 (t, *J* = 6.9 Hz, 2H, NCH_2), 2.54 (qt, *J* = 7.0, 1.3 Hz, 2H, NCH_2*CH_2*).

^{13}C NMR (101 MHz, $CDCl_3$) δ ppm 159.8 (CON), 147.9 (ArC), 147.3 (N=CH), 133.7 (ArC), 133.3 (ArCH), 132.7 (CH_2=*CH*), 129.4 (ArC), 127.8 (ArCH), 122.2 (ArC), 119.2 (CH=*CH_2*), 46.8 (NCH_2), 33.4 (NCH_2*CH_2*).

^{1}H NMR of 1r (400 MHz, $CDCl_3$)

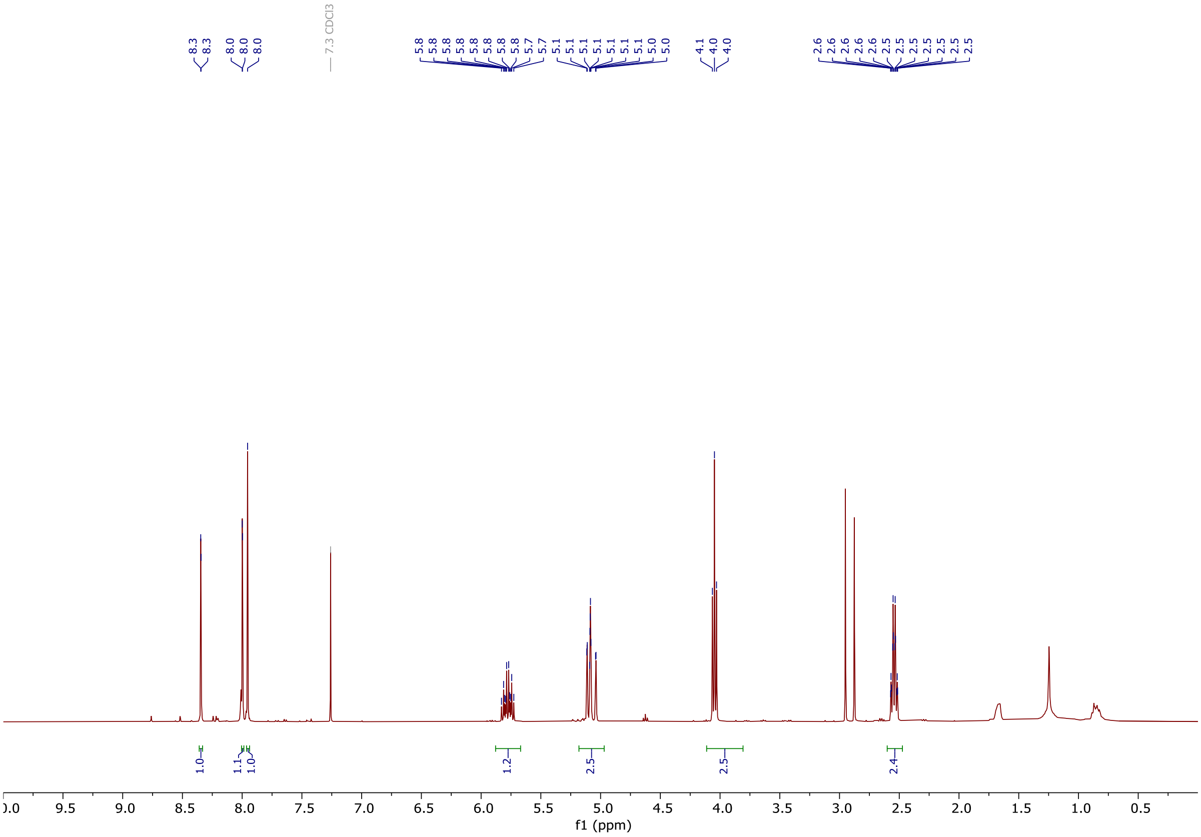

^{13}C NMR of 1r (101 MHz, $CDCl_3$)

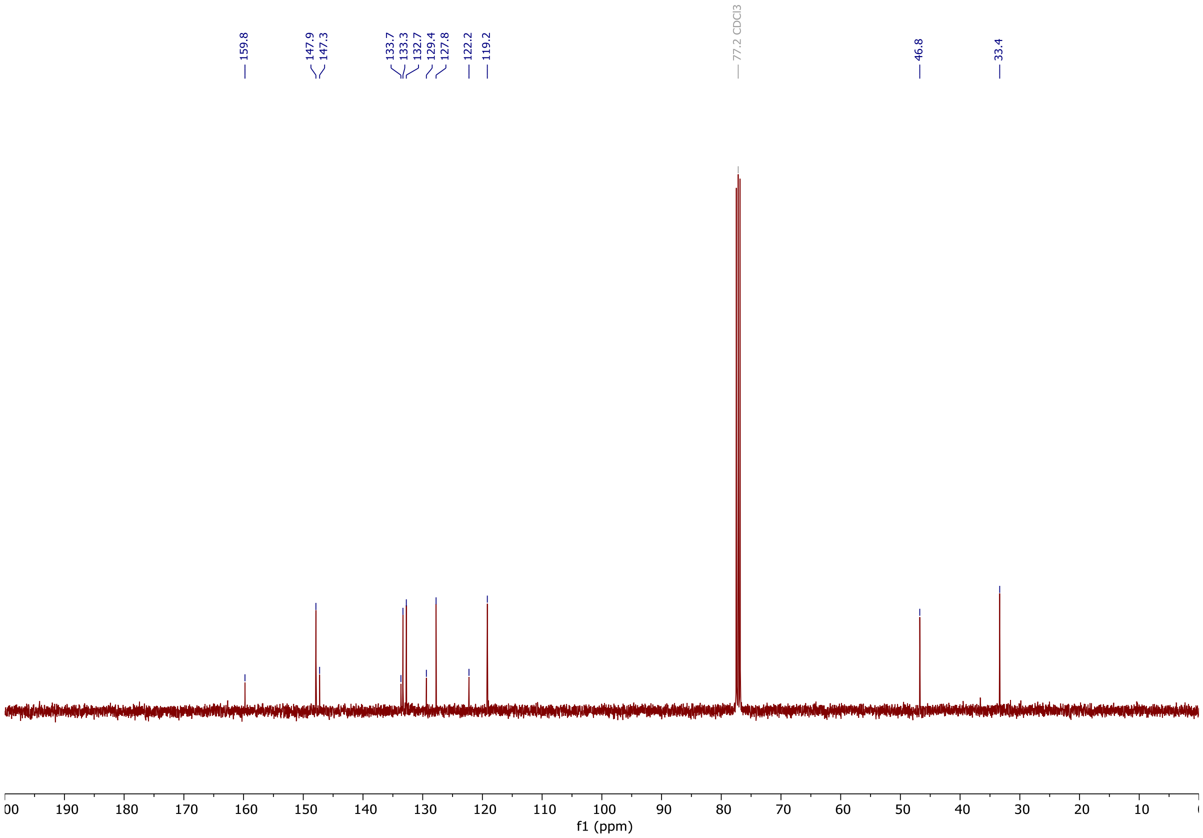

Characterization and NMR data for products 2

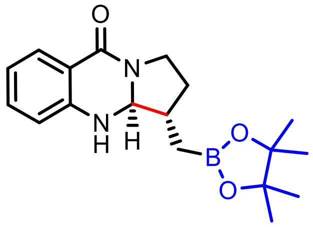

Chemical Formula: $C_{18}H_{25}BN_2O_3$
Molecular Weight: 328.2190

(3*S*,3a*S*)-3-((4,4,5,5-Tetramethyl-1,3,2-dioxaborolan-2-yl)methyl)-2,3,3a,4-tetrahydropyrrolo[2,1-*b*]quinazolin-9(1*H*)-one (2a)

Compound **2a** was prepared according to General Procedure D, running the reaction at 25 °C. The title compound was isolated by column chromatography (hexane: ethyl acetate, 80:20 - 50:50) as a white solid (41.4 mg, 0.126 mmol, 63%, dr >95:5).

^1H NMR (400 MHz, $CDCl_3$) δ (ppm) = 7.87 (dd, *J* = 7.8, 1.6 Hz, 1H, ArCH), 7.32 – 7.14 (m, 1H, ArCH), 6.83 (td, *J* = 7.5, 1.0 Hz, 1H, ArCH), 6.60 (dd, *J* = 8.1, 1.0 Hz, 1H, ArCH), 5.77 (s, 1H, NH), 4.57 (d, *J* = 8.1 Hz, 1H, NHC*H*), 3.75 (ddd, *J* = 11.4, 9.3, 1.8 Hz, 1H, NCH_2), 3.68 – 3.56 (m, 1H,

NCH_2), 2.43 – 2.30 (m, 1H, NCH_2CH_2), 2.25 – 2.14 (m, 1H, NHCHC*H*), 1.68 – 1.55 (m, 1H, NCH_2CH_2), 1.25 – 1.14 (m, 1H, BCH_2), 1.32 (s, 12H, CH_3 x 4), 1.00 – 0.84 (m, 1H, BCH_2).

^{13}C NMR (101 MHz, $CDCl_3$) δ ppm 162.4 (CON), 147.9 (ArC), 132.9 (ArCH), 128.3 (ArCH), 119.3 (ArCH), 118.1 (ArCH), 114.4 (ArC), 84.0 (Cq x 2), 75.9 (NCH), 43.1 (NCH_2), 41.7 (NHCHCH), 31.5 (NCH_2CH_2), 25.1 (CH_3 x 2), 25.0 (CH_3 x 2). The signal for CH_2Bpin was not observed.

^{11}B NMR (128 MHz, $CDCl_3$) δ ppm 33.45.

IR ν_{max} (neat/cm^{-1}): 3056, 2976, 1633, 1611, 1363, 1324, 1142, 849, 750.

Mp: 198 °C.

HRMS: calculated for $C_{18}H_{25}N_2O_3BNa$ $[M + Na]^+$ 351.1840, found 351.1850.

Specific rotation: $[\alpha]_D^{22}$ + 22.84 (c 2.94, $CHCl_3$).

Enantiomeric purity of **2a** was determined by HPLC analysis in comparison with authentic racemic material (er = 93:7; **IA** column, 90:10 hexanes: *i*PrOH, 0.5 mL/min, 20 °C, 254 nm).

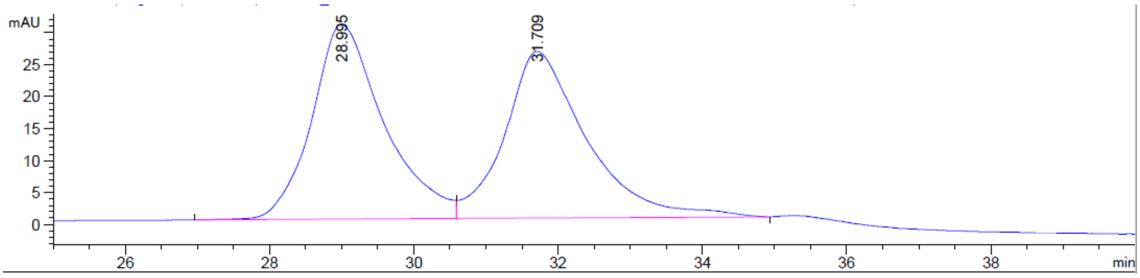

Peak #	RetTime [min]	Type	Width [min]	Area [mAU*s]	Height [mAU]	Area %
1	28.995	BV	1.0076	2121.06934	30.41676	50.7540
2	31.709	VB	1.1054	2058.04785	26.06002	49.2460

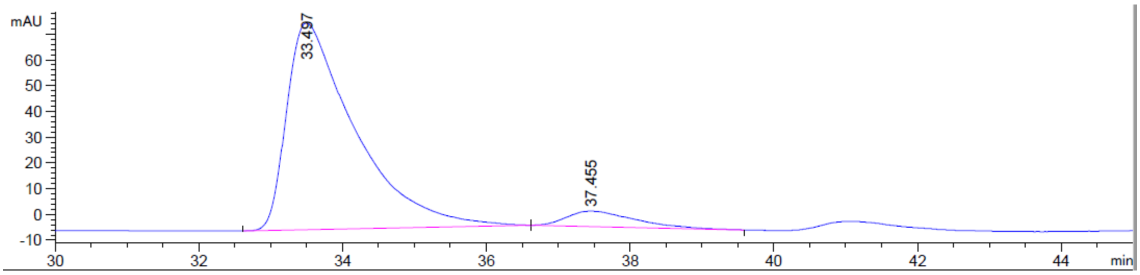

Peak #	RetTime [min]	Type	Width [min]	Area [mAU*s]	Height [mAU]	Area %
1	33.497	BB	0.9444	5292.93555	80.14960	93.1610
2	37.455	BB	0.7758	388.55487	5.98172	6.8390

^{1}H NMR of 2a (400 MHz, $CDCl_3$)

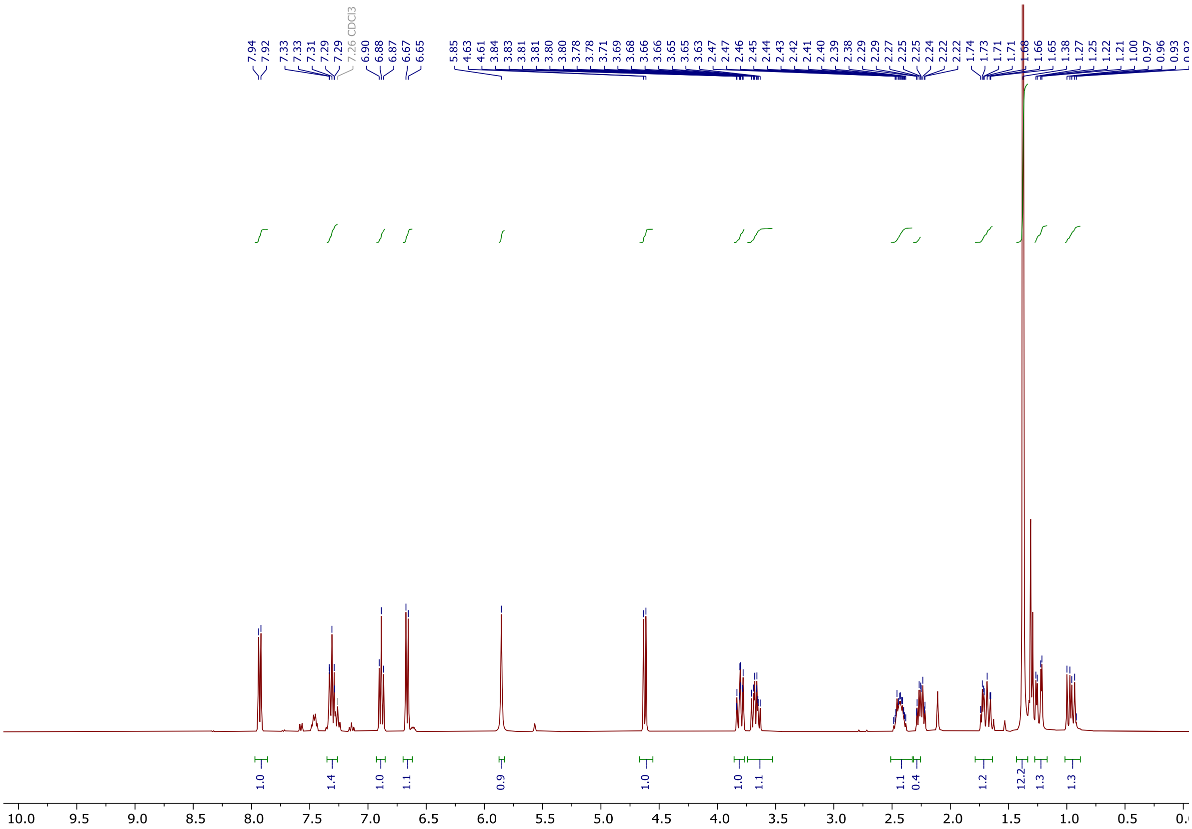

^{13}C NMR of 2a (101 MHz, $CDCl_3$)

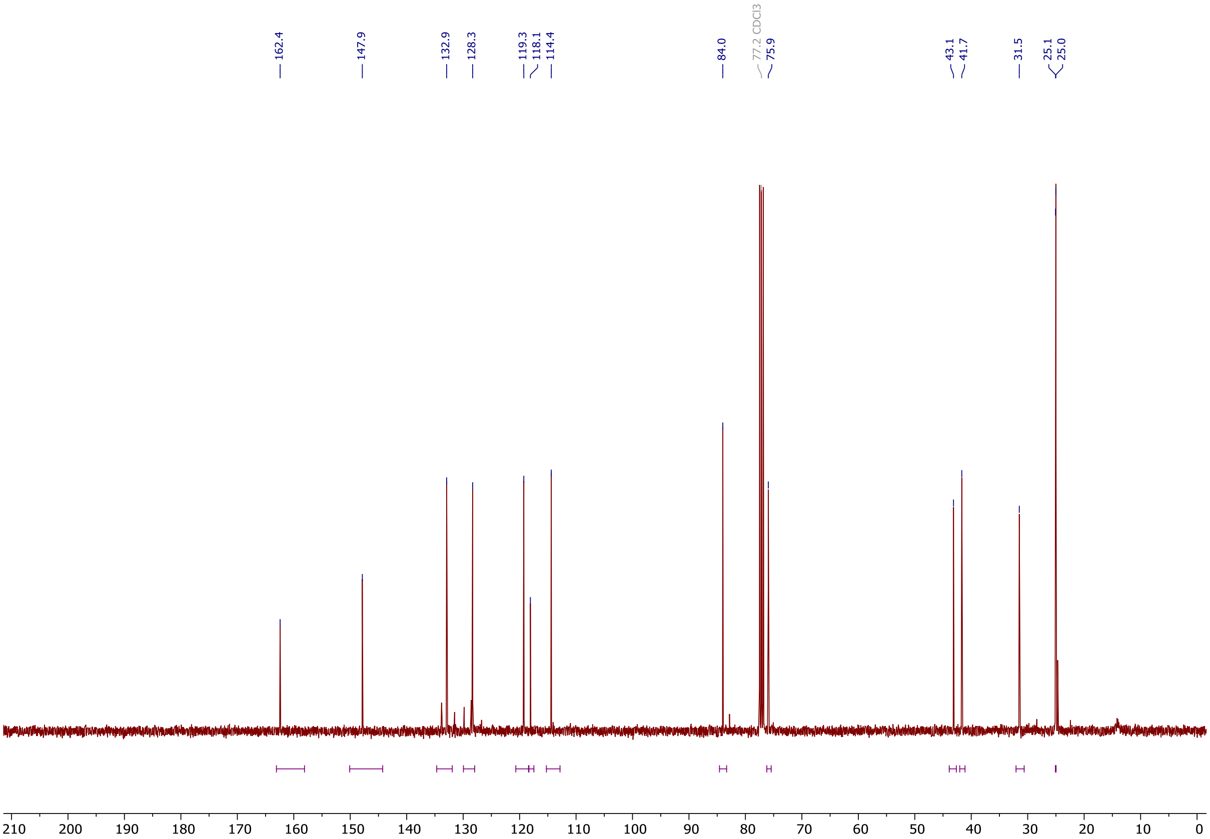

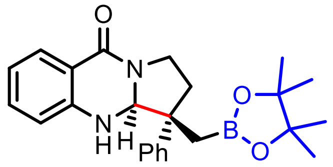

Chemical Formula: $C_{24}H_{29}BN_2O_3$
Molecular Weight: 404.3170

(3*S*,3a*S*)-3-Phenyl-3-((4,4,5,5-tetramethyl-1,3,2-dioxaborolan-2-yl)methyl)-2,3,3a,4-tetrahydropyrrolo[2,1-*b*]quinazolin-9(1*H*)-one (2b)

Compound **2b** was prepared according to General Procedure C, running the reaction at 25 °C. The title compound was isolated by column chromatography (hexane: ethyl acetate, 80:20 - 40:60) as a white amorphous solid (60.6 mg, 0.150 mmol, 75%, dr = 91:9).

^{1}H NMR (500 MHz, $CDCl_3$) δ ppm 7.93 (dd, *J* = 7.7, 1.5 Hz, 1H, ArCH), 7.37 – 7.30 (m, 4H, ArCH), 7.31 – 7.20 (m, 2H, ArCH), 6.83 (td, *J* = 7.5, 1.0 Hz, 1H, ArCH), 6.64 (dd, *J* = 8.0, 1.0 Hz, 1H, ArCH), 5.53 (s, 1H, NH), 5.35 (d, *J* = 1.1 Hz, 1H, NHC*H*), 3.82 (ddd, *J* = 11.9, 9.3, 1.4 Hz, 1H, NCH_2), 3.71 (ddd, *J* = 12.1, 10.9, 7.2 Hz, 1H, NCH_2), 2.35 (ddd, *J* = 12.7, 7.2, 1.5 Hz, 1H, NCH_2CH_2), 2.19 (ddd, *J* = 12.8, 10.9, 9.3 Hz, 1H, NCH_2CH_2), 1.78 (d, *J* = 16.0 Hz, 1H, BCH_2), 1.31 (d, *J* = 16.0 Hz, 1H, BCH_2), 1.03 (s, 6H, CH_3 x 2), 0.96 (s, 6H, CH_3 x 2).

^{13}C NMR (126 MHz, $CDCl_3$) δ 163.0 (CON), 147.2 (ArC), 144.5 (ArC), 133.1 (ArCH), 128.6 (ArCH x 2), 128.3 (ArCH), 126.8 (ArCH), 125.9 (ArCH x 2), 118.8 (ArCH), 117.2 (ArC), 114.7 (ArCH), 83.4 (Cq x 2), 75.1 (NCH), 50.8 (NCH_2), 41.6 (NCH_2CH_2), 38.0 (Cq), 24.6 (CH_3 x 2), 24.5 (CH_3 x 2). The signal for B*C*H_2 was not observed.

^{11}B NMR (160 MHz, $CDCl_3$) δ ppm 32.80.

IR ν_{max} (neat/cm^{-1}): 3315, 2975, 1628, 1612, 1367, 1327, 1142, 847, 751.

HRMS: calculated for $C_{24}H_{29}N_2O_3BNa$ $[M + Na]^+$ 427.2140, found 427.2163.

Specific rotation: $[\alpha]_D^{23}$ + 85.50 (c 0.12, $CHCl_3$).

Enantiomeric purity of **2b** was determined by HPLC analysis in comparison with authentic racemic material (er = 95:5; **IA** column, 90:10 hexane: *i*PrOH, 0.5 mL/min, 20 °C, 254 nm)

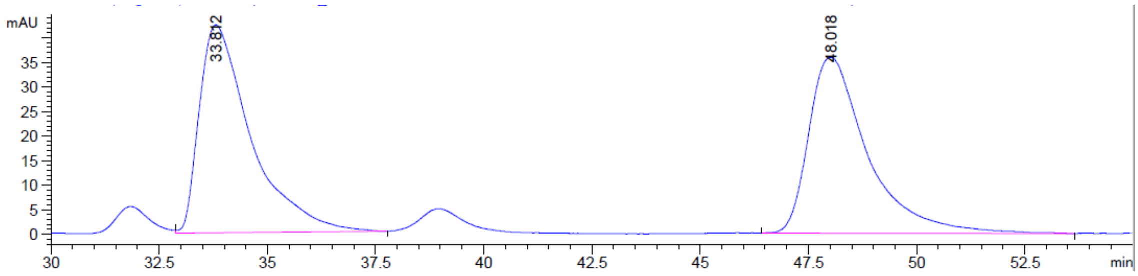

Peak #	RetTime [min]	Type	Width [min]	Area [mAU*s]	Height [mAU]	Area %
1	33.812	VB	1.2024	3496.40698	42.29721	50.7659
2	48.018	BB	1.3494	3390.90698	35.78976	49.2341

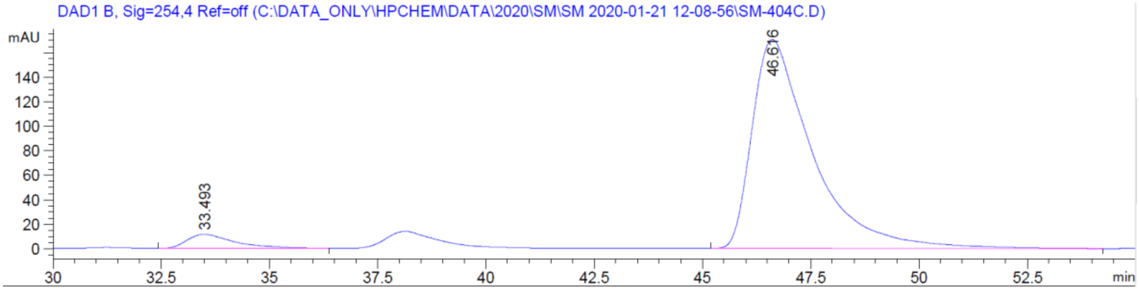

Peak #	RetTime [min]	Type	Width [min]	Area [mAU*s]	Height [mAU]	Area %
1	33.493	BB	1.0739	843.07208	11.35961	4.8688
2	46.616	BB	1.3856	1.64729e4	170.12840	95.1312

^{1}H NMR of 2b (500 MHz, $CDCl_3$)

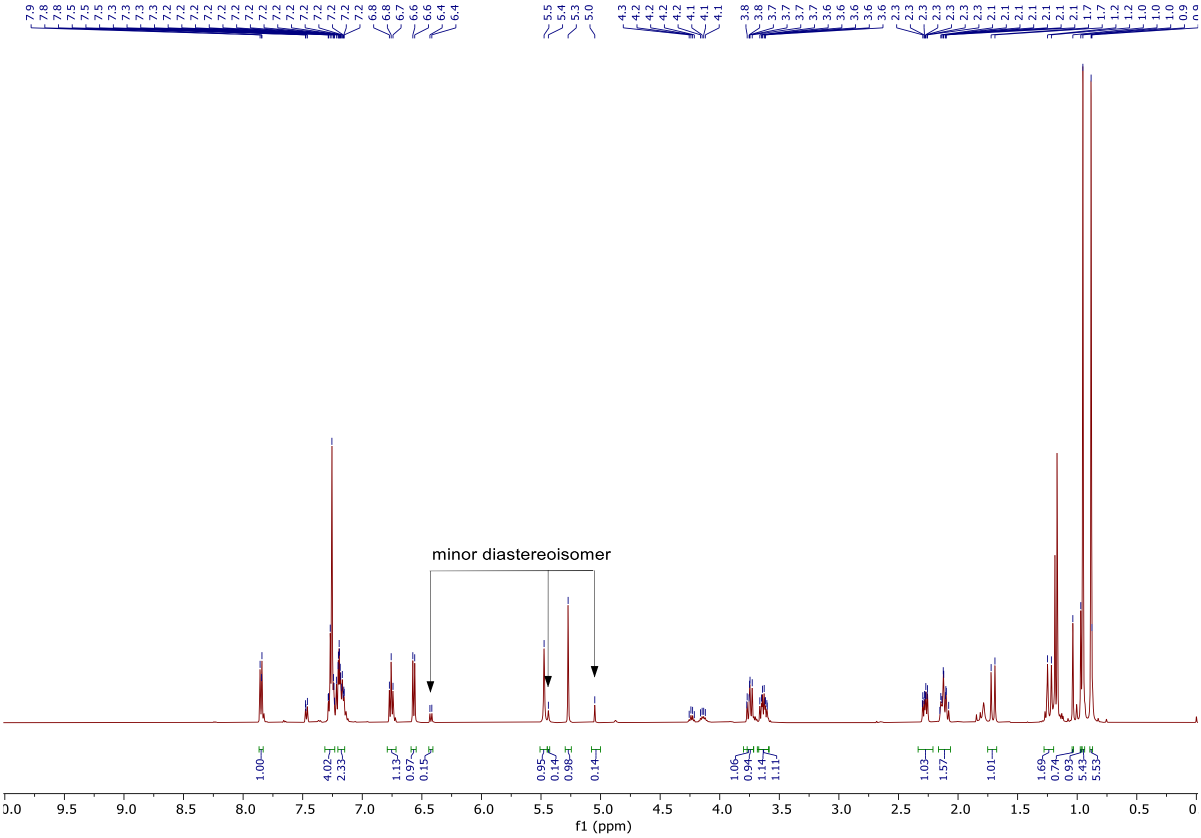

^{13}C NMR of 2b (126 MHz, $CDCl_3$)

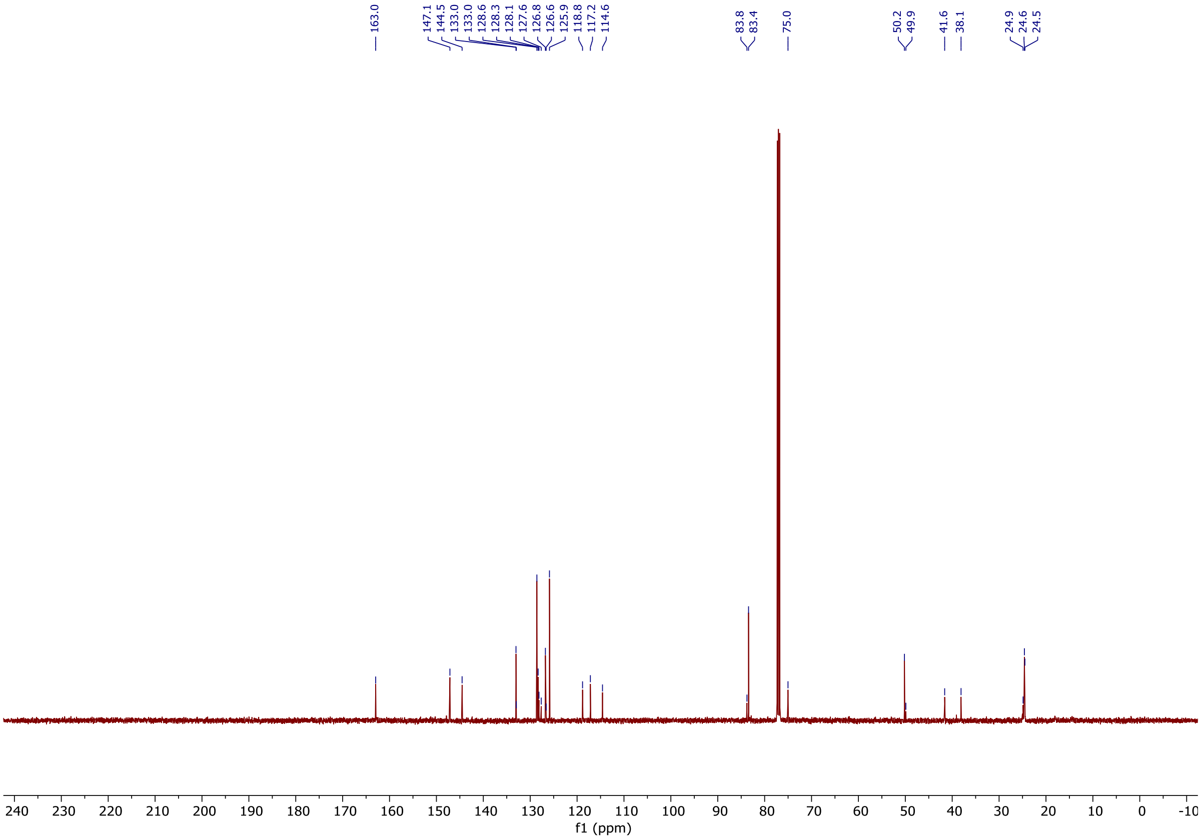

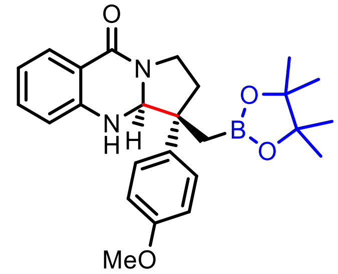

Chemical Formula: $C_{25}H_{31}BN_2O_4$
Molecular Weight: 434.3430

(3*S*,3a*S*)-3-(4-Methoxyphenyl)-3-((4,4,5,5-tetramethyl-1,3,2-dioxaborolan-2-yl)methyl)-2,3,3a,4-tetrahydropyrrolo[2,1-*b*]quinazolin-9(1H)-one (2c)

Compound **2c** was prepared according to General Procedure C, running the reaction at 25 °C. The title compound was isolated by column chromatography (hexane: diethyl ether, 25:75 to 0:100) as a white solid (81 mg, 0.186 mmol, 95%, dr = 93:7).

^{1}H NMR (400 MHz, 400 MHz, $CDCl_3$) δ ppm 7.92 (d, *J* = 7.6 Hz, 1H, ArC*H*), 7.31 – 7.21 (m, 3H, ArC*H* + 2 x ArC*H*), 6.88 (d, *J* = 8.5 Hz, 2H, ArC*H*), 6.83 (t, *J* = 7.5 Hz, 1H, ArC*H*), 6.63 (d, *J* = 7.9 Hz, 1H, ArC*H*), 5.43 (s, 1H, N*H*), 5.28 (s, 1H, NHC*H*), 3.85 – 3.76 (m, 1H, NCH_2), 3.80 (s, 3H,

OCH_3), 3.69 (td, J = 11.4, 7.1 Hz, 1H, NCH_2), 2.33 (dd, J = 12.8, 7.0 Hz, 1H, NCH_2CH_2), 2.20 – 2.12 (m, 1H, NCH_2CH_2), 1.74 (d, J = 16.0 Hz, 1H, BCH_2), 1.27 (d, J = 16.2 Hz, 1H, BCH_2), 1.04 (s, 6H, CH_3 x 2), 0.99 (s, 6H, CH_3 x 2).

^{13}C NMR (101 MHz, $CDCl_3$) δ ppm 163.0 (*C*ON), 158.4 (Ar*C*), 147.3 (Ar*C*), 136.7 (Ar*C*), 133.1 (Ar*C*H), 128.4 (Ar*C*H), 127.1 (Ar*C*H x 2), 119.0 (Ar*C*H), 117.4 (Ar*C*), 114.7 (Ar*C*H x 2), 114.0 (Ar*C*H), 83.5 (*C*q x 2), 75.6 (NH*C*H), 55.5 (OCH_3), 49.7 (NCH_2), 41.8 (*C*q), 38.2 (NCH_2CH_2), 24.8 (CH_3 x 2), 24.7 (CH_3 x 2). The signal for BCH_2 was not observed.

^{11}B NMR (128 MHz, $CDCl_3$) δ ppm 33.41 .

IR ν_{max} (neat/cm^{-1}): 3271, 2975, 2930, 1636, 1610, 1361, 1249, 1141, 1032, 753.

Mp: 175 °C

HRMS : calculated for $C_{25}H_{32}O_4N_2B$ [M + H]$^+$ 435.2450, found 435.2448.

Specific rotation: $[\alpha]_D^{24}$ +76.75 (c 0.08, $CHCl_3$).

Enantiomeric purity of **2c** was determined by HPLC analysis in comparison with authentic racemic material (er = 92:8; **IA** column, 85:15 hexanes: *i*PrOH, 0.5 mL/min, 20 °C, 254 nm)

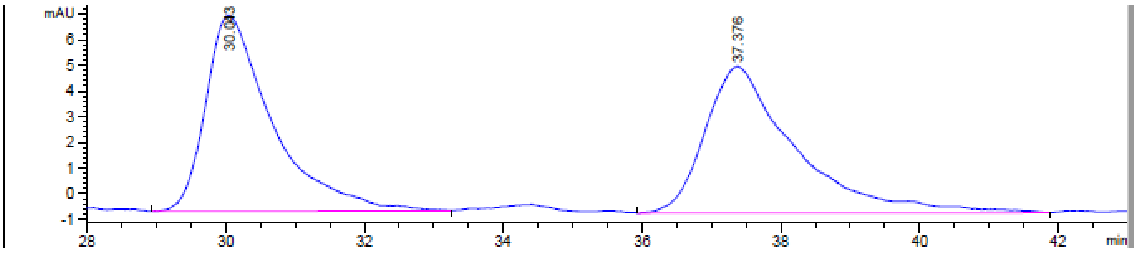

Peak #	RetTime [min]	Type	Width [min]	Area [mAU*s]	Height [mAU]	Area %
1	30.043	BB	0.9685	511.52444	7.64640	49.7376
2	37.376	BB	1.2837	516.92261	5.71279	50.2624

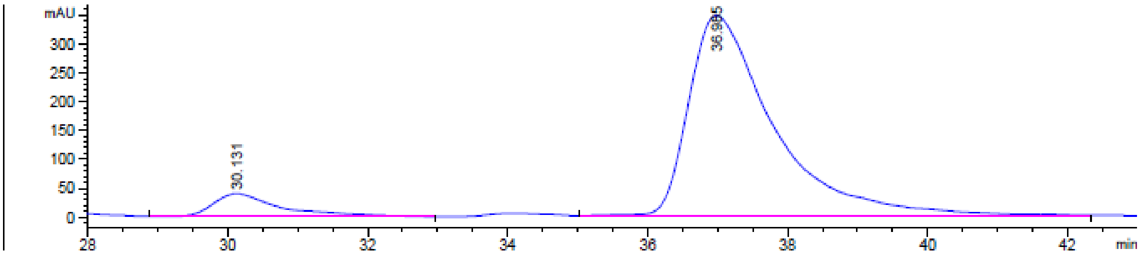

Peak #	RetTime [min]	Type	Width [min]	Area [mAU*s]	Height [mAU]	Area %
1	30.131	VB	0.9662	2658.89478	39.65800	8.1617
2	36.985	VV	1.2651	2.99188e4	348.65237	91.8383

^{1}H NMR of 2c (400 MHz, $CDCl_3$)

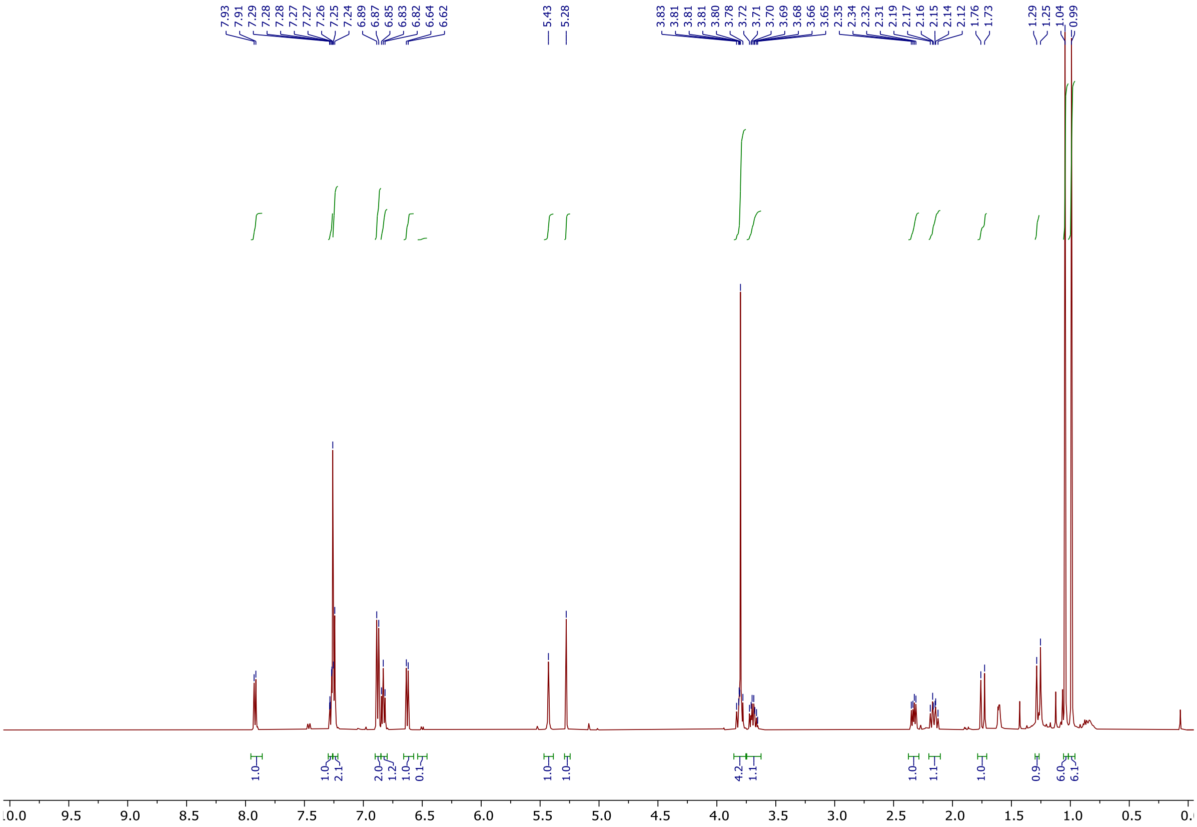

^{13}C NMR of 2c (101 MHz, $CDCl_3$)

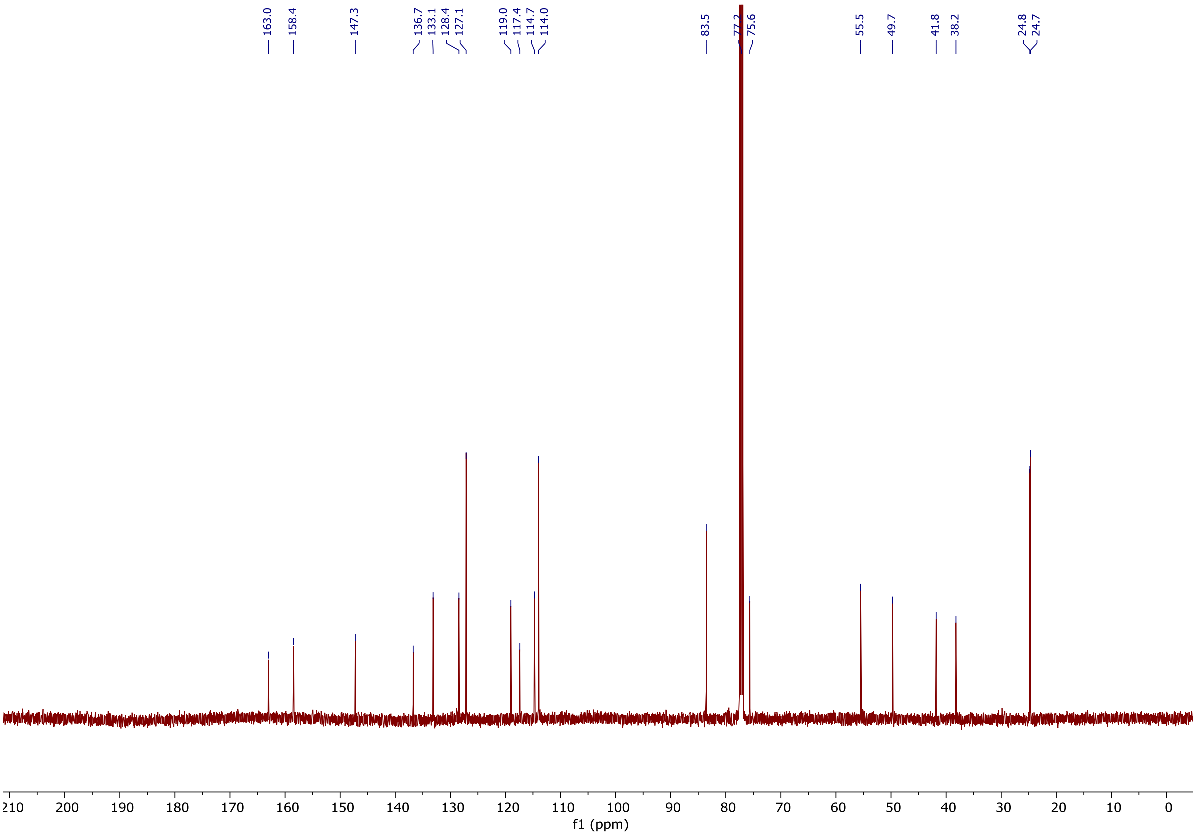

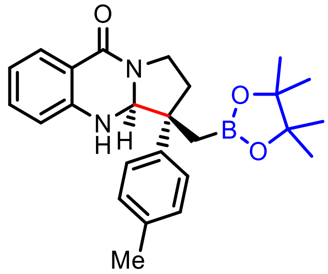

Chemical Formula: $C_{25}H_{31}BN_2O_3$
Molecular Weight: 418.34

(3*S*,3a*S*)-3-((4,4,5,5-Tetramethyl-1,3,2-dioxaborolan-2-yl)methyl)-3-(p-tolyl)-2,3,3a,4-tetrahydropyrrolo[2,1-*b*]quinazolin-9(1*H*)-one (2d)

Compound **2d** was prepared according to General Procedure C, running the reaction at 25 °C. The title compound was isolated by column chromatography (hexane: ethyl acetate, 80:20 - 50:50) as a white solid (61.0 mg, 0.146 mmol, 73%, dr = 91:9).

^{1}H NMR (400 MHz, $CDCl_3$) δ ppm 7.97 (d, *J* = 8.0 Hz, 1H, ArC*H*), 7.32 (t, *J* = 7.7 Hz, 1H, ArC*H*), 7.27 (d, *J* = 8.1 Hz, 2H, ArC*H* x 2), 7.20 (d, *J* = 7.8 Hz, 2H, ArC*H* x 2), 6.88 (t, *J* = 7.6 Hz, 1H, ArC*H*), 6.68 (d, *J* = 8.0 Hz, 1H, ArC*H*), 5.50 (s, 1H, N*H*), 5.36 (s, 1H, NHC*H*), 3.92 – 3.82 (m, 1H, NCH_2), 3.79 – 3.72 (m, 1H, NCH_2), 2.39 (s, 3H, CH_3), 2.43 – 2.34 (m, 1H, NCH_2CH_2), 2.22 (dd, *J* = 22.1, 11.0 Hz, 1H, NCH_2CH_2), 1.80 (d, *J* = 16.0 Hz, 1H, BCH_2), 1.35 (d, *J* = 16.0 Hz, 1H, BCH_2), 1.09 (s, 6H, CH_3 x 2), 1.03 (s, 6H, CH_3 x 2).

^{13}C NMR (101 MHz, $CDCl_3$) δ ppm 163.0 (*C*ON), 147.3 (Ar*C*), 141.5 (Ar*C*), 136.5 (Ar*C*), 133.1 (Ar*C*H), 129.3 (Ar*C*H x 2), 128.4 (Ar*C*H), 125.9 (Ar*C*H x 2), 118.9 (Ar*C*H), 117.3 (Ar*C*), 114.7 (Ar*C*H), 83.5 (*C*q x 2), 75.5 (NH*C*H), 50.0 (N*C*H_2), 41.8 (*C*q), 38.1 (NCH_2CH_2), 24.7 (*C*H_3 x 2), 24.6 (*C*H_3 x 2), 21.0 (*C*H_3). The signal for BCH_2 was not observed.

^{11}B NMR (128 MHz, $CDCl_3$) δ ppm 32.8.

IR ν_{max} (neat/cm^{-1}): 3363, 2976, 1637, 1612, 1485, 1364, 1324, 1143, 971, 849, 754.

Mp: 142 °C.

HRMS : calculated for $C_{25}H_{31}O_3N_2BNa$ $[M + Na]^+$ 441.2314, found 441.2320.

Specific rotation: $[\alpha]_D^{22}$ + 105.5 (c 0.65, $CHCl_3$).

Enantiomeric purity of **2d** was determined by HPLC analysis in comparison with authentic racemic material (er = 96:4; **IA** column, 90:10 hexanes: *i*PrOH, 0.5 mL/min, 20 °C, 254 nm).

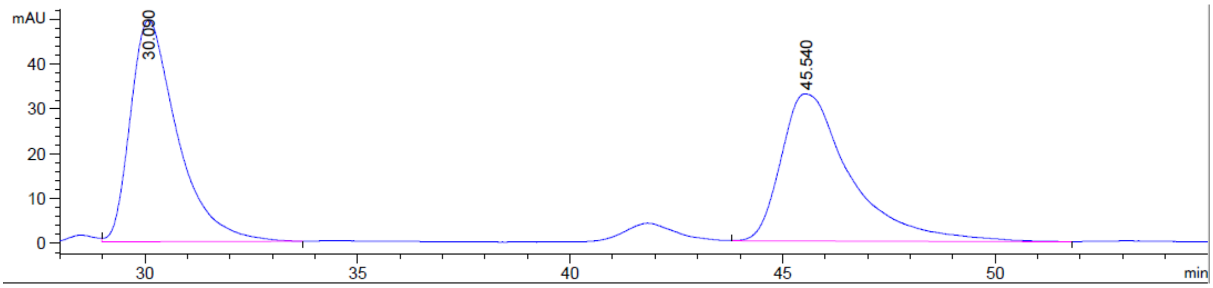

Peak #	RetTime [min]	Type	Width [min]	Area [mAU*s]	Height [mAU]	Area %
1	30.090	VB	1.1245	3780.98682	49.42635	50.4384
2	45.540	BB	1.4718	3715.25439	32.79236	49.5616

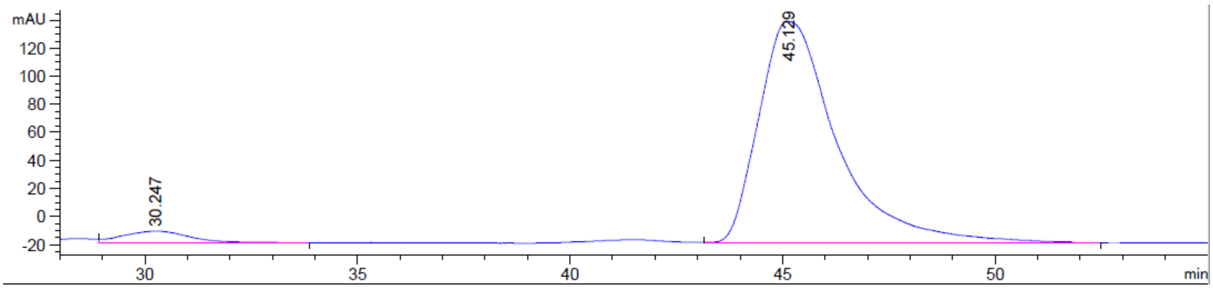

Peak #	RetTime [min]	Type	Width [min]	Area [mAU*s]	Height [mAU]	Area %
1	30.247	VB	1.3301	927.25250	8.28294	4.3832
2	45.129	VB	1.7210	2.02273e4	158.06352	95.6168

^{1}H NMR of 2d (400 MHz, $CDCl_3$)

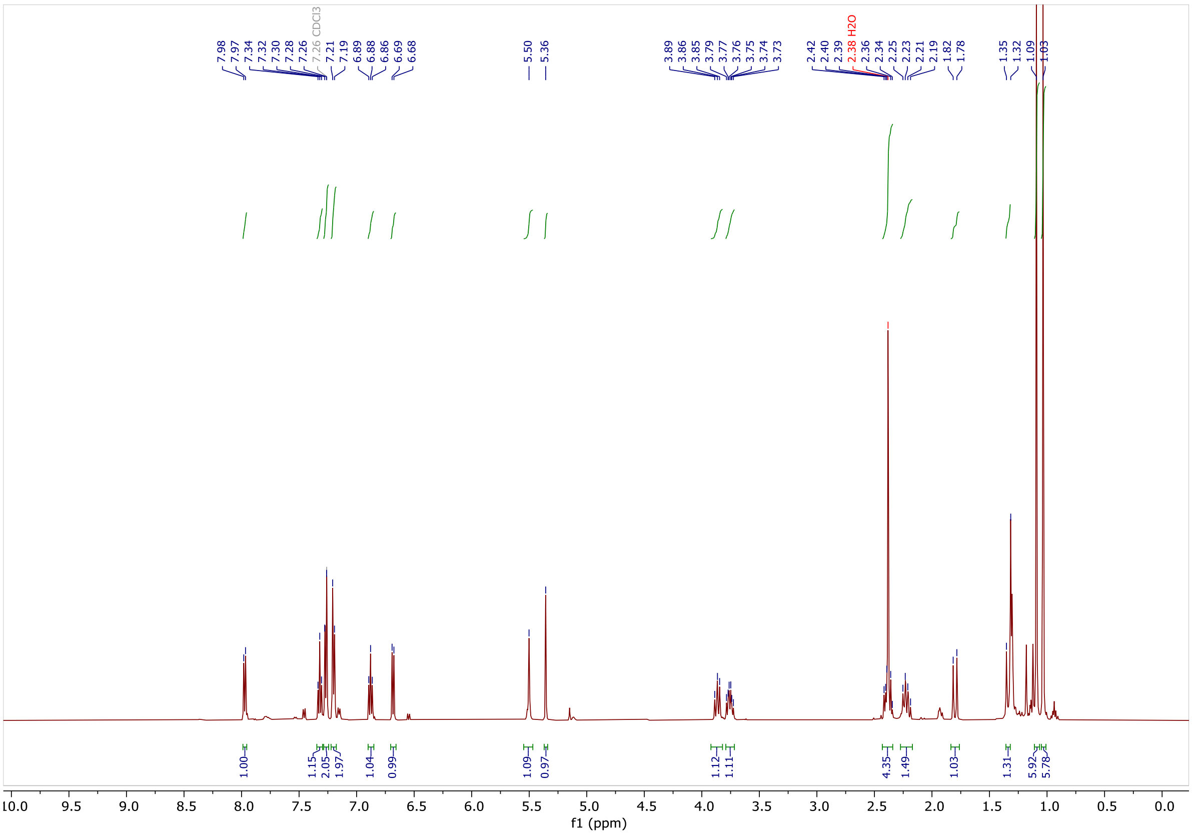

^{13}C NMR of 2d (101 MHz, $CDCl_3$)

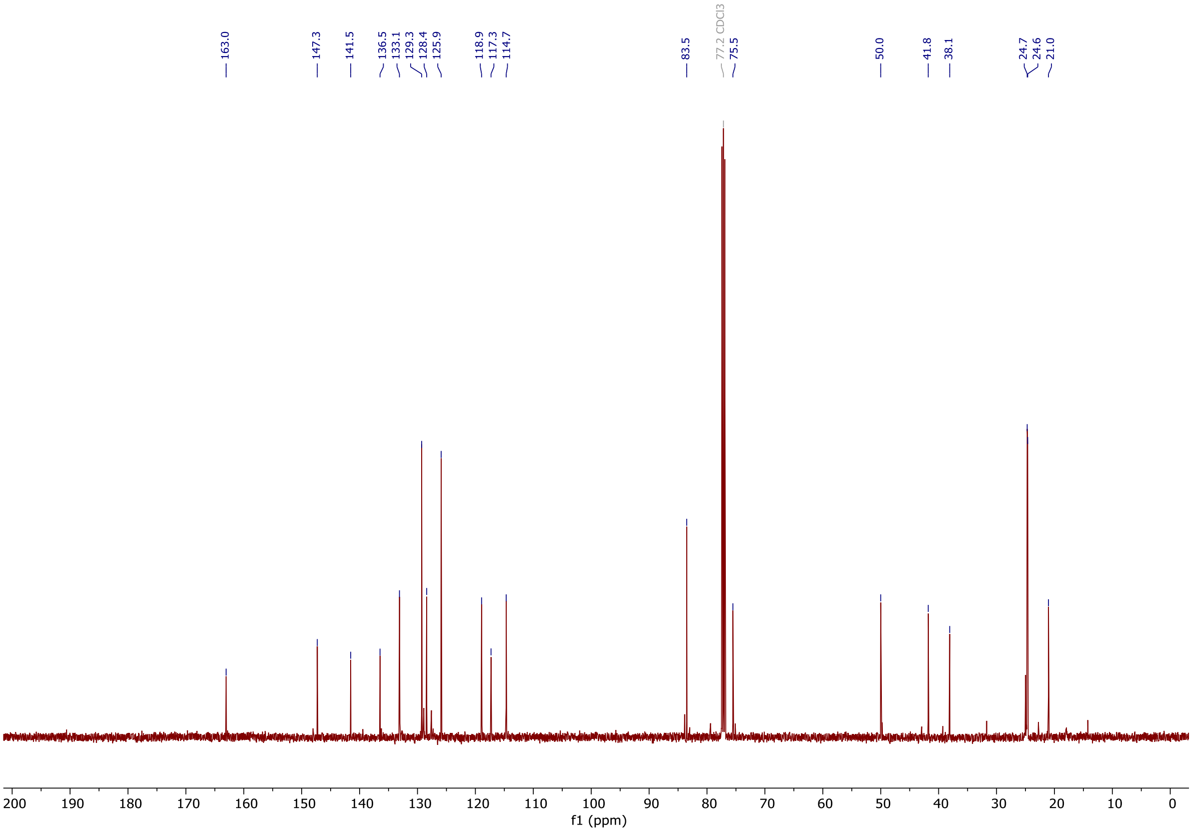

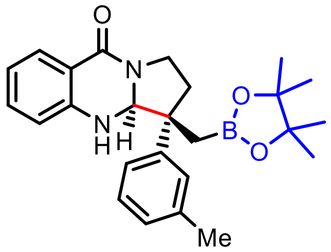

Chemical Formula: $C_{25}H_{31}BN_2O_3$
Molecular Weight: 418.34

(3*S*,3a*S*)-3-(3-Methylphenyl)-3-((4,4,5,5-tetramethyl-1,3,2-dioxaborolan-2-yl)methyl)-2,3,3a,4-tetrahydropyrrolo[2,1-*b*]quinazolin-9(1*H*)-one (2e)

Compound **2e** was prepared according to General Procedure C, on 0.2 mmmol scale and running the reaction at 0 °C. The title compound was isolated by column chromatography (hexane: ethyl acetate, 80:20 - 50:50) as a white amorphous solid (50 mg, 0.12 mmol, 60%, dr = 98:2).

^1H NMR (400 MHz, $CDCl_3$) δ ppm 7.92 (dd, *J* = 7.8, 1.5 Hz, 1H, ArCH), 7.35 – 7.18 (m, 2H, ArCH + ArCH), 7.15 – 7.08 (m, 2H, ArCH + ArCH), 7.05 (d, *J* = 7.5 Hz, 1H, ArCH), 6.83 (td, *J* = 7.9, 1.0 Hz, 1H, ArCH), 6.64 (dd, *J* = 8.1, 1.0 Hz, 1H, ArCH), 5.51 (s, 1H, NH), 5.33 (d, *J* = 1.1 Hz, 1H,

NHCH), 3.82 (ddd, J = 11.1, 9.3, 1.5 Hz, 1H, NCH_2), 3.70 (*app.* td, J = 11.4, 7.2 Hz, 1H, NCH_2), 2.35 (s, 3H, $ArCH_3$), 2.39 – 2.29 (m, 1H, NCH_2CH_2), 2.18 (ddd, J = 12.8, 10.8, 9.3 Hz, 1H, NCH_2CH_2), 1.76 (d, J = 15.9 Hz, 1H, BCH_2), 1.29 (d, J = 16.0 Hz, 1H, BCH_2), 1.03 (s, 6H, CH_3 x 2), 0.97 (s, 6H, CH_3 x 2).

^{13}C NMR (101 MHz, $CDCl_3$) δ ppm 164.5 (CON), 147.3 (ArC), 144.6 (ArC), 138.2 (ArC), 133.1 (ArCH), 128.6 (ArCH), 128.5 (ArCH), 127.6 (ArCH), 126.8 (ArCH), 123.1 (ArCH), 118.9 (ArCH), 117.3 (ArC), 114.7 (ArCH), 83.5 (Cq x 2), 75.3 (NHCH), 50.3 (NCH_2), 41.8 (Cq), 38.3 (NCH_2CH_2), 24.8 (CH_3 x 2), 24.7 (CH_3 x 2), 21.8 ($ArCH_3$). The signal for BCH_2 was not observed.

^{11}B NMR (128 MHz, $CDCl_3$) δ ppm 32.67

IR ν_{max} (neat/cm^{-1}): 3374, 2976, 1638, 1611, 1485, 1432, 1389, 1251, 1144, 968, 849, 754.

HRMS: calculated for $C_{25}H_{31}N_2O_3BNa$ $[M + Na]^+$ 441.2320, found 441.2305

Specific rotation: $[\alpha]_D^{23}$ + 114.77 (c 0.665, $CHCl_3$).

Enantiomeric purity of **2e** was determined by HPLC analysis in comparison with authentic racemic material (er = 97:3; **IA** column, 90:10 hexanes: *i*PrOH, 0.5 mL/min, 20 °C, 254 nm)

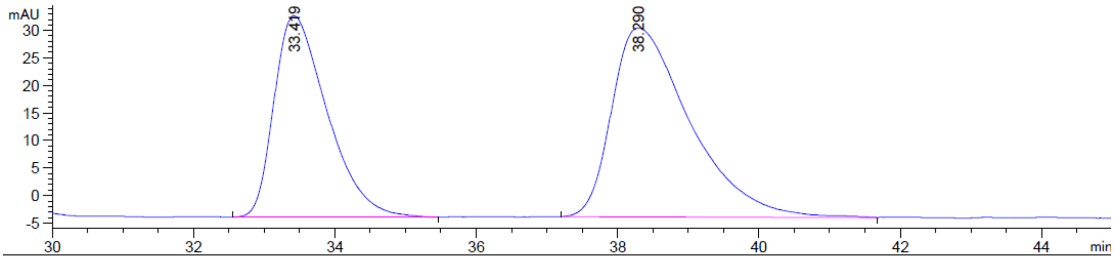

Peak #	RetTime [min]	Type	Width [min]	Area [mAU*s]	Height [mAU]	Area %
1	33.419	BB	0.7760	1914.01978	36.60474	42.0434
2	38.290	BB	1.0519	2638.46851	34.36064	57.9566

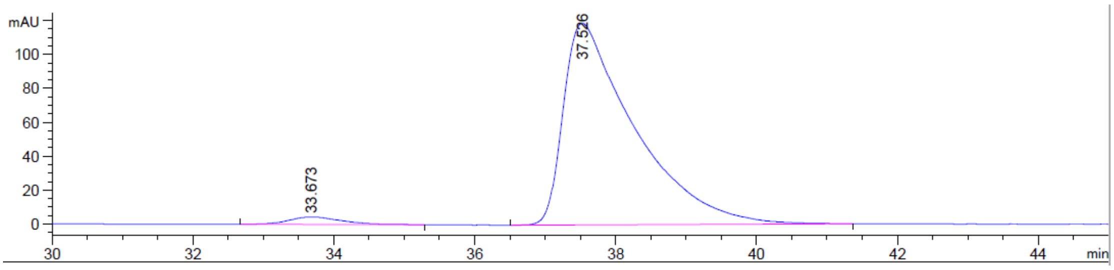

Peak #	RetTime [min]	Type	Width [min]	Area [mAU*s]	Height [mAU]	Area %
1	33.686	BB	0.7536	1415.20691	27.44531	2.5864
2	37.527	BB	0.9883	5.33025e4	752.54834	97.4136

^{1}H NMR of 2e (400 MHz, $CDCl_3$)

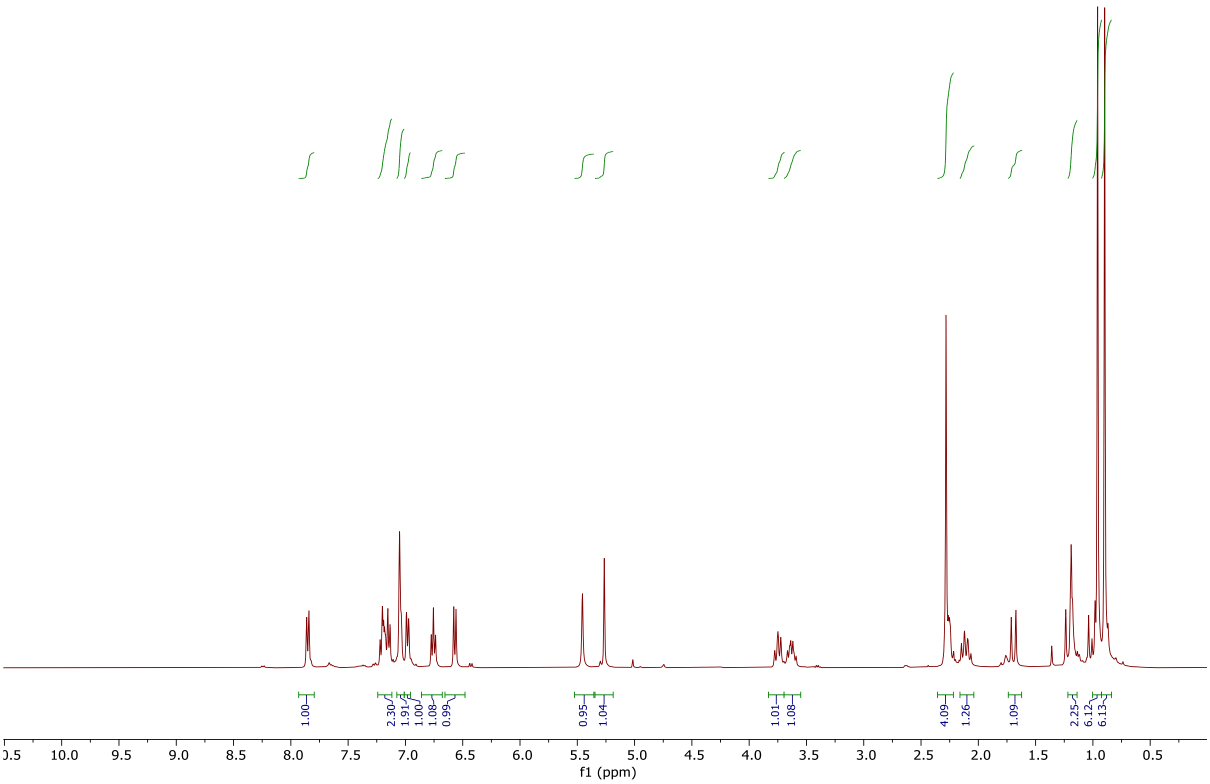

^{13}C NMR of 2e (101 MHz, $CDCl_3$)

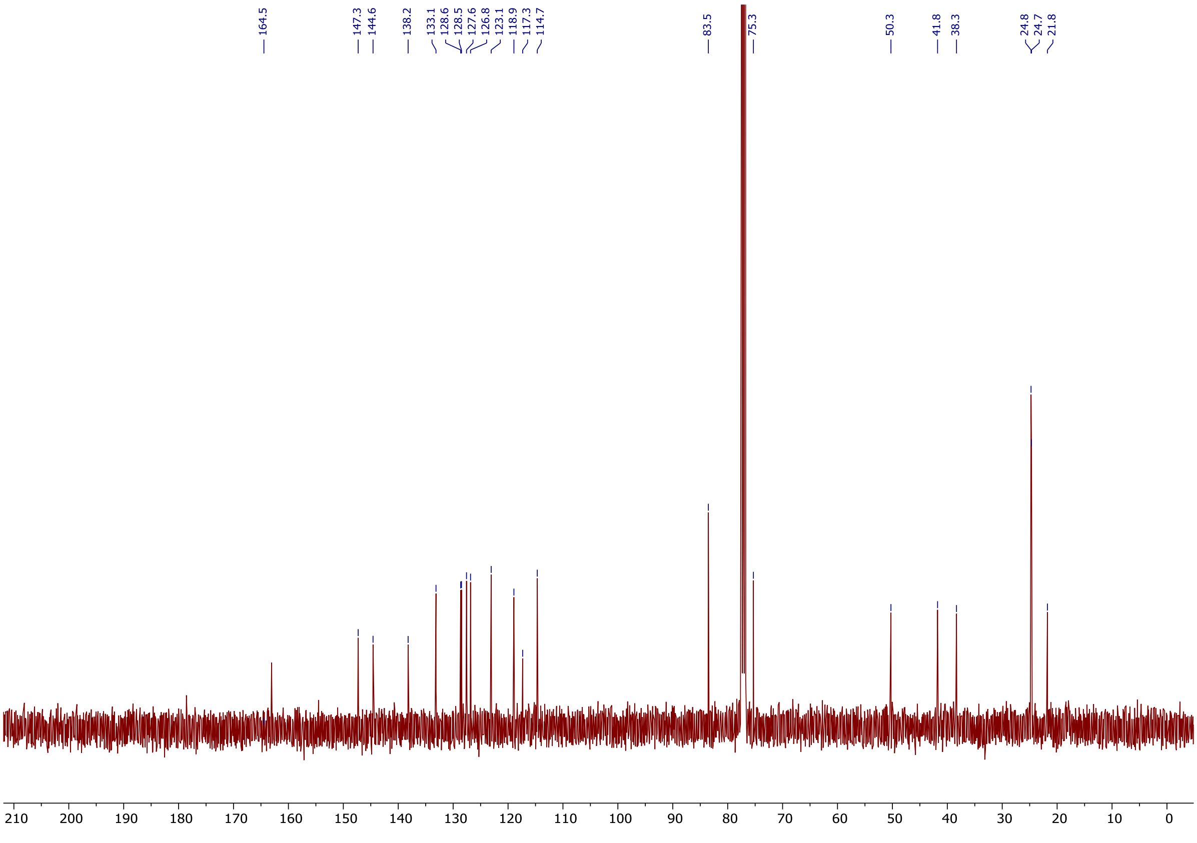

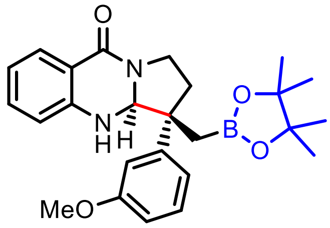

Chemical Formula: $C_{25}H_{31}BN_2O_4$
Molecular Weight: 434.3430

(3*S*,3a*S*)-3-(3-Methoxyphenyl)-3-((4,4,5,5-tetramethyl-1,3,2-dioxaborolan-2-yl)methyl)-2,3,3a,4-tetrahydropyrrolo[2,1-b]quinazolin-9(1H)-one (2f)

Compound **2f** was prepared according to General Procedure C, on a 0.183 mmol scale and running the reaction at 0 °C. The title compound was isolated by column chromatography (hexane: diethyl ether, 25:75 to 0:100) as a white solid (41 mg, 0.094 mmol, 51%, dr = 94:6).

^{1}H NMR (400 MHz, $CDCl_3$) δ ppm 7.94 (dd, *J* = 7.8, 1.6 Hz, 1H, ArCH), 7.33 – 7.24 (m, 2H, ArCH + ArCH), 6.94 (dd, *J* = 7.4, 1.7 Hz, 1H, ArCH), 6.90 (t, *J* = 2.2 Hz, 1H, ArCH), 6.85 (td, *J* = 7.6, 1.1 Hz, 1H, ArCH), 6.80 (dd, *J* = 8.3, 2.5 Hz, 1H, ArCH), 6.65 (dd, *J* = 8.1, 1.0 Hz, 1H, ArCH), 5.49 (s, 1H, NH), 5.34 (d, *J* = 1.2 Hz, 1H, NHC*H*), 3.83 (s, 3H, OCH_3), 3.90 – 3.63 (m, 2H, NCH_2), 2.35 (ddd, *J* = 12.9, 7.2, 1.6 Hz, 1H, NCH_2CH_2), 2.27 – 2.13 (m, 1H, NCH_2CH_2), 1.78 (d, *J* = 16.1 Hz, 1H, CH_2Bpin), 1.31 (d, *J* = 16.2 Hz, 1H, CH_2Bpin), 1.07 (s, 6H, CH_3 x 2), 1.00 (s, 6H, CH_3 x 2).

^{13}C NMR (101 MHz, $CDCl_3$) δ ppm 163.0 (CON), 159.9 (ArC), 147.2 (ArC), 146.3 (ArC), 133.1 (ArCH), 129.7 (ArCH), 128.4 (ArCH), 119.0 (ArCH), 118.4 (ArCH), 117.3 (ArC), 114.7 (ArCH), 112.8 (ArCH), 111.5 (ArCH), 83.5 (Cq x 2), 75.3 (NCH), 55.4 (OCH_3), 50.4 (Cq), 41.7 (NCH_2), 38.1 (NCH_2CH_2), 24.7 (*C*H_3 x 2), 24.6 (*C*H_3 x 2).

^{11}B NMR (128 MHz, $CDCl_3$) δ 33.62

IR ν_{max} (neat/cm^{-1}): 2950, 2921, 2852, 1672, 1609, 1458, 1376, 1322, 1260, 773, 724, 696.

Mp: 154-5 °C

HRMS : calculated for $C_{25}H_{32}O_4N_2B$ $[M + H]^+$ 435.2450, found 435.2445.

Specific rotation: $[\alpha]_D^{23}$ + 85.5 (c 2.68, $CHCl_3$).

Enantiomeric purity of **2f** was determined by HPLC analysis in comparison with authentic racemic material (er = 94:6; **IA** column, 95:5 hexanes: *i*PrOH, 0.5 mL/min, 20 °C, 254 nm).

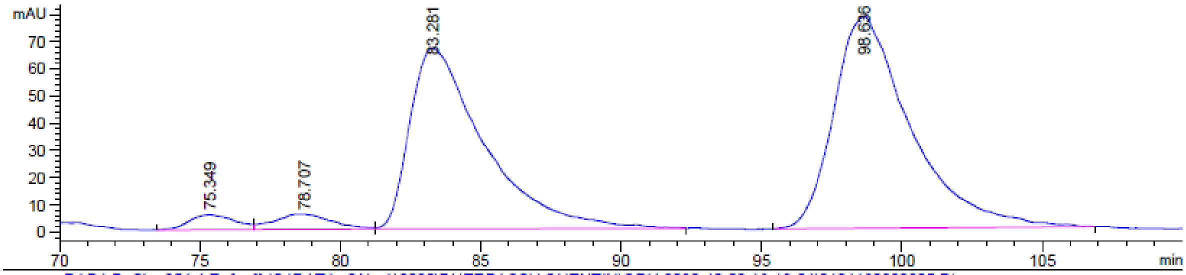

Peak #	RetTime [min]	Type	Width [min]	Area [mAU*s]	Height [mAU]	Area %
1	75.349	BV	1.4156	617.94238	5.53028	2.1609
2	78.707	VV	1.7105	806.16541	5.60327	2.8192
3	83.281	VB	2.4915	1.22743e4	66.68161	42.9231
4	98.636	BB	2.6160	1.48976e4	78.14946	52.0968

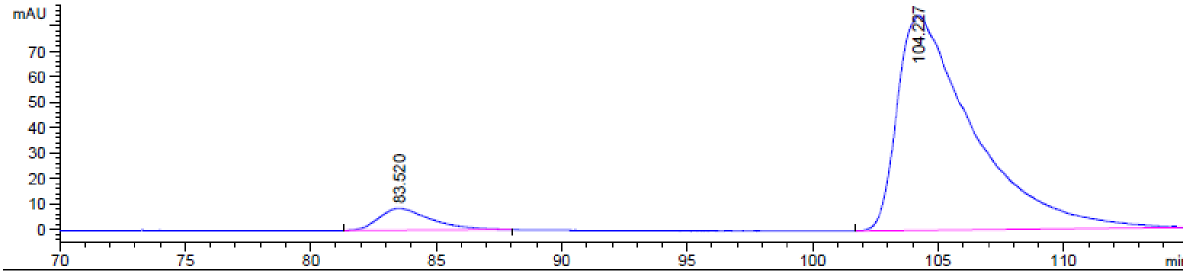

Peak #	RetTime [min]	Type	Width [min]	Area [mAU*s]	Height [mAU]	Area %
1	83.520	BB	1.6669	1221.76001	8.60281	6.4541
2	104.227	BB	2.5490	1.77082e4	84.36022	93.5459

^{1}H NMR of 2f (400 MHz, $CDCl_3$)

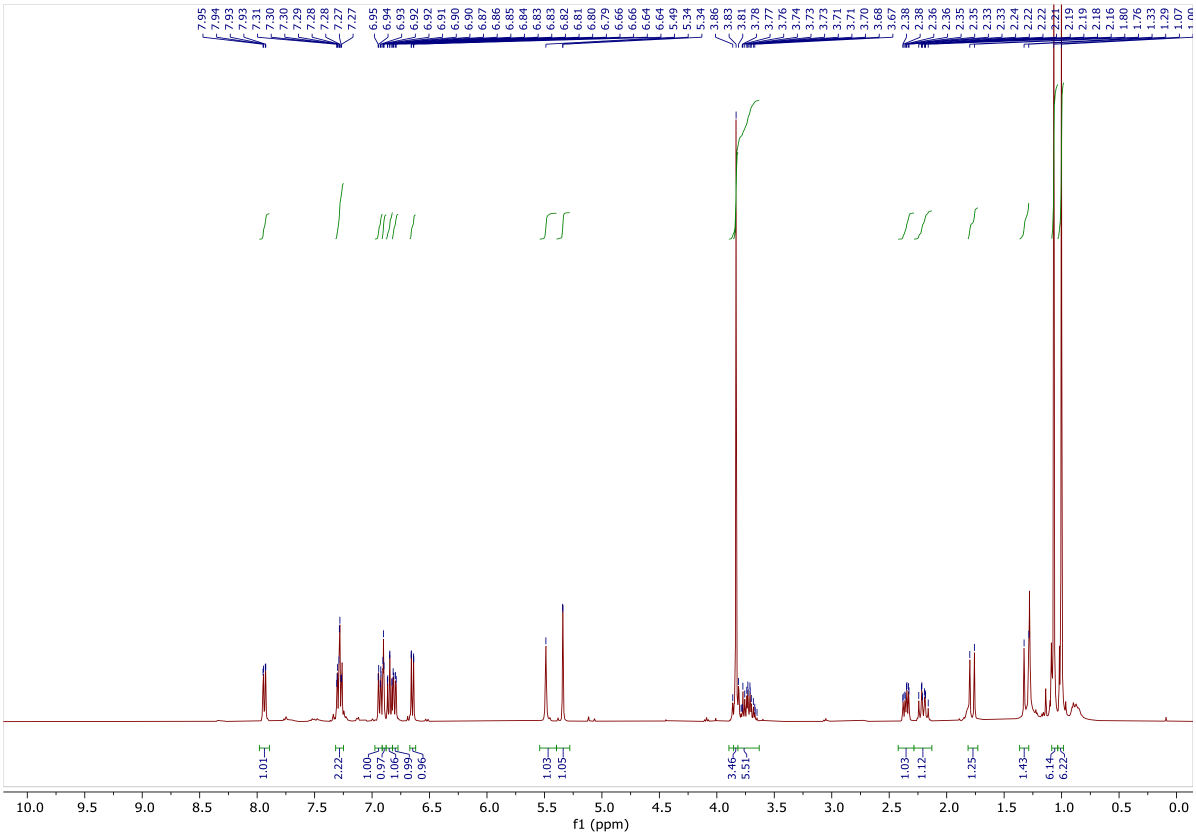

^{13}C NMR 2f (101 MHz, $CDCl_3$)

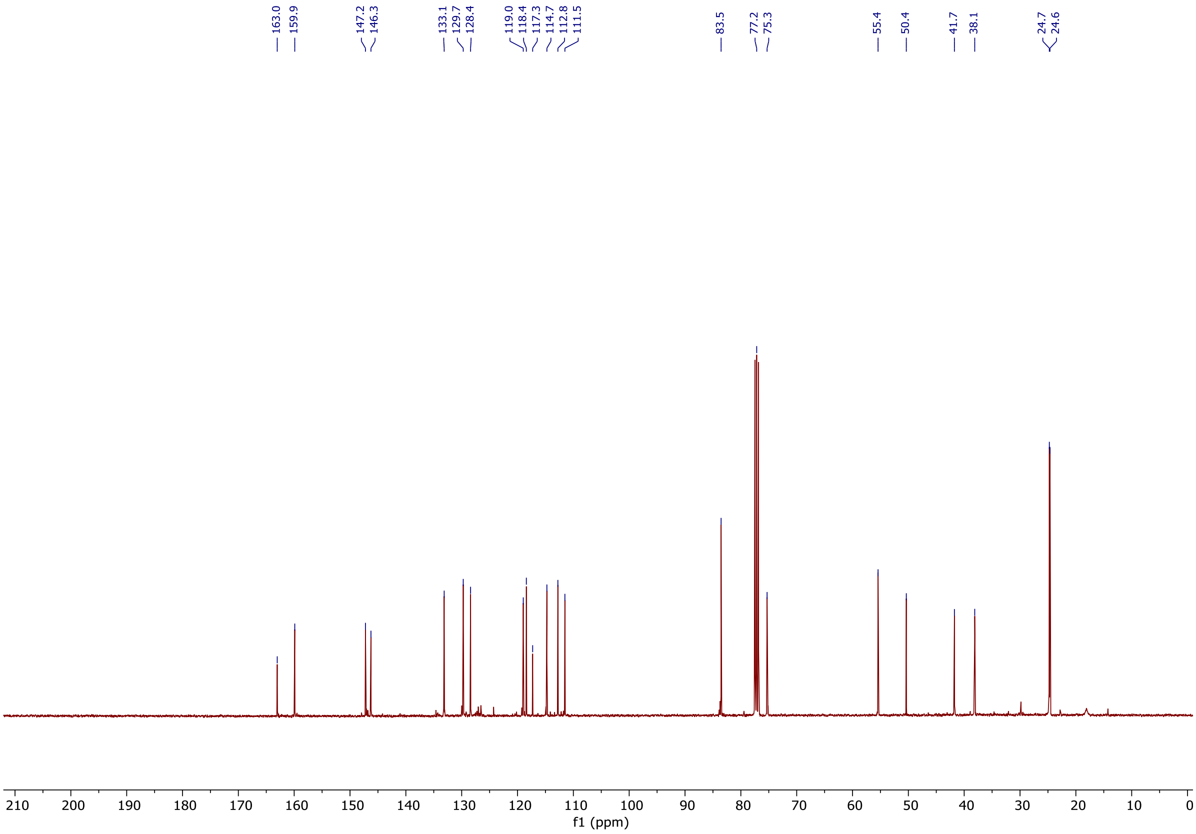

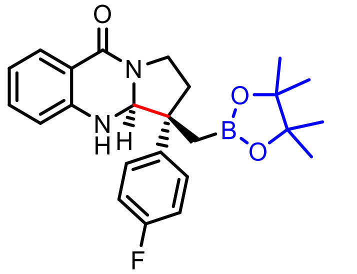

Chemical Formula: $C_{24}H_{28}BFN_2O_3$
Molecular Weight: 422.31

(3*S*,3a*S*)-3-(4-Fluorophenyl)-3-((4,4,5,5-tetramethyl-1,3,2-dioxaborolan-2-yl)methyl)-2,3,3a,4-tetrahydropyrrolo[2,1-b]quinazolin-9(1H)-one (2g)

Compound **2g** was prepared according to General Procedure C, on a 0.255 mmol scale and running the reaction at 25 °C. The title compound was isolated by column chromatography (hexane: diethyl ether, 25:75 to 0:100) as a white solid (51.5 mg, 0.128 mmol, 61%, dr = 85:15).

^{1}H NMR (400 MHz, $CDCl_3$) δ ppm 7.92 (dd, *J* = 7.8, 1.6 Hz, 1H, ArC^{1}*H*), 7.33 – 7.24 (m, 3H, ArC*H* x 2 + ArC*H*), 7.06 (tt, *J* = 8.6, 3.2 Hz, 2H, ArC*H*), 6.84 (td, *J* = 7.5, 1.0 Hz, 1H, ArC*H*), 6.64 (dd, *J* = 8.1, 1.0 Hz, 1H, ArC*H*), 5.47 (br, 1H, N*H*), 5.29 (d, *J* = 1.4 Hz, 1H, NC*H*), 3.81 (ddd, *J* = 11.0,

9.3, 1.5 Hz, 1H, NC*H*$_2$), 3.69 (ddd, *J* = 12.0, 10.8, 7.2 Hz, 1H, NC*H*$_2$), 2.32 (ddd, *J* = 12.9, 7.2, 1.5 Hz, 1H, NCH$_2$C*H*$_2$), 2.14 (ddd, *J* = 12.8, 10.8, 9.3 Hz, 1H, NCH$_2$C*H*$_2$), 1.76 (d, *J* = 16.1 Hz, 1H, C*H*$_2$Bpin), 1.28 (d, *J* = 16.1 Hz, 1H, C*H*$_2$Bpin), 1.05 (s, 6H, C*H*$_3$ x 2), 0.98 (s, 6H, C*H*$_3$ x 2).

^{13}C NMR (101 MHz, CDCl$_3$) δ ppm 163.0 (*C*ON), 161.7 (d, $^{1}J_{C\text{-}F}$ = 245.9 Hz, Ar*C*), 147.1 (Ar*C*), 140.5 (d, *J* = 3.4 Hz, Ar*C*), 133.2 (Ar*C*H), 128.5 (Ar*C*H), 127.7 (d, $^{3}J_{C\text{-}F}$ = 7.8 Hz, Ar*C*H x 2), 119.2 (Ar*C*H), 117.4 (Ar*C*), 115.5 (d, $^{2}J_{C\text{-}F}$ = 21.3 Hz, Ar*C*H x 2), 114.9 (Ar*C*H), 83.7 (*C*q x 2), 75.3 (N*C*H), 49.9 (*C*q), 41.7 (N*C*H$_2$), 38.4 (NCH$_2$*C*H$_2$), 24.8 (*C*H$_3$ x 2), 24.6 (*C*H$_3$ x 2). The signal for B*C*H$_2$ was not observed.

^{11}B NMR (128 MHz, CDCl$_3$) δ 33.64

^{19}F NMR (376 MHz, CDCl$_3$) δ - -114.1 (tt, *J* = 8.5, 5.4 Hz, 1F).

IR ν_{max} (neat/cm^{-1}): 2922, 2852, 1627, 1612, 1475, 1365, 1141, 863, 751.

HRMS : calculated for $C_{24}H_{27}O_3N_2BF$ [M + H]$^+$ 421.2104, found 421.2115.

Specific rotation: $[\alpha]_D^{20}$ + 69.9 (c 2.44, CHCl$_3$).

Enantiomeric purity of **2g** was determined by HPLC analysis in comparison with authentic racemic material (er = 89:11; **IA** column, 90:10 hexanes: *i*PrOH, 0.5 mL/min, 20 °C, 254 nm).

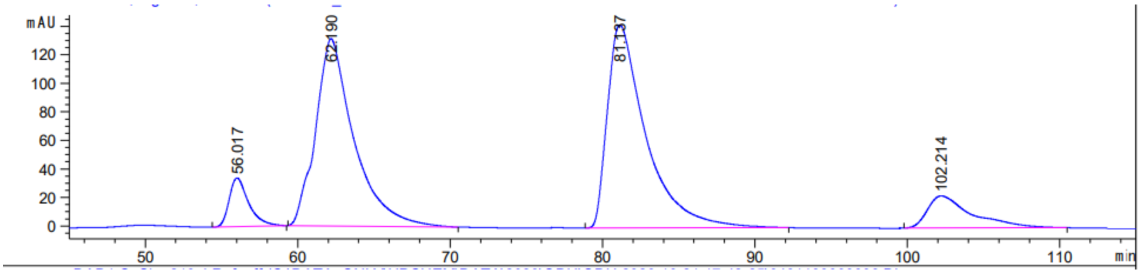

Peak #	RetTime [min]	Type	Width [min]	Area [mAU*s]	Height [mAU]	Area %
1	56.017	BB	1.2594	3027.81494	34.18322	5.4566
2	62.190	BB	2.2250	2.29327e4	131.21855	41.3280
3	81.137	BB	2.2947	2.49807e4	142.25414	45.0188
4	102.214	BB	2.3670	4548.25244	22.49382	8.1966

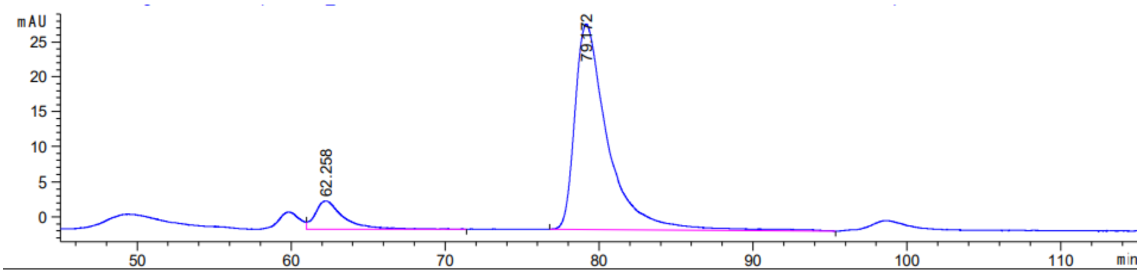

Peak #	RetTime [min]	Type	Width [min]	Area [mAU*s]	Height [mAU]	Area %
1	62.258	VV	1.9240	534.30524	4.04782	10.8320
2	79.172	BB	2.1950	4398.33740	29.37237	89.1680

^{1}H NMR of 2g (400 MHz, $CDCl_3$)

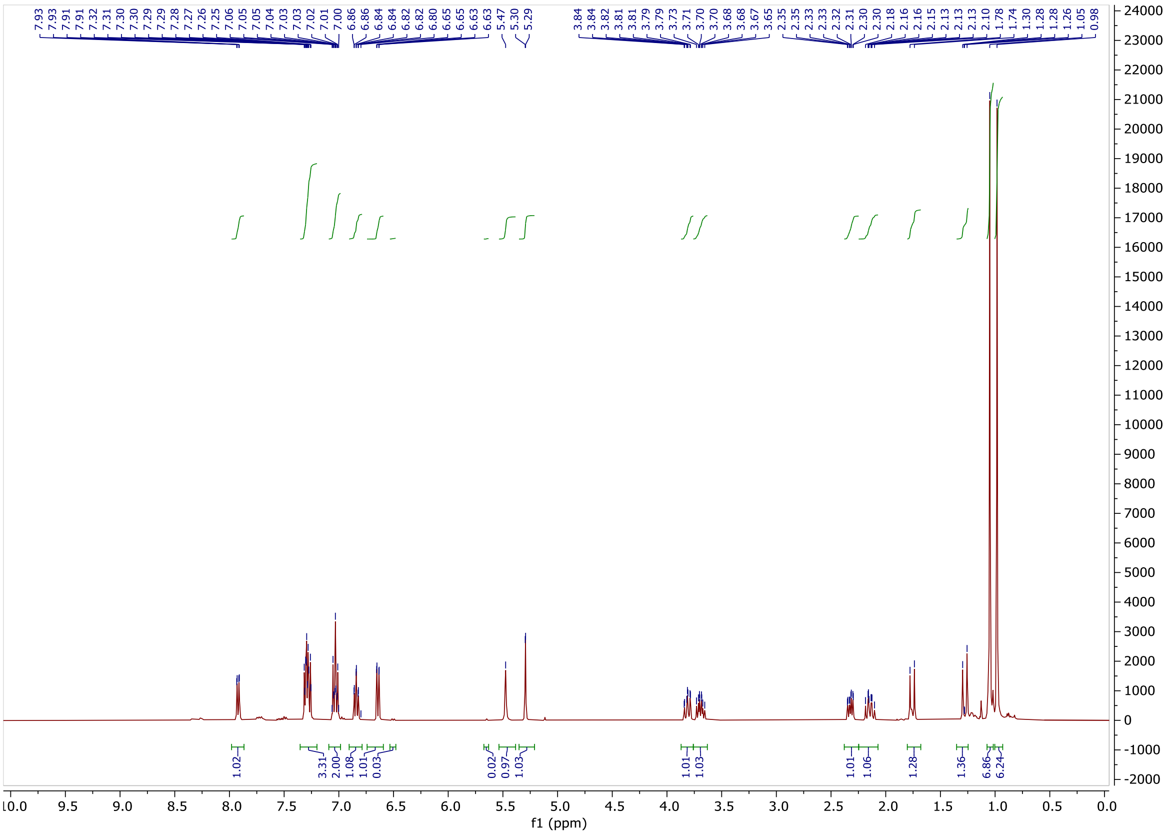

^{13}C NMR of 2g (101 MHz, $CDCl_3$)

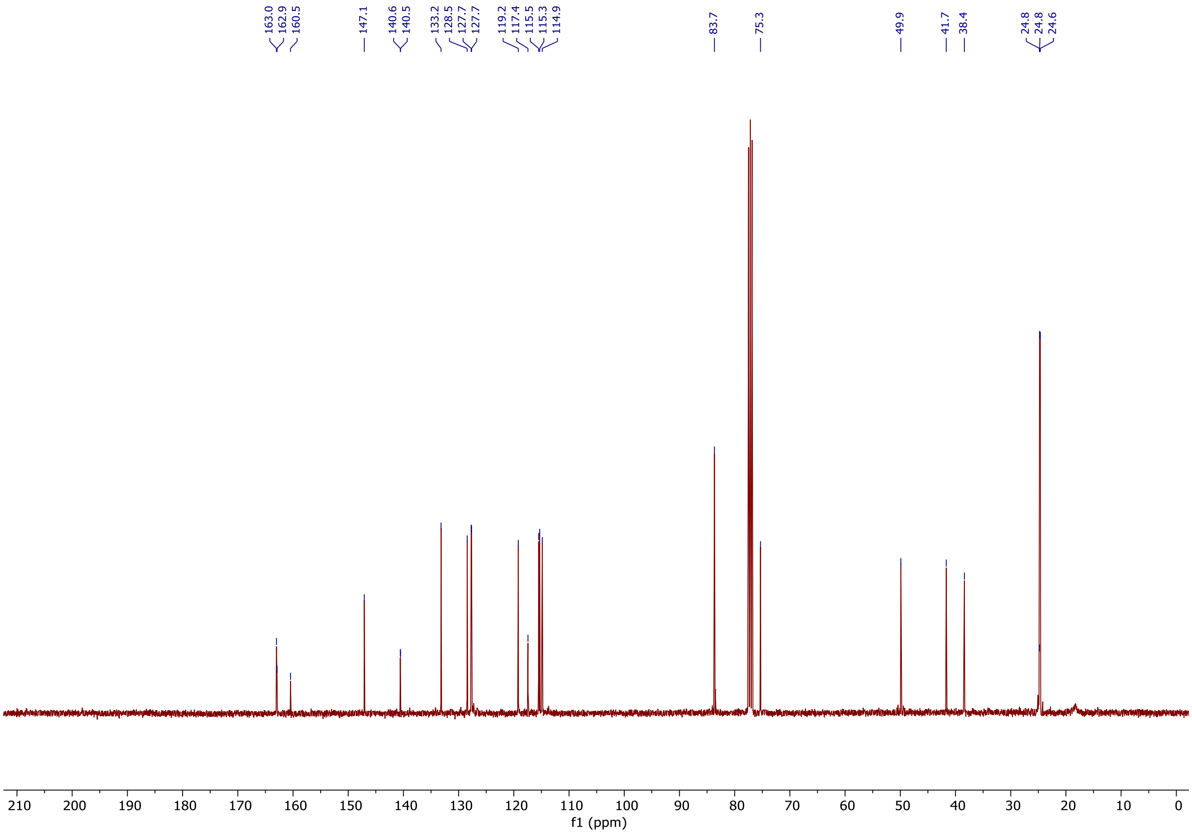

^{19}F NMR of 2g (376 MHz, $CDCl_3$)

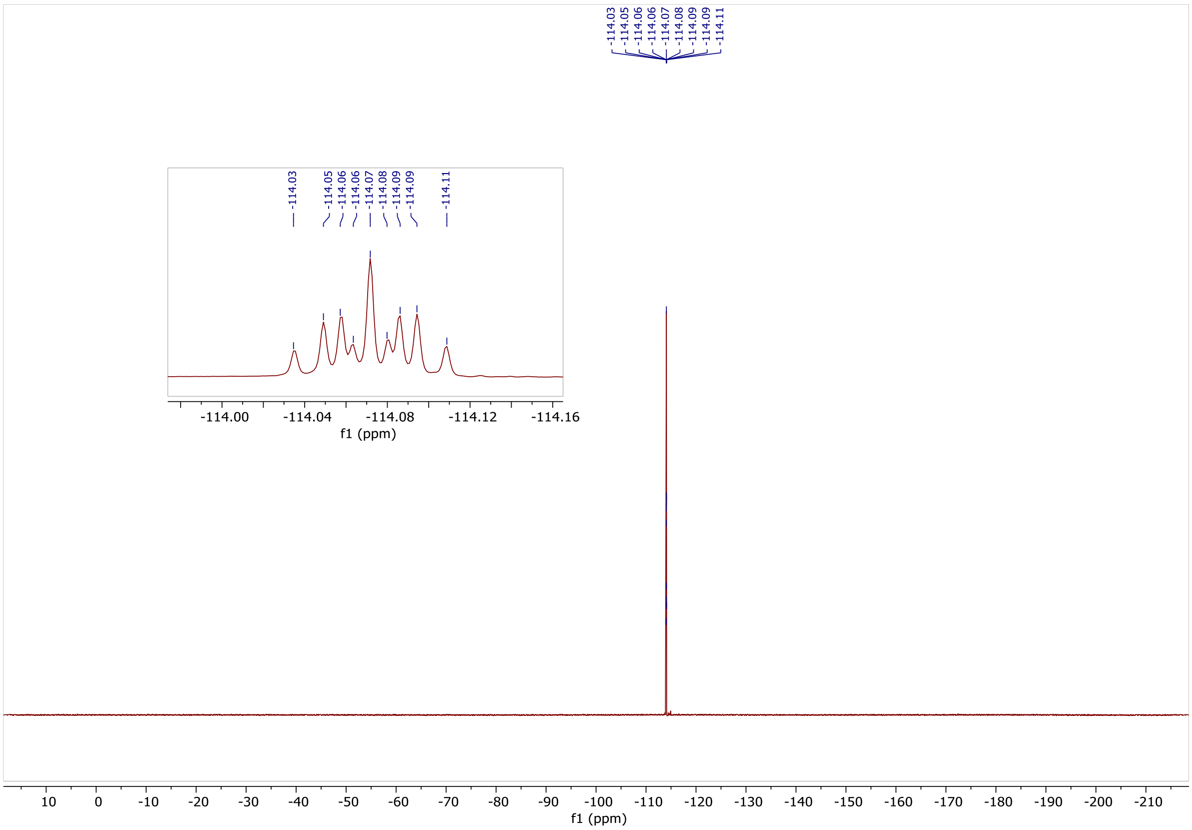

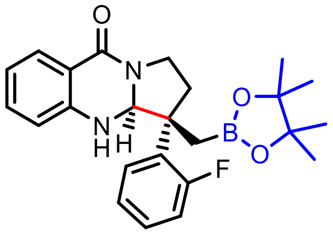

Chemical Formula: $C_{24}H_{28}BFN_2O_3$
Molecular Weight: 422.31

(3*S*,3a*S*)-3-(4-Fluorophenyl)-3-((4,4,5,5-tetramethyl-1,3,2-dioxaborolan-2-yl)methyl)-2,3,3a,4-tetrahydropyrrolo[2,1-*b*]quinazolin-9(1*H*)-one (2h)

Compound **2h** was prepared according to General Procedure C, running the reaction at 25 °C. The title compound was isolated by column chromatography (hexane: diethyl ether, 25:75 - 0:100) as a white amorphous solid (69 mg, 0.0164 mmol, 64%, dr = 95:5).

^{1}H NMR (400 MHz, $CDCl_3$) δ ppm 7.92 (dd, *J* = 7.8, 1.6 Hz, 1H, ArC*H*), 7.36 – 7.20 (m, 3H, ArC*H* + 2 x ArC*H*), 7.12 (dd, *J* = 7.6, 1.4 Hz, 1H, ArC*H*), 7.10 – 7.00 (m, 1H, ArC*H*), 6.84 (ddd, *J* = 8.2, 7.3, 1.0 Hz, 1H, ArC*H*), 6.66 (dd, *J* = 8.1, 1.0 Hz, 1H, ArC*H*), 5.77 (*br.* s, 1H, N*H*), 5.38 (*br.* s, 1H, NHC*H*), 3.83 (ddd, *J* = 12.0, 9.5, 1.4 Hz, 1H, NC*H*$_2$), 3.70 (ddd, *J* = 12.0, 11.0, 7.2 Hz, 1H, NC*H*$_2$),

2.67 – 2.53 (m, 1H, NCH_2CH_2), 2.17 (ddd, *J* = 12.9, 11.0, 9.5 Hz, 1H, NCH_2CH_2), 1.75 (dd, *J* = 16.0, 1.5 Hz, 1H, BCH_2), 1.47 (dd, *J* = 15.9, 1.9 Hz, 1H, BCH_2), 1.04 (s, 6H, 2 x CH_3), 0.94 (s, 6H, 2 x CH_3).

^{13}C NMR (101 MHz, $CDCl_3$) δ ppm 163.1 (*C*ON), 161.1 (d, $^1J_{C\text{-}F}$ = 246.4 Hz, Ar*C*), 147.3 (Ar*C*), 133.2 (Ar*C*H), 131.7 (d, $^2J_{C\text{-}F}$ = 13.4 Hz, Ar*C*), 128.8 (d, $^3J_{C\text{-}F}$ = 8.9 Hz, Ar*C*H), 128.4 (Ar*C*H), 128.3 (d, $^3J_{C\text{-}F}$ = 5.5 Hz, Ar*C*H), 124.2 (d, $^4J_{C\text{-}F}$ = 3.2 Hz, Ar*C*H), 119.0 (Ar*C*H), 117.2 (Ar*C*), 116.5 (d, $^2J_{C\text{-}F}$ = 24.0 Hz, Ar*C*H), 114.6 (Ar*C*H), 83.5 (*C*q x 2), 74.9 (NH*C*H), 49.2 (N*C*H_2), 41.5 (*C*q), 36.9 (NCH_2CH_2), 24.8 (*C*H_3 x 2), 24.6 (*C*H_3 x 2). The signal for BCH_2 was not observed.

^{11}B NMR (128 MHz, $CDCl_3$) δ ppm 33.7.

^{19}F NMR (376 MHz, $CDCl_3$) δ ppm -109.43 (br, 1F, minor), -111.35 (br, 1F, major).

IR ν_{max} (neat/cm^{-1}): 3378, 2977, 1643, 1612, 1486, 1430, 1364, 1214, 1143, 971, 848, 754.

HRMS: calculated for $C_{24}H_{29}N_2O_3BF$ $[M + H]^+$ 423.2254, found 423.2246

Specific rotation: $[\alpha]_D^{23}$ + 113.75 (c 1.14, $CHCl_3$).

Enantiomeric purity of **2h** was determined by HPLC analysis in comparison with authentic racemic material (er = 97:3; **IA** column, 95:5 hexanes: *i*PrOH, 0.5 mL/min, 20 °C, 254 nm).

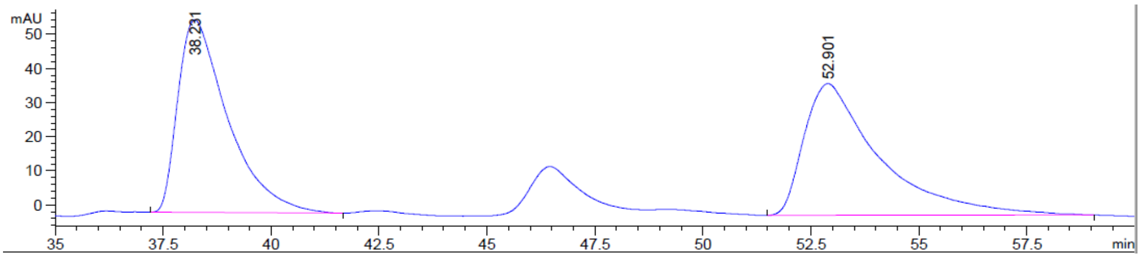

Peak #	RetTime [min]	Type	Width [min]	Area [mAU*s]	Height [mAU]	Area %
1	38.231	BB	1.1555	4525.78223	56.32325	49.1828
2	52.901	BB	1.6612	4676.18311	38.54922	50.8172

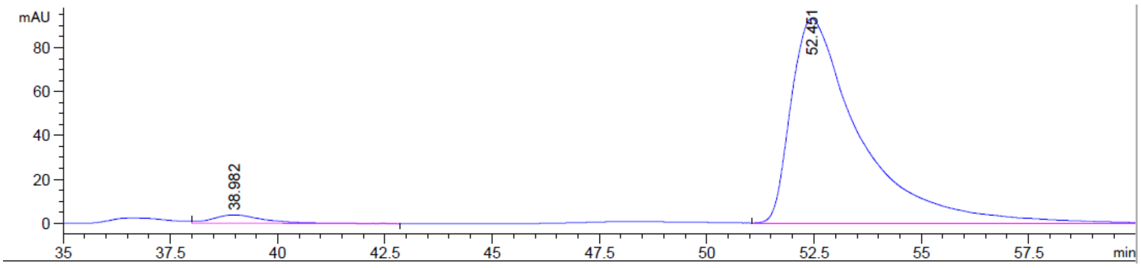

Peak #	RetTime [min]	Type	Width [min]	Area [mAU*s]	Height [mAU]	Area %
1	38.982	VB	1.2365	321.42728	3.82456	2.9590
2	52.451	VB	1.6607	1.05413e4	93.40483	97.0410

^{1}H NMR of 2h (400 MHz, $CDCl_3$)

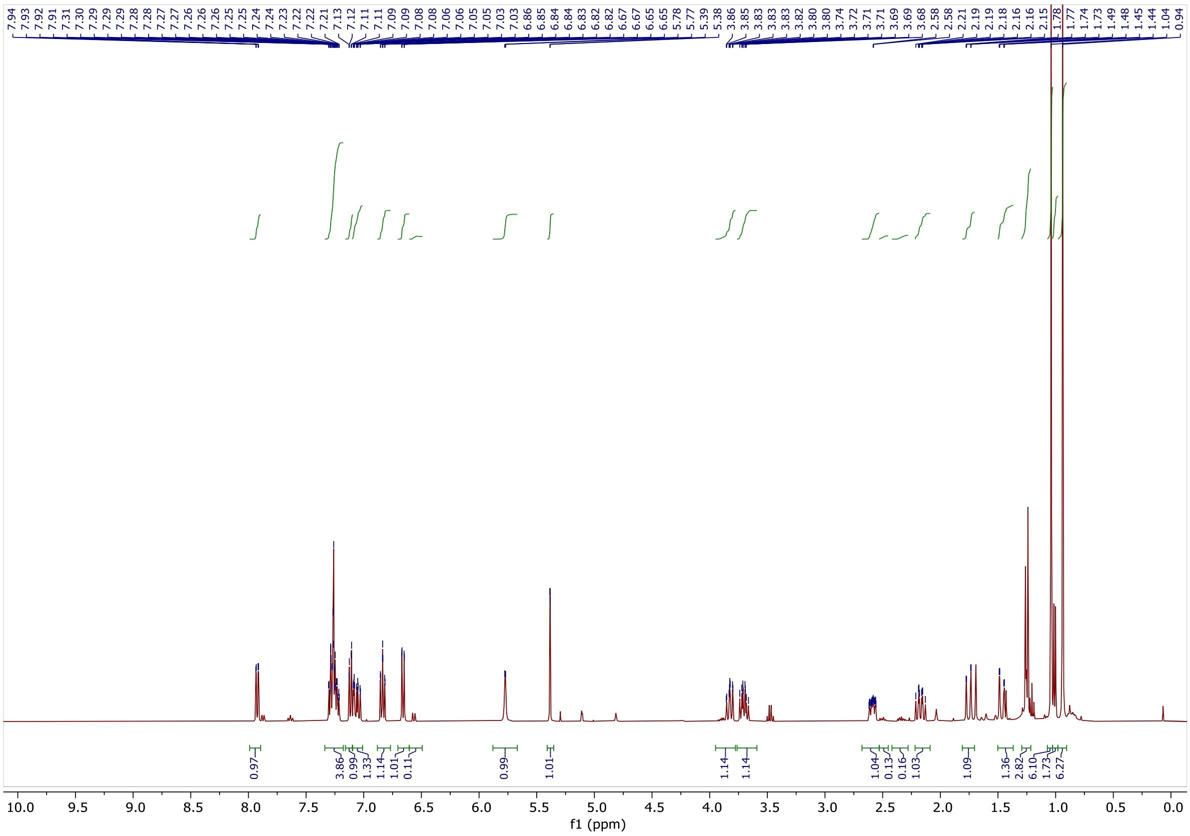

^{13}C NMR of 2h (101 MHz, $CDCl_3$)

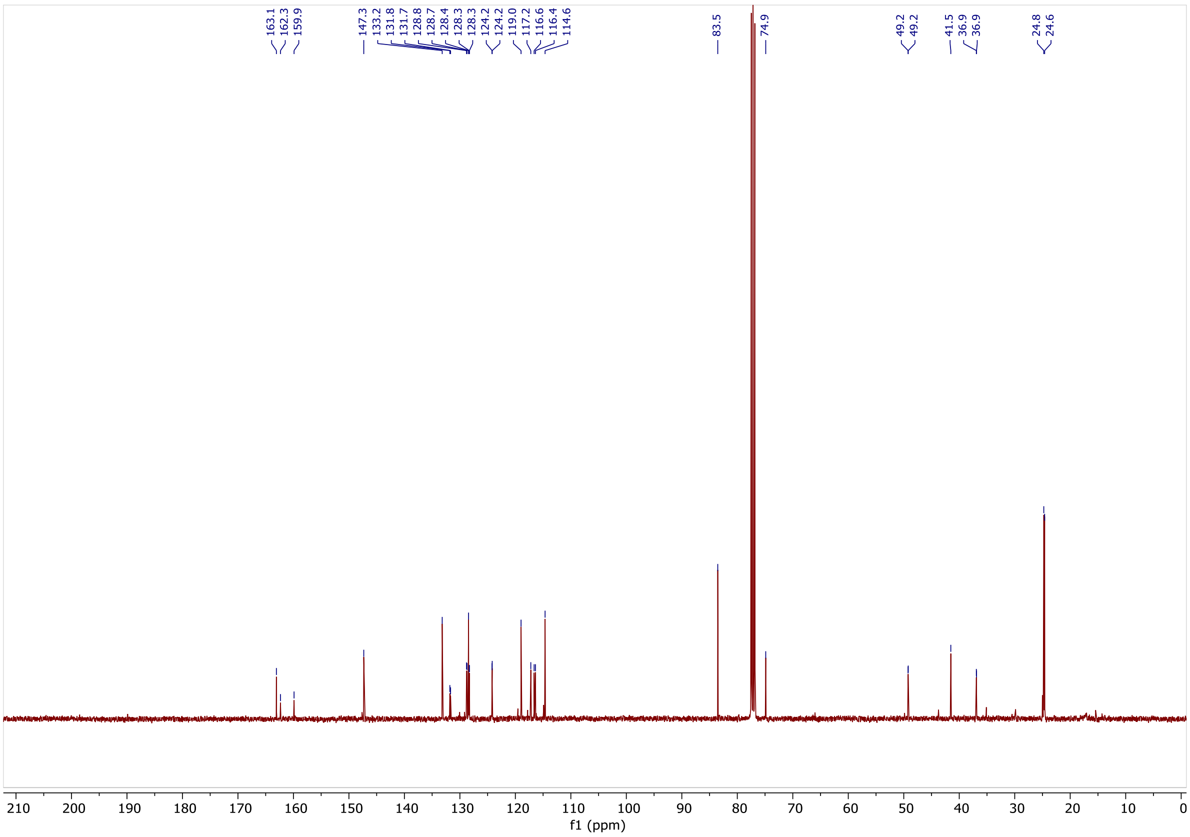

^{19}F NMR of 2h (376 MHz, $CDCl_3$)

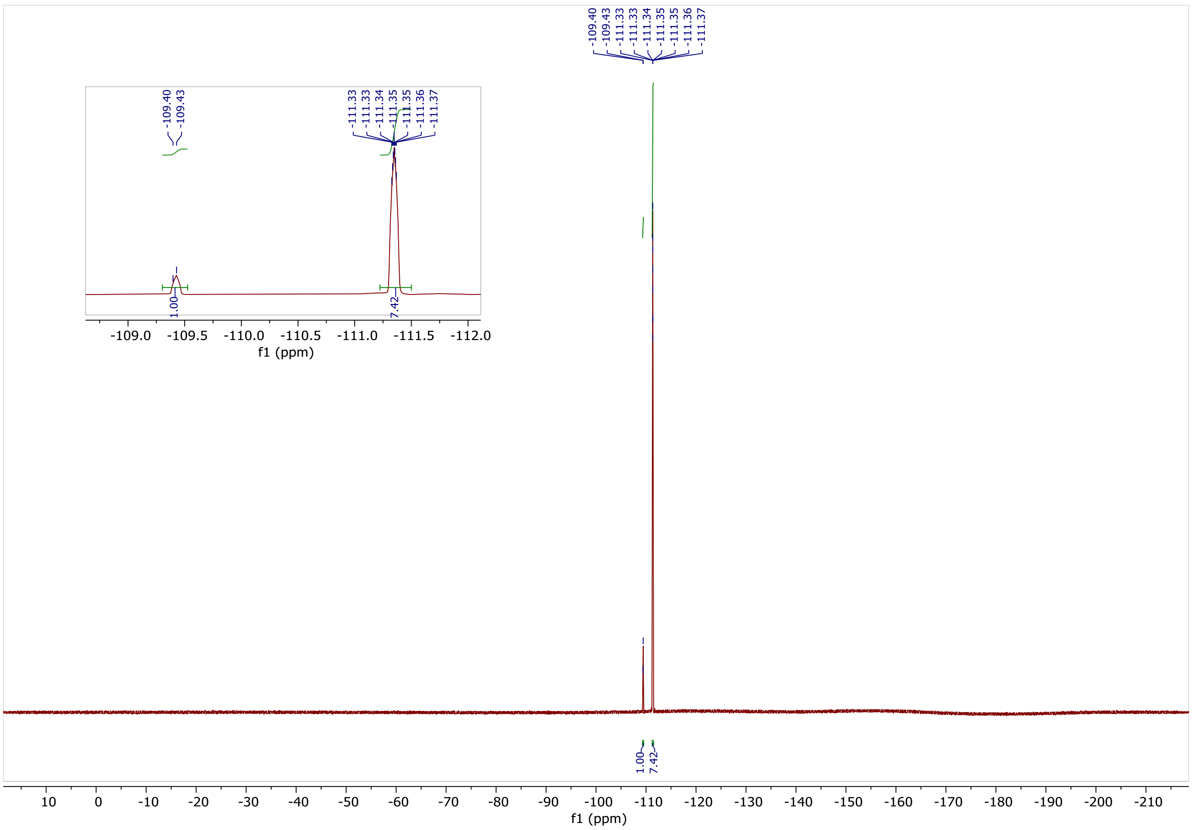

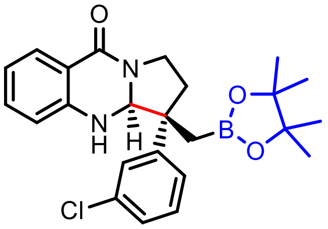

Chemical Formula: $C_{24}H_{28}BClN_2O_3$
Molecular Weight: 438.7590

(3*S*,3a*S*)-3-(3-Chlorophenyl)-3-((4,4,5,5-tetramethyl-1,3,2-dioxaborolan-2-yl)methyl)-2,3,3a,4-tetrahydropyrrolo[2,1-b]quinazolin-9(1H)-one (2i)

Compound **2i** was prepared according to General Procedure C, on a 0.1 mmol scale and running the reaction at 0 °C. The title compound was isolated by column chromatography (hexane: Et_2O, 25:75 to 0:100) as a white solid (31 mg, 0.071 mmol, 71%, dr = 91:9).

^{1}H NMR (400 MHz, 400 MHz, $CDCl_3$) δ ppm 7.93 (dd, *J* = 7.8, 1.6 Hz, 1H, ArC*H*), 7.37 – 7.26 (m, 5H, ArC*H* + ArC*H* x 4), 6.85 (td, *J* = 7.6, 1.0 Hz, 1H, ArC*H*), 6.66 (d, *J* = 8.0 Hz, 1H, ArC*H*), 5.45 (s, 1H, N*H*), 5.32 (s, 1H, NC*H*), 3.83 (ddd, *J* = 11.1, 9.4, 1.6 Hz, 1H, NC*H*$_2$), 3.76 – 3.64 (m, 1H, NC*H*$_2$), 2.34 (ddd, *J* = 12.8, 7.3, 1.6 Hz, 1H, NCH_2CH_2), 2.16 (ddd, *J* = 12.8, 10.8, 9.4 Hz, 1H,

NCH_2CH_2), 1.77 (d, *J* = 16.1 Hz, 1H, CH_2Bpin), 1.28 (d, *J* = 16.2 Hz, 1H, CH_2Bpin), 1.07 (s, 6H, 2 x CH_3), 1.00 (s, 6H, 2 x CH_3).

^{13}C NMR (101 MHz, $CDCl_3$) δ ppm 162.9 (*C*ON), 147.0 (Ar*C*), 146.8 (Ar*C*), 134.6 (Ar*C*), 133.2 (Ar*C*H), 130.0 (Ar*C*H), 128.5 (Ar*C*H), 127.1 (Ar*C*H), 126.6 (Ar*C*H), 124.3 (Ar*C*H), 119.3 (Ar*C*H), 117.4 (Ar*C*), 114.9 (Ar*C*H), 83.7 (*C*q x 2), 75.1 (N*C*H), 50.4 (*C*q), 41.7 (N*C*H₂), 38.3 (NCH_2CH_2), 24.8 (CH_3 x 2), 23.8 (CH_3 x 2). The signal for CH_2Bpin was not observed

^{11}B NMR (128 MHz, $CDCl_3$) δ 33.39

IR ν_{max} (neat/cm^{-1}): 2924, 2854, 1638, 1611, 1505, 1482, 1432, 1363, 1143, 848, 753, 694.

Mp: 150-1 °C

HRMS : calculated for $C_{24}H_{29}O_3N_2BCl$ $[M + H]^+$ 439.1954, found 439.1948.

Specific rotation: $[\alpha]_D^{28}$+ 59.95 (c 1, $CHCl_3$).

Enantiomeric purity of **2i** was determined by HPLC analysis in comparison with authentic racemic material (er = 98:2; **IA** column, 95:5 hexanes: *i*PrOH, 0.5 mL/min, 20 °C, 254 nm)

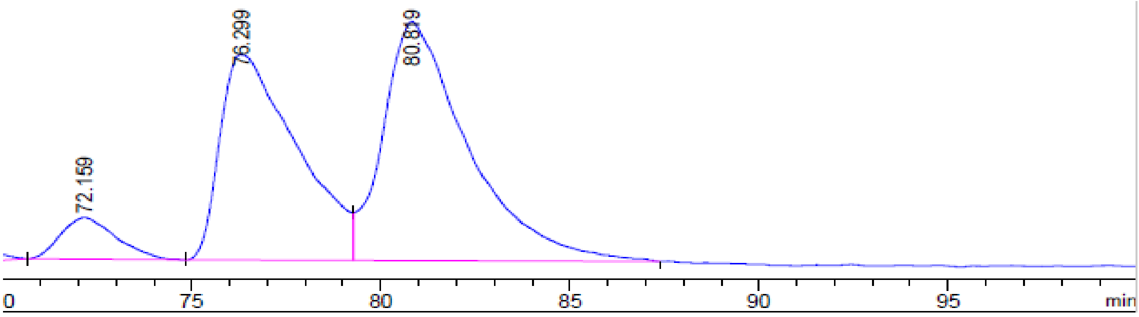

Peak #	RetTime [min]	Type	Width [min]	Area [mAU*s]	Height [mAU]	Area %
3	76.318	BB	1.6068	5013.26953	41.69369	41.4482
4	80.818	BB	1.6305	5755.28564	44.21752	47.5830

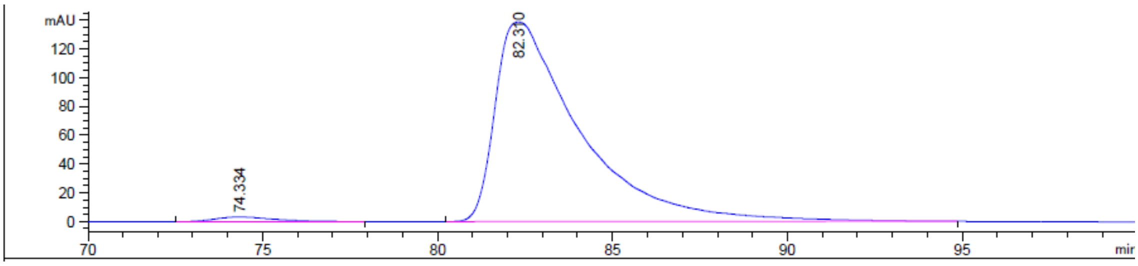

Peak #	RetTime [min]	Type	Width [min]	Area [mAU*s]	Height [mAU]	Area %
1	74.334	BB	1.4529	397.98813	3.26788	1.6687
2	82.310	BB	2.3201	2.34522e4	138.59808	98.3313

^{1}H NMR of 2i (400 MHz, $CDCl_3$)

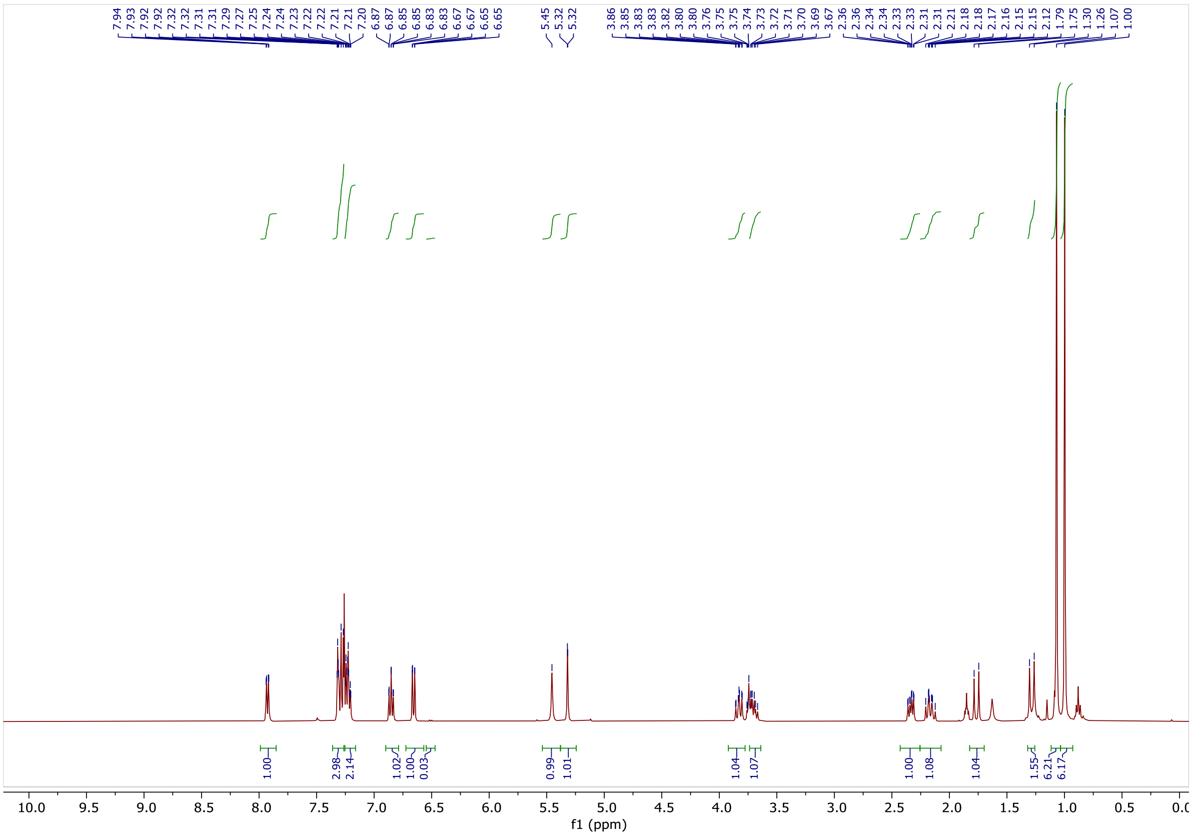

^{13}C NMR of 2i (101 MHz, $CDCl_3$)

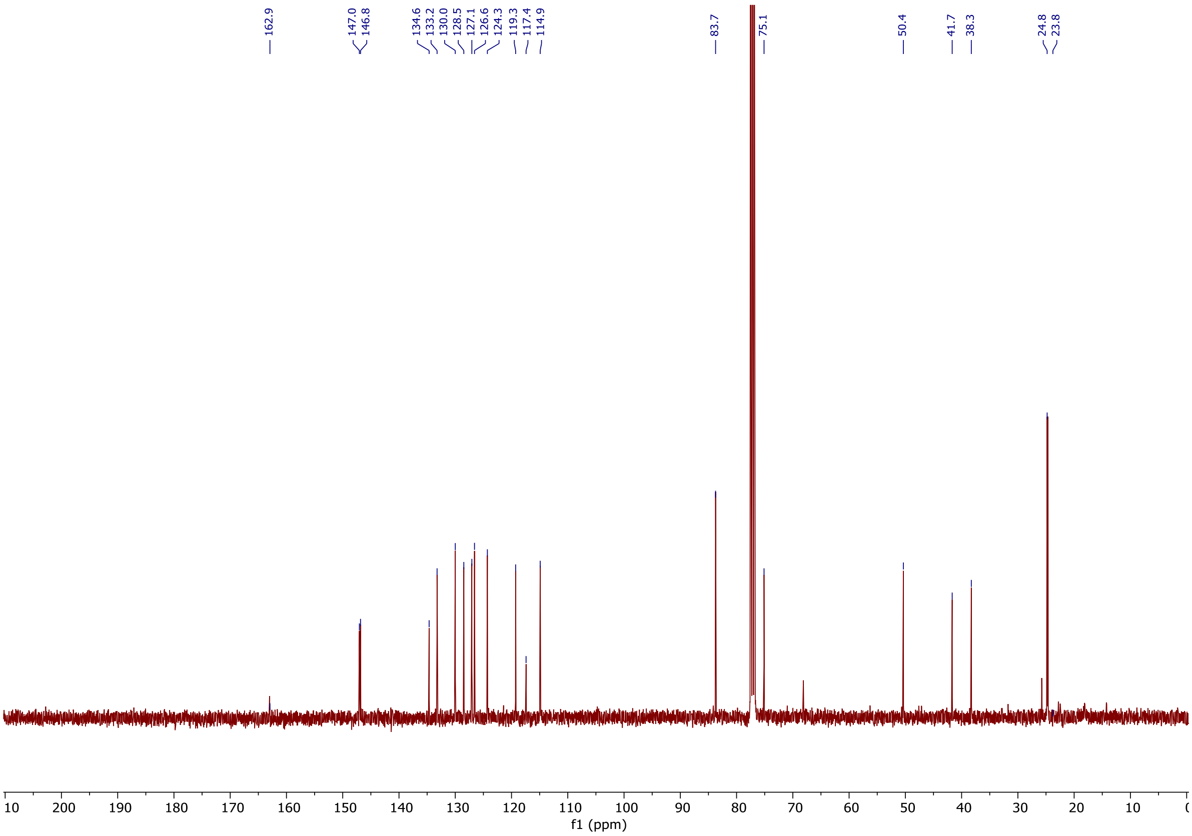

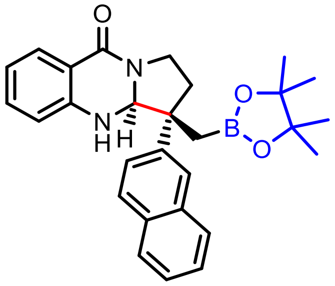

Chemical Formula: $C_{28}H_{31}BN_2O_3$
Molecular Weight: 454.3770

(3*S*,3a*S*)-3-(Naphthalen-2-yl)-3-((4,4,5,5-tetramethyl-1,3,2-dioxaborolan-2-yl)methyl)-2,3,3a,4-tetrahydropyrrolo[2,1-*b*]quinazolin-9(1*H*)-one (2j)

Compound **2j** was prepared according to General Procedure C, running the reaction at 25 °C. The title compound was isolated by column chromatography (hexane: diethyl ether, 25:75 to 0:100) as a white solid (67.6 mg, 0.149 mmol, 75%, dr = 67:33).

^{1}H NMR (400 MHz, $CDCl_3$) δ ppm δ 8.0 – 7.9 (m, 3H, ArC*H* major + ArC*H* minor + 1 x ArC*H* minor), 7.9 – 7.7 (m, 7H, 4 x ArC*H* major + 3 x ArC*H* minor), 7.7 (dd, *J* = 8.8, 2.0 Hz, 1H, ArC*H* minor), 7.5 – 7.4 (m, 5H, 3 x ArC*H* major + 2 x ArC*H* minor), 7.3 (ddd, *J* = 8.0, 7.2, 1.6 Hz, 1H, ArC*H* major), 7.2 (ddd, *J* = 8.1, 7.3, 1.6 Hz, 1H, ArC*H* minor), 6.9 – 6.8 (m, 2H, ArC*H* major + ArC*H* minor), 6.7 (dd, *J* = 8.1, 1.0 Hz, 1H, ArC*H* major), 6.5 (dd, *J* = 8.1, 1.1 Hz, 1H, ArC*H* minor), 5.5 (s, 1H, N*H* major), 5.5 (s, 2H, NC*H* major + N*H* minor), 5.2 (s, 1H, NC*H* minor), 3.9 – 3.7 (m, 4H, 2 x NCH_2 major + 2 x NCH_2 minor), 2.5 (ddd, *J* = 12.8, 7.1, 1.6 Hz, 1H, NCH_2CH_2 major), 2.4 – 2.2 (m, 3H, 1 x NCH_2CH_2 major + 2 x NCH_2CH_2 minor), 2.0 (d, *J* = 16.1 Hz, 1H, CH_2Bpin minor), 1.9 (d, *J* = 16.0 Hz, 1H, CH_2Bpin major), 1.4 (d, *J* = 16.2 Hz, 1H, CH_2Bpin major), 1.3 (d, *J* = 16.1 Hz, 1H, CH_2Bpin minor), 1.1 (s, 6H, 2 x CH_3 minor), 1.0 (s, 6H, 2 x CH_3 minor), 1.0 (s, 6H, 2 x CH_3 major), 0.9 (s, 6H, 2 x CH_3 major). Isolated as a 67:33 mixture of diastereoisomers.

^{13}C NMR (101 MHz, $CDCl_3$) δ ppm 163.0 (*C*ON major), 162.8 (*C*ON minor), 148.1 (Ar*C* minor), 147.3 (Ar*C* major), 141.8 (Ar*C* major), 140.4 (Ar*C* minor), 133.3 (Ar*C* major), 133.2 (Ar*C*H major), 133.1 (Ar*C* minor), 133.1 (ArCH minor), 132.3 (Ar*C* major), 132.1 (Ar*C* minor), 128.5 (ArCH major), 128.4 (Ar*C*H major), 128.4 (Ar*C*H minor), 128.2 (Ar*C*H minor), 128.0 (Ar*C*H major), 127.7 (Ar*C*H minor), 127.6 (Ar*C*H major), 127.4 (Ar*C*H minor), 126.4 (Ar*C*H major), 126.3 (2 x Ar*C*H minor), 126.1 (Ar*C*H minor), 126.0 (Ar*C*H major), 126.0 (Ar*C*H minor), 124.5 (Ar*C*H major), 124.5 (Ar*C*H major), 119.0 (Ar*C*H major), 119.0 (Ar*C*H minor), 117.3 (*ArC* major), 117.2 (*ArC* minor), 114.8 (Ar*C*H major), 114.7 (Ar*C*H minor), 83.9 (2 x *C*q minor), 83.5 (2 x *C*q

major), 79.5 (NHCH minor), 75.5 (NH*C*H major), 50.5 (N*C*H_2 major), 50.4 (N*C*H_2 minor), 43.0 (*C*q minor), 41.8 (*C*q major), 39.4 (NCH_2*C*H_2 minor), 38.1 (NCH_2*C*H_2 major), 24.7 (2 x *C*H_3 minor), 24.7 (2 x *C*H_3 minor + 2 x *C*H_3 major), 24.5 (2 x *C*H_3 major). The signal for B*C*H_2 was not observed.

^{11}B NMR (128 MHz, $CDCl_3$) δ ppm 34.2

IR ν_{max} (neat/cm^{-1}): 2930, 1602, 1498, 1468, 1435, 1344, 1291, 1241, 1159, 1109, 1024, 910, 885, 758, 698.

HRMS : calculated for $C_{28}H_{32}O_3N_2B$ [M + H]$^+$ 455.2500, found 455.2483.

Mp: analysis not obtained (inseparable mixture of diastereomers).

Specific rotation: analysis not obtained (inseparable mixture of diastereomers).

Enantiomeric purity of **2j** was determined by HPLC analysis in comparison with authentic racemic material (er$_{(major)}$ = 95:5; er$_{(minor)}$ = 94:6 **IA** column, 90:10 hexanes: *i*PrOH, 0.5 mL/min, 20 °C, 254 nm) .

Chromatograms for racemic 2j

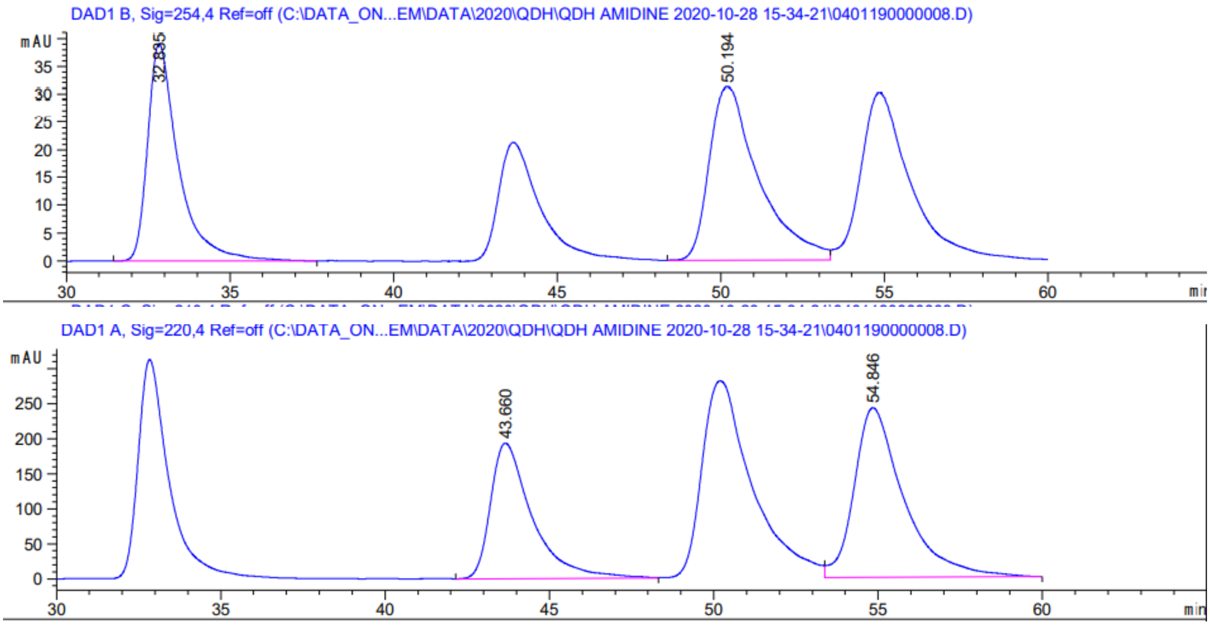

Peak #	RetTime [min]	Type	Width [min]	Area [mAU*s]	Height [mAU]	Area %
1	32.836	VV	0.9678	2.06194e4	313.29349	40.8525
2	50.195	VV	1.5381	2.98534e4	281.32077	59.1475

Peak #	RetTime [min]	Type	Width [min]	Area [mAU*s]	Height [mAU]	Area %
1	32.835	BB	0.9661	2568.98071	39.12075	43.8735
2	50.194	BV	1.5301	3286.45093	31.16858	56.1265

Chromatogram for enantioenriched 2j

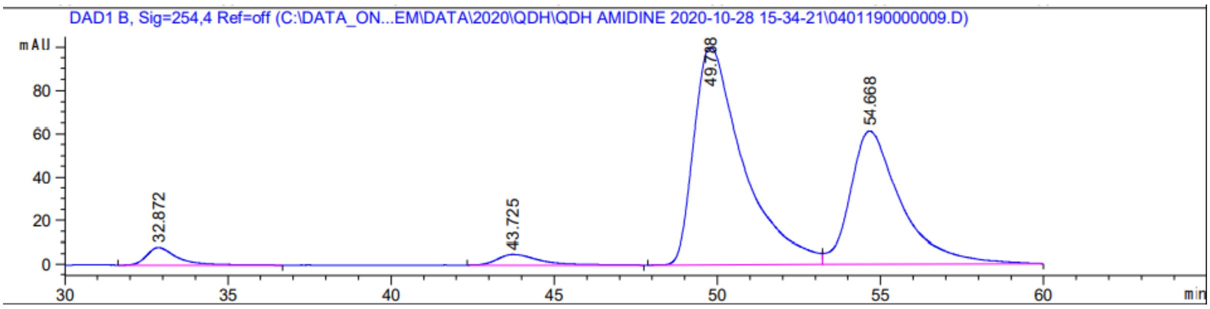

Peak #	RetTime [min]	Type	Width [min]	Area [mAU*s]	Height [mAU]	Area %
1	32.872	BB	0.9671	528.27875	8.07632	2.9205
2	43.725	BB	1.2836	443.90048	4.94337	2.4540
3	49.788	BV	1.5237	1.05049e4	100.14904	58.0740
4	54.668	VBA	1.5638	6611.72314	61.42211	36.5515

^{1}H NMR of 2j (400 MHz, $CDCl_3$)

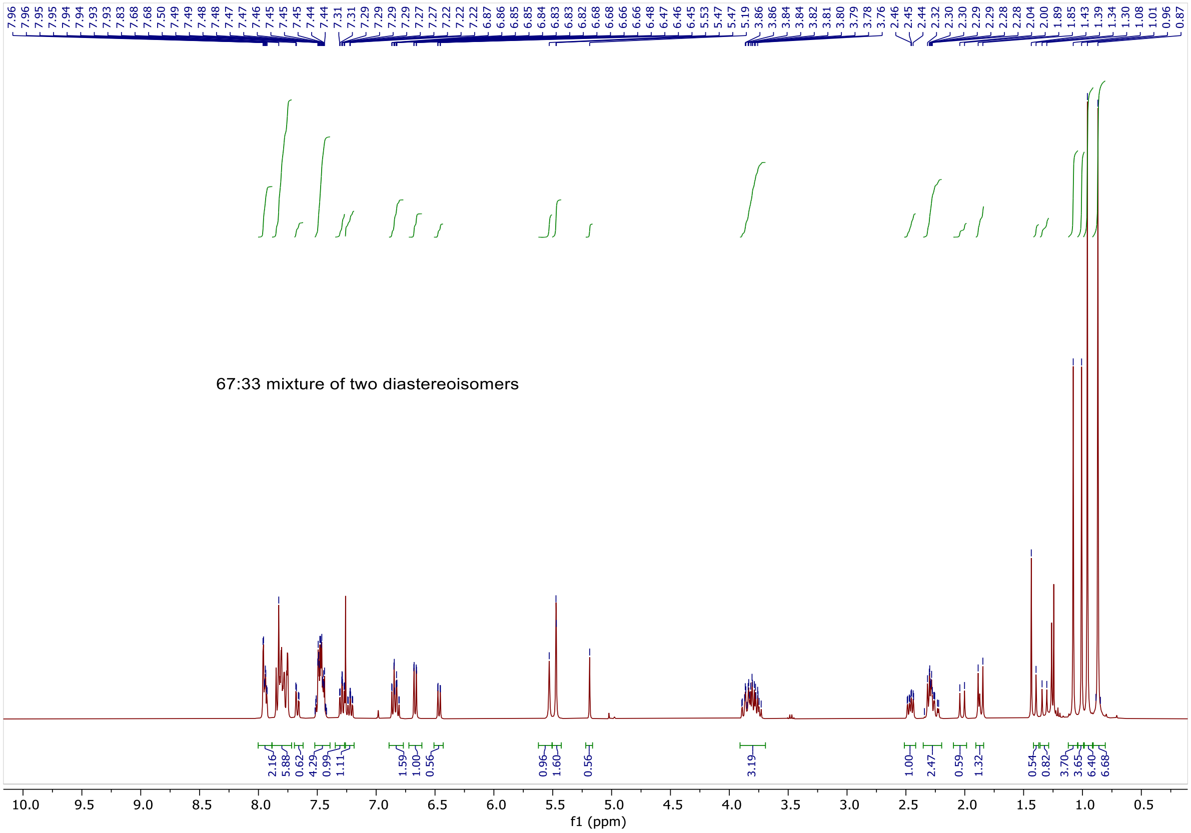

^{13}C NMR of 2j (101 MHz, CDCl$_3$)

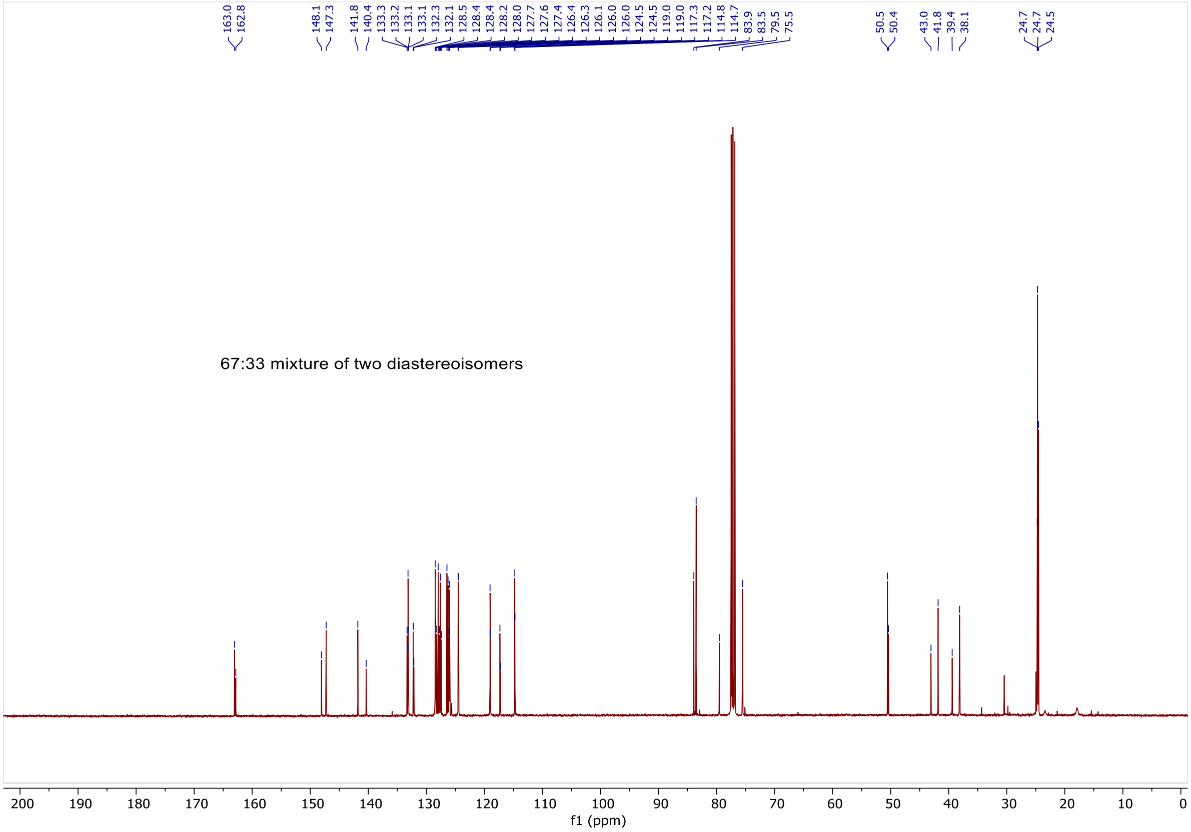

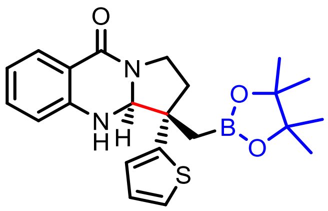

Chemical Formula: $C_{22}H_{27}BN_2O_3S$
Molecular Weight: 410.34

(3*R*,3a*S*)-3-((4,4,5,5-Tetramethyl-1,3,2-dioxaborolan-2-yl)methyl)-3-(thiophen-2-yl)-2,3,3a,4-tetrahydropyrrolo[2,1-*b*]quinazolin-9(1*H*)-one (2k)

Compound **2k** was prepared according to General Procedure C, running the reaction at 25 °C. The title compound was isolated by column chromatography (hexane: ethyl acetate, 80:20 - 50:50) as a brown oil (64.8 mg, 0.158 mmol, 79%, dr = 95:5).

^{1}H NMR (400 MHz, CDCl$_3$) δ ppm 7.88 (dd, *J* = 7.8, 1.6 Hz, 1H, ArC*H*), 7.27 – 7.19 (m, 2H, ArC*H* + ArC*H*), 7.00 – 6.95 (m, 2H, ArC*H* + ArC*H*), 6.86 – 6.79 (m, 1H, ArC*H*), 6.60 (d, *J* = 8.0 Hz, 1H, ArC*H*), 5.18 (s, 1H, N*H*), 4.80 (s, 1H, NHC*H*), 3.85 – 3.70 (m, 2H, NC*H*$_2$), 2.52 (ddd, *J* = 12.9, 6.8,

2.4 Hz, 1H, NCH_2CH_2), 2.34 – 2.21 (m, 1H, NCH_2CH_2), 1.73 (d, *J* = 15.1 Hz, 1H, BCH_2), 1.31 (d, *J* = 16.0 Hz, 1H, BCH_2), 1.09 (s, 6H, CH_3 x 2), 1.08 (s, 6H, CH_3 x 2).

^{13}C NMR (101 MHz, $CDCl_3$) δ ppm 162.7 (*C*ON), 148.7 (Ar*C*), 147.2 (Ar*C*), 133.2 (Ar*C*H), 128.3 (Ar*C*H), 127.1 (Ar*C*H), 123.8 (Ar*C*H), 123.7 (Ar*C*H), 119.3 (Ar*C*H), 116.9 (Ar*C*), 114.7 (Ar*C*H), 83.5 (*C*q x 2), 78.0 (NH*C*H), 49.1 (*C*q), 42.2 (NCH_2), 37.4 (NCH_2CH_2), 24.8 (CH_3 x 2), 24.7 (CH_3 x 2). The signal for BCH_2 was not observed.

^{11}B NMR (128 MHz, $CDCl_3$) δ ppm 33.1.

IR ν_{max} (neat/cm^{-1}): 3368, 3067, 2977, 1671, 1471, 1365, 1260, 1143, 1032, 848, 775, 696.

HRMS: calculated for $C_{22}H_{28}N_2O_3BS$ $[M + H]^+$ 411.1835, found 411.1893.

Specific rotation: $[\alpha]_D^{26}$ + 7.75 (c 0.48, $CHCl_3$).

Enantiomeric purity of **2k** was determined by HPLC analysis in comparison with authentic racemic material (er = 98:2; **IA** column, 90:10 hexanes: *i*PrOH, 0.5 mL/min, 20 °C, 254 nm)

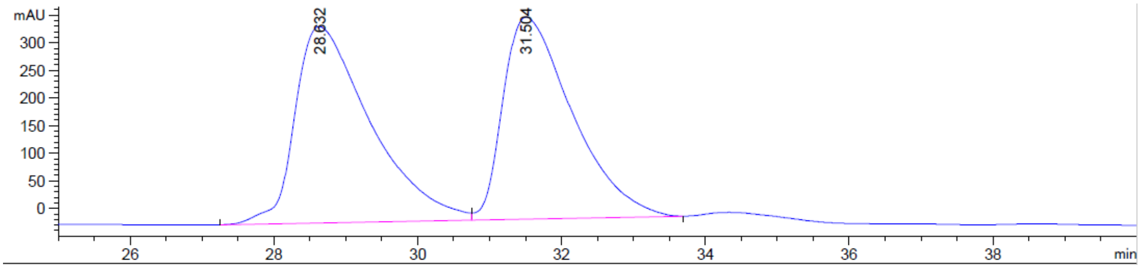

Peak #	RetTime [min]	Type	Width [min]	Area [mAU*s]	Height [mAU]	Area %
1	28.632	BV	1.0620	2.60471e4	355.84473	51.4939
2	31.504	VB	1.0142	2.45358e4	365.95679	48.5061

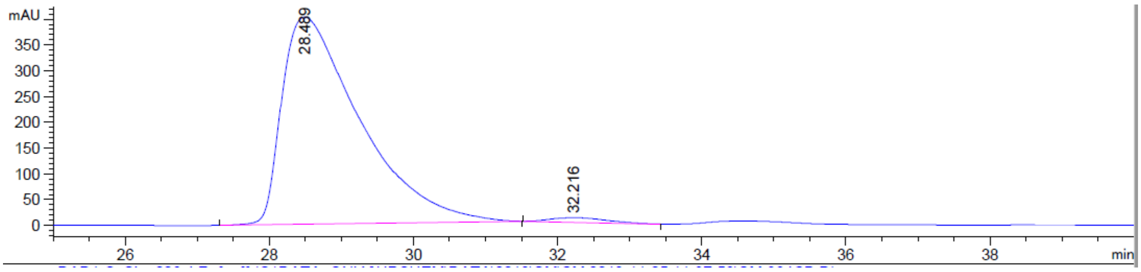

Peak #	RetTime [min]	Type	Width [min]	Area [mAU*s]	Height [mAU]	Area %
1	28.489	BB	1.0924	3.01672e4	401.57123	98.4362
2	32.216	BB	0.6303	479.25464	9.04242	1.5638

^1H NMR of 2k (400 MHz, $CDCl_3$)

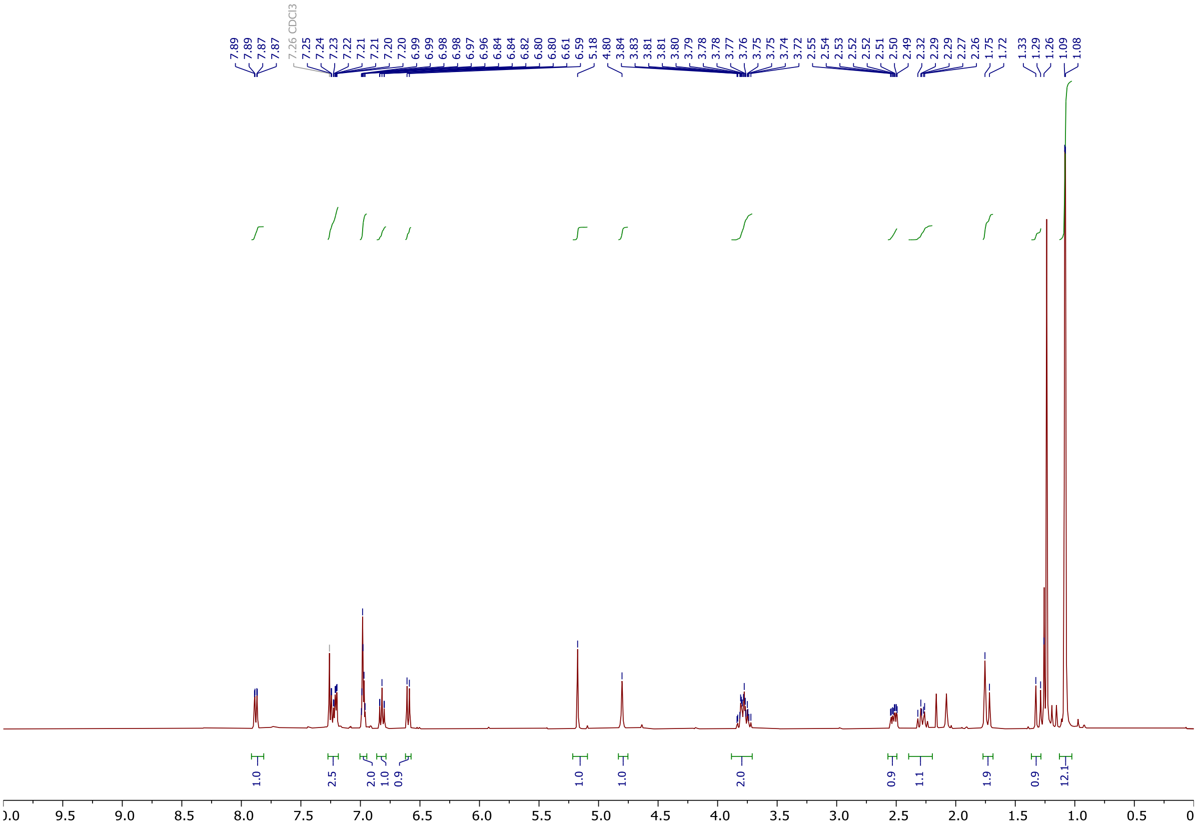

^{13}C NMR of 2k (101 MHz, $CDCl_3$)

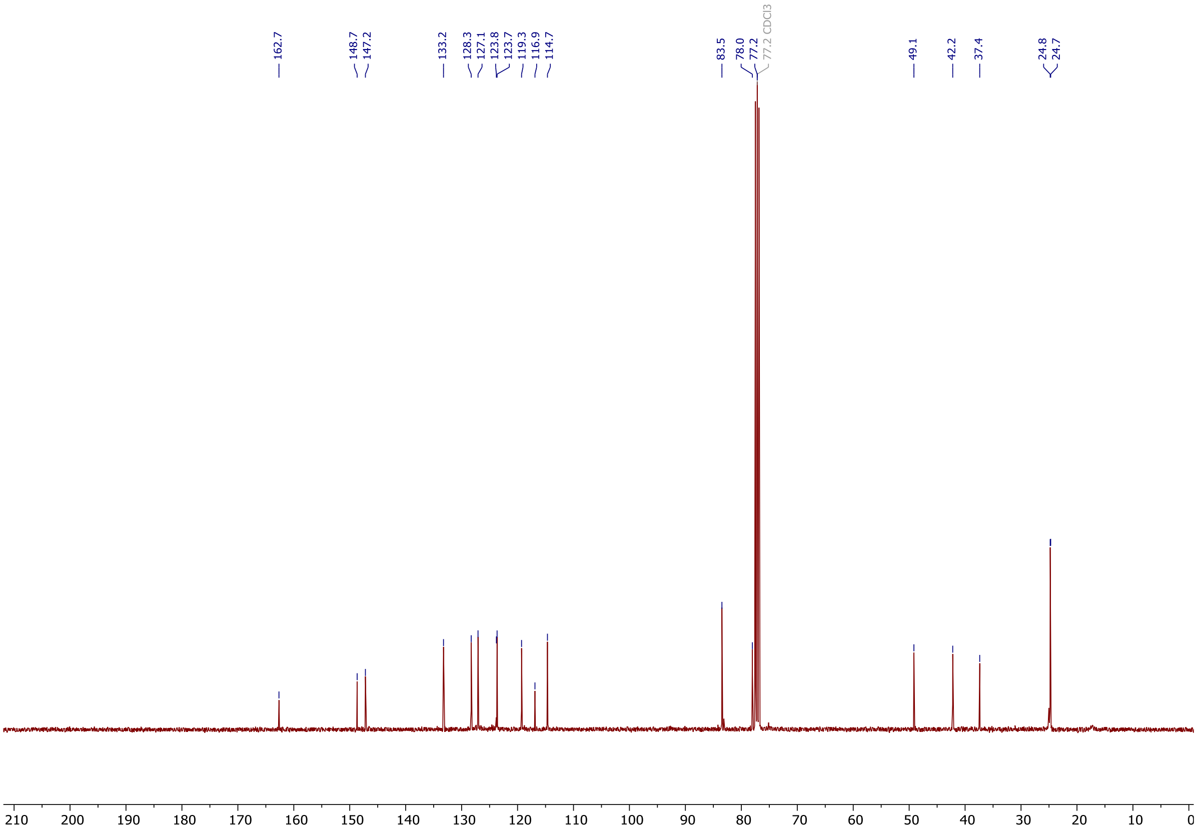

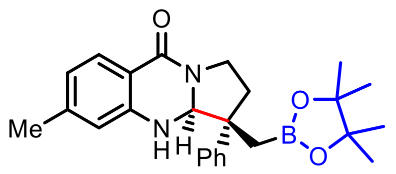

Chemical Formula: $C_{25}H_{31}BN_2O_3$
Molecular Weight: 418,34

(3*S*,3a*S*)-6-Methyl-3-phenyl-3-((4,4,5,5-tetramethyl-1,3,2-dioxaborolan-2-yl)methyl)-2,3,3a,4-tetrahydropyrrolo[2,1-*b*]quinazolin-9(1*H*)-one (2l)

Compound **2l** was prepared according to General Procedure C, running the reaction at 25 °C. The title compound was isolated by column chromatography (hexane: ethyl acetate, 80:20 - 50:50) as a white solid (66.0 mg, 0.158 mmol, 79%, dr = 91:9).

^{1}H NMR (400 MHz, $CDCl_3$) δ ppm 7.80 (d, *J* = 7.9 Hz, 1H, ArC*H*), 7.36 – 7.28 (m, 4H, ArC*H* x 4), 7.23 (td, *J* = 5.9, 2.6 Hz, 1H, ArC*H*), 6.65 (dd, *J* = 8.0, 1.5 Hz, 1H, ArC*H*), 6.45 (s, 1H, ArC*H*), 5.42 (s, 1H, N*H*), 5.30 (d, *J* = 1.2 Hz, 1H, NHC*H*), 3.81 (ddd, *J* = 11.0, 9.4, 1.5 Hz, 1H, NCH_2), 3.69 (td, *J* = 11.4, 7.1 Hz, 1H, NCH_2), 2.34 (ddd, *J* = 12.9, 7.2, 1.6 Hz, 1H, NCH_2CH_2), 2.30 (s, 3H, CH_3), 2.17 (ddd, *J* = 13.3, 10.9, 9.3 Hz, 1H, NCH_2CH_2), 1.77 (d, *J* = 15.9 Hz, 1H, BCH_2), 1.29 (d, *J* = 16.0 Hz, 1H, BCH_2), 1.03 (s, 6H, CH_3 x 2), 0.95 (s, 6H, CH_3 x 2).

^{13}C NMR (101 MHz, $CDCl_3$) δ ppm 163.2 (*C*ON), 147.3 (Ar*C*), 144.7 (Ar*C*), 143.8 (Ar*C*), 128.7 (Ar*C*H x 2), 128.4 (Ar*C*H), 126.8 (Ar*C*H), 126.0 (Ar*C*H x 2), 120.3 (Ar*C*H), 115.7 (Ar*C*), 114.7 (Ar*C*H), 83.5 (*C*q x 2), 75.3 (NH*C*H), 50.2 (NCH_2), 41.6 (*C*q), 38.1 (NCH_2CH_2), 24.7 (CH_3 x 2), 24.6 (CH_3 x 2), 21.8 (CH_3). The signal for BCH_2 was not observed.

^{11}B NMR (128 MHz, $CDCl_3$) δ ppm 30.3

IR ν_{max} (neat/cm^{-1}): 3325, 2975, 1671, 1615, 1364, 1324, 1115, 849,698.

Mp: 177°C

HRMS : calculated for $C_{25}H_{31}N_2O_3BNa$ $[M + Na]^+$ 441.3440, found 441.3463.

Specific rotation: $[\alpha]_D^{24}$ + 38.32 (c 1.9, $CHCl_3$).

Enantiomeric purity of **2l** was determined by HPLC analysis in comparison with authentic racemic material (er = 97:3; **IA** column, 90:10 hexanes: *i*PrOH, 0.5 mL/min, 20 °C, 254 nm).

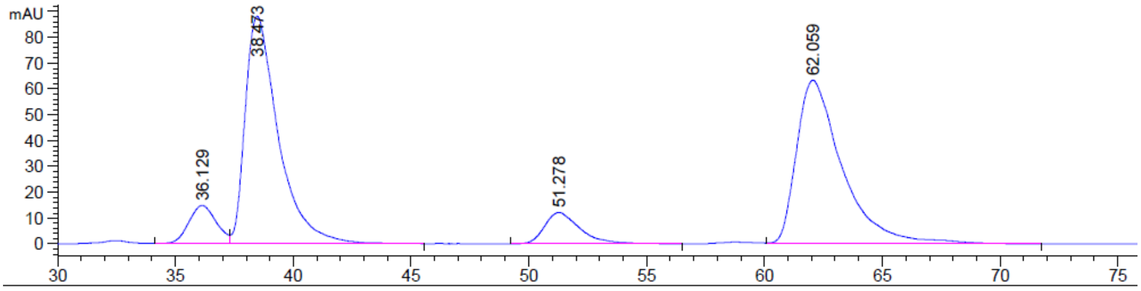

Peak #	RetTime [min]	Type	Width [min]	Area [mAU*s]	Height [mAU]	Area %
4	36.127	BV	1.1342	7337.75146	91.43832	5.4031
5	38.472	VB	1.3893	5.35939e4	550.73657	39.4635
6	51.277	BB	1.3455	7639.97803	74.80816	5.6256
7	62.060	BB	1.8311	5.30497e4	395.96356	39.0628

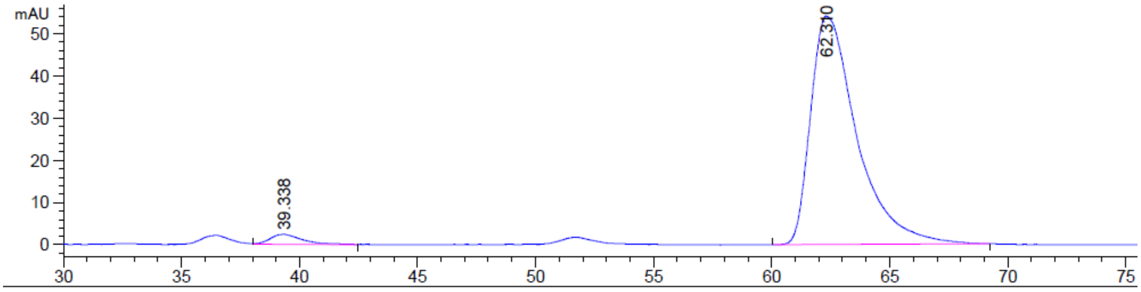

Peak #	RetTime [min]	Type	Width [min]	Area [mAU*s]	Height [mAU]	Area %
1	39.338	VB	1.1683	230.31639	2.38139	3.0314
2	62.310	BB	1.9204	7367.26611	54.11228	96.9686

^{1}H NMR of 2l (400 MHz, $CDCl_3$)

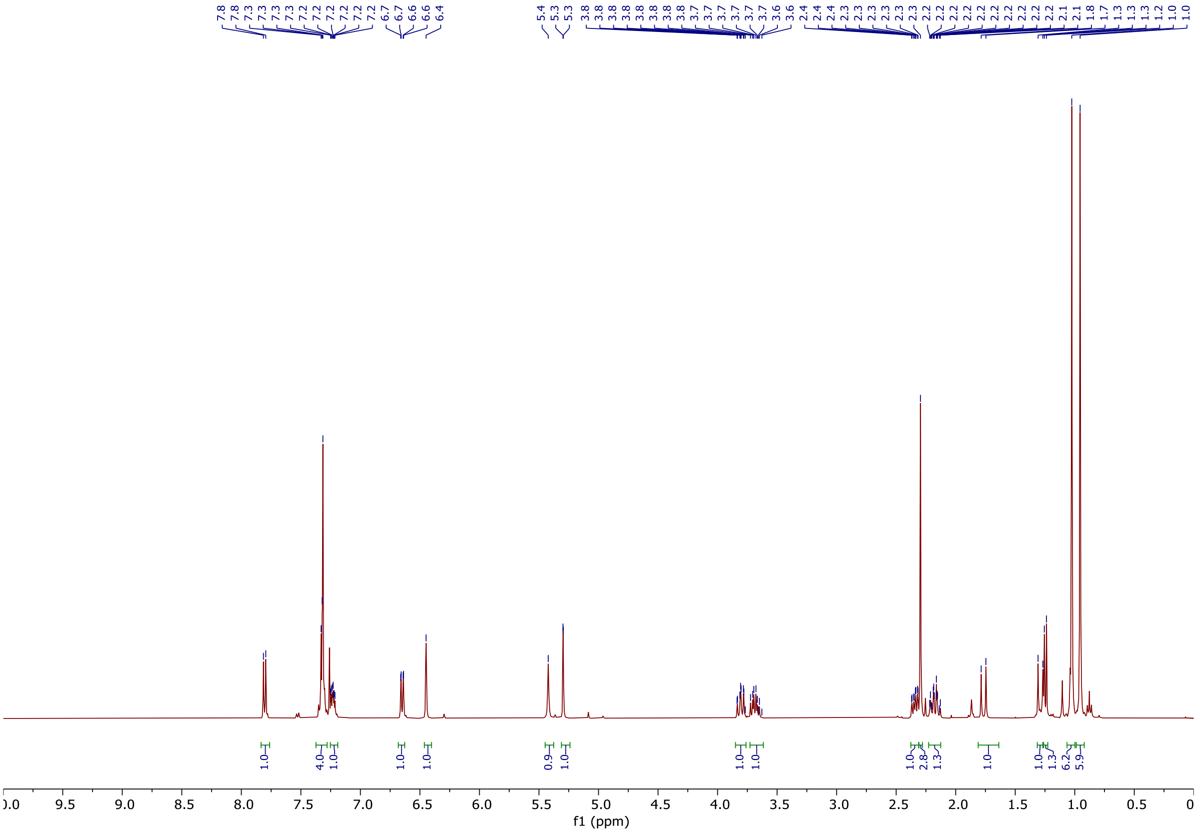

^{13}C NMR of 2l (101 MHz, CDCl$_3$)

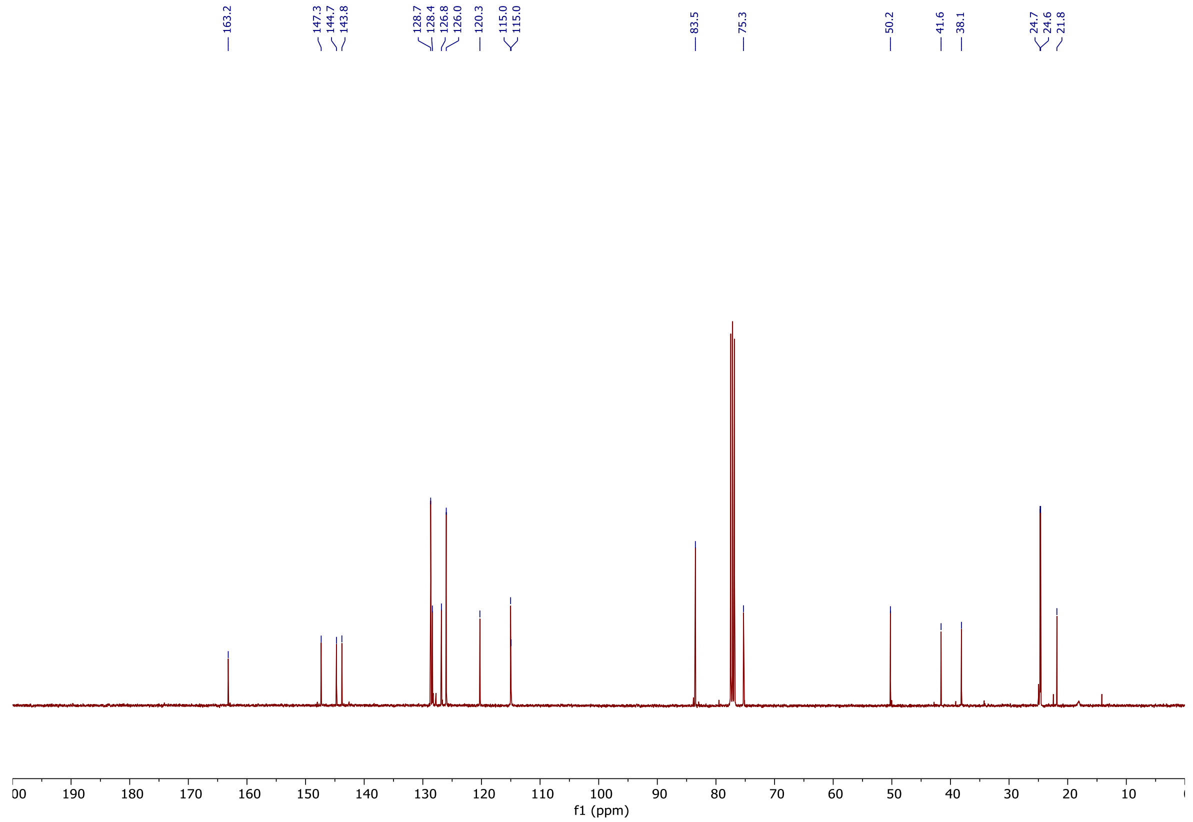

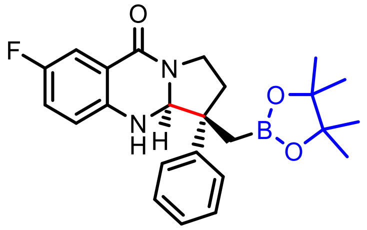

Chemical Formula: $C_{24}H_{28}BFN_2O_3$
Molecular Weight: 422.31

(3*S*,3a*S*)-7-Fluoro-3-phenyl-3-((4,4,5,5-tetramethyl-1,3,2-dioxaborolan-2-yl)methyl)-2,3,3a,4-tetrahydropyrrolo[2,1-*b*]quinazolin-9(1*H*)-one (2m)

Compound **2m** was prepared according to General Procedure C, running the reaction at 25 °C. The title compound was isolated by column chromatography (hexane: ethyl acetate, 80:20 - 50:50) as a white solid (66.0 mg, 0.156 mmol, 78%, dr = 80:20).

^{1}H NMR (400 MHz, CDCl$_3$) δ ppm 7.65 (dd, *J* = 8.8, 3.0 Hz, 1H, ArC*H,* major), 7.62 (dd, *J* = 8.8, 3.0 Hz, 1H, ArC*H,* minor), 7.58 – 7.53 (m, 1H, ArC*H,* minor), 7.41 – 7.23 (m, 9H, ArC*H*, 5H major + 4H minor), 7.04 (td, *J* = 8.4, 3.0 Hz, 1H, ArC*H,* major), 6.99 (td, *J* = 8.8, 8.0, 3.3 Hz, 1H, ArC*H*, minor), 6.65 (dd, *J* = 8.8, 4.2 Hz, 1H, ArC*H*, major), 6.50 (dd, *J* = 8.8, 4.2 Hz, 1H, ArC*H*, minor), 5.47 (d, *J* = 1.8 Hz, 1H, N*H*, major), 5.34 – 5.30 (m, 2H, 1H, NHC*H*, major + 1H, N*H,* minor), 5.10

(d, J = 1.2 Hz, 1H, NHCH, minor), 3.90 – 3.78 (m, 2H, NCH_2, 1H major + 1H minor), 3.73 (m, 2H, NCH_2, 1H major + 1H minor), 2.46 – 2.35 (m, 1H, NCH$_2$CH_2, major), 2.32 – 2.16 (m, 3H, NCH$_2$CH_2, 1H major + 2H minor), 1.89 (d, J = 16.0 Hz, 1H, BCH_2, minor), 1.78 (d, J = 15.9 Hz, 1H, BCH_2, major), 1.34 (d, J = 15.9 Hz, 1H, BCH_2, major), 1.28 (d, J = 16.0 Hz, 1H, BCH_2, minor), 1.13 (s, 6H, CH_3 x 2, minor), 1.07 (s, 6H, CH_3 x 2, minor), 1.05 (s, 6H, CH_3 x 2, major), 1.00 (s, 6H, CH_3 x 2, major). Isolated as a 80:20 mixture of diastereomers.

^{13}C NMR (101 M Hz, CDCl$_3$) δ ppm 162.1 (d, $^4J_{C\text{-}F}$ = 2.4 Hz, *C*ON), 156.6 (d, $^1J_{C\text{-}F}$ = 237.5 Hz, Ar*C*), 144.5 (ArC), 143.48 (d, $^4J_{C\text{-}F}$ = 1.8 Hz, ArC), 128.7 (ArCH x 2), 127.0 (ArCH), 126.0 (ArCH x 2), 120.4 (d, $^2J_{C\text{-}F}$ = 23.9 Hz, ArCH), 118.6 (d, $^3J_{C\text{-}F}$ = 6.9 Hz, ArC), 116.2 (d, $^3J_{C\text{-}F}$ = 7.0 Hz, ArCH), 114.17 (d, $^2J_{C\text{-}F}$ = 23.6 Hz , ArCH), 83.6 (*C*q x 2), 75.5 (NH*C*H, major), 50.3 (N*C*H$_2$, major), 41.8 (*C*q, major), 38.2 (NCH$_2$*C*H$_2$, major), 24.7 (CH$_3$ x 2), 24.6 (CH$_3$ x 2). The signal for B*C*H$_2$ was not observed. Only the signals for the major diastereomer are reported.

^{19}F NMR (376 MHz, CDCl$_3$) δ ppm -124.92 (td, J = 8.4, 4.3 Hz, 1F).

^{11}B NMR (128 MHz, CDCl$_3$) δ ppm 32.3.

IR ν_{max} (neat/cm^{-1}): 3696, 2980, 2865, 1638, 1498, 1360, 1143, 1054, 1032, 1012.

Mp: 151 °C.

HRMS : calculated for $C_{24}H_{28}N_2O_3BFNa$ $[M + Na]^+$ 445.2063, found 445.2069.

Specific rotation: $[\alpha]_D^{26}$ - 20.00 (c 0.01, CHCl$_3$).

Enantiomeric purity of **2m** was determined by HPLC analysis in comparison with authentic racemic material (er = 94:6; **IA** column, 90:10 hexanes: *i*PrOH, 0.5 mL/min, 20 °C, 254 nm).

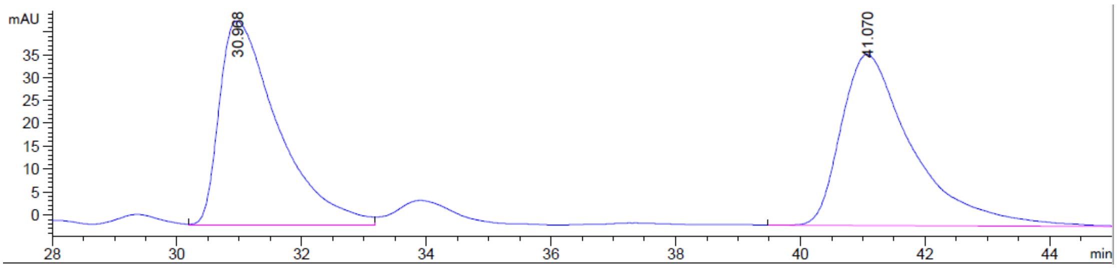

Peak #	RetTime [min]	Type	Width [min]	Area [mAU*s]	Height [mAU]	Area %
1	30.968	VV	0.9765	2948.71069	44.76279	49.4444
2	41.070	BB	1.1853	3014.97534	37.52817	50.5556

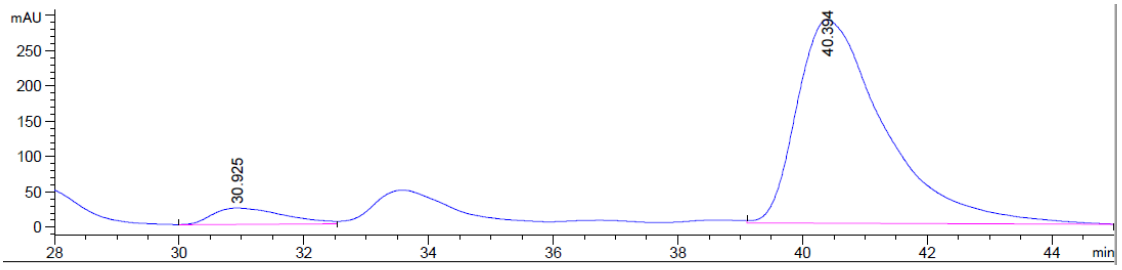

Peak #	RetTime [min]	Type	Width [min]	Area [mAU*s]	Height [mAU]	Area %
1	30.925	BV	1.1368	1836.38477	23.06685	6.1349
2	40.394	VBA	1.4579	2.80969e4	288.08270	93.8651

^{1}H NMR of 2m (400 MHz, $CDCl_3$)

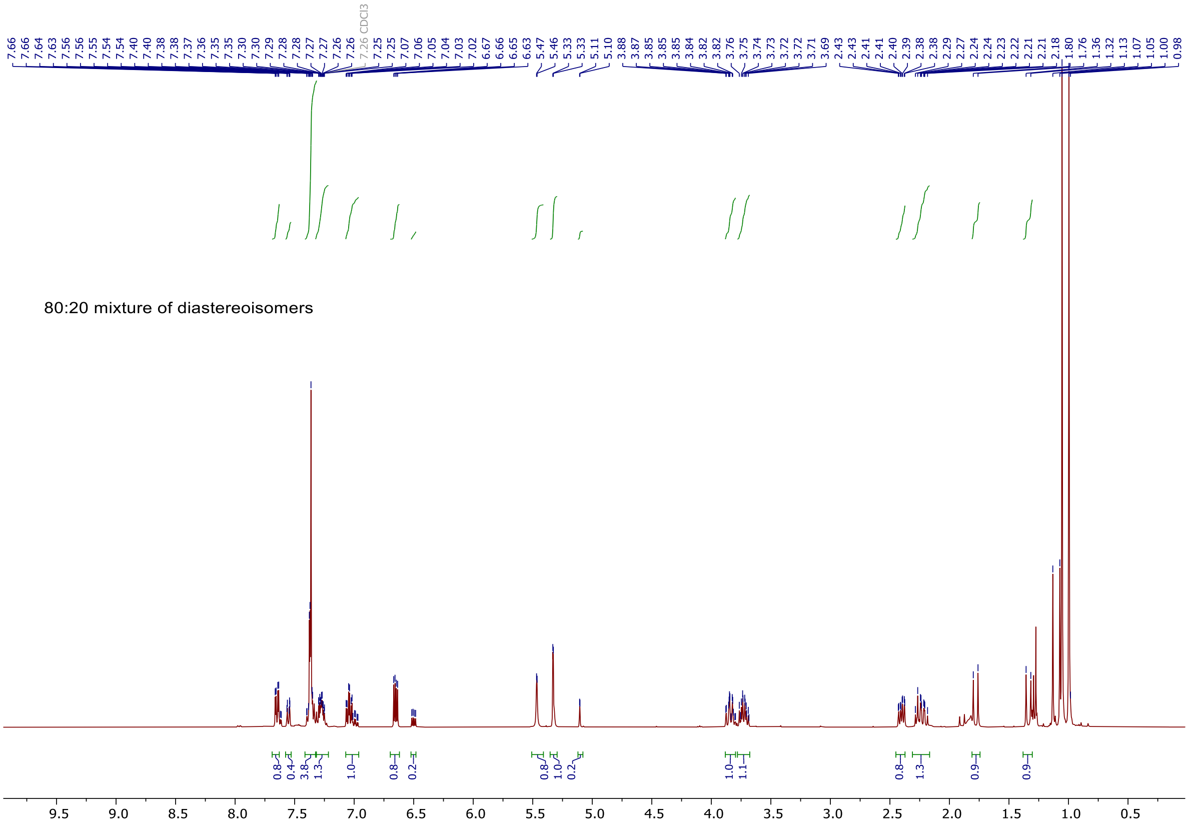

^{13}C NMR of 2m (101 MHz, $CDCl_3$)

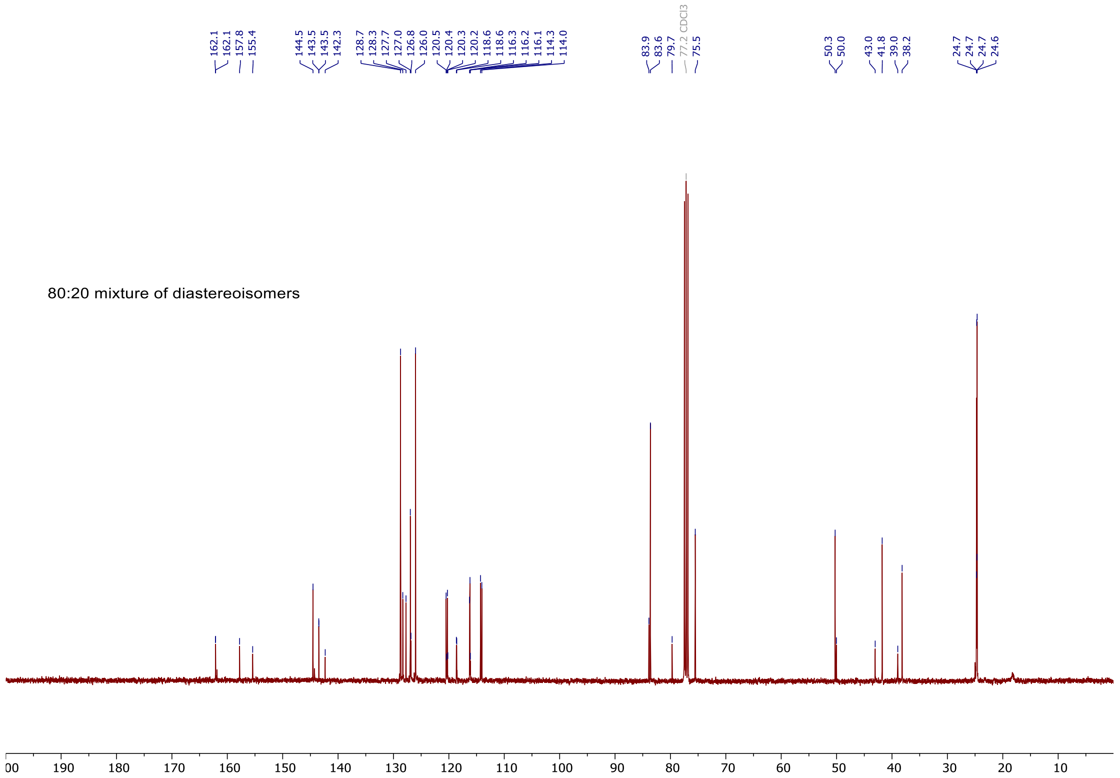

^{19}F NMR of 2m (376 MHz, $CDCl_3$)

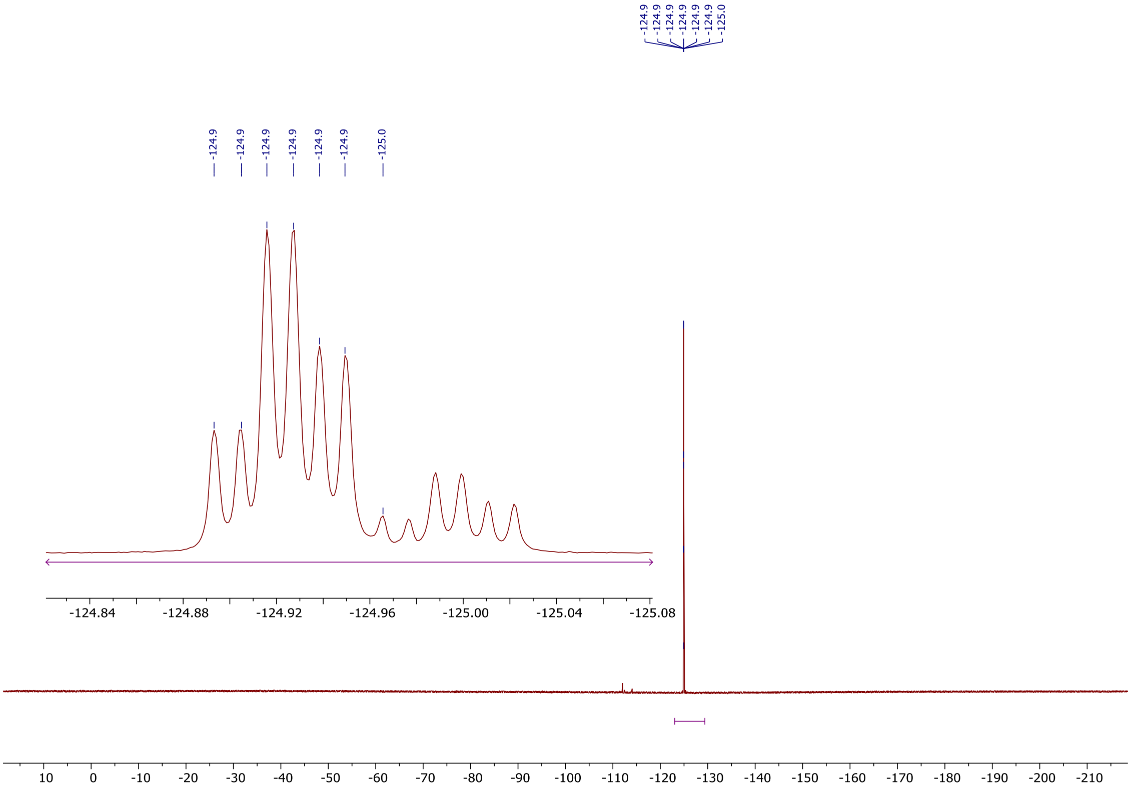

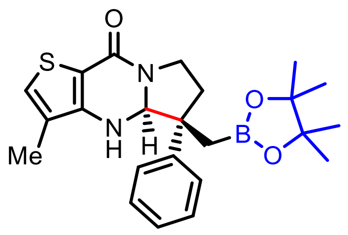

Chemical Formula: $C_{23}H_{29}BN_2O_3S$
Molecular Weight: 424.37

(4a*S*,5*R*)-3-Methyl-5-phenyl-5-((4,4,5,5-tetramethyl-1,3,2-dioxaborolan-2-yl)methyl)-4a,5,6,7-tetrahydropyrrolo[1,2-*a*]thieno[3,2-*d*]pyrimidin-9(4H)-one (2n)

Compound **2n** was prepared according to General Procedure C, on a 0.2 mmol scale, running the reaction at 25 °C, and without the use of *i*PrOH. The title compound was isolated by column chromatography (EtOAc: diethyl ether, 0:100 to 10:90) as a white solid (56 mg, 0.013 mmol, 66%, dr > 95:5).

^{1}H NMR (400 MHz, 400 MHz, $CDCl_3$) δ ppm 7.43 – 7.29 (m, 4H, ArC*H*), 7.27 – 7.17 (m, 1H, ArC*H*), 6.81 (d, *J* = 1.5 Hz, 1H, ArC*H*), 5.80 (d, *J* = 5.4 Hz, 1H, N*H*), 5.32 (d, *J* = 5.4 Hz, 1H, NHC*H*), 3.75 (ddd, *J* = 10.9, 9.3, 1.3 Hz, 1H, NCH_2), 3.57 (td, *J* = 11.4, 7.0 Hz, 1H, NCH_2), 2.42 – 2.36 (m, 1H, NCH_2CH_2), 2.34 (d, *J* = 1.2 Hz, 3H, CH_3), 2.17 (ddd, *J* = 12.6, 11.1, 9.3 Hz, 1H, NCH_2CH_2), 1.67 (d, *J* = 15.5 Hz, 1H, BCH_2), 1.33 (d, *J* = 15.5 Hz, 1H, BCH_2), 1.00 (s, 6H, CH_3 x 2), 0.98 (s, 6H, CH_3 x 2).

^{13}C NMR (101 MHz, $CDCl_3$) δ ppm 161.0 (*C*ON), 155.6 (Ar*C*), 144.8 (Ar*C*), 128.7 (Ar*C*H x 2), 126.9 (Ar*C*H), 126.9 (Ar*C*), 126.1 Ar*C*H x 2), 121.1 (Ar*C*H), 117.8 (Ar*C*), 83.9 (*C*q x 2), 77.2 (NH*C*H), 49.8 (NCH_2), 40.5 (*C*q), 39.0 (NCH_2CH_2), 24.7 (CH_3 x 2), 24.7 (CH_3 x 2), 15.4 (CH_3). The signal for BCH_2 was not observed.

^{11}B NMR (128 MHz, $CDCl_3$) δ 35.49.

IR ν_{max} (neat/cm^{-1}): 3249, 2977, 1618, 1492, 1360, 1144, 970, 766.

Mp: 103 °C.

HRMS : calculated for $C_{23}H_{29}O_3N_2BSNa$ $[M + Na]^+$ 447.1884, found 447.1876

Specific rotation: $[\alpha]_D^{21}$ +110.87 (c = 0.6, $CHCl_3$).

Enantiomeric purity of **2n** was determined by HPLC analysis in comparison with authentic racemic material (er = 88:12; **IA** column, 90:10 hexanes: *i*PrOH, 0.5 mL/min, 20 °C, 254 nm).

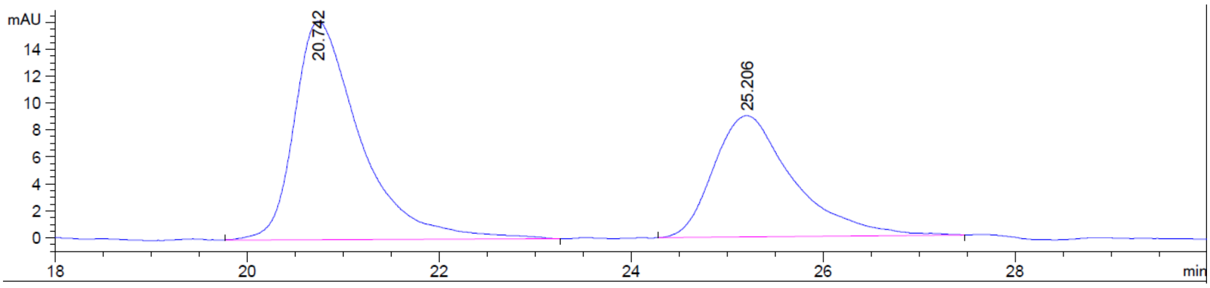

Peak #	RetTime [min]	Type	Width [min]	Area [mAU*s]	Height [mAU]	Area %
1	20.742	BB	0.6822	773.30768	16.22099	59.7200
2	25.206	BB	0.7355	521.58051	9.00788	40.2800

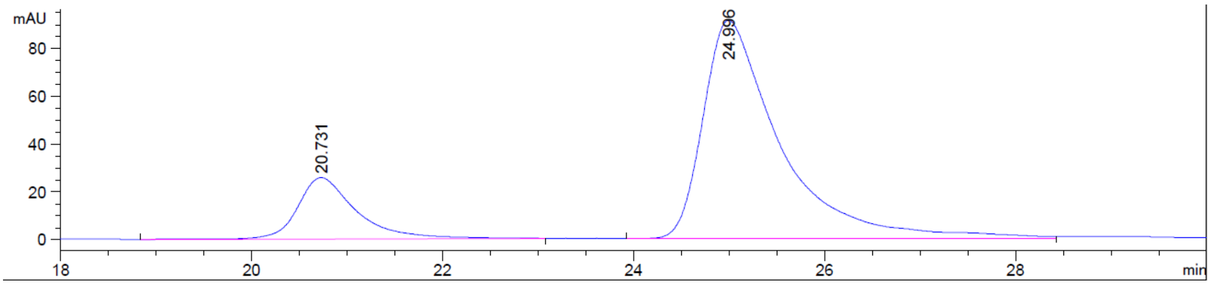

Peak #	RetTime [min]	Type	Width [min]	Area [mAU*s]	Height [mAU]	Area %
1	20.726	BB	0.6428	2801.42578	62.27605	12.4269
2	24.995	BB	0.7632	1.97419e4	375.60776	87.5731

^{1}H NMR of 2n (400 MHz, $CDCl_3$)

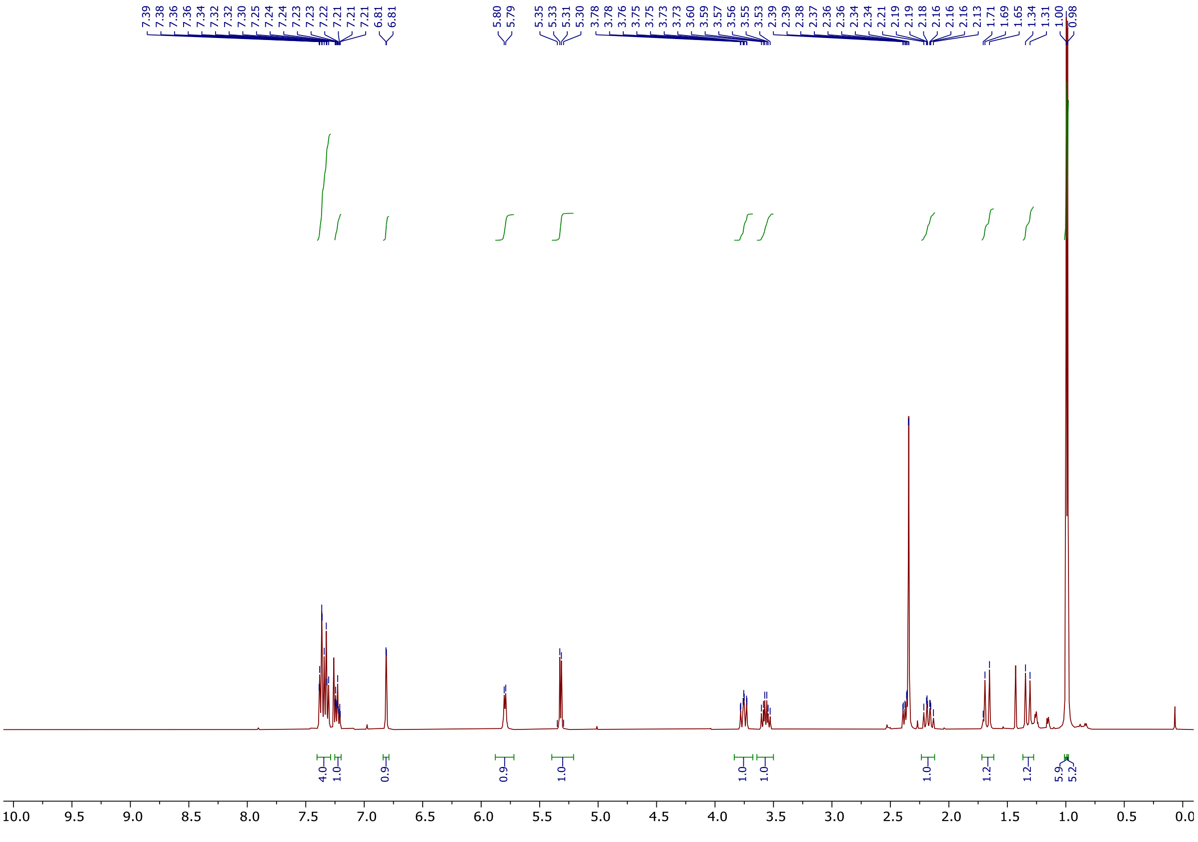

^{13}C NMR of 2n (101 MHz, CDCl$_3$)

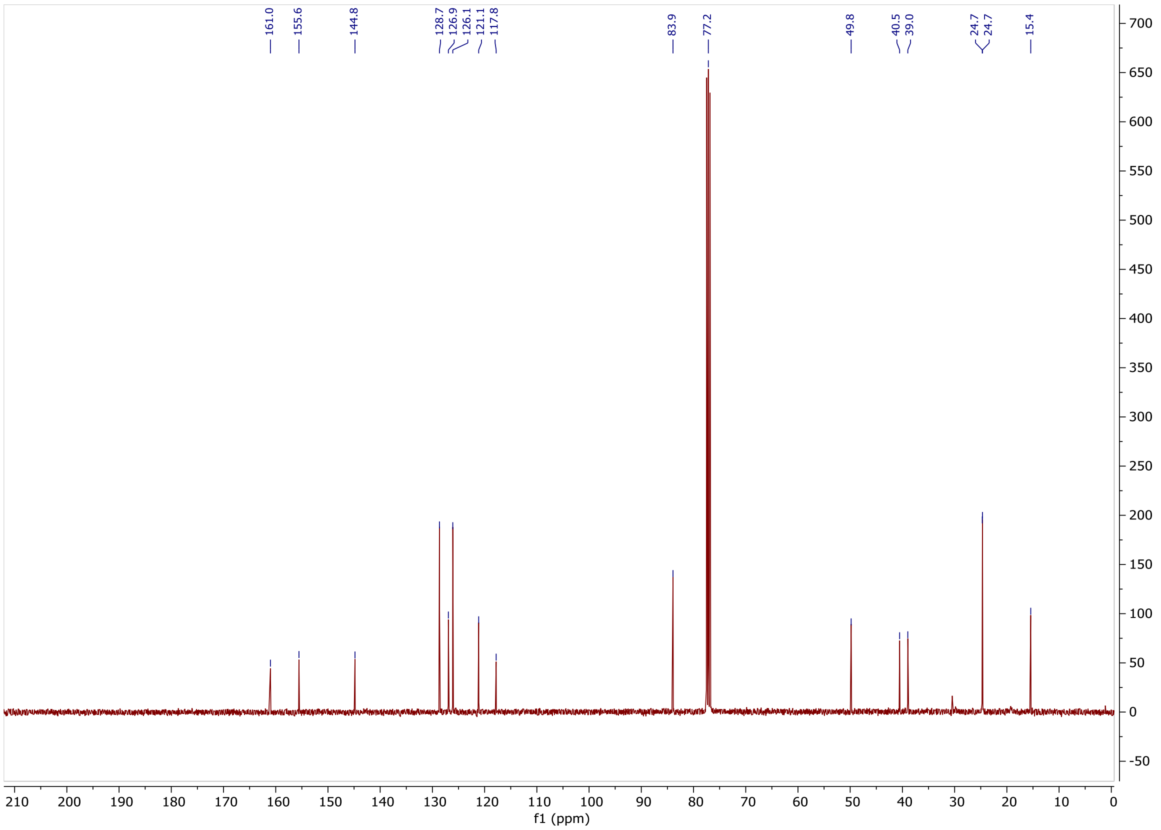

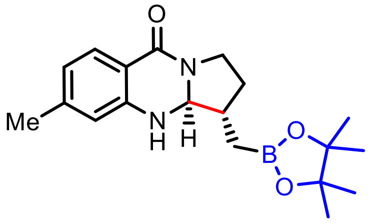

Chemical Formula: $C_{19}H_{27}BN_2O_3$
Molecular Weight: 342,25

(3*S*,3a*S*)-7-Methyl-3-((4,4,5,5-tetramethyl-1,3,2-dioxaborolan-2-yl)methyl)-2,3,3a,4-tetrahydropyrrolo[2,1-*b*]quinazolin-9(1*H*)-one (2o)

Compound **2o** was prepared according to General Procedure D, running the reaction at 25 °C. The title compound was isolated by column chromatography (hexane: ethyl acetate, 80:20 - 50:50) as a white solid (52.0 mg, 0.152 mmol, 76%, dr >95:5).

^{1}H NMR (400 MHz, CDCl$_3$) δ ppm 7.74 (d, *J* = 7.9 Hz, 1H, ArC*H*), 6.63 (d, *J* = 7.9 Hz, 1H, ArC*H*), 6.38 (s, 1H, ArC*H*), 5.64 (br, 1H, N*H*), 4.52 (d, *J* = 8.1 Hz, 1H, NHC*H*), 3.79 – 3.66 (m, 1H, NC*H$_2$*), 3.64 – 3.50 (m, 1H, NC*H$_2$*), 2.40 – 2.31 (m, 1H, NHCHC*H*), 2.27 (s, 3H, C*H$_3$*), 2.23 – 2.10 (m, 1H, NCH$_2$C*H$_2$*), 1.68 – 1.52 (m, 1H, NCH$_2$C*H$_2$*), 1.30 (s, 12H, C*H$_3$* x 4), 1.14 (dd, *J* = 16.5, 4.7 Hz, 1H, BC*H$_2$*), 0.89 (dd, *J* = 16.5, 9.9 Hz, 1H, BC*H$_2$*).

^{13}C NMR (101 MHz, $CDCl_3$) δ ppm 162.6 (*C*ON), 147.9 (Ar*C*), 143.6 (Ar*C*), 128.3 (Ar*C*H), 120.5 (Ar*C*H), 115.7 (Ar*C*), 114.7 (Ar*C*H), 84.0 (*C*q x 2), 76.1 (NH*C*H), 43.0 (N*C*H_2), 41.7 (NCH_2*C*H_2), 31.5 (NHCH*C*H), 25.1 (*C*H_3 x 2), 25.0 (*C*H_3), 21.8 (*C*H_3 x 2). The signal for B*C*H_2 was not observed.

^{11}B NMR (162 MHz, $CDCl_3$) δ ppm 33.34.

IR ν_{max} (neat/cm^{-1}): 3339, 3294, 2924, 2854, 1634, 1619, 1482, 1378, 1320, 1269, 1143, 847.

Mp: 228°C.

HRMS : calculated for $C_{19}H_{28}N_2O_3B$ $[M + H]^+$ 343.2187, found 343.2190.

Specific rotation: $[\alpha]_D^{24}$ + 15.04 (c 0.23, $CHCl_3$).

Enantiomeric purity of **2o** was determined by HPLC analysis in comparison with authentic racemic material (er = 97:3; **AI** column, 90:10 hexanes: *i*PrOH, 0.5 mL/min, 20 °C, 254 nm).

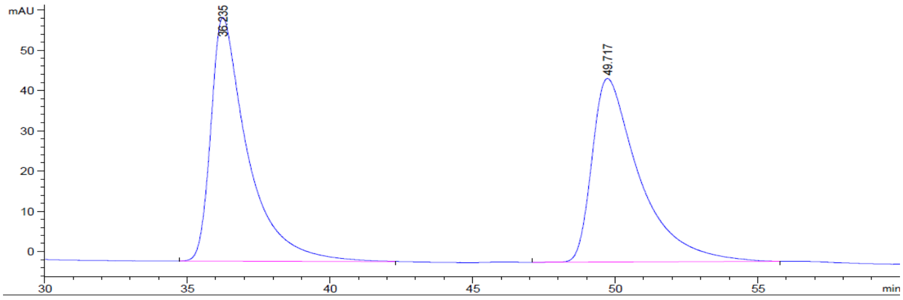

Peak #	RetTime [min]	Type	Width [min]	Area [mAU*s]	Height [mAU]	Area %
1	36.235	BB	1.2803	5436.07959	60.55727	50.7202
2	49.717	VB	1.6842	5281.71045	45.57081	49.2798

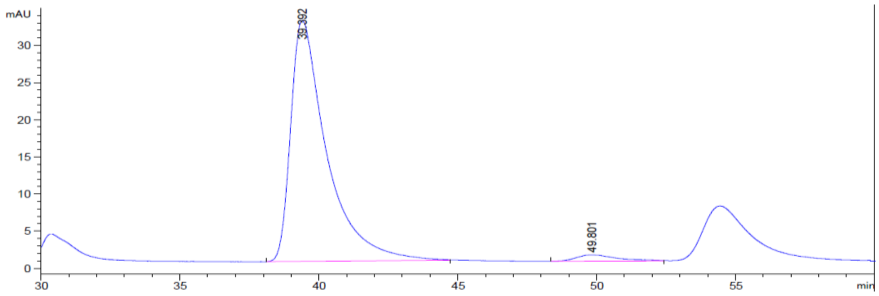

Peak #	RetTime [min]	Type	Width [min]	Area [mAU*s]	Height [mAU]	Area %
1	39.392	BB	1.2964	2923.99146	32.43238	97.1440
2	49.801	BB	1.1994	85.96486	8.48542e-1	2.8560

^{1}H NMR of 2o (400 MHz, $CDCl_3$)

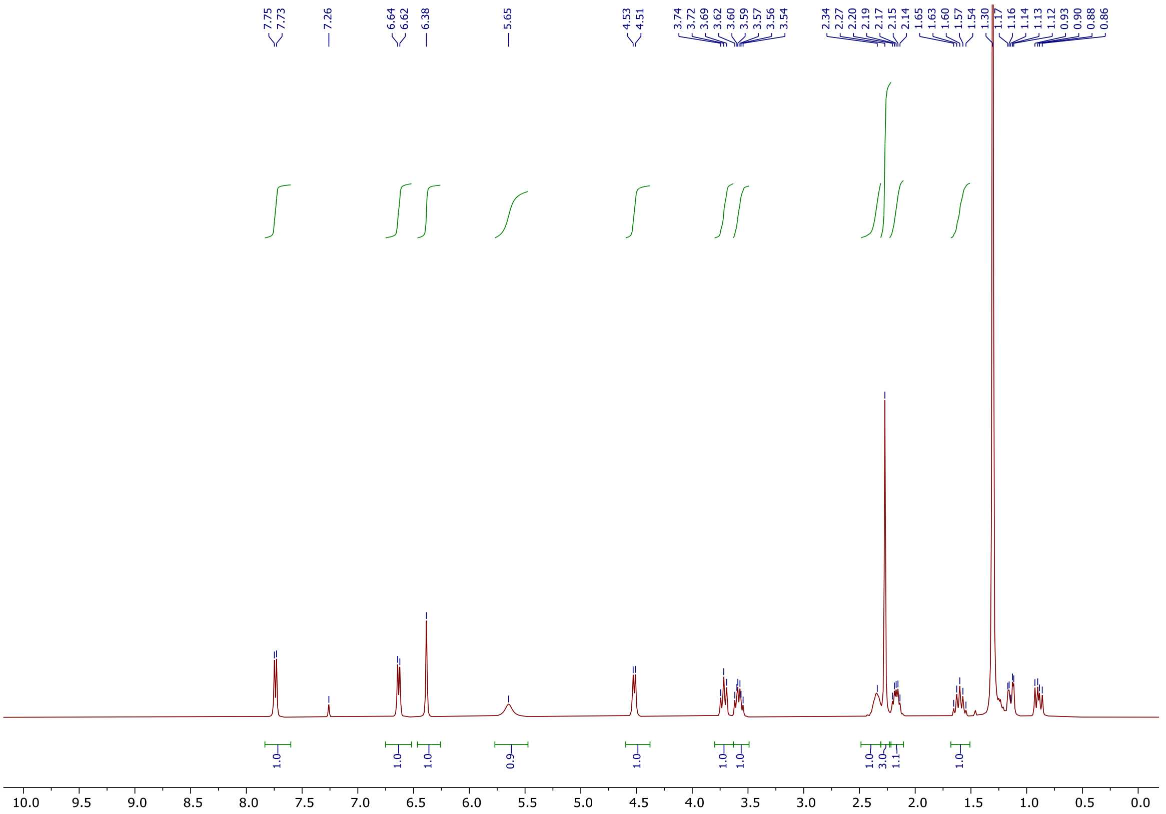

^{13}C NMR of 2o (101 MHz, $CDCl_3$)

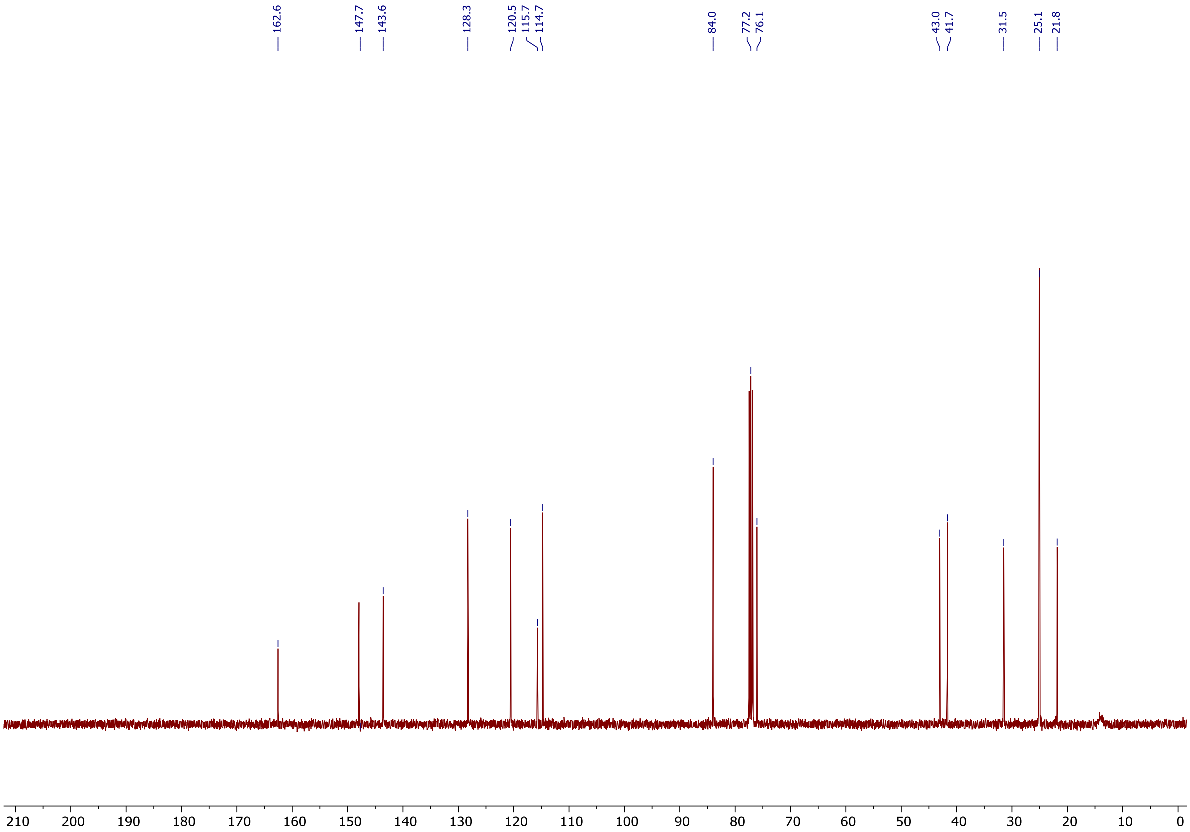

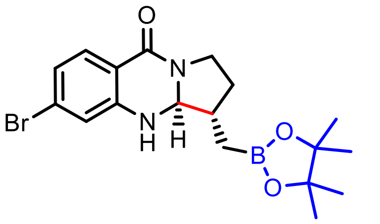

Chemical Formula: $C_{18}H_{24}BBrN_2O_3$
Molecular Weight: 407,12

(3*S*,3a*S*)-7-Bromo-3-((4,4,5,5-tetramethyl-1,3,2-dioxaborolan-2-yl)methyl)-2,3,3a,4-tetrahydropyrrolo[2,1-*b*]quinazolin-9(1*H*)-one (2p)

Compound **2p** was prepared according to General Procedure D, running the reaction at 25 °C. The title compound was isolated by column chromatography (hexane: ethyl acetate, 80:20 - 50:50) as a white solid (43.2 mg, 0.106 mmol, 53%, dr 95:5).

^{1}H NMR (400 MHz, CD_2Cl_2) δ ppm 7.63 (d, *J* = 8.3 Hz, 1H, ArCH), 6.91 (dd, *J* = 8.3, 1.8 Hz, 1H, ArCH), 6.77 (d, *J* = 1.8 Hz, 1H, ArCH), 6.02 (s, 1H, NH), 4.55 (dd, *J* = 8.2, 2.4 Hz, 1H, NHC*H*), 3.65 (ddd, *J* = 11.5, 9.4, 1.8 Hz, 1H, NCH_2), 3.53 (ddd, *J* = 12.0, 10.4, 7.4 Hz, 1H, NCH_2), 2.33 (tdd, *J* = 11.4, 9.2, 5.6 Hz, 1H, NHCHC*H*), 2.18 (dtd, *J* = 12.7, 7.2, 1.8 Hz, 1H, NCH_2CH_2), 1.61 (dddd, *J* = 12.9, 11.7, 10.4, 9.4 Hz, 1H, NCH_2CH_2), 1.31 (s, 6H, CH_3 x 2), 1.30 (s, 6H, CH_3 x 2), 1.16 (dd, *J* = 16.9, 4.7 Hz, 1H, BCH_2), 0.88 (dd, *J* = 16.6, 10.3 Hz, 1H, BCH_2).

^{13}C NMR (101 MHz, CD_2Cl_2) δ ppm 161.3 (CON), 148.5 (ArC), 129.4 (ArCH), 126.9 (ArC), 121.9 (ArCH), 119.9 (ArC), 116.9 (ArCH), 84.7 (Cq x 2), 77.2 (NHCH), 43.0 (NCH_2), 41.6 (NCH_2CH_2), 31.4 (NHCH*C*H), 24.8 (CH_3 x 2), 24.7 (CH_3 x 2). The signal for BCH_2 was not observed.

^{11}B NMR (162 MHz, $CDCl_3$) δ ppm 30.28.

IR ν_{max} (neat/cm^{-1}): 3706, 3293, 2973, 2924, 1635, 1603, 1502, 1477, 1372, 1143, 1055, 847.

Mp: 224°C.

HRMS: calculated for $C_{18}H_{24}N_2O_3BBrNa$ $[M + Na]^+$ 429.0961, found 429.0956.

Specific rotation: $[\alpha]_D^{26}$ + 50.71 (c 0.14, $CHCl_3$).

Enantiomeric purity of **2p** was determined by HPLC analysis in comparison with authentic racemic material (er = 92:8; **AI** column, 95:5 hexanes: *i*PrOH, 1 mL/min, 20 °C, 254 nm).

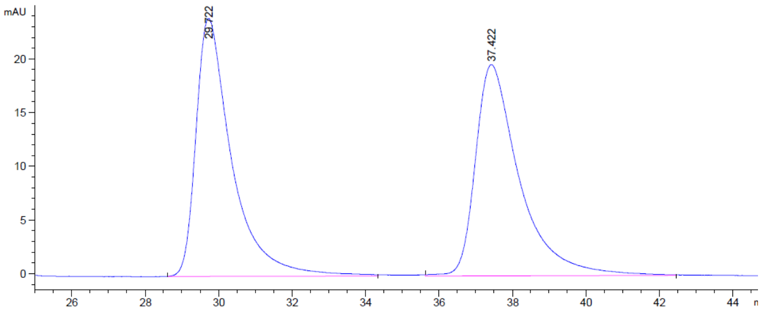

Peak #	RetTime [min]	Type	Width [min]	Area [mAU*s]	Height [mAU]	Area %
1	29.722	BB	0.9908	1631.38440	24.00791	49.8231
2	37.422	BB	1.2425	1642.96948	19.66765	50.1769

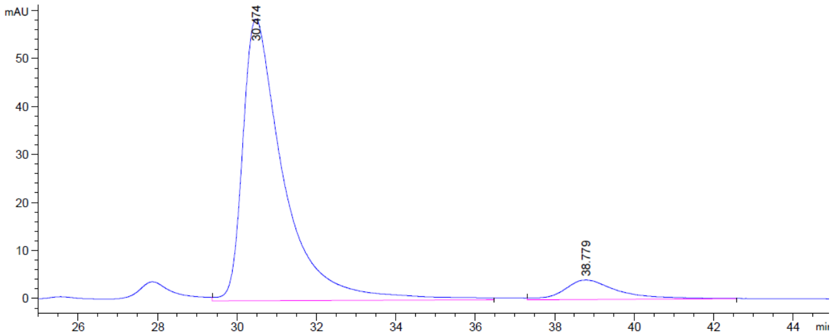

Peak #	RetTime [min]	Type	Width [min]	Area [mAU*s]	Height [mAU]	Area %
1	30.474	VB	1.0366	4216.83057	58.66883	91.6260
2	38.779	BB	1.3434	385.38846	4.04917	8.3740

^{1}H NMR of 2p (400 MHz, CD_2Cl_2)

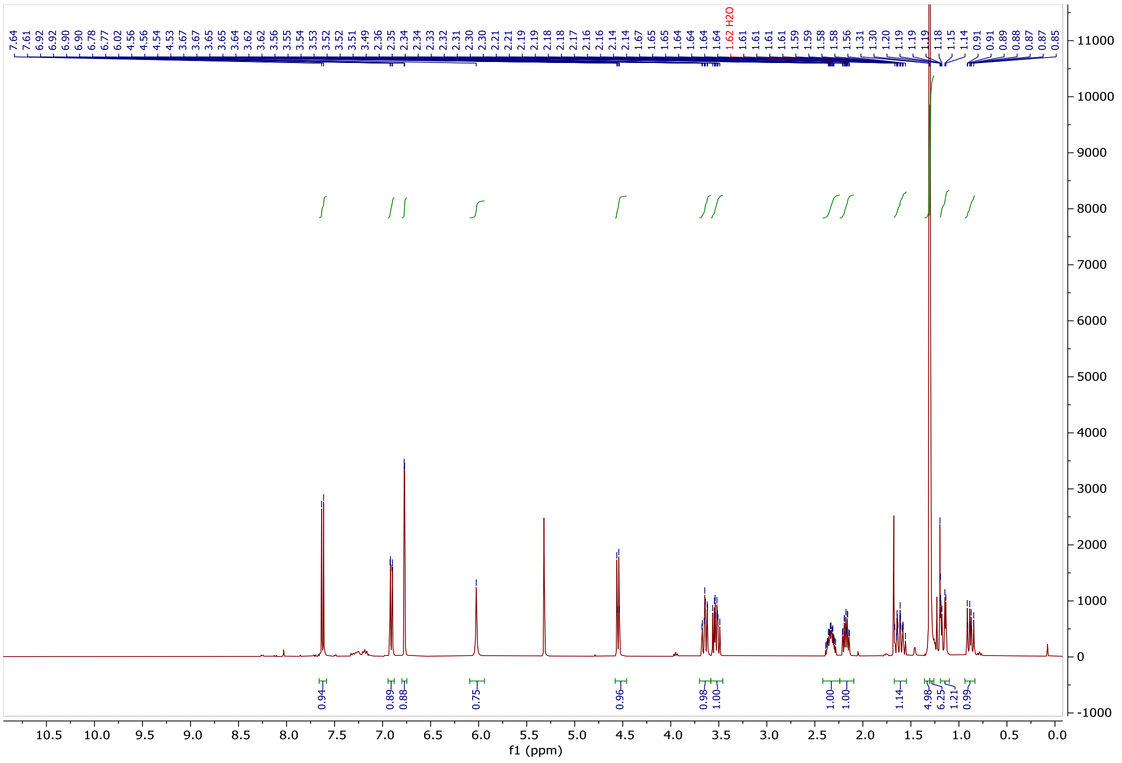

^{13}C NMR of 2p (101 MHz, CD_2Cl_2)

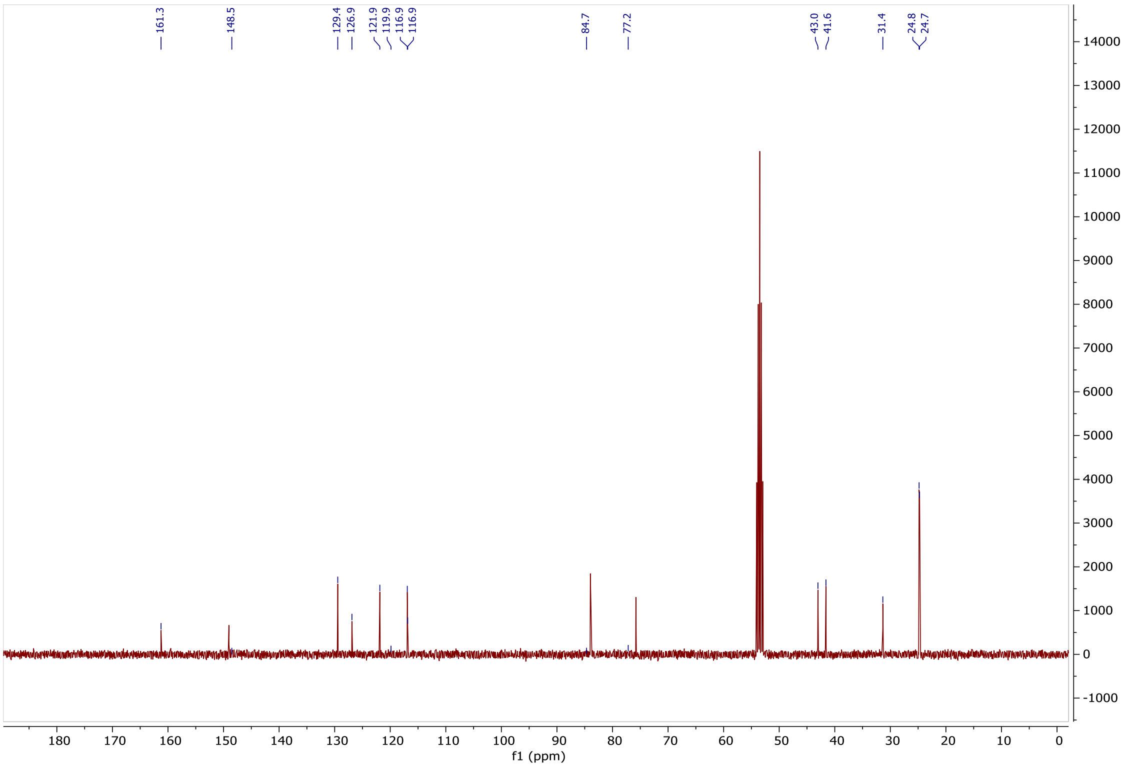

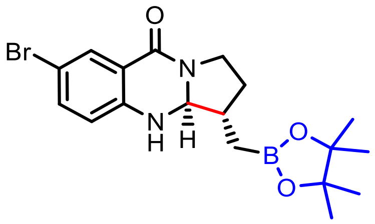

Chemical Formula: $C_{18}H_{24}BBrN_2O_3$
Molecular Weight: 407,12

(3*S*,3a*S*)-6-Bromo-3-((4,4,5,5-tetramethyl-1,3,2-dioxaborolan-2-yl)methyl)-2,3,3a,4-tetrahydropyrrolo[2,1-*b*]quinazolin-9(1*H*)-one (2q)

Compound **2q** was prepared according to General Procedure C, running the reaction at 25 °C. The title compound was isolated by column chromatography (hexane: ethyl acetate, 80:20 - 50:50) as a white solid (66.5 mg, 0.164 mmol, 82%, dr 95:5).

^{1}H NMR (400 MHz, $CDCl_3$) δ ppm 7.71 (d, *J* = 8.3 Hz, 1H, ArC*H*), 6.93 (dd, *J* = 8.3, 1.8 Hz, 1H, ArC*H*), 6.72 (d, *J* = 1.8 Hz, 1H, ArC*H*), 5.95 (s, 1H, N*H*), 4.56 (d, *J* = 8.2 Hz, 1H, NHC*H*), 3.78 – 3.71 (m, 1H, NC*H*$_2$), 3.68 – 3.54 (m, 1H, NC*H*$_2$), 2.35 (ddt, *J* = 11.3, 7.8, 3.6 Hz, 1H, NCH$_2$C*H*$_2$), 2.27 – 2.07 (m, 1H, NCH$_2$C*H*$_2$), 1.72 – 1.57 (m, 1H, NHCHC*H*), 1.32 (s, 6H, C*H*$_3$ x 2), 1.32 (s, 6H, C*H*$_3$ x 2), 1.19 (dd, *J* = 16.7, 4.1 Hz, 1H, BC*H*$_2$), 0.88 (dd, *J* = 16.6, 10.5 Hz, 1H, BC*H*$_2$).

^{13}C NMR (101 MHz, CDCl$_3$) δ ppm 161.8 (CON), 148.8 (ArC), 129.9 (ArCH), 127.3 (ArCH), 122.4 (ArCH), 117.0 (ArC), 116.9 (ArC), 84.2 (Cq x 2), 75.9 (NHCH), 43.2 (NCH$_2$), 41.7 (NCH$_2$*C*H$_2$), 31.6 (NHCH*C*H), 25.2 (*C*H$_3$ x 2), 25.1(CH$_3$ x 2). The signal for CH$_2$Bpin was not observed.

^{11}B NMR (162 MHz, CDCl$_3$) δ ppm 33.46.

IR ν_{max} (neat/cm^{-1}): 3299, 2975, 1629, 1601, 1370, 1358, 1143, 1110, 847, 762.

Mp: 228°C.

HRMS : calculated for $C_{18}H_{25}N_2O_3BBr$ [M + H]$^+$ 407.1130, found 407.1136.

Specific rotation: $[\alpha]_D^{27}$ + 80.89 (c 0.09, CHCl$_3$).

Enantiomeric purity of **2q** was determined by HPLC analysis in comparison with authentic racemic material (er = 82:18; **AI** column, 95:5 hexanes: *i*PrOH, 1 mL/min, 20 °C, 254 nm).

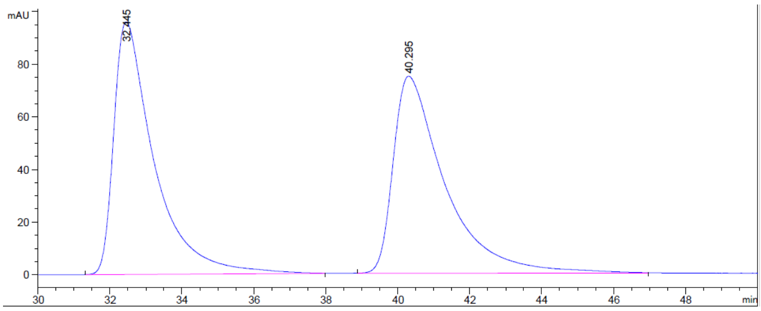

Peak #	RetTime [min]	Type	Width [min]	Area [mAU*s]	Height [mAU]	Area %
1	32.445	BB	1.1121	7470.01807	95.95779	49.9456
2	40.295	BB	1.4400	7486.27734	74.89079	50.0544

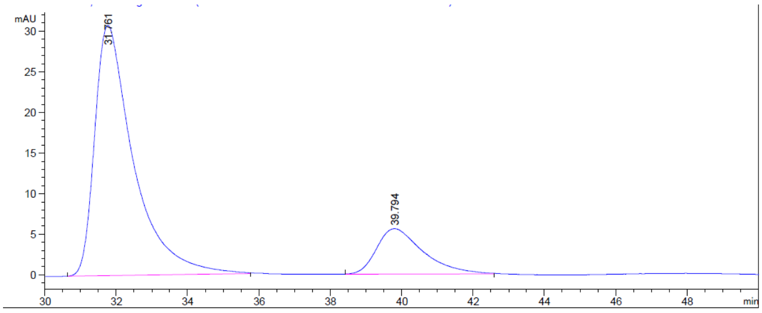

Peak #	RetTime [min]	Type	Width [min]	Area [mAU*s]	Height [mAU]	Area %
1	31.761	BB	1.1222	2325.59424	30.81568	82.1350
2	39.794	BB	1.2762	505.83582	5.59450	17.8650

^{1}H NMR of 2q (400 MHz, $CDCl_3$)

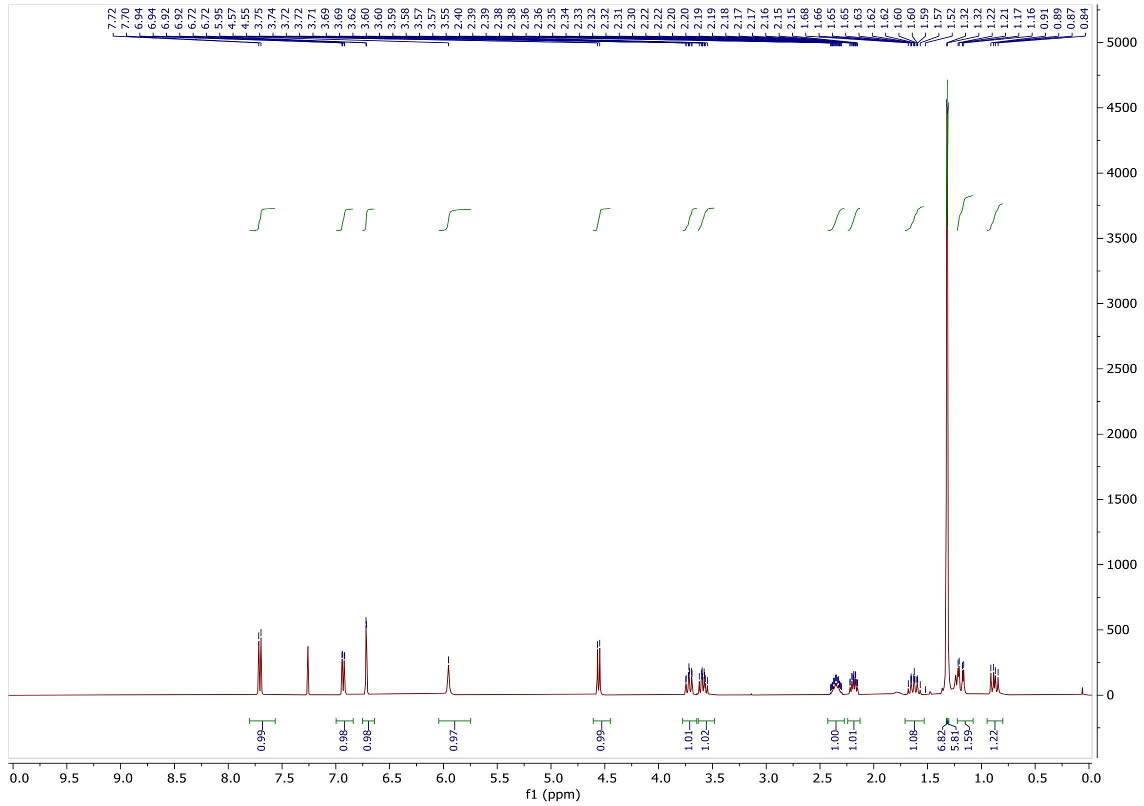

^{13}C NMR of 2q (101 MHz, $CDCl_3$)

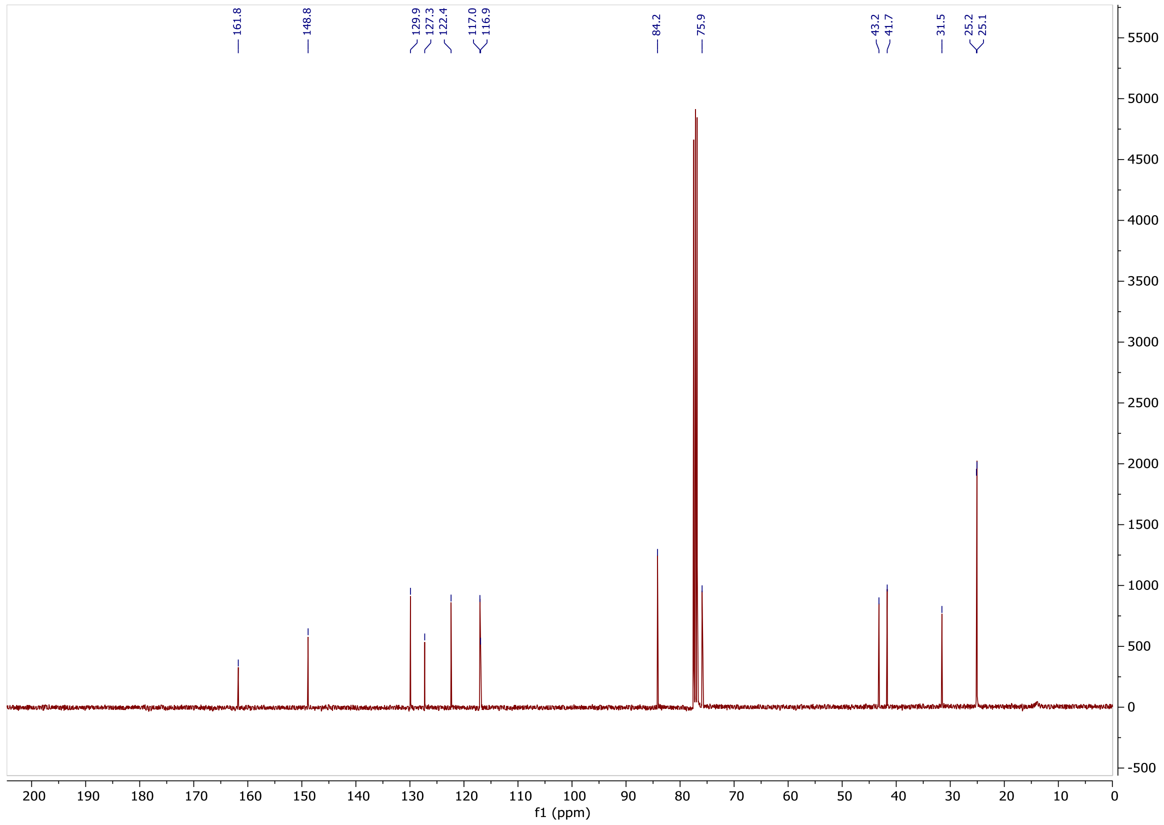

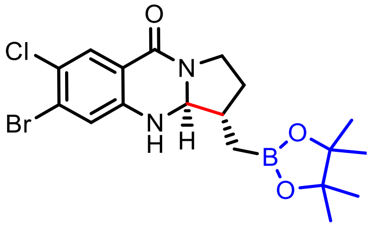

Chemical Formula: $C_{18}H_{23}BBrClN_2O_3$
Molecular Weight: 441,56

(3*S*,3a*S*)-6-Bromo-7-chloro-3-((4,4,5,5-tetramethyl-1,3,2-dioxaborolan-2-yl)methyl)-2,3,3a,4-tetrahydropyrrolo[2,1-*b*]quinazolin-9(1*H*)-one (2r)

Compound **2r** was prepared according to General Procedure D, running the reaction at 25 °C. The title compound was isolated by column chromatography (hexane: ethyl acetate, 80:20 - 50:50) as a white solid (76.6 mg, 0.174 mmol, 87%, dr >95:5).

^{1}H NMR (500 MHz, $CDCl_3$) δ ppm 7.88 (s, 1H, ArC*H*), 6.83 (s, 1H, ArC*H*), 5.99 (s, 1H, N*H*), 4.54 (d, *J* = 8.2 Hz, 1H, NHC*H*), 3.71 (ddd, *J* = 11.4, 9.3, 1.7 Hz, 1H, NCH_2), 3.58 (ddd, *J* = 12.1, 10.5, 7.4 Hz, 1H, NCH_2), 2.40 – 2.28 (m, 1H, NCH_2CH_2), 2.24 – 2.14 (m, 1H, NCH_2CH_2), 1.69 – 1.56 (m, 1H, NHCHC*H*), 1.32 (s, 6H, CH_3 x 2), 1.31 (s, 6H, CH_3 x 2), 1.19 (dd, *J* = 16.6, 4.2 Hz, 1H, BCH_2), 0.88 (dd, *J* = 16.6, 10.5 Hz, 1H, BCH_2).

^{13}C NMR (126 MHz, $CDCl_3$) δ 160.7 (*C*ON), 146.8 (Ar*C*), 129.5 (Ar*C*H), 127.0 (Ar*C*), 124.5 (Ar*C*), 119.1 (Ar*C*), 118.5 (Ar*C*H), 84.2 (*C*q x 2), 75.9 (N*C*H), 43.3 (NCH_2), 41.6 (NCH_2CH_2), 31.5 (NHCH*C*H), 25.2 (CH_3 x 2), 25.1 (CH_3 x 2). The signal for CH_2Bpin was not observed.

^{11}B NMR (160 MHz, $CDCl_3$) δ ppm 33.10.

IR ν_{max} (neat/cm^{-1}): 3313, 2974, 2924, 1636, 1444, 1371, 1321, 1230, 1145, 847.

Mp: 211 °C.

HRMS: calculated for $C_{18}H_{23}N_2O_3BNa$ $[M + Na]^+$ 441.0738, found 441.0746.

Specific rotation: $[\alpha]_D^{23}$ + 42.13 (c 0.15, $CHCl_3$).

Enantiomeric purity of **2r** was determined by HPLC analysis in comparison with authentic racemic material (er = 85:15; **IA** column, 95:5 hexanes: *i*PrOH, 0.5 mL/min, 20 °C, 254 nm).

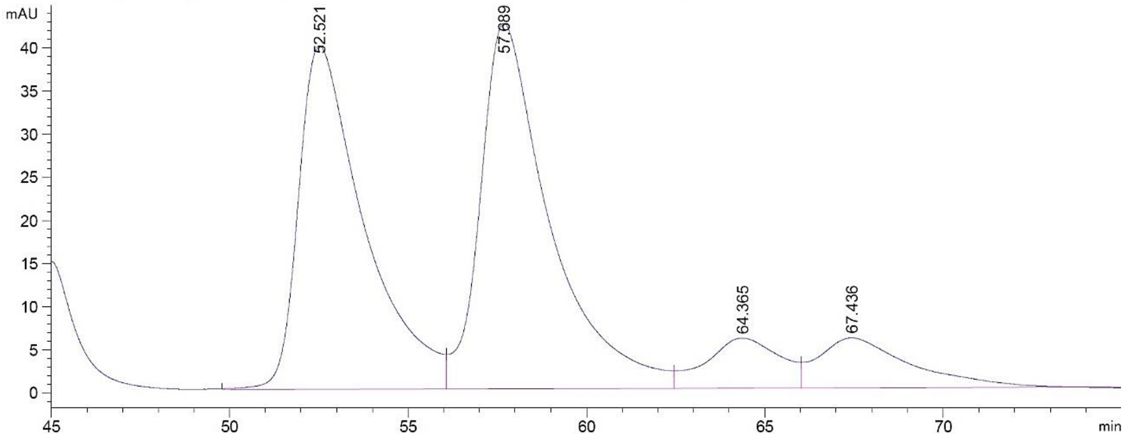

Peak #	RetTime [min]	Type	Width [min]	Area [mAU*s]	Height [mAU]	Area %
2	52.521	BV	1.8416	5090.30420	39.79944	39.9933
3	57.689	VV	1.9500	5726.72510	42.35927	44.9935
4	64.365	VV	2.0790	838.39343	5.81186	6.5871
5	67.436	VB	2.5601	1047.04797	5.80091	8.2264

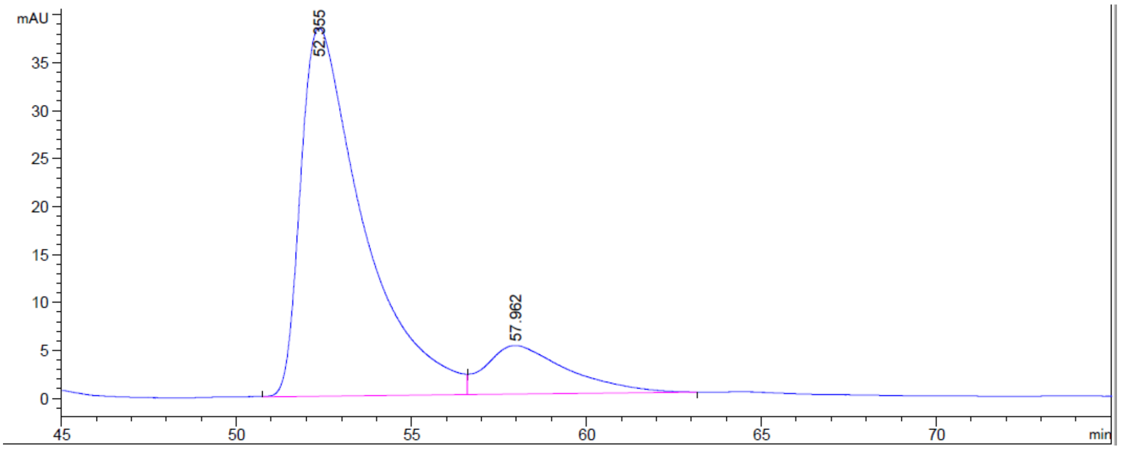

Peak #	RetTime [min]	Type	Width [min]	Area [mAU*s]	Height [mAU]	Area %
1	52.355	BV	1.8020	4781.12451	38.40094	85.2568
2	57.962	VB	2.3015	826.78583	5.06224	14.7432

^{1}H NMR of 2r (400 MHz, $CDCl_3$)

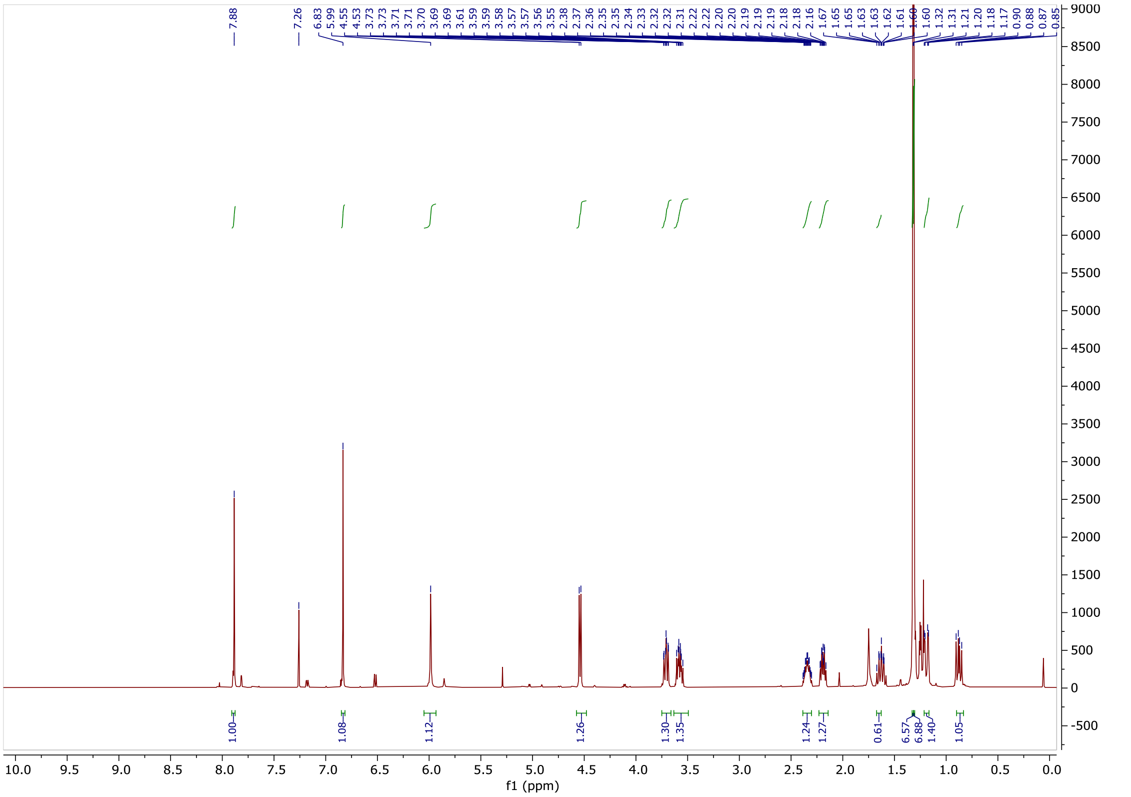

^{13}C NMR of 2r (101 MHz, $CDCl_3$)

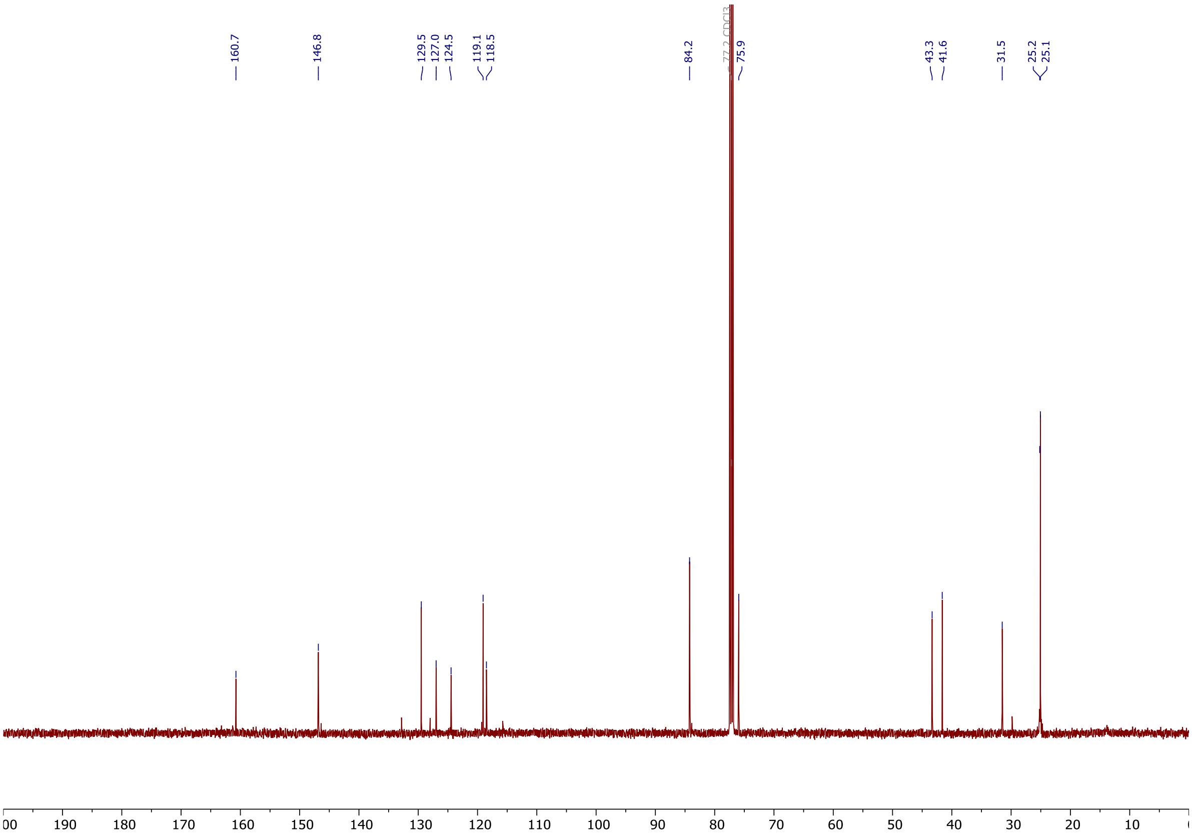

Data for gram-scale reaction and product manipulation

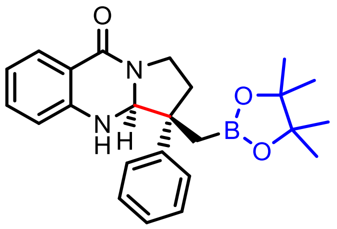

Chemical Formula: $C_{24}H_{29}BN_2O_3$
Molecular Weight: 404.3170

(3*S*,3a*S*)-3-Phenyl-3-((4,4,5,5-tetramethyl-1,3,2-dioxaborolan-2-yl)methyl)-2,3,3a,4-tetrahydropyrrolo[2,1-*b*]quinazolin-9(1H)-one (2b)

Compound **2b** was prepared according to General Procedure C, on a gram scale (1 g, 36.2 mmol) and running the reaction at 25 °C. The title compound was isolated by column chromatography (hexane : diethyl ether, 50:50 to 25:75) as a white solid (1.15 g, 28.4 mmol, 78%, dr 96:4).

HRMS : calculated for $C_{25}H_{31}O_3N_2BNa$ $[M + Na]^+$ 441.2320, found 441.2305.

Enantiomeric purity of **2b** was determined by HPLC analysis in comparison with authentic racemic material (er = 98:2; **IA** column, 90:10 hexanes: *i*PrOH, 0.5 mL/min, 20 °C, 254 nm)

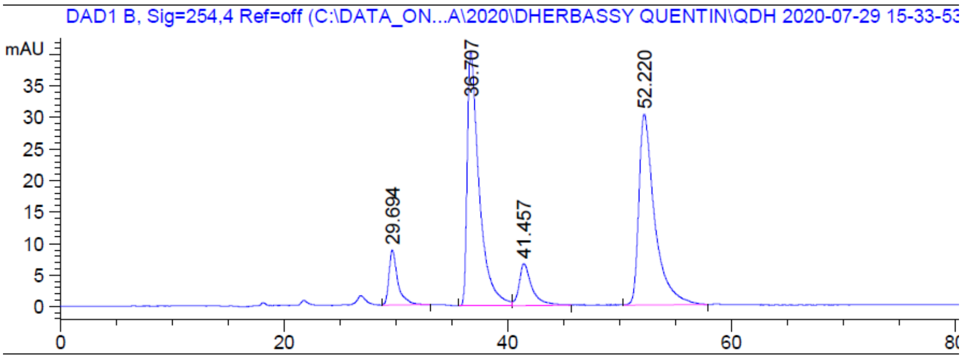

Peak #	RetTime [min]	Type	Width [min]	Area [mAU*s]	Height [mAU]	Area %
1	29.694	BB	0.8090	481.45813	8.70868	6.8810
2	36.707	BV	1.0793	3006.79590	40.44520	42.9734
3	41.457	VB	1.1106	521.38287	6.62154	7.4516
4	52.220	BB	1.4304	2987.24268	30.29498	42.6939

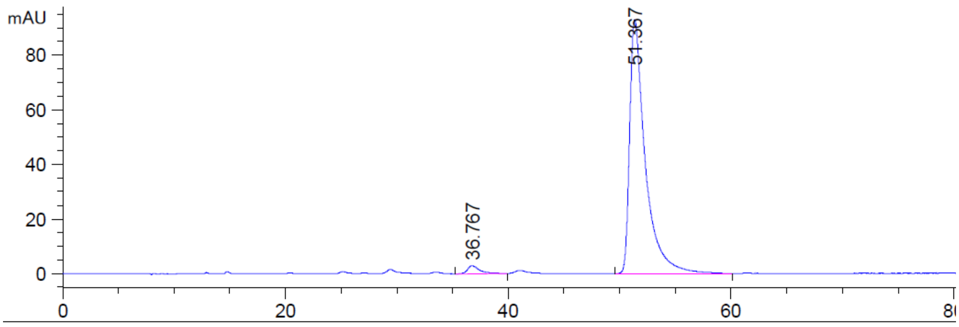

Peak #	RetTime [min]	Type	Width [min]	Area [mAU*s]	Height [mAU]	Area %
1	36.749	BB	0.8371	1234.40161	17.49669	2.2210
2	51.368	BB	1.3789	5.43441e4	560.64838	97.7790

^{1}H NMR of 2b obtained from the gram-scale reaction (400 MHz, $CDCl_3$)

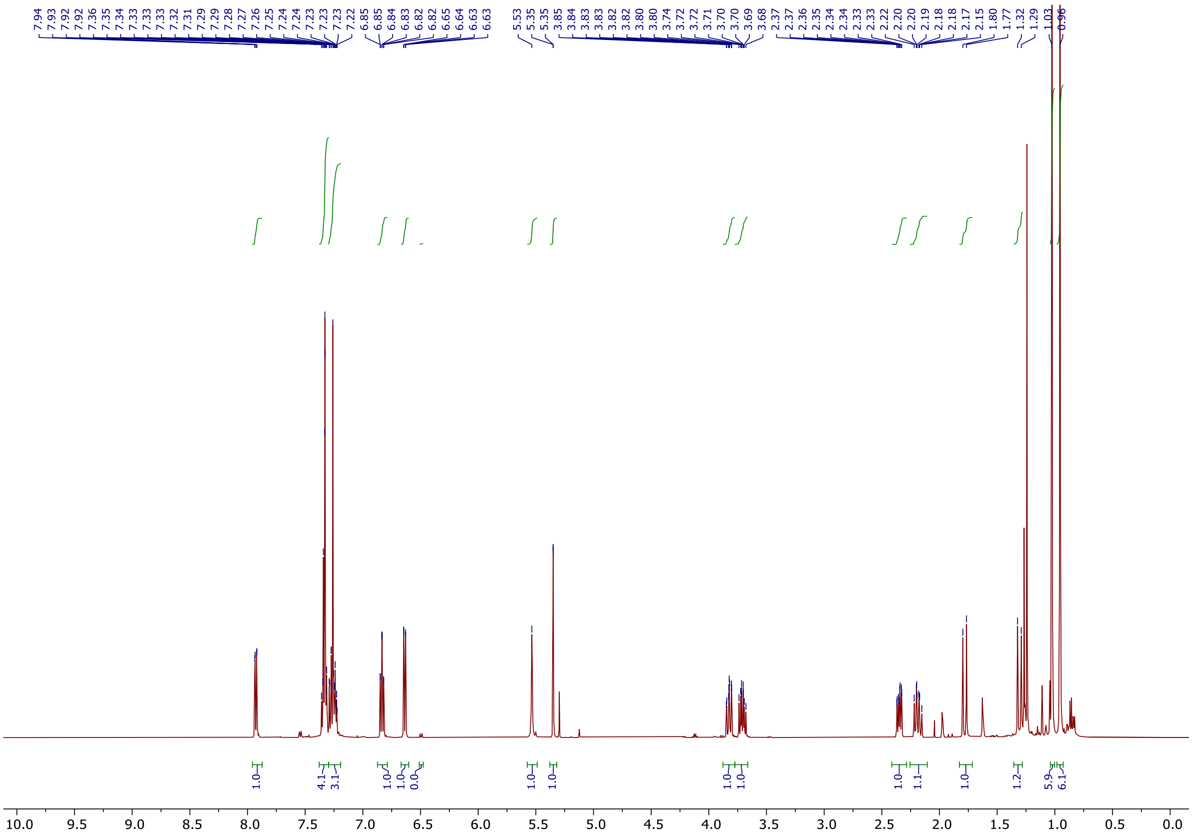

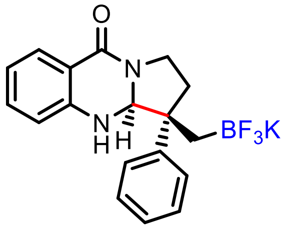

Chemical Formula: $C_{18}H_{17}BF_3KN_2O$
Molecular Weight: 384.25

(3*S*,3*aS*)-3-Phenyl-3-((trifluoro-λ4-boraneyl)methyl)-2,3,3a,4-tetrahydropyrrolo[2,1-*b*]quinazolin-9(1H)-one, potassium salt (3)

To a solution of **2b** (60 mg, 0.148 mmol, 1 equiv.) in MeOH (1.5 mL) at 0 °C was added dropwise a solution of KHF_2 (34.7 mg, 0.444 mmol, 3 equiv.) in H_2O (0.5 mL). The reaction mixture was then allowed to warm up to room temperature and stirred for 30 min. Solid KHF_2 (11.6 mg, 0.148 mmol, 1 equiv.) was added and the reaction mixture was stirred for 15 min to achieve complete conversion. The mixture was diluted with EtOH (10 mL) and the volatiles were removed under reduced pressure (this process was repeated twice) to give a solid residue. The product was extracted from the latter by exhaustively washing with anhydrous acetone (3 x 10 mL). The acetone solution was concentrated under reduced pressure (~10 mL) and the product was precipitated by addition of Et_2O. Crystallization of the crude product from an acetonitrile solution (10 mL), layered with a mixture of Et_2O/*n*-pentane (10 mL, 1:1 v/v), afforded the title compound as a white powder (42 mg, 0.109 mmol, 74% yield, dr >95:5).

^{1}H NMR (400 MHz, 400 MHz, CD_3CN) δ ppm 7.71 (dd, *J* = 7.7, 1.6 Hz, 1H, ArC*H*), 7.53 – 7.45 (m, 2H, ArC*H*), 7.32 – 7.21 (m, 3H, ArC*H* x 2), 7.15 (tt, *J* = 8.4, 1.2 Hz, 1H, ArC*H*), 6.84 – 6.73 (m, 2H, ArC*H* x 2), 5.42 (*br.* s, 1H, N*H*), 4.95 (d, *J* = 2.2 Hz, 1H, NC*H*), 3.66 – 3.50 (m, 2H, NCH_2), 2.51 (ddd, *J* = 12.2, 6.3, 2.9 Hz, 1H, NCH_2CH_2), 2.17 – 2.02 (m, 1H, NCH_2CH_2), 1.00 – 0.86 (m, 1H, CH_2BF_3K), 0.84 – 0.71 (m, 1H, CH_2BF_3K).

^{13}C NMR (101 MHz, CD_3CN) δ ppm 162.5 (CON), 148.7 (ArC), 147.3 (ArC), 133.0 (ArCH), 127.9 (ArCH x 2), 127.7 (ArCH), 127.4 (ArCH x 2), 125.8 (ArCH), 118.6 (ArCH), 118.1 (ArC), 116.0 (ArC), 78.3 (NHCH), 51.4 (Cq), 42.5 (NCH_2), 35.3 (NCH_2CH_2). The signal for BCH_2 was not observed.

^{11}B NMR (128 MHz, CD_3CN) δ ppm 4.38.

^{19}F NMR (376 MHz, CD_3CN) δ ppm -132.84.

IR ν_{max} (neat/cm^{-1}): 3396, 3057, 2891, 1637, 1496, 1485, 1441, 1073, 948, 700, 661.

Mp: 178 °C

HRMS : calculated for $C_{18}H_{17}O_1N_2BF_3$ $[M]^-$ 345.1395, found 345.1388.

Specific rotation: $[\alpha]_D^{20}$ +76.31 (c = 1.54, CH_3CN).

Enantiomeric purity for the title compound was determined by HPLC analysis, after derivatization of **3** to the corresponding *tert*-butyldimethylsilyl (TBDMS) ether (by sequential oxidation and *O*-protection), and in comparison with authentic racemic material (er = 92:8; **IA** column, 95:15 hexanes: *i*PrOH, 0.5 mL/min, 20 °C, 254 nm).

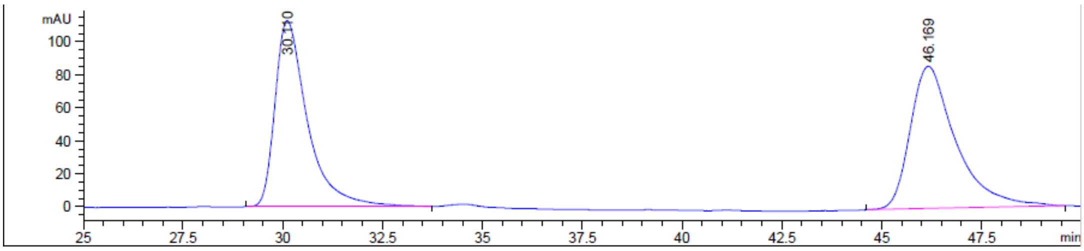

Peak #	RetTime [min]	Type	Width [min]	Area [mAU*s]	Height [mAU]	Area %
1	30.109	BB	0.7699	4808.53125	86.86121	48.7048
2	46.169	BB	0.9421	5064.28613	66.08083	51.2952

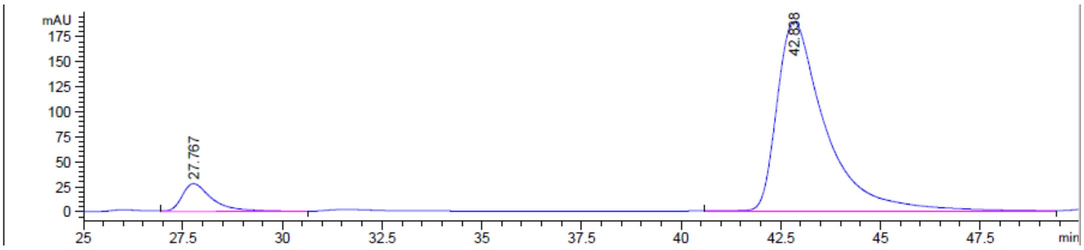

Peak #	RetTime [min]	Type	Width [min]	Area [mAU*s]	Height [mAU]	Area %
1	27.766	BB	0.7626	8864.04395	169.93329	8.4605
2	42.840	BB	1.2167	9.59055e4	1131.67676	91.5395

^{1}H NMR of 3 (400 MHz, CD_3CN)

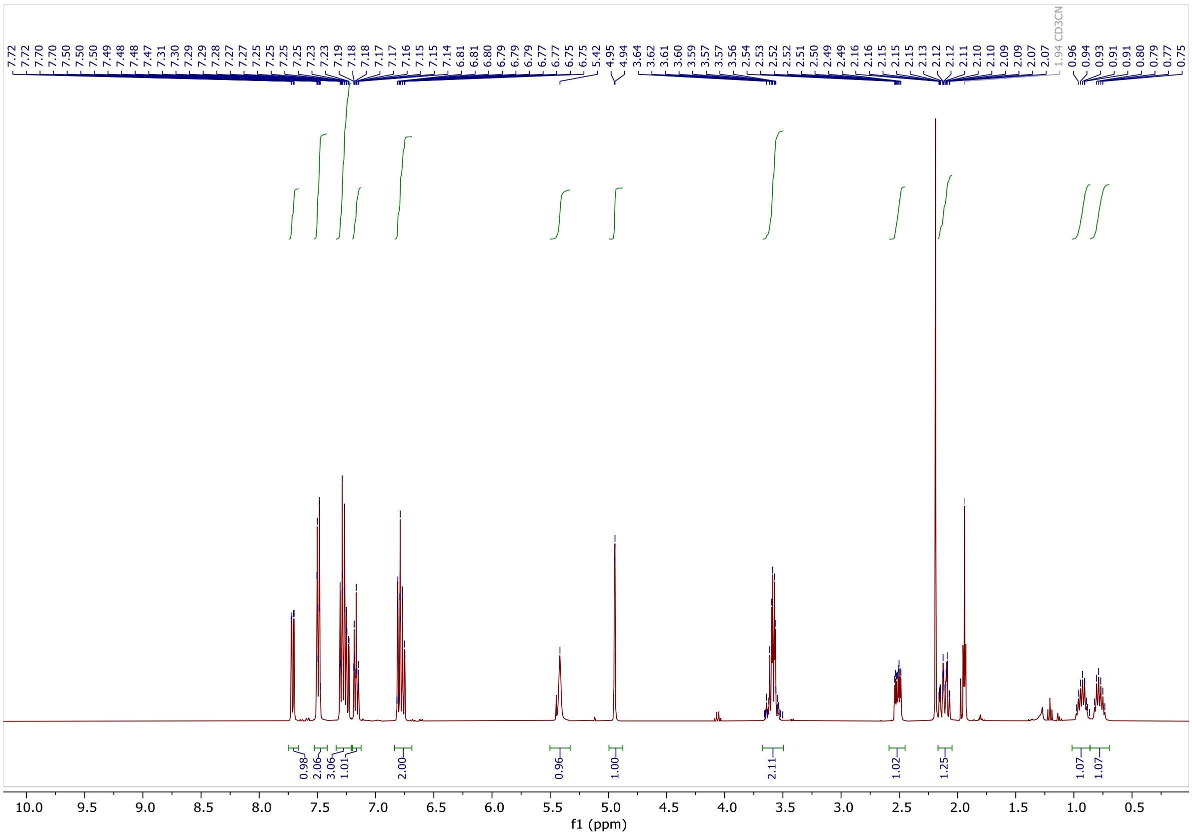

^{13}C NMR of 3 (101 MHz, CD_3CN)

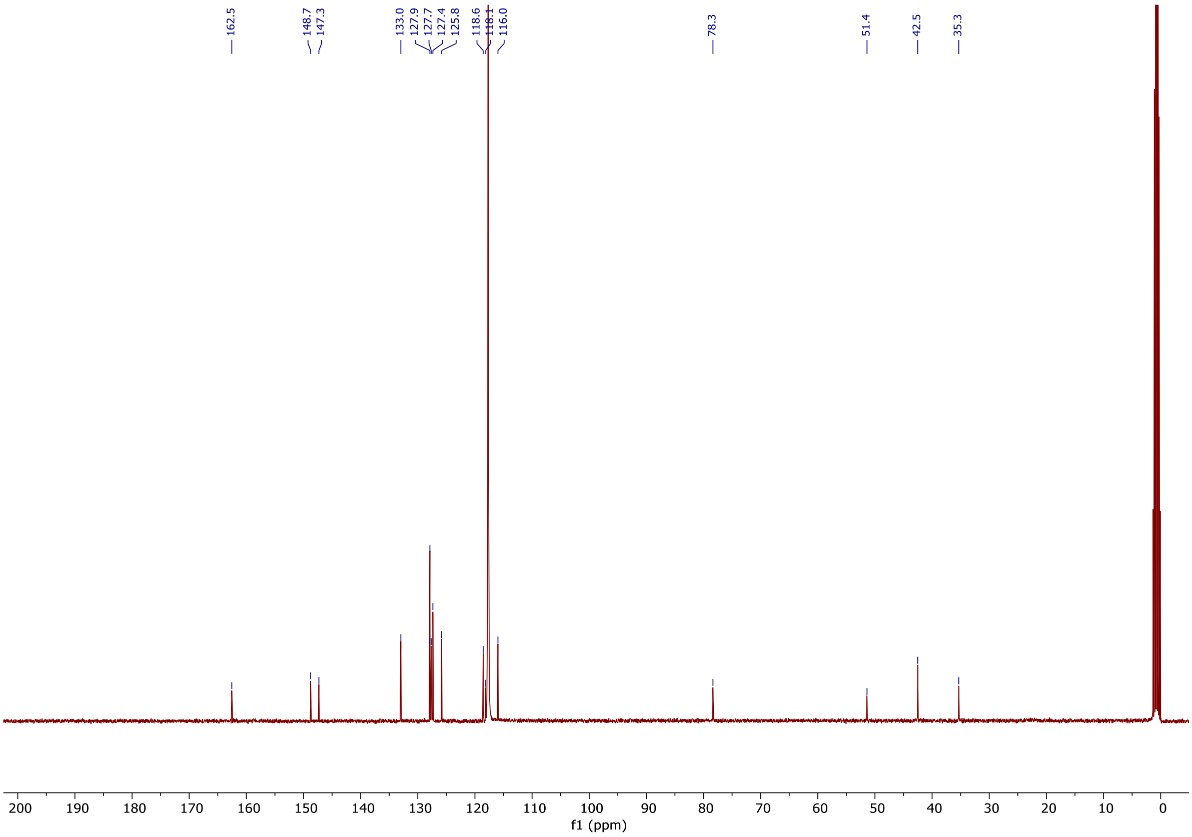

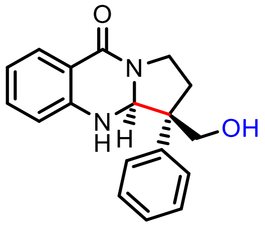

Chemical Formula: $C_{18}H_{18}N_2O_2$
Molecular Weight: 294.35

(3*R*,3*aS*)-3-(Hydroxymethyl)-3-phenyl-2,3,3a,4-tetrahydropyrrolo[2,1-*b*]quinazolin-9(1H)-one (4)

Boronic ester **2b** (44 mg, 0.109 mmol, 1 equiv) and K_2CO_3 (40 mg, 0.22 mmol, 2 equiv), were added to a round bottom flask, and the flask flushed with nitrogen. THF and H_2O_2 (0.1 mL, 2 equiv, 30% w/w) were then added and the mixture was stirred at -20 °C for 6 h. The mixture was diluted with EtOAc and quenched with aqueous saturated $Na_2S_2O_3$. The phases were separated, and the aqueous phase was extracted with EtOAc. The combined organic phases were dried, and the volatiles removed under reduced pressure. Purification of the crude material by column chromatography (EtOAc : Hexane 75:50 to 100:0) afforded the title compound as an amorphous white powder (28 mg, 0.095 mmol, 86%, dr >95:5).

^{1}H NMR (500 MHz, $CDCl_3$) δ ppm 7.93 (dd, *J* = 7.8, 1.6 Hz, 1H, ArC*H*), 7.44 – 7.38 (m, 4H, ArC*H* x 4), 7.35 – 7.29 (m, 2H, ArC*H* x 2), 6.93 (t, *J* = 7.5 Hz, 1H, ArC*H*), 6.81 (d, *J* = 8.0 Hz, 1H, ArC*H*), 5.36 (d, *J* = 2.8 Hz, 1H, NC*H*), 5.24 (*br.* s, 1H, N*H*), 4.23 (dd, *J* = 11.7, 7.8 Hz, 1H, C*H*$_2$OH), 3.89 (dd, *J* = 11.7, 4.8 Hz, 1H, C*H*$_2$OH), 3.82 – 3.67 (m, 2H, NC*H*$_2$), 2.37 (ddd, *J* = 13.3, 7.7, 2.4 Hz, 1H, NCH$_2$C*H*$_2$), 2.33 (*br.* s, 1H, O*H*), 2.26 – 2.15 (m, 1H, NCH$_2$C*H*$_2$).

^{13}C NMR (126 MHz, $CDCl_3$) δ ppm 163.0 (*C*ON), 146.6 (Ar*C*), 141.0 (Ar*C*), 133.3 (Ar*C*H), 129.3 (Ar*C*H x 2), 128.4 (Ar*C*H), 127.8 (Ar*C*H), 126.8 (Ar*C*H x 2), 120.6 (Ar*C*H), 118.7 (Ar*C*), 116.5 (Ar*C*H), 75.3 (N*C*H), 66.9 (*C*H$_2$OH), 53.7 (*C*q), 42.0 (N*C*H$_2$), 32.7 (NCH$_2$*C*H$_2$).

IR ν_{max} (neat/cm^{-1}): 3272, 2919, 1627, 1610, 1579, 1484, 1430, 1317, 1150, 1039, 976, 754, 735, 695.

Mp: decomp 175 °C

HRMS : calculated for $C_{19}H_{19}O_2N_2$ [M + H]$^+$ 295.1441, found 295.1439.

Specific rotation: $[\alpha]_D^{20}$ + 302.5° (c =0.69, $CHCl_3$).

Enantiomeric purity of **4** was determined by HPLC analysis in comparison with authentic racemic material after derivatization to the OTBDMS silyl ether (er = 98:2; **IA** column, 95:5 hexanes: *i*PrOH, 0.5 mL/min, 20 °C, 254 nm).

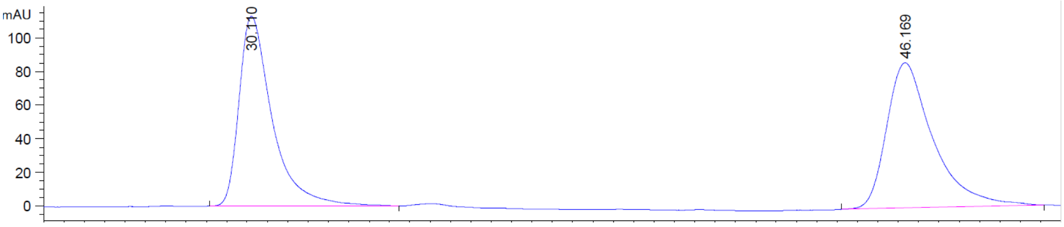

Peak #	RetTime [min]	Type	Width [min]	Area [mAU*s]	Height [mAU]	Area %
1	30.109	BB	0.7699	4808.53125	86.86121	48.7048
2	46.169	BB	0.9421	5064.28613	66.08083	51.2952

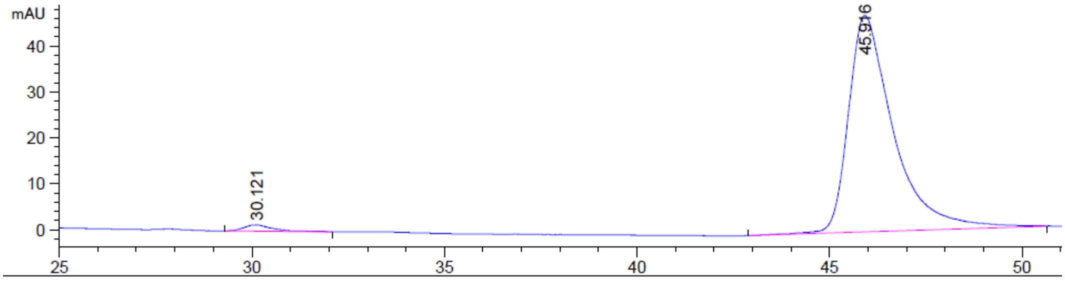

Peak #	RetTime [min]	Type	Width [min]	Area [mAU*s]	Height [mAU]	Area %
1	30.121	BB	0.6259	67.32597	1.32253	1.7344
2	45.916	BB	1.1623	3814.46069	47.02772	98.2656

^{1}H NMR of 4 (500 MHz, $CDCl_3$)

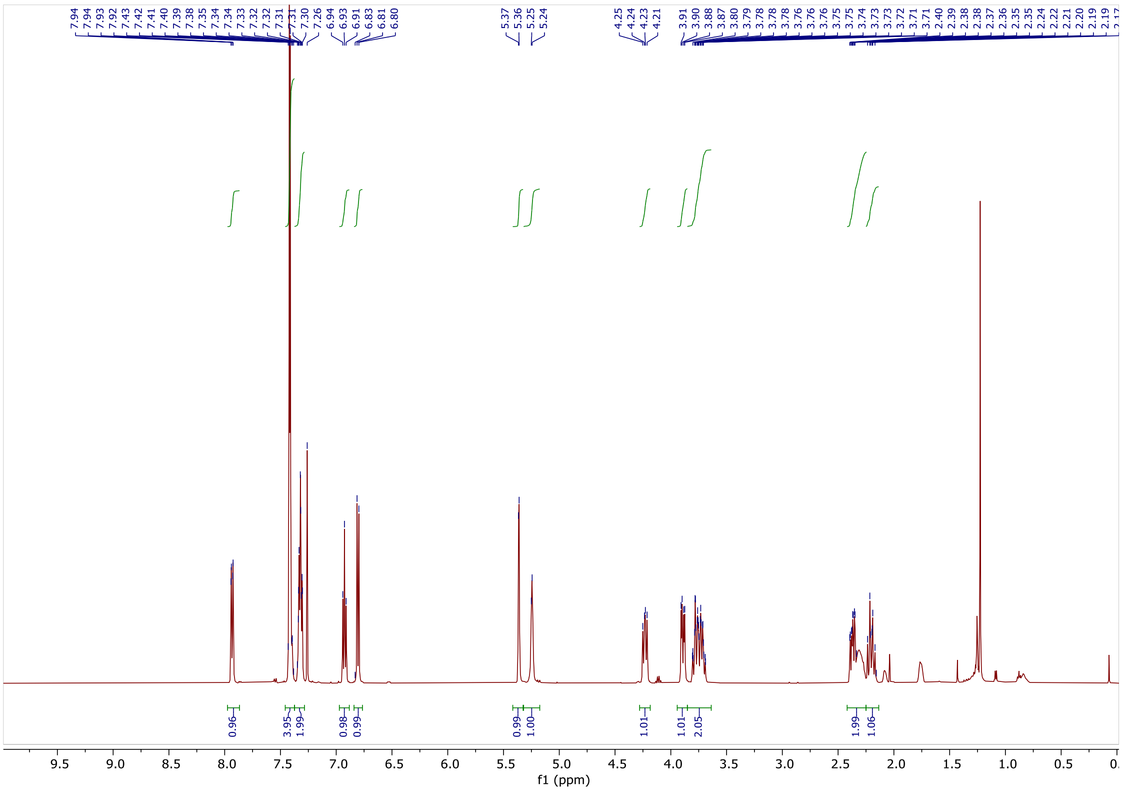

^{13}C NMR of 4 (126 MHz, $CDCl_3$)

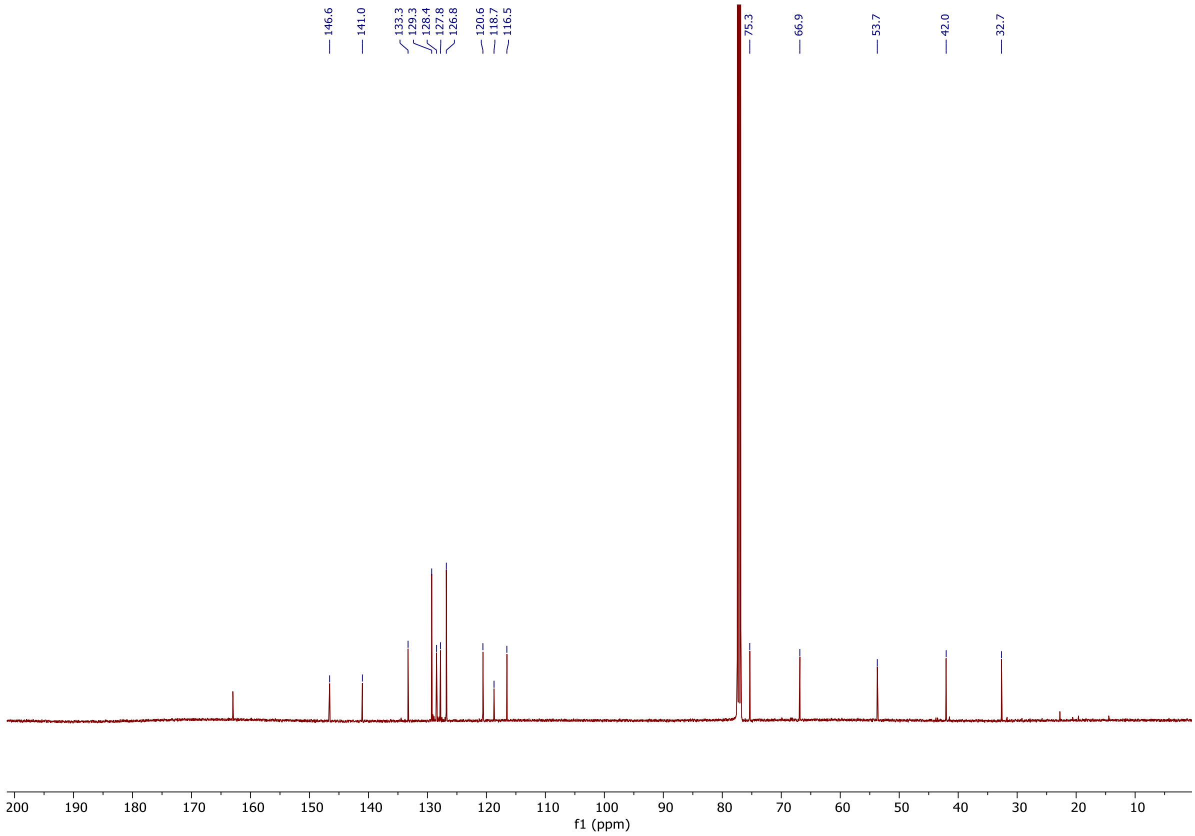

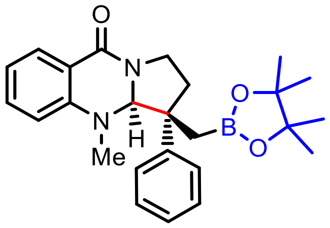

Chemical Formula: $C_{25}H_{31}BN_2O_3$

Molecular Weight: 418.34

(3*R*,3*aS*)-4-Methyl-3-phenyl-3-((4,4,5,5-tetramethyl-1,3,2-dioxaborolan-2-yl)methyl)-2,3,3a,4-tetrahydropyrrolo[2,1-*b*]quinazolin-9(1H)-one (5)

To a suspension of NaH (15 mg, 0.375 mmol, 1.5 equiv, 60% w/w dispersion in oil) in THF (1.5 mL) at 0 °C was added dropwise a solution of **2b** (100 mg, 0.247 mmol, 1 equiv) in THF (1.5 mL). The mixture was stirred 30 min at 0 °C, and MeI (53 mg, 0.373 mmol, 1.5 equiv) was added. The reaction mixture was allowed to warm up to room temperature and stirred overnight. The reaction was quenched with MeOH, diluted with Et_2O and filtered through a silica gel plug. Removal of the volatiles followed by chromatography (Et_2O: Hexane 20:50 to 75:25, neutralized with Et_3N) afforded the title compound as an amorphous off-white powder (60 mg, 0.143 mmol, 58%, dr >95:5).

^{1}H NMR (500 MHz, CDCl$_3$) δ ppm 7.94 (dd, *J* = 7.7, 1.7 Hz, 1H, ArCH), 7.48 (dd, *J* = 7.8, 2.0 Hz, 2H, ArCH), 7.37 (t, *J* = 7.8 Hz, 2H, ArCH x 2), 7.34 – 7.22 (m, 2H, ArCH x 2), 6.80 (td, *J* = 7.5, 1.0 Hz, 1H, ArCH), 6.56 (d, *J* = 8.3 Hz, 1H, ArCH), 5.14 (s, 1H, NCH$_3$C*H*), 3.92 (ddd, *J* = 12.4, 10.4, 7.9 Hz, 1H, NCH$_2$), 3.77 (ddd, *J* = 12.1, 9.7, 1.9 Hz, 1H, NCH$_2$), 2.70 (ddd, *J* = 13.5, 7.9, 1.9 Hz, 1H, NCH$_2$C*H*$_2$), 2.27 (s, 3H, NCH$_3$), 2.20 (ddd, *J* = 12.5, 10.1, 2.2 Hz, 1H, NCH$_2$C*H*$_2$), 1.56 (dd, *J* = 16.3, 2.2 Hz, 1H, CH$_2$Bpin), 1.36 (d, *J* = 16.1 Hz, 1H, CH$_2$Bpin), 1.22 (s, 6H, CH$_3$ x 2), 1.16 (s, 6H, CH$_3$ x 2).

^{13}C NMR (126 MHz, CDCl$_3$) δ ppm 162.4 (CON), 149.4 (ArC), 144.7 (ArC), 133.6 (ArCH), 128.7 (ArCH x 2), 128.0 (ArCH), 126.9 (ArCH), 126.6 (ArCH x 2), 118.2 (ArCH), 116.4 (ArC), 111.6 (ArCH), 85.0 (NCH), 83.4 (Cq x 2), 52.3 (Cq), 42.6 (NCH$_2$), 36.1 (NCH$_2$*C*H$_2$), 34.4 (NCH$_3$), 24.9 (CH$_3$ x 2), 24.8 (CH$_3$ x 2). The signal for *C*H$_2$Bpin was not observed.

^{11}B NMR (160 MHz, CDCl$_3$) δ 34.53

IR ν_{max} (neat/cm^{-1}): 3353, 2975, 1650, 1605, 1484, 1442, 1357, 1141, 967, 848, 751, 699.

Mp: analysis not obtained (amorphous solid).

HRMS : calculated for $C_{25}H_{32}N_2O_3B$ [M + H]$^+$ 419.2505, found 419.2505.

Specific rotation: $[\alpha]_D^{20}$ + 155.7 (c 0.8, CHCl$_3$).

Enantiomeric purity of **5** was determined by HPLC analysis in comparison with authentic racemic material (er = 97:3; **IA** column, 95:5 hexanes: *i*PrOH, 0.5 mL/min, 20 °C, 254 nm).

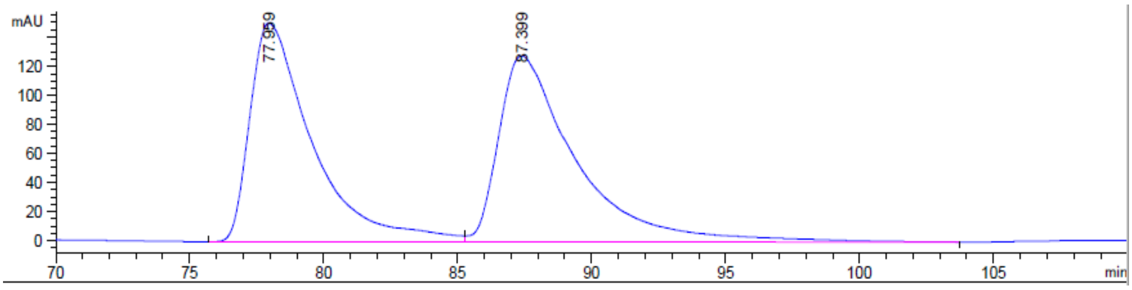

Peak #	RetTime [min]	Type	Width [min]	Area [mAU*s]	Height [mAU]	Area %
1	77.949	BV	2.3000	7.88206e4	488.08936	50.0668
2	87.396	VV	2.7784	7.86104e4	411.93799	49.9332

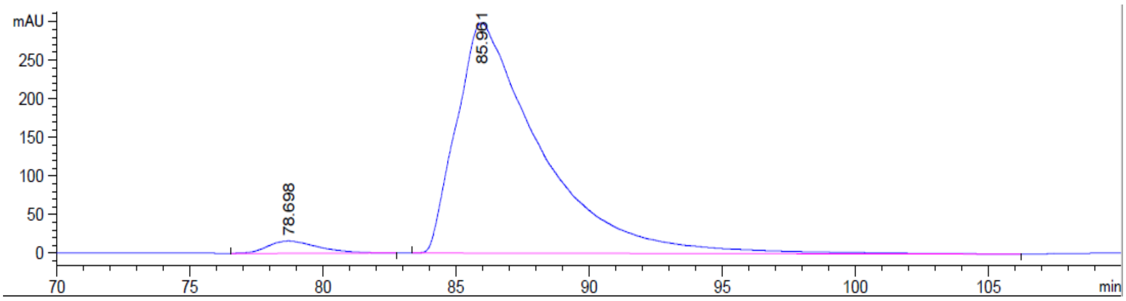

Peak #	RetTime [min]	Type	Width [min]	Area [mAU*s]	Height [mAU]	Area %
1	78.720	BV	2.0117	1.03030e4	72.77448	3.4791
2	85.963	VB	2.8559	2.85841e5	1294.62012	96.5209

^{1}H NMR of 5 (500 MHz, $CDCl_3$)

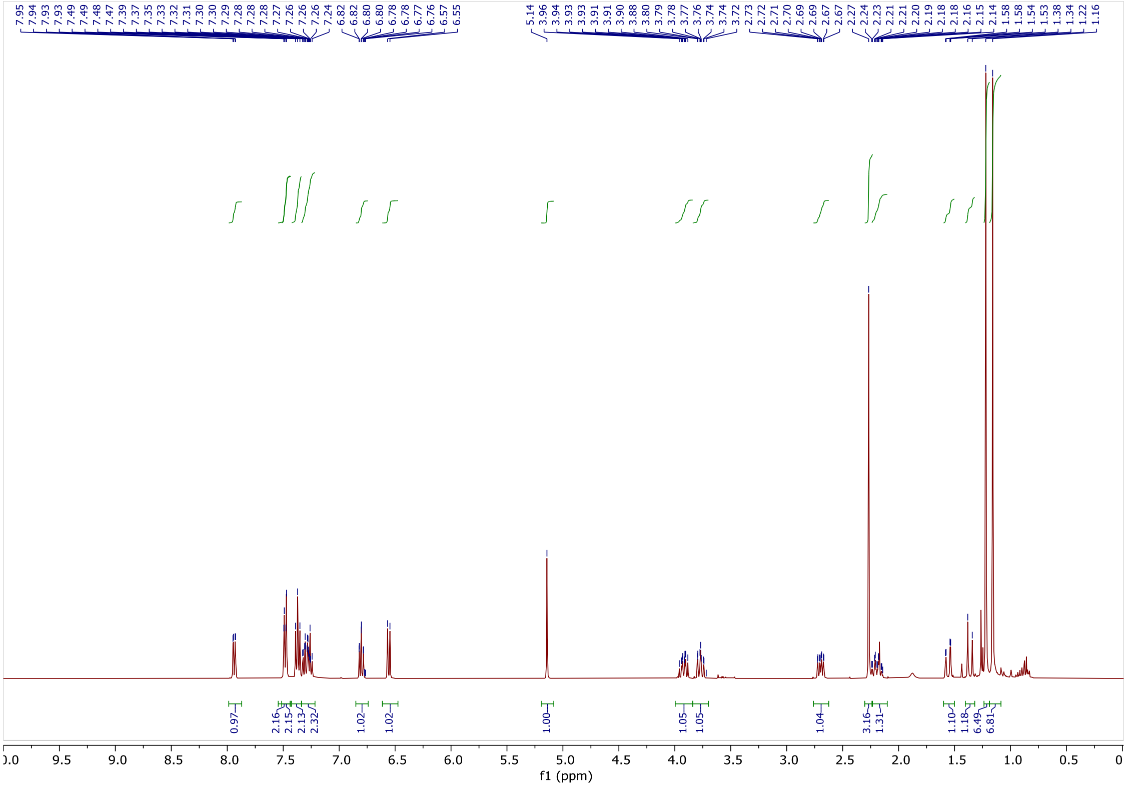

^{13}C NMR of 5 (126 MHz, $CDCl_3$)

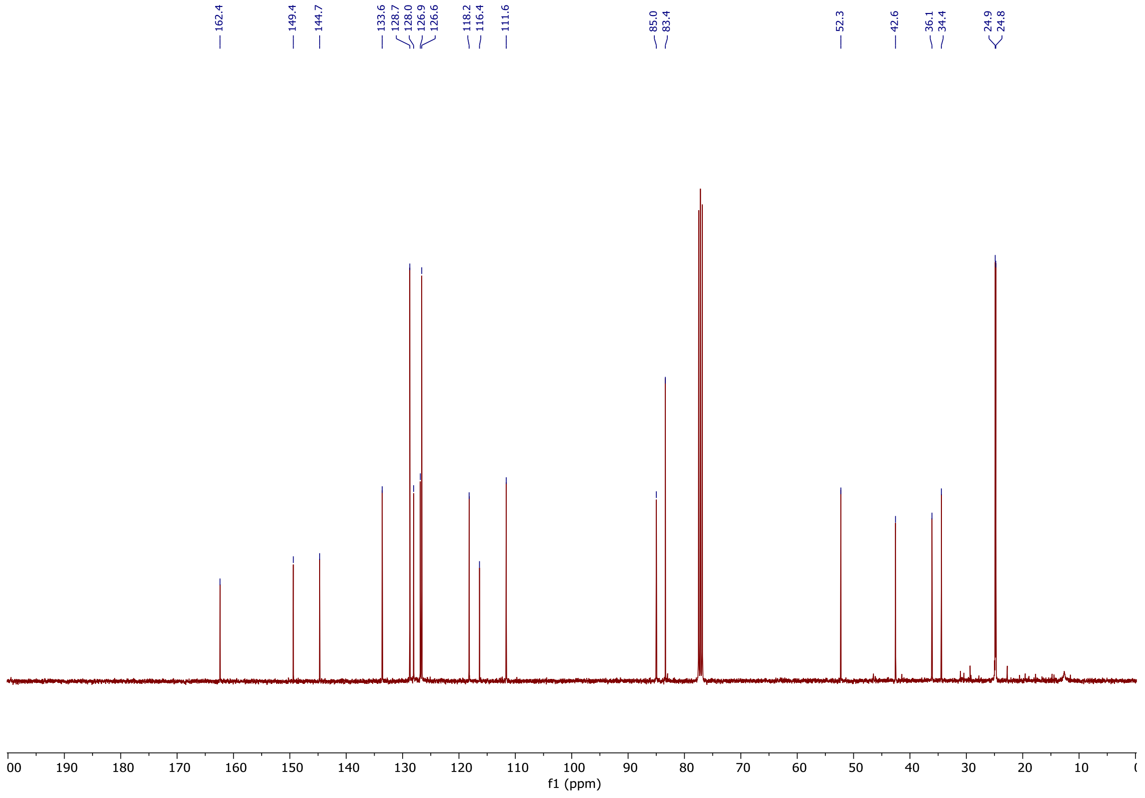

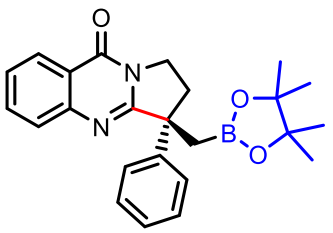

Chemical Formula: $C_{24}H_{27}BN_2O_3$
Molecular Weight: 402.30

(*R*)-3-Phenyl-3-((4,4,5,5-tetramethyl-1,3,2-dioxaborolan-2-yl)methyl)-2,3-dihydropyrrolo[2,1-*b*]quinazolin-9(1H)-one (6)

To a solution of **2b** (44.3 mg, 0.110 mmol, 1 equiv) in CH_2Cl_2 (0.5 mL) at 0 °C was added dropwise a solution of 2,3-dichloro-5,6-dicyano-1,4-benzoquinone (37.3 mg, 0.164 mmol, 1.5 equiv) in CH_2Cl_2 (1.5 mL). The reaction mixture was stirred at 0 °C for 10 min, after which complete conversion of the starting material was observed by TLC analysis. The crude mixture was filtered through a short silica gel plug, diluted with Et_2O and quenched with aqueous saturated $Na_2S_2O_3$. Column chromatography (hexane/Et_2O, 25:75) afforded the title compound as a yellow oil (42 mg, 0.104 mmol, 95% yield).

^{1}H NMR (400 MHz, 400 MHz, $CDCl_3$) δ ppm 8.24 (d, *J* = 7.3 Hz, 1H, ArCH), 7.70 – 7.61 (m, 2H, ArCH x 2), 7.37 (ddd, *J* = 8.1, 6.1, 2.2 Hz, 1H, ArCH), 7.33 – 7.25 (m, 2H, ArCH), 7.25 – 7.19 (m, 2H, ArCH), 7.11 (tt, *J* = 7.3, 1.2 Hz, 1H, ArCH), 4.27 (ddd, *J* = 12.1, 7.6, 2.8 Hz, 1H, NCH_2), 3.74 (ddd, *J* = 12.1, 9.8, 7.3 Hz, 1H, NCH_2), 2.72 – 2.56 (m, 2H, NCH_2CH_2), 1.81 (d, *J* = 16.2 Hz, 1H, CH_2Bpin), 1.70 (d, *J* = 16.2 Hz, 1H, CH_2Bpin), 1.01 (s, 6H, CH_3 x 2), 0.87 (s, 6H, CH_3 x 2).

^{13}C NMR (101 MHz, $CDCl_3$) δ ppm 164.0 (N=CN), 161.3 (CON), 149.6 (ArC), 144.3 (ArC), 134.0 (ArCH), 128.7 (ArCH x 2), 127.6 (ArCH), 127.1 (ArCH), 126.5 (ArCH), 126.2 (ArCH), 126.1 (ArCH x 2), 120.9 (ArC), 83.3 (Cq x 2), 52.5 (Cq), 43.7 (NCH_2), 35.2 (NCH_2CH_2), 24.8 (CH_3 x 2), 24.6 (CH_3 x 2). The signal for CH_2Bpin was not observed.

^{11}B NMR (128 MHz, $CDCl_3$) δ 32.6.

IR ν_{max} (neat/cm^{-1}): 3060, 2977, 1676, 1611, 1469, 1362, 1332, 1166, 846, 774, 696.

HRMS : calculated for $C_{24}H_{28}O_3N_2B$ $[M + H]^+$ 403.2187, found 421.2185.

Specific rotation: $[\alpha]_D^{23}$ + 13.48 (c = 3.3, $CHCl_3$).

Enantiomeric purity of **6** was determined by HPLC analysis in comparison with authentic racemic material (er = 95:5; **Amylose 1** column, 90:10 hexanes: *i*PrOH, 0.5 mL/min, 20 °C, 254 nm).

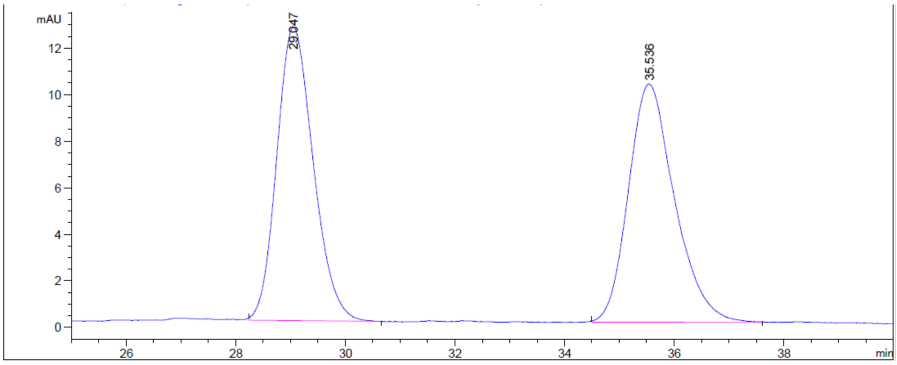

Peak #	RetTime [min]	Type	Width [min]	Area [mAU*s]	Height [mAU]	Area %
1	29.047	BB	0.7149	583.35065	12.59958	49.3581
2	35.536	BB	0.8688	598.52417	10.23291	50.6419

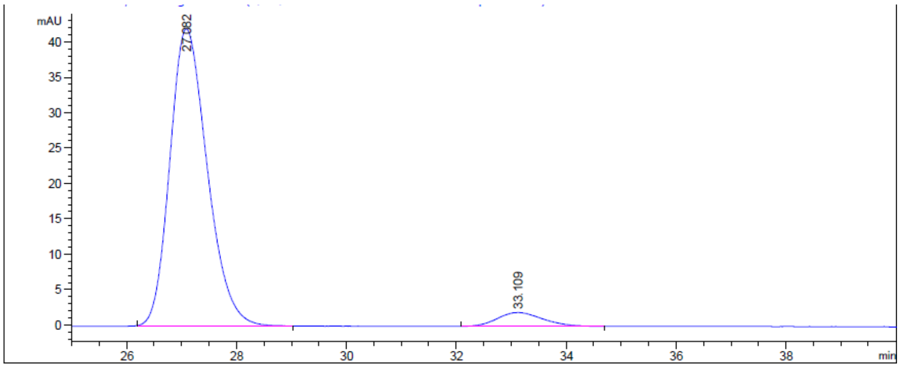

Peak #	RetTime [min]	Type	Width [min]	Area [mAU*s]	Height [mAU]	Area %
1	27.082	BB	0.7392	1989.83618	41.99187	94.6788
2	33.109	BB	0.8819	111.83360	1.94092	5.3212

^{1}H NMR of 6 (400 MHz, $CDCl_3$)

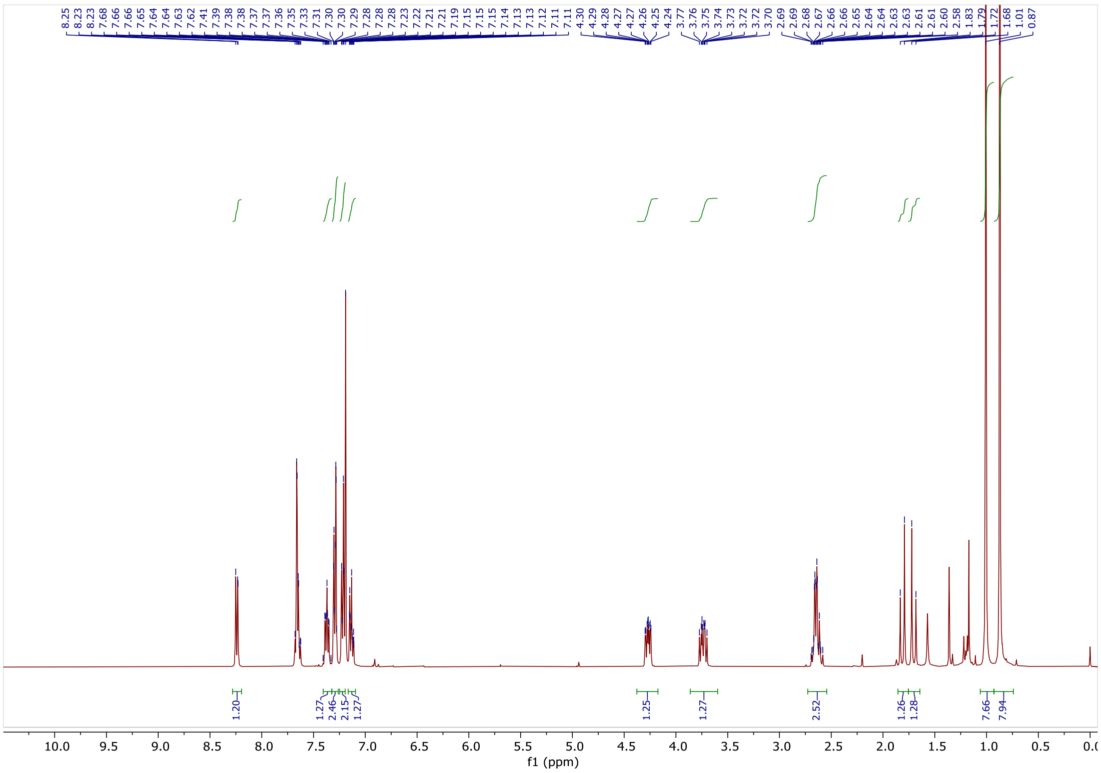

^{13}C NMR of 6 (101 MHz, $CDCl_3$)

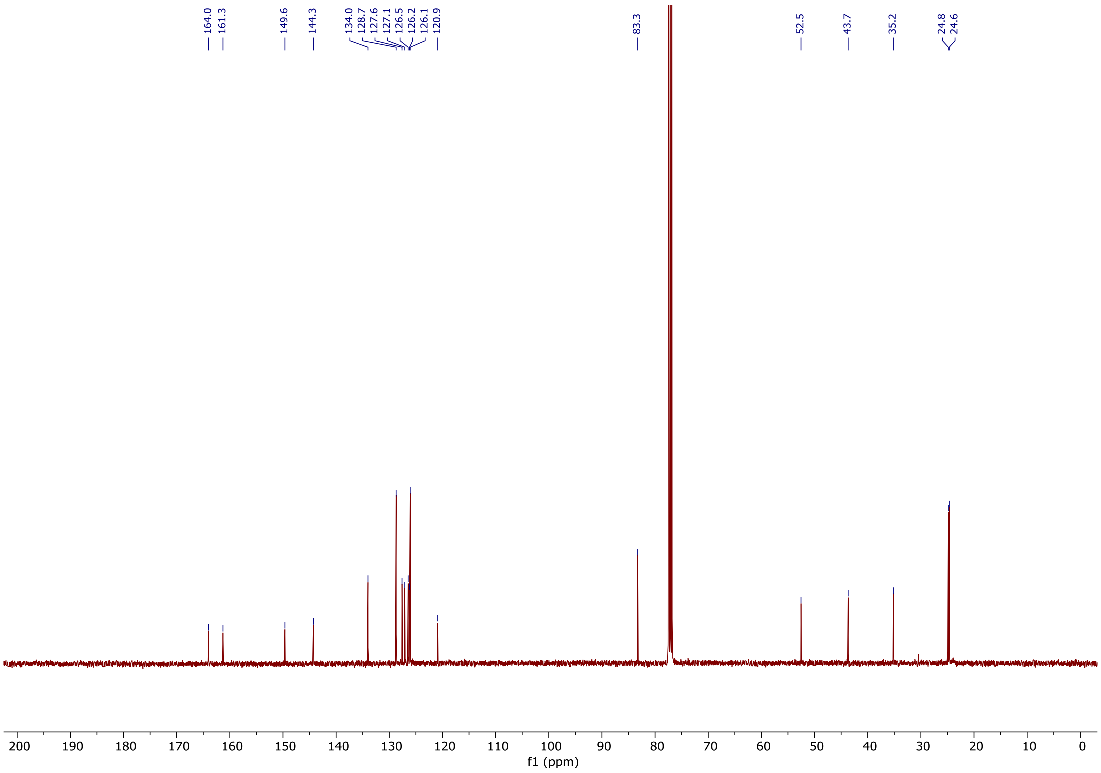

X-ray crystallographic data

Data Collection

X-ray data for compounds **2a derivative**, **2e derivative** and **2f** was collected at a temperature of 100 K on a Rigaku FR-X diffractometer with an Atlas HP6000 detector with MoKα radiation, (λ = 0.71073 Å).

All diffractometers were equipped with an Oxford Cryosystems Cobra nitrogen flow gas system. Data was measured using CrysAlisPro suite of programs.

Crystal structure determinations and refinements

X-ray data were processed and reduced using CrysAlisPro suite of programs. The crystal structures were solved and refined against all F2 values using the SHELX and Olex 2 suite of programs.[2,3] All the non-hydrogen atoms were refined anisotropically. Hydrogen atoms were placed in calculated positions refined using idealised geometries (riding model) and assigned fixed isotropic displacement parameters. Some carbon atoms were found disordered and modelled over two positions were possible. In such cases, C-C bond distances were restrained using DFIX and SADI commands. The atomic displacement parameters (adp) of the disordered atoms have been restrained using the RIGU command.

These data sets can be obtained free of charge via www.ccdc.cam.ac.uk/conts/retrieving.html (or from the Cambridge Crystallographic Data Centre, 12 Union Road, Cambridge CB21EZ, UK; fax: (+44)1223 336033; or deposit@ccdc.cam.ac.uk).

Single Crystal X-ray Diffraction Data for 2a derivative – CCDC: 2063314

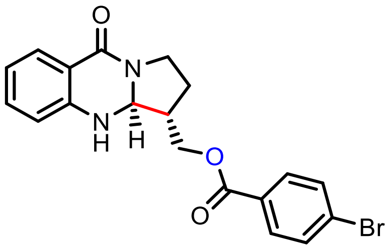

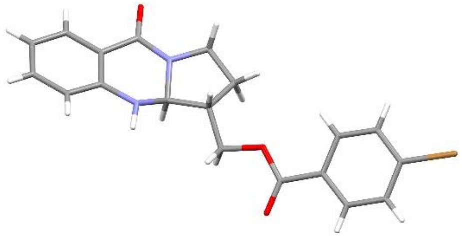

*Crystal data and structure refinement for **2a derivative**:*

Bond precision: C-C = 0.0090 A **Wavelength** = 1.54184

Cell: a=16.5454(4) b=12.6837(3) c=7.8630(4)
alpha=90 beta=90.785(3) gamma=90

Temperature: 100 K

	**Calculated**	**Reported**
**Volume**	1649.95(10)	1649.95(10)
**Space group**	P 21	P 1 21 1
**Hall group**	P 2yb	P 2yb
**Moiety formula**	C19 H17 Br N2 O3	1(C19 H17 Br N2 O3)
**Sum formula**	C19 H17 Br N2 O3	C19 H17 Br N2 O3
**Mr**	401.25	401.25
**Dx,g cm-3**	1.615	1.615
**Z**	4	4
**Mu (mm-1)**	3.582	3.582
**F000**	816.0	816.0
**F000’**	815.39	
**h,k,lmax**	20,15,9	20,15,9
**Nref**	6903[3611]	6584
**Tmin,Tmax**	0.751,0.921	0.713,1.000
**Tmin’**	0.654	

Correction method = # Reported T Limits: Tmin=0.713 Tmax=1.000

AbsCorr = MULTI-SCAN

Data completeness = 1.82/0.95 **Theta(max)** = 76.277

R(reflections) = 0.0374(5528) **wR2(reflections)** = 0.0949(6584)

S = 1.046 **Npar** = 567

Single Crystal X-ray Diffraction Data for 2e derivative – CCDC: 2063315

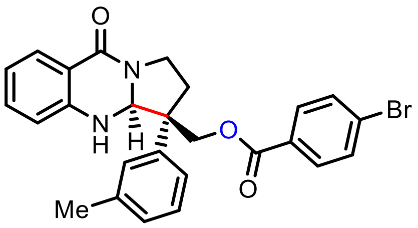

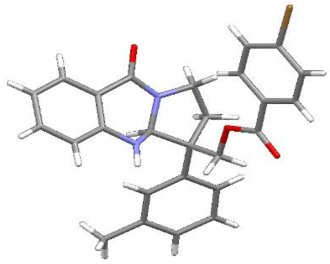

Crystal data and structure refinement for ***2e derivative****:*

Bond precision: C-C = 0.0051 A **Wavelength** = 1.54184

**Cell:**	a=6.87527(10)	b=14.3973(3)	c=11.43140(18)
	alpha=90	beta=93.2536(14)	gamma=90

Temperature: 100 K

	Calculated	Reported
**Volume**	1129.72(3)	1129.72(3)
**Space group**	P 21	P 1 21 1
**Hall group**	P 2yb	P 2yb
**Moiety formula**	C26 H23 Br N2 O3	C26 H23 Br N2 O3
**Sum formula**	C26 H23 Br N2 O3	C26 H23 Br N2 O3
**Mr**	491.36	491.37
**Dx,g cm-3**	1.444	1.445
**Z**	2	2
**Mu (mm-1)**	2.728	2.728
**F000**	504.0	504.0
**F000'**	503.94	
**h,k,lmax**	8,18,14	8,17,14
**Nref**	4760[2478]	4481
**Tmin,Tmax**	0.784,0.963	0.798,1.000
**Tmin'**	0.784	

Correction method = # Reported T Limits: Tmin=0.798 Tmax=1.000

AbsCorr = MULTI-SCAN

Data completeness = 1.81/0.94 **Theta(max)** = 76.566

R(reflections) = 0.0322(4309) **wR2(reflections)** = 0.0768(4481)

S = 1.063 **Npar** = 293

Single Crystal X-ray Diffraction Data for compound 2f – CCDC: 2063313

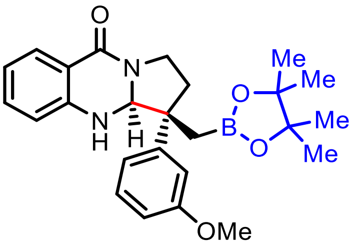

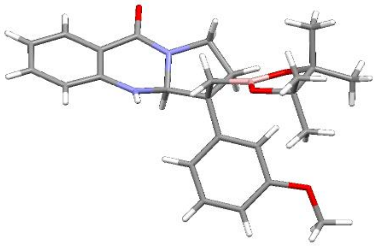

*Crystal data and structure refinement for compound **2f**:*

Bond precision: C-C = 0.0098 A **Wavelength** = 1.54184

**Cell:**	a=6.9009(4)	b=11.915(2)	c=30.636(3)
	alpha=90	beta=90	gamma=90

Temperature: 100 K

	**Calculated**	**Reported**
**Volume**	P 21 21 21	P 21 21 21
**Space group**	P 2ac 2ab	P 2ac 2ab
**Hall group**	P 2yb	P 2yb
**Moiety formula**	2(C25 H31 B N2 O4), C5 H12	C25 H31 B N2 O4, 0.5(C5 H12)
**Sum formula**	C55 H74 B2 N4 O8	C27.50 H37 B N2 O4
**Mr**	940.80	470.40
**Dx,g cm-3**	1.240	1.240
**Z**	2	4
**Mu (mm-1)**	0.651	0.651
**F000**	1012.0	1012.0
**F000’**	1014.91	
**h,k,lmax**	8,14,38	8,15,38
**Nref**	5241[3013]	5064
**Tmin,Tmax**	0.981,0.993	0.464,1.000
**Tmin’**	0.661	

Correction method = # Reported T Limits: Tmin=0.464 Tmax=1.000
AbsCorr = MULTI-SCAN
Data completeness = 1.68/0.97 **Theta(max)** = 75.688
R(reflections) = 0.0792(2975) **wR2(reflections)** = 0.2415(5064)
S = 1.011 **Npar** = 341

References

1. Huang, H.-M.; Adams; R. W.; Procter, D. J. Reductive cyclisations of amidines involving aminal radicals. *Chem. Commun.* **2018**, *54*, 10160–10163.
2. Sheldrick, G. M. Crystal Structure Refinement with SHELXL. *Acta Crystallogr. Sect. A Found. Crystallogr.* **2015**, *71*, 3–8.
3. Dolomanov, O. V.; Bourhis, L. J.; Gildea, R. J.; Howard, J. A. K.; Puschmann, H. OLEX2 : A Complete Structure Solution , Refinement and Analysis Program. *J. Appl. Crystallogr.* **2009**, *42*, 339–341.
